# Negative allosteric modulation of NMDA receptors with GluN2B subunit: synthesis of γ-amino alcohols embedded in a 1-benzoxepine scaffold

**DOI:** 10.1039/d6md00252h

**Published:** 2026-08-04

**Authors:** Felicitas Wendling, Yuan Chang-Halabi, Judith Schmidt, Dirk Schepmann, Julian A. Schreiber, W. Felix Zhu, Marcel Bermudez, Bernhard Wünsch

**Affiliations:** a Institute of Pharmaceutical and Medicinal Chemistry, University of Münster Corrensstraße 48 D-48149 Münster Germany wuensch@uni-muenster.de +49 251 8333311; b GRK 2515, Chemical Biology of Ion Channels (Chembion), Universität Münster Corrensstr. 48 D-48149 Münster Germany

## Abstract

Novel ligands for the ifenprodil binding site of NMDA receptors with GluN2B subunit were designed, synthesized and pharmacologically evaluated. Derived from potent negative allosteric modulators, the γ-amino alcohol pharmacophore was conformationally restricted by embedding it into a 1-benzoxepine ring. Furthermore, the phenolic OH moiety was replaced bioisosterically by a primary or secondary amino moiety as well as by amido groups. The 7- to 9-step synthesis started with 4-aminosalicylic acid comprising a Dieckmann condensation of diester 7 and aminolysis of β-keto esters 8 as key steps. Membrane fragments of mouse fibroblast cells stably transfected with GluN1-1a and GluN2B subunits of the NMDA receptor and [^3^H]ifenprodil were used in radioligand receptor binding studies. In the benzylpiperidine d-series, the affinity towards GluN2B subunit-containing NMDA receptors increased from benzylamine 11d to primary amine 12d to formamide 13d (*K*_i_ = 278 nM). Larger acyl moieties reduced the NMDA receptor affinity. Removal of the benzylic OH moiety led to allylamines 18b and 18d with considerable GluN2B affinity. However, their σ_1_ affinity was even higher than their GluN2B affinity. It was concluded that the benzylic OH moiety is not essential for high GluN2B affinity, but is important for selectivity over σ_1_ receptors. The novel class of 1-benzoxepine-based negative allosteric modulators of the NMDA receptor revealed promising lipophilicity (*e.g.*, 13b: log *D*_7.4_ = 1.99). Binding at human serum albumin correlated with the lipophilicity (*e.g.*, 13b: PPB = 81%). Most of the 1-benzoxepines showed more than 65% metabolic stability during incubation with mouse liver microsomes and NADPH for 90 min.

## Abstract 2

High-nanomolar affinity towards NMDA receptors with GluN2B subunit.

## Graphical abstract

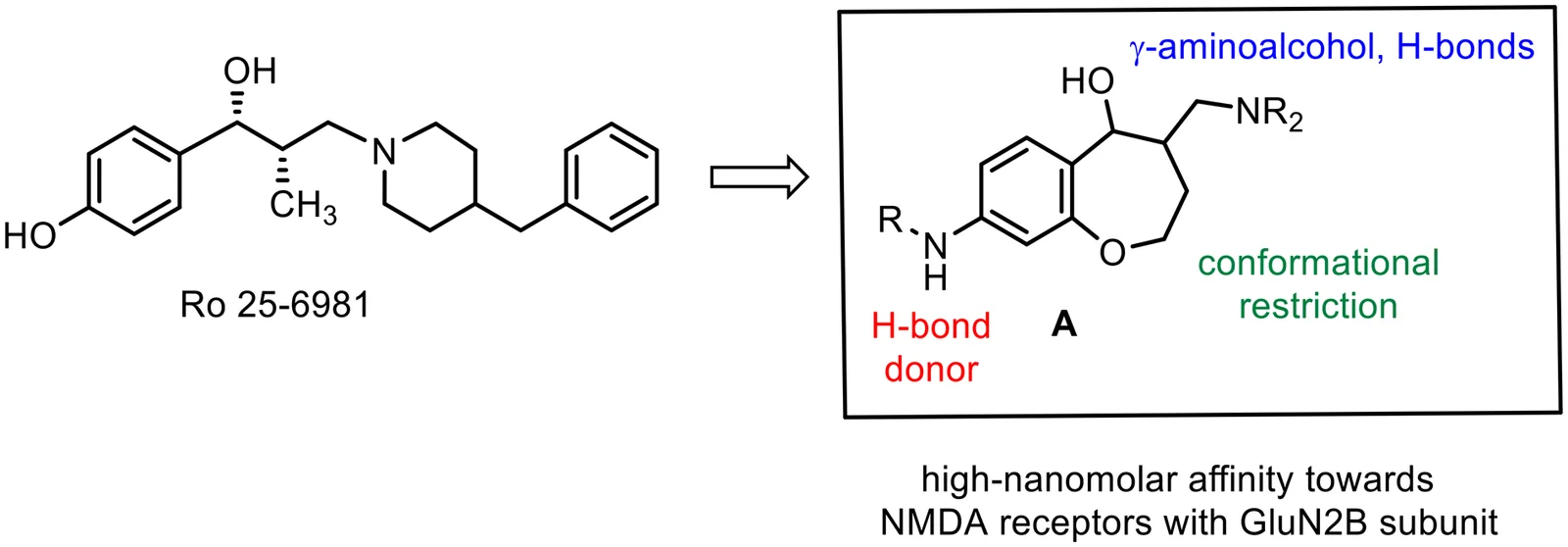

## 1. Introduction

Decarboxylation of (*S*)-glutamate, the main excitatory neurotransmitter in the central nervous system of mammals, leads to γ-aminobutyric acid (GABA), the main inhibitory neurotransmitter. (*S*)-Glutamate activates eight metabotropic (mGlu1–8) and three ionotropic glutamate receptor subtypes. The ionotropic glutamate receptor subtypes are named according to their prototypical agonists *N*-methyl-d-aspartate (NMDA), 2-amino-3-(3-hydroxy-5-methylisoxazol-4-yl)propionic acid (AMPA) and kainate receptors.^1–3^

Activation of NMDA receptors requires not only the presence of (*S*)-glutamate, but also the presence of the co-agonist glycine and the removal of the Mg^2+^ block by slight depolarization of the membrane *via* AMPA activation.^3^ Since several events are necessary to activate NMDA receptors, it is involved in complex neuronal processes such as long-term potentiation (LTP), long-term depression (TTD) and synaptic plasticity, which are associated with learning and memory.^4,5^

On the contrary, strong or permanent overactivation of the NMDA receptor can result in a vicious circle (circulus vitiosus) induced by massive influx of Ca^2+^ ions subsequently leading to cell death and massive release of (*S*)-glutamate, which again activates NMDA receptors. This process, termed excitotoxicity,^6^ is postulated to be involved in acute events, such as stroke, brain injury and epileptic seizures as well as in chronic neurodegenerative diseases, including multiple sclerosis, Alzheimer's and Parkinson's disease.^2,6–8^ Therefore, drugs, which are able to interrupt this vicious circle, are of high interest for potential treatment and slowing down of neurodegenerative processes.

The NMDA receptor is formed as a dimer of dimers, where each dimer possesses one GluN1 subunit presenting the glycine binding site. The remaining subunits needed for tetramerization are various combinations of GluN2A-D or GluN3A-B subunits, that possess binding sites for (*S*)-glutamate (GluN2) or glycine (GluN3). Consequently, tetramers formed only by GluN1 and GluN3 subunits do no longer recognize (*S*)-glutamate, but behave as excitatory glycine receptors.^9^ Therefore, NMDA receptors with GluN3 subunits were not considered in this project. Whereas only one gene exists for the GluN1 subunit, four different genes encode for the subunits GluN2A-D. The different GluN2 subunits determine the properties and gating characteristics of the NMDA receptor associated ion channel.^10–13^

NMDA receptors containing two GluN1 and two GluN2B subunits were in the focus of this study. In the forebrain of adult individuals, GluN2A and GluN2B subunits are expressed predominantly, whereas GluN2C (cerebellum) and GluN2D subunits (diencephalon, brain stem, cerebellum) are less prominently expressed.^14^ A particularly high density of NMDA receptors with GluN2B subunits was detected in the cortex, thalamus, striatum, and hippocampus.^12^ GluN1/GluN2B-containing NMDA receptors reveal fast deactivation kinetics, high Mg^2+^ sensitivity, high Ca^2+^ permeability, and high conductance. In contrast to NMDA receptors with GluN2A subunit, GluN2B subunit containing NMDA receptors are less sensitive against H^+^ and Zn^2+^ ions.^14,15^ NMDA receptors containing GluN1 and GluN2B subunits form a special binding site located at the subunit interface within the N-terminal domain. Negative allosteric modulators (NAM) addressing this binding site represent interesting drug candidates, as they display less side effects than prototypical non-selective open-channel blockers such as phencyclidine, (*S*)-ketamine and (+)-MK-801.^16–18^

**Fig. 1:**
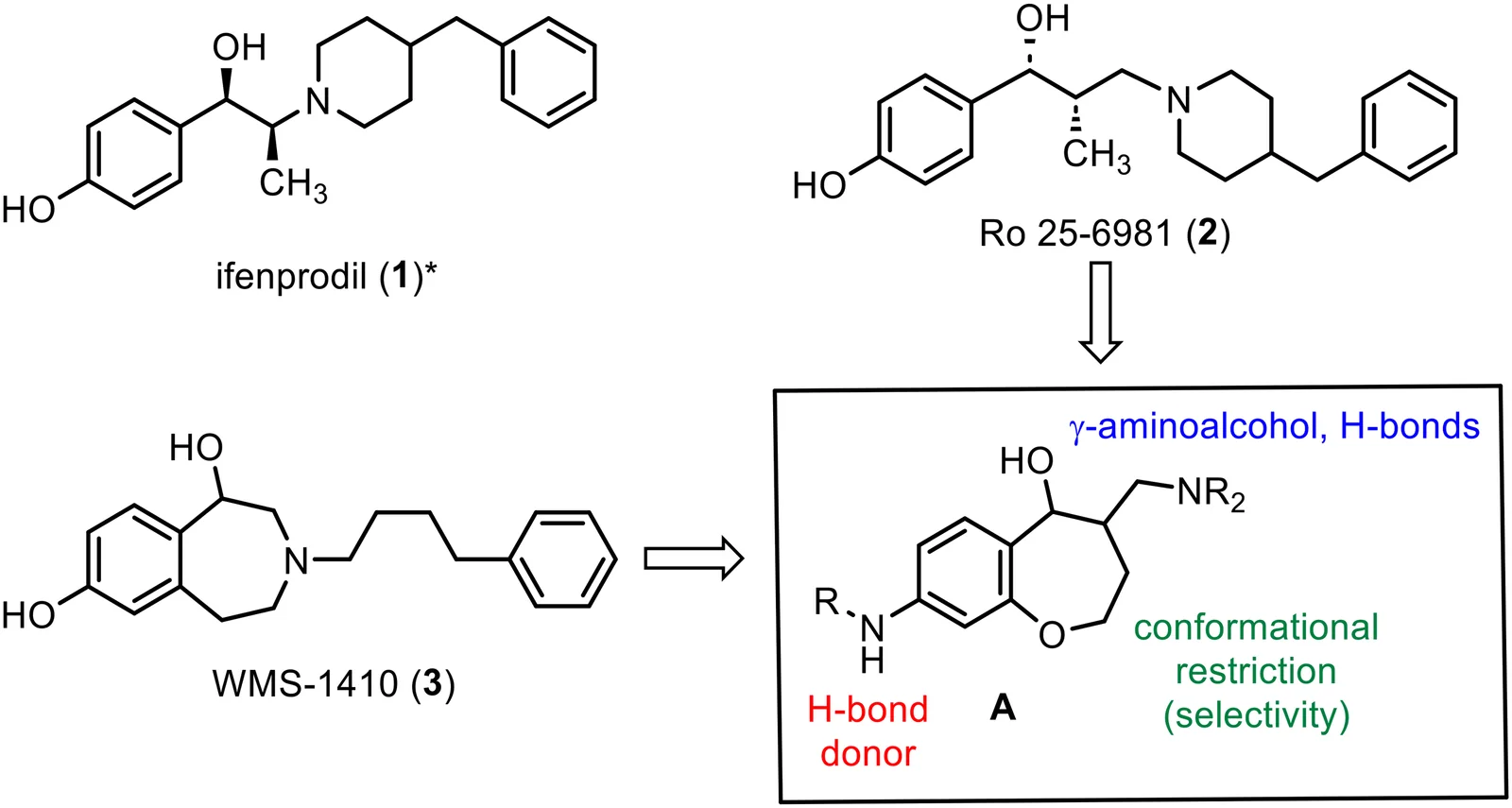
Design of ligands A to address the ifenprodil binding site of NMDA receptors containing the GluN2B subunit.

The special binding site of GluN2B subunit-containing NMDA receptors was named after the first discovered binding NAM ifenprodil (1).^18,19^ (Fig. 1) The unlike-configured racemic mixture of the β-amino alcohol ifenprodil is used in Japan as a cerebral vasodilator, which can be explained by its additional antagonism at α_1_ receptors. Nevertheless, in animal models of Alzheimer's disease and neuropathic pain, ifenprodil showed beneficial outcomes rendering it as a prototype for drug development.^20–22^

The interactions of ifenprodil with its binding site have been analyzed after co-crystallization of the isolated N-terminal domain as well as the full heterotetrameric GluN2B subunit-containing NMDA receptor.^11,23^ Key interactions of ifenprodil with the ifenprodil binding site are three H-bonds between the phenolic OH group of ifenprodil and the terminal carboxy moiety of Glu236, the benzylic OH group and the C=O backbone of Ser 132 as well as between the protonated piperidine ring and the terminal carbamoyl moiety of Gln110. π/π and hydrophobic interactions stabilize the terminal phenyl moiety within the binding pocket. Important binding partners forming π/π and hydrophobic interactions are Tyr109 (GluN1a), Leu135 (GluN1a), Ile111 (GluN2B), Phe114 (GluN2B), Phe176 (GluN2B) and Pro177 (GluN2B). According to docking studies, MD simulations and site-directed mutagenesis, the negative allosteric modulator Ro 25-6981 (2) and WMS-1410 (3) exhibit comparable interactions within the ifenprodil binding site *in silico* and show similar reduction of the inhibitory effect at tested mutants GluN2B F114A, GluN2B F176A and GluN2B E236A^24^ (Fig. 1).

The low bioavailability due to fast conjugation of the phenol with glucuronic acid^25^ together with the low selectivity against other targets^26–29^ limit the broad application of ifenprodil as a neuroprotective drug in Alzheimer's disease. In order to keep the key interactions of ifenprodil at its binding site, but improve the bioavailability and selectivity, 1-benzoxepin-5-ols of type A were designed. In A, the phenolic OH group of ifenprodil was replaced by an N-based H-bond donor. The resulting secondary amides and amines should no longer be prone to glucuronidation, but keep the H-bond interaction with Glu236. Secondly, the CH_3_ group of ifenprodil (1) and Ro 25-6981 (2) was embedded in a conformationally restricted seven-membered 1-benzoxepine ring. Embedding the piperidine N-atom of ifenprodil into the 3-benzazepine ring as in WMS-1410 (3) had increased the selectivity over σ_1_, σ_2_, α_1_, α_2_ and 5-HT receptors.^29^ Finally, the γ-amino alcohol substructure of Ro 25-6981 was retained, which should allow the formation of crucial H-bonds with the C(=O)NH_2_ moiety of Gln110.

## 2. Results and discussion

### 2.1. Synthesis

**Scheme 1:**
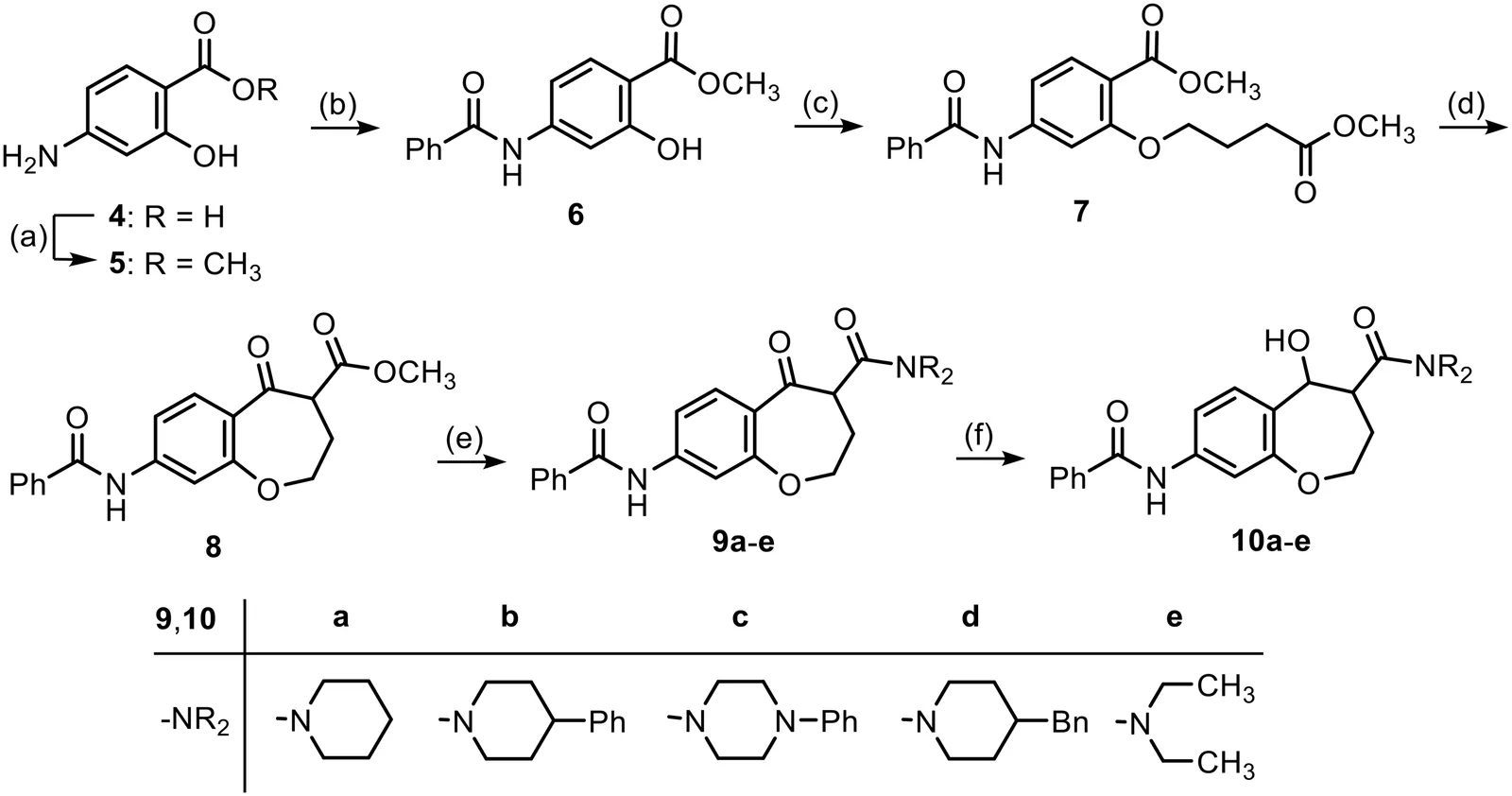
Synthesis of β-hydroxy amides 10. Reagents and reaction conditions: (a) CH_3_OH, H_2_SO_4_, reflux, 12 h, 76%. (b) Benzoyl chloride, THF, NEt_3_, rt, 2 h, 74%. (c) BrCH_2_CH_2_CH_2_CO_2_CH_3_, DMF, K_2_CO_3_, 80 °C, 16 h, 84%. (d) LiHMDS, THF, 0 °C, then rt, 16 h, 51%. (e) R_2_NH, DMAP, 120 °C, 16–32 h, 33–90%. (f) NaBH_4_, CH_3_OH, rt, 1–4 h, 60–99% (mixture of diastereomers); for 10b and 10e*cis*- and *trans*-configured diastereomers were separated.

The synthesis of 1-benzoxepines of type A started with esterification of 4-aminosalicylic acid (4) with methanol (Scheme 1). The resulting methyl ester 5 was benzoylated and subsequently alkylated with methyl 4-bromobutyrate to afford the diester 7. The subsequent Dieckmann condensation of diester 7 was performed upon slow addition of three equivalents of LiHMDS (10 mL h^−1^ with a 1 M solution in THF) and stirring the reaction mixture at room temperature for 16 h. The 1-benzoxepine-4-carboxylate 8 was obtained in 51% yield as 67 : 33 mixture of keto and enol tautomers according to the ^1^H NMR spectrum (CDCl_3_).

Reaction of the β-keto ester 8 with secondary amines at 120 °C led to the β-keto amides 9, which were reduced with NaBH_4_ to afford the β-hydroxy amides 10. During the reduction of β-keto amides 9, a novel center of chirality was established. The produced β-hydroxy amides 10 were formed as mixtures of *cis*- and *trans*-configured diastereomers. For the 4-phenylpiperidide 10b and the diethylamide 10e, *cis*- and *trans*-configured diastereomers could be separated and characterized (Scheme 1).

**Scheme 2:**
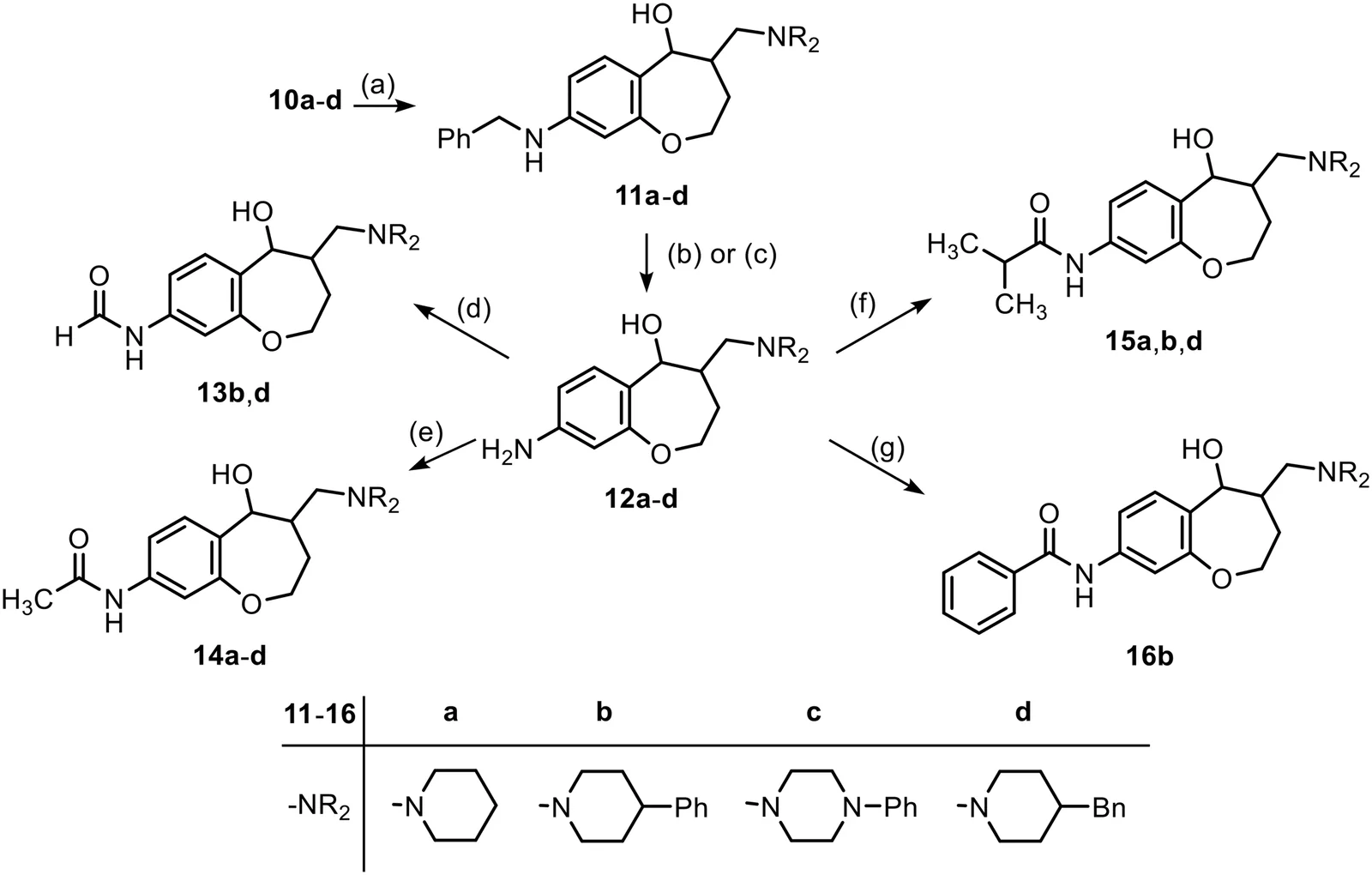
Synthesis of γ-amino alcohols. Reagents and reaction conditions: (a) LiAlH_4_, THF, reflux, 4–6 h, 31–89%. (b) 11a, 11b, *cis*-11d: H_2_ (balloon), Pd/C, CH_3_OH, rt, 24–48 h, 45% (12a), 47% (12b), 46% (*cis*-12d). (c) 11c, 11d: NH_4_ HCO_2_, Pd/C, CH_3_OH, 60 °C, 4–9 h, 36% (12c), 21% (12d). (d) HCO_2_H, Ac_2_O, THF, 30 °C, 2 h, 37% (13b), 23% (13d). (e) Ac_2_O, CH_2_Cl_2_, rt, 1–3 h, 35–65%. (f) Isobutyric anhydride, CH_3_OH, 80 °C, 20 min or 3 h, 28–72%. (g) PhCOCl, THF, NEt_3_, rt, 1 h, 58%.

LiAlH_4_ reduction of the β-hydroxy amides 10a–d provided the γ-amino alcohols 11a–d with a benzyl protective group at the aromatic amino moiety. *cis*- and *trans*-configured γ-amino alcohols were obtained with a ratio of 70 : 30 (11a) and 80 : 20 (11b–d). Two methods were applied for the removal of the *N*-benzyl protective group: H_2_ and Pd/C removed the benzyl group from 11a, 11b, 11d and a transfer hydrogenolysis with NH_4_HCO_2_ and Pd/C removed the benzyl moiety from 11c and 11d. Both methods led to comparable yields of primary amines 12. The resulting primary amines 12 allowed the introduction of diverse acyl substituents. Reaction of primary amines 12b and 12d with HCO_2_H and Ac_2_O led to the formyl derivatives 13b and 13d. Ac_2_O and isobutyric anhydride converted the primary amines 12 into acetamides 14 and isobutyramides 15, respectively. Benzamide 16b was obtained by benzoylation of primary amine 12b with benzoyl chloride (Scheme 2).

**Scheme 3:**
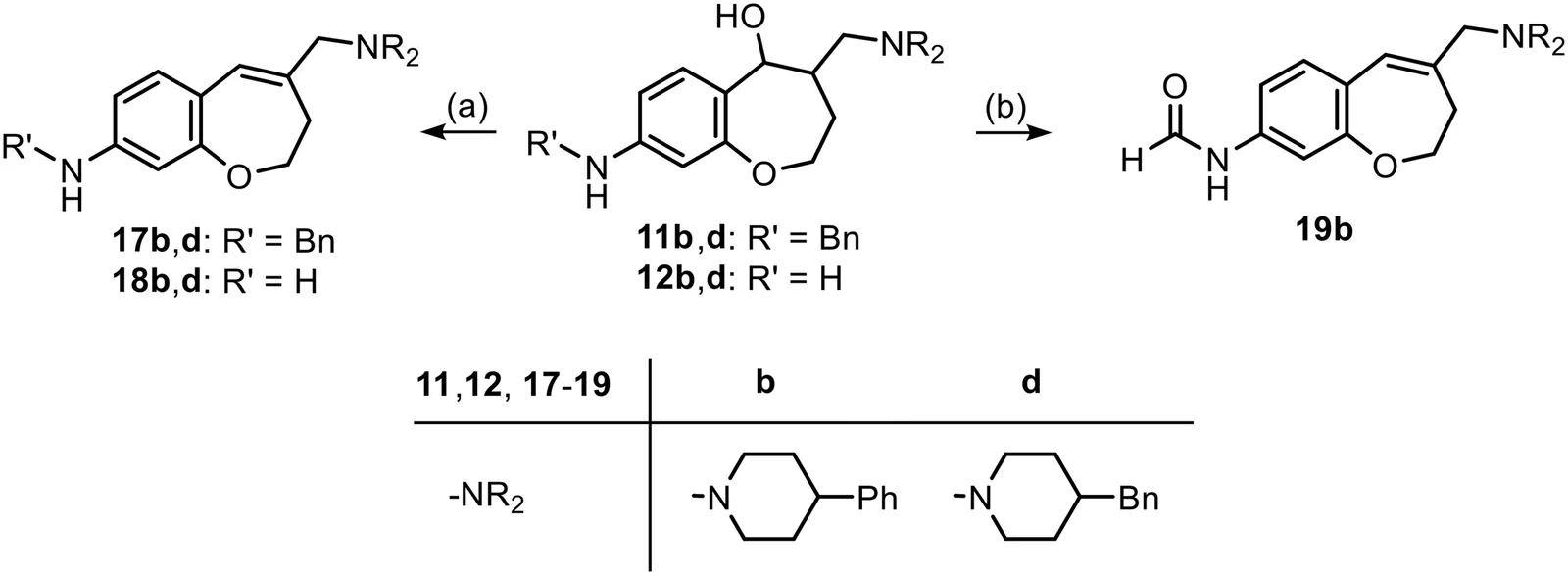
Synthesis of 2,3-dihydro-1-benzoxepines 17–19. Reagents and reaction conditions: (a) H_2_SO_4_, THF, rt, 24 h, 13–68%. (b) 12b: HCO_2_H, rt, 25 min, 64%.

In order to investigate the importance of the benzylic OH moiety, 1-benzoxepines without benzylic OH group were prepared. H_2_SO_4_ was used to eliminate water from the benzylamines 11b and 11d as well as the primary amines 12b and 12d, to achieve the 2,3-dihydro-1-benzoxepines 17b,d and 18b,d. The formamide 19b was obtained upon treatment of the primary amine 12b with HCO_2_H. For the development of structure affinity relationships, the 4-phenylpiperidino and the 4-benzylpiperino groups represented the preferred γ-amino moiety (Scheme 3).

### 2.2. Receptor affinity

The affinity of the synthesized γ-amino alcohols 11–16 and the allylamines 17–19 towards the ifenprodil binding site of GluN2B subunit-containing NMDA receptors was determined in competitive radioligand receptor binding studies. Membrane fragments from L(tk-) mouse fibroblast cells stably transfected with GluN1-1a and GluN2B subunits of the NMDA receptor served as receptor material and [^3^H]ifenprodil as radioligand.^30^ In order to evaluate the selectivity, the affinity at σ_1_ (guinea pig brain membranes, [^3^H](+)-pentazocine) and σ_2_ receptors (rat liver membrane preparations, [^3^H]di-*o*-tolylguanidine) was also recorded in competitive receptor binding studies.^31–33^ The affinity of selected compounds to the PCP binding site within the ion channel pore of the NMDA receptor was determined with pig brain cortex membrane preparations and [^3^H]MK-801 as radioligand.^34,35^ The results of the competitive receptor binding studies are summarized in Table 1.

**Table 1:** Affinities of the γ-amino alcohols 11–16 and the allylamines 17–19 towards the ifenprodil binding site and the PCP binding site of the NMDA receptor as well as σ_1_ and σ_2_ receptors. For each receptor subtype, affinity data of reference compounds are included

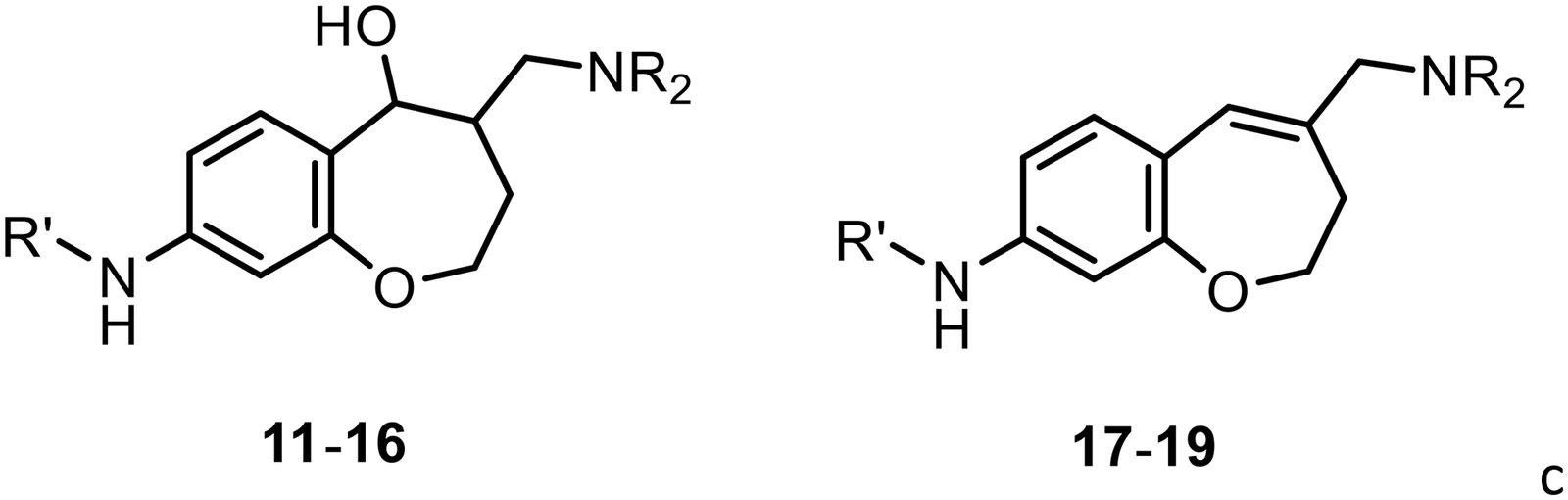							
Compd.	R′	NR_2_	Ratio *cis*–*trans*	*K*_i_ [nM] (*n* = 2)^a^			
				GluN2B	PCP	σ_1_	σ_2_
11a	PhCH_2_	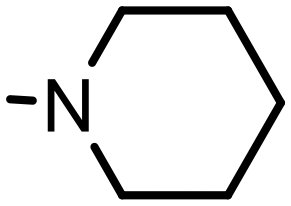	70 : 30	889	20%	140	22%
11b	PhCH_2_	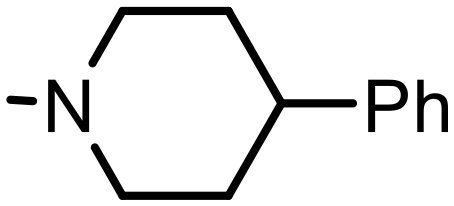	80 : 20	256 ± 69	—	177 ± 22	1600
11c	PhCH_2_	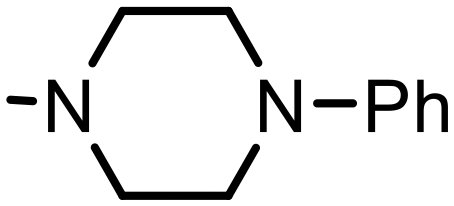	45 : 55	615	—	2850	0%
11d	PhCH_2_	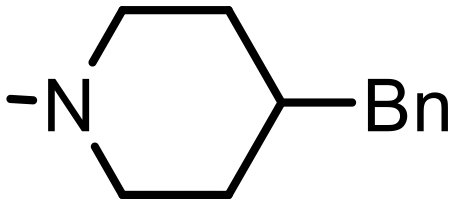	80 : 20	857	—	324	11%
12a	H	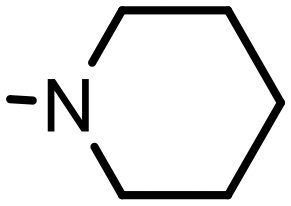	63 : 37	9%	0%	20%	0%
12b	H	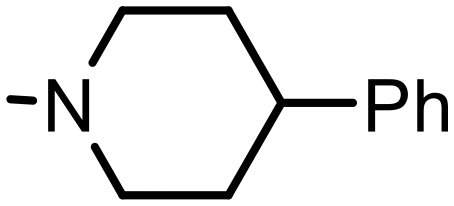	45 : 55	969	0%	121	472
12c	H	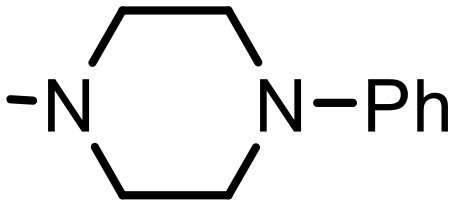	45 : 55	0%	—	897	1720
12d	H	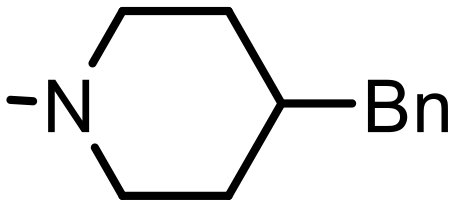	100 : 0	689	—	877	1160
13b	HCO	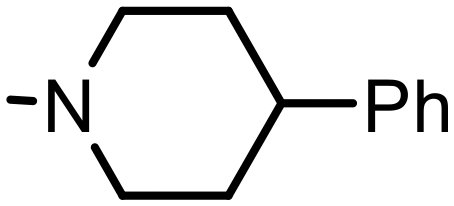	80 : 20	8%	—	362	1960
13d	HCO	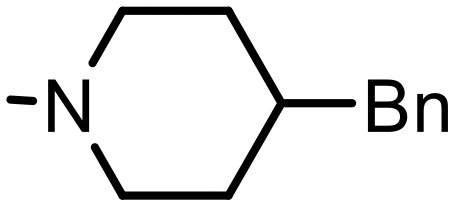	20 : 80	278 ± 28	—	145 ± 35	827
14a	H_3_CCO	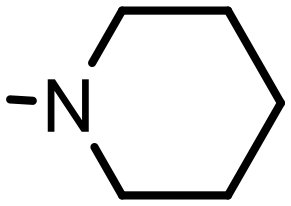	40 : 60	0%	—	0%	1890
14b	H_3_CCO	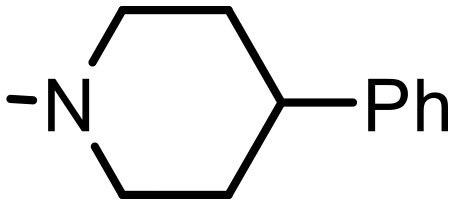	70 : 30	17%	27%	1010	0%
14c	H_3_CCO	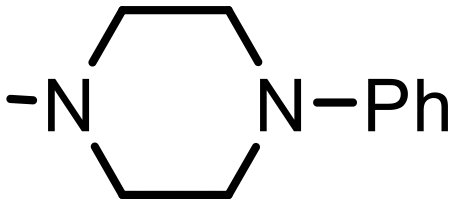	33 : 67	0%	—	0%	3%
14d	H_3_CCO	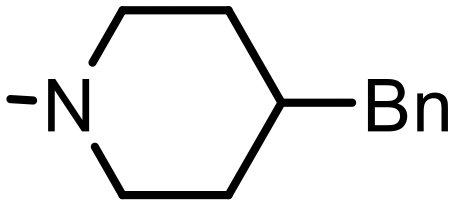	75 : 25	5%	—	25%	10%
15a	iPrCO	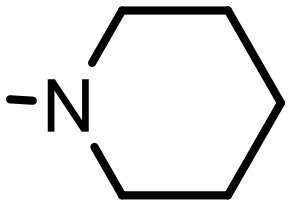	60 : 40	21%	11%	18%	15%
15b	iPrCO	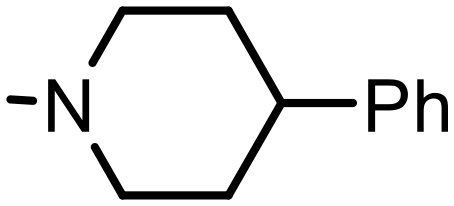	17 : 83	586	17%	178	1%
16b	PhCO	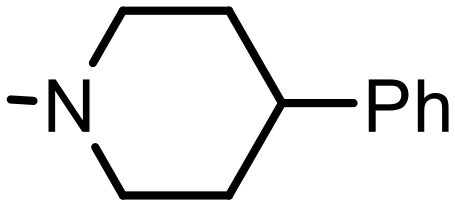	100 : 0	1400	—		
16d	PhCO	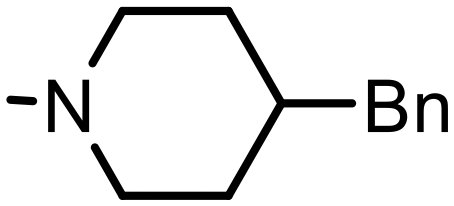	80 : 20	1640	—	1230	2670
17b	PhCH_2_	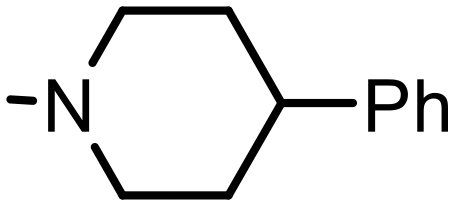	—	478	—	224	1300
17d	PhCH_2_	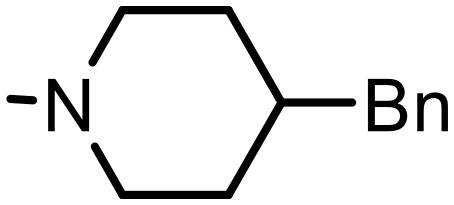	—	10%	—	1000	1170
18b	H	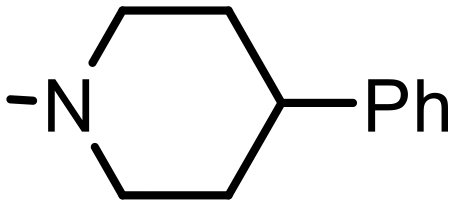	—	489	—	61 ± 16^a^	—
18d	H	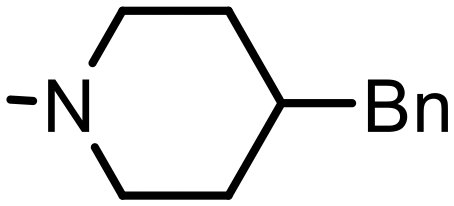	—	602	—	133 ± 26^a^	—
19b	HCO	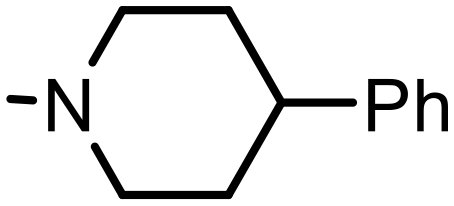	—	1100	—	188	1960
(1*R*,2*S*)-Ifenprodil (1)^36^			—	14.4 ± 3.3^a^	—	22 ± 3^a^	4.6 ± 2.3^a^
(*R)*-WMS-1410 (3)			—	84 ± 18^a^	—	194	>10 μM
Memantine			—	—	429 ± 42^a^	—	—
(+)-pentazocine				—	—	5.4 ± 0.5^a^	—
Di-*o*-tolylguanidine			—	—	—	71 ± 8^a^	54 ± 8^a^

a Due to the rather low affinity of most of the test compounds, *K*_i_ values were recorded as duplicates and are given without SEM values. Exceptions with SEM values (*n* = 3) are the σ_1_ affinity data for 18b and 8d and the affinity data for the reference compounds. Values in % represent the inhibition of the radioligand binding at a test compound concentration of 1 μM.

1-Benzoxepines with the 4-benzylpiperidino moiety (**d**-series) were the preferred ligands, since the prototypical GluN2B antagonists ifenprodil (1) and Ro 25-6981 (2) contain this pharmacophore elements as well. For these 4-benzylpiperidines, the GluN2B affinity increased from the benzylamine 11d (*K*_i_ = 857 nM) over the primary amine 12d (*K*_i_ = 689 nM) up to the formamide 13d (*K*_i_ = 278 nM). Larger acyl moieties at the aromatic amino group were less tolerated, *e.g.*, acetamide 14d and benzamide 16d. Elimination of water from primary amine 12d led to the allylamine 18d with almost the same GluN2B affinity (*K*_i_ = 602 nM).

Shortening the benzyl substituent by one CH_2_ moiety resulted in 4-phenylpiperidine substituted GluN2B ligands (**b**-series). Most 4-phenylpiperidines (**b**-series) are equal or less potent than the corresponding 4-benzylpiperidines (**d**-series), *e.g.*, *K*_i_(12b) > *K*_i_(12d); *K*_i_(13b) > *K*_i_(13d). As an exception, the 4-phenylpiperidines 11b and 17b with benzylamino moiety show higher GluN2B affinity than the analogous 4-benzylpiperidines 11d and 17d. Bioisosteric replacement of the 4-phenylpiperidino moiety by a 4-phenylpiperazino moiety (**c**-series, CH/N-exchange) reduced the interactions with the ifenprodil binding site of GluN2B subunit-containing NMDA receptors. The same applies for the **a**-series of compounds containing the piperidino moiety without planar lipophilic phenyl or benzyl group.

In general, the affinity towards GluN2B subunit-containing NMDA receptors was only moderate. It is assumed that the OH moiety of ifenprodil (1), Ro 25-6981 (2) and WMS-1410 (3) is a more efficient H-bond donor than the NH-moieties of benzylamines 11 and 17, primary amines 12 and 18 or amides 13–16 and 19. The highest GluN2B affinity was detected for 11b (*K*_i_ = 256 nM) and 13d (*K*_i_ = 278 nM). In this series of ligands, the small formamido moiety of 13d represents the best bioisosteric replacement of the phenolic OH moiety.

As the lead compound ifenprodil reveals high affinity towards both σ receptor subtypes, the σ_1_ and σ_2_ affinity of the γ-amino alcohols 11–16 and allylamines 17–19 was recorded as well. The σ_1_ and σ_2_ affinity of the γ-amino alcohols 11–16 is in the micromolar to high nanomolar range (*K*_i_ > 100 nM). Particularly noteworthy are the σ_1_ and σ_2_ affinities of γ-amino alcohols 11b (*K*_i_(σ_1_) = 177 nM, *K*_i_(σ_2_) = 31 nM) and the σ_1_ affinity of 13d (*K*_i_ = 145 nM), which even exceed the GluN2B affinity of these ligands. The highest σ_1_ affinity was observed for the primary amines 18b and 18d without benzylic OH moiety in 5-position. The high σ_1_ affinity of both compounds is in good accordance with reported σ_1_ pharmacophore models.^37–39^

The GluN2B, σ_1_ and σ_2_ affinity of the diastereomerically pure β-hydroxy amides *cis*-10b, *cis*-10e, and *trans*-10e was also tested, although the crucial basic amino group in the side chain was missing. As expected, at concentrations of 1 μM, the amides could not compete considerably with the respective radioligands in the assays indicating very low affinity towards these receptors.

### 2.3. Physicochemical and pharmacokinetic properties of selected ligands

The lipophilicity, plasma protein binding and metabolic stability of selected γ-amino alcohols 11–16 and allylamines 18 were investigated. log *D*_7.4_ values were recorded as an indicator for the ligand's lipophilicity. In order to get first insight into the pharmacokinetic behavior of the ligands, the plasma protein binding and the metabolic stability were recorded (Table 2).

**Table 2:** Physicochemical and pharmacokinetic properties of selected 1-benzoxepines

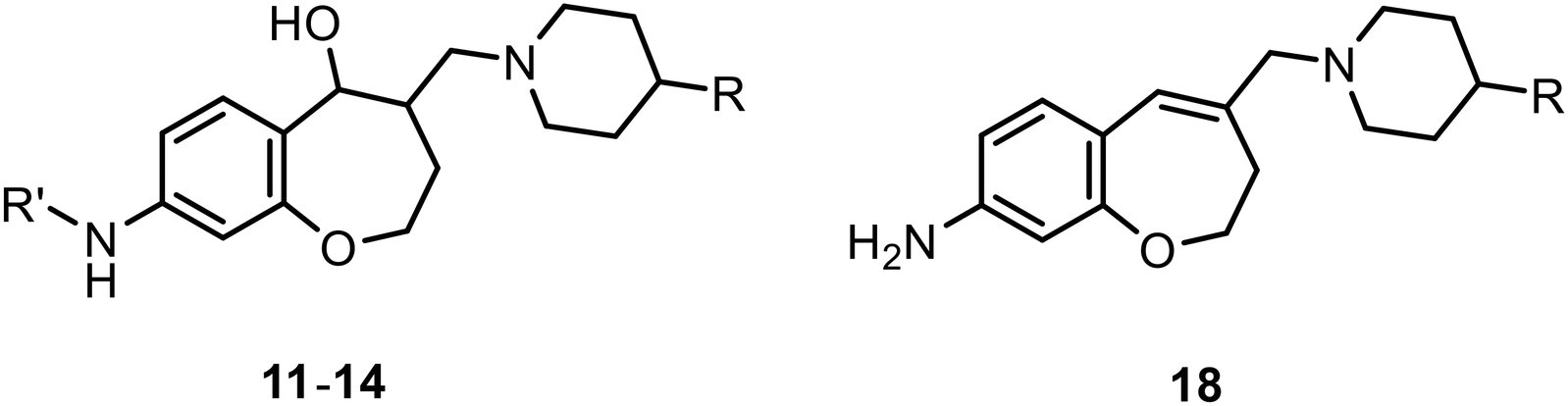					
Compd.	R′	R	log *D*_7.4_ ± SD (*n* = 3)	Plasma protein binding ± SD (%, *n* = 3)^a^	Metabolic stability ± SD (%, *n* = 3)^b^
11a	Bn	H	1.64 ± 0.03	87 ± 0.8	14 ± 5
11d	Bn	Bn	3.81 ± 0.02	>98	66 ± 5
12a	H	H	−0.18 ± 0.03	36 ± 0.8	100
12b	H	Ph	1.38 ± 0.05	72 ± 0.8	87 ± 8
13b	HC=O	Ph	1.99 ± 0.03	81 ± 0.9	66 ± 2
14a	H_3_C–C=O	H	−0.10 ± 0.05	42 ± 0.8	89 ± 8
14b	H_3_C–C=O	Ph	1.98 ± 0.07	78 ± 1.0	77 ± 4
14d	H_3_C–C=O	Bn	2.20 ± 0.01	83 ± 0.6	74 ± 4
18b	H	Ph	2.58 ± 0.03	92 ± 0.4	84 ± 1
18d	H	Bn	2.58 ± 0.01	94 ± 0.4	58 ± 2
Imipramine^c^					27 ± 3

a Interaction with human serum albumin was recorded by HPAC analysis.

b Amount (in %) of parent compound after incubation with mouse liver microsomes and NADPH for 90 min.

c Imipramine was used as reference compound to prove the activity of the microsomes and NADPH in the individual metabolic experiments.

The log *D*_7.4_ values of selected ligands were recorded by the recently reported micro-shake flask method.^40,41^ In these experiments, LC-MS was used to quantify the compounds in the aqueous layer after distribution between a MOPS buffer pH 7.4 and a *n*-octanol layer. The recorded log *D*_7.4_ values are in a promising range. The primary amine 12a and the corresponding acetamide 14a containing an unsubstituted piperidino moiety show almost the same log *D*_7.4_ values of −0.18 and −0.10. Introduction of a phenyl or benzyl moiety at 4-position of the piperidine ring increased the log *D*_7.4_ value by 1.2–2.2 log units. However, the log *D*_7.4_ values of phenyl and benzyl substituted piperidines do not differ considerably (compare 14b/14d, 18b/18d). The type of acyl moiety has little impact on the log *D*_7.4_ value, as the formamide 13b and the acetamide 14b have almost the same log *D*_7.4_ values. Removal of the benzylic OH moiety as in 18 resulted in increased log *D*_7.4_ values (Table 2).

High performance affinity chromatography (HPAC) at a stationary phase coated with human serum albumin was used to determine the plasma protein binding of the ligands or more precisely the affinity towards human serum albumin.^40,42,43^ This simplification is allowed, as human serum albumin represents the most abundant protein in human blood and is predominantly responsible for the transport of xenobiotics. The data show a clear correlation between the compound's lipophilicity and the human serum albumin binding. Very polar γ-amino alcohols, such as 12a and 14a reveal very low binding at human serum albumin of 36% and 42%, respectively. Lipophilic ligands 14d, 18b and 18d exhibit considerably higher human serum albumin binding of >80%, whereas intermediate human serum albumin binding was observed for γ-amino alcohols with intermediate lipophilicity (Table 2).

Since several clinical candidates fail due to low metabolic stability, the transformation of the ligands in the presence of mouse liver microsomes and NADPH was recorded already at a very early stage of development. The amount of intact compound after an incubation period of 90 min at 37 °C was recorded by LC-MS.^40,44^ The functionality of the assay was controlled with the reference compound imipramine, which was metabolically transformed considerably under the assay conditions. High metabolic stability, *i.e.*, more than 80% intact parent compound after 90 min, was observed for primary amines 12a (100%), 12b (87%), and 18b (84%) as well as acetamide 14a (89%). In contrast, the benzylamine 11a was intensively metabolized with mouse liver microsomes and NADPH. Only 14% of the parent compound 11a could be detected after an incubation period of 90 min. All other compounds showed intermediate metabolic stability of 58–77% parent compound after 90 min (Table 2).

### 2.4. Molecular docking

**Fig. 2:**
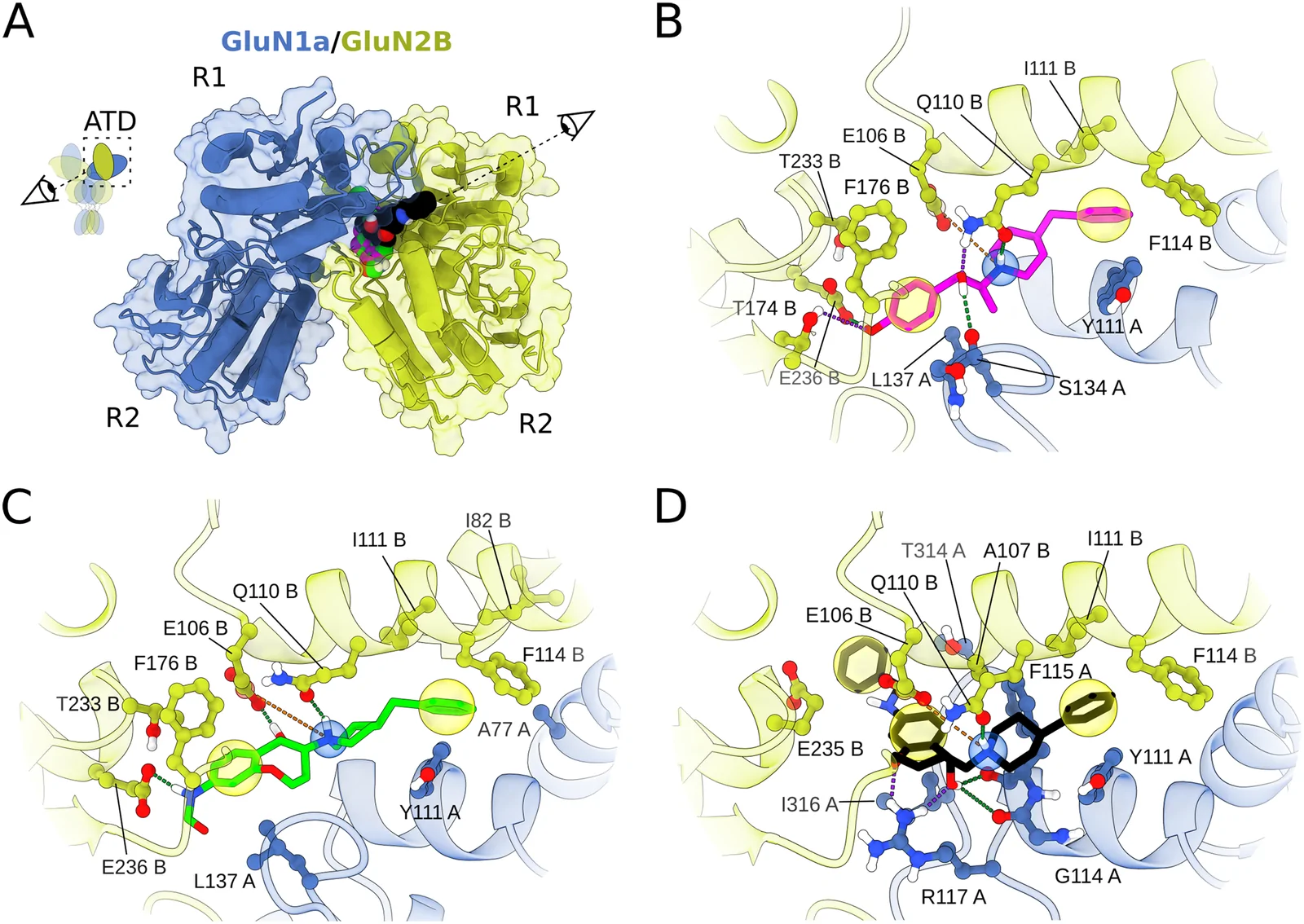
Binding mode investigations at the amino-terminal domain of the human GluN1a/GluN2B NMDA receptor (A). Proposed binding modes of ifenprodil (B), and 1-benzoxepine-based derivatives 13d (C) and 11b (D) obtained by molecular docking. Ligand–receptor interactions are illustrated by yellow spheres (hydrophobic contacts), red (hydrogen bond acceptors) and green pseudobonds (hydrogen bond donors).

To explore the binding mode and molecular interactions of the γ-amino alcohols, molecular docking was performed for compounds 11b and 13d, which exhibited the highest binding affinity. Since these 1-benzoxepine-based ligands displaced [^3^H]ifenprodil from its binding site, they were hypothesized to bind to the same allosteric pocket within the amino-terminal domain (ATD) of the human GluN1a/GluN2B NMDA receptor (Fig. 2A). The reliability of the docking protocol was validated by redocking ifenprodil into the homology model of the human ATD, which reproduced the binding pose of the reference structure (RMSD = 0.7 Å) (Fig. S1).

The proposed binding modes of γ-amino alcohols 11b and 13d suggest that the piperidine moiety adopts a binding orientation similar to that of ifenprodil, reproducing key interactions within the allosteric binding pocket. Most importantly, a hydrogen bond was predicted between the protonated piperidine N-atom and Gln110 of the GluN2B R1 domain, an interaction that has been reported to play a key role in binding ifenprodil and its derivatives.^45,46^ In addition, hydrophobic contacts between the aromatic ring of the benzyl or phenyl substituent and the hydrophobic pocket formed by GluN1a Tyr109 and GluN2B Phe114 were observed, consistent with previous reports identifying this region as a key determinant of ifenprodil sensitivity and WMS-14103 (3) binding.^24,45^ Furthermore, compound 13d established additional hydrophobic contacts within this subpocket, involving Ile82 (GluN1a) and Ala77 (GluN2B), resulting in a more extensive occupation of the hydrophobic cavity compared with compound 11b.

However, differences were observed in the orientation of the benzoxepine scaffold. Compound 13d closely reproduced the binding mode of ifenprodil and WMS-1410 (Fig. 2C and S1), with its aromatic moiety engaging in a T-shaped π–π interaction with GluN2B Phe176 and forming a hydrogen-bond with GluN2B Glu236 in the phenol-binding subpocket. This interaction involves the deprotonated carboxylate group of Glu236 and the amide NH of the formamido moiety. Both interactions have been reported to be relevant for the inhibitory potency of ifenprodil and WMS-1410 (3).^24,47^ In addition, docking results suggest that the benzylic hydroxyl group at the 5-position can form a hydrogen-bond with the GluN2B R1 domain *via* the side chain of Glu106. In contrast, the 4-phenylpiperidine 11b was predicted to adopt the benzylamino moiety in an alternative orientation, likely due to steric hindrance, preventing occupation of the phenol-binding subpocket (Fig. 2D). This new orientation present new interactions of the aromatic motif of 1-benzoxepine ring with Phe115 and Ile316 of GluN1a R1 domain and Ala107 of GluN2 R1 domain. Phe115 is positioned in proximity to the aromatic motif of the 1-benzoxepine ring, suggesting a possible T-shaped π–π interaction, although the geometry is not optimal. The biding pose shows an additional hydrogen-bond between the Arg117 and the O-atom of the carbonyl group of the backbone of Phe115 and the main chain of Gly114 of the GluN1a R1 domain with the benzylic OH group at the 5-position. These observations suggest that the differences in binding interactions and affinity between the 11 and 17 series, is because the benzylic OH group may enable additional hydrogen-bonding contacts that stabilize the non-canonical binding mode. Previous SAR studies have shown that the absence of the ring moiety attached to the piperidine group drastically reduces the binding affinity of ifenprodil analogs.^48^ However, compound 11a, was still capable of displacing ifenprodil. Docking results consistently indicate two alternative inverted binding modes for this ligand (Fig. S1), in both of which the benzylamino moiety occupies the hydrophobic pocket, while the ionizable piperidine moiety interacts with Glu106 of the GluN2B R2 domain through favorable electrostatic interactions. Meanwhile, the benzylamine interacts with the hydrophobic pocket residues like ifenprodil and the γ-amino alcohols 11b and 13d.

## 3. Conclusion

In this project, two key modifications were performed on the scaffold of potent NAM at NMDA receptors with GluN2B subunit: the flexible side chain containing the amino alcohol pharmacophore was conformationally restricted by embedding it into a seven-membered oxepine ring and the phenolic OH moiety was replaced by various secondary amines and amides.

For ligands with a 4-benzylpiperidinomethyl moiety in 4-position of the 1-benzoxepine scaffold, the GluN2B affinity increased from the benzylamine 11d, over the primary amine 12d up to the formamide 13d. Larger acyl moieties (*e.g.*, acetyl, isobutyryl, benzoyl) at the anilino group were not tolerated by the ifenprodil binding pocket of the NMDA receptor.

Unexpectedly, ligands without benzylic OH moiety, such as 18b and 18d revealed the same or even higher GluN2B affinity as the analogs 12b and 12d with benzylic OH moiety. Obviously, the benzylic OH moiety is not essential for binding at the ifenprodil binding site of GluN2B subunit-containing NMDA receptors. However, the benzylic OH moiety is able to increase the selectivity over the σ_1_ receptor. Whereas the γ-amino alcohol 12b has slightly higher affinity for the NMDA receptor, the allylamine 18d without benzylic OH moiety shows a fivefold preference for the σ_1_ receptor.

The most potent NMDA receptor antagonists of this compound series are the γ-amino alcohols 11b (*K*_i_ = 256 nM) and 13d (*K*_i_ = 278 nM) and the allylamines 17b (*K*_i_ = 478 nM) and 18b (*K*_i_ = 489 nM). However, the affinity at NMDA receptors with GluN2B subunit was generally rather low. It was concluded that the reduced affinity was due to the suboptimal H-bond donor (NH_2_, NHalkyl or NHacyl instead of OH), the unfavorable conformational restriction in the 1-benzoxepine ring, or the additional O-atom of the 1-benzoxepine ring increasing the electron density of the benzene ring.

## 4. Experimental

### 4.1. Chemistry, general

Unless otherwise noted, moisture sensitive reactions were conducted under dry N_2_. CH_2_Cl_2_ was distilled over CaH_2_. THF was distilled over sodium/benzophenone. Et_2_O and toluene were dried over molecular sieve 0.4 Å. Thin layer chromatography (tlc): silica gel 60 F254 plates (Merck). Flash chromatography (fc): silica gel 60, 40–64 μm (Merck); parentheses include: diameter of the column (*d*), length of the stationary phase, fraction size (*V*), eluent. Melting point: melting point apparatus Mettler Toledo MP50 Melting Point System, uncorrected. MS: microOTOF-Q II (Bruker Daltonics); APCI, atmospheric pressure chemical ionization. IR: FT-IR spectrophotometer MIRacle 10 (Shimadzu) equipped with ATR technique. Circular dichroism spectroscopy: JASCO J-600 spectropolarimeter (Jasco, Groß-Umstadt), 0.1 cm dell, solvent CH_3_CN. Nuclear magnetic resonance (NMR) spectra were recorded on Agilent 600-MR (600 MHz for ^1^H, 151 MHz for ^13^C) or Agilent 400-MR spectrometer (400 MHz for ^1^H, 101 MHz for ^13^C); *δ* in ppm related to tetramethylsilane and measured referring to CHCl_3_ (*δ* = 7.26 ppm (^1^H NMR) and *δ* = 77.2 ppm (^13^C NMR)), CHD_2_OD (*δ* = 3.31 ppm (^1^H NMR) and *δ* = 49.0 ppm (^13^C NMR)) and DMSO-*d*_6_ (*δ* = 2.54 ppm (^1^H NMR) and *δ* = 39.5 ppm (^13^C NMR)); coupling constants are given with 0.5 Hz resolution; the assignments of ^13^C and ^1^H NMR signals were supported by 2-D NMR techniques where necessary.

### 4.2. HPLC equipment and methods

HPLC method to determine the purity of compounds: pump: L-7100, degasser: L-7614, autosampler: L-7200, UV detector: L-7400, interface: D-7000, data transfer: D-line, data acquisition: HSM-Software (all from LaChrom, Merck Hitachi); equipment 2: pump: LPG-3400SD, degasser: DG-1210, autosampler: ACC-3000T, UV-detector: VWD-3400RS, interface: DIONEX UltiMate 3000, data acquisition: Chromeleon 7 (Thermo Fisher Scientific); column: LiChrospher® 60 RP-select B (5 μm), LiChroCART® 250-4 mm cartridge; flow rate: 1.0 mL min^−1^; injection volume: 5.0 μL; detection at *λ* = 210 nm; solvents: A: demineralized water with 0.05% (V/V) trifluoroacetic acid, B: acetonitrile with 0.05% (V/V) trifluoroacetic acid; gradient elution (% A): 0–4 min: 90%; 4–29 min: gradient from 90% to 0%; 29–31 min: 0%; 31–31.5 min: gradient from 0% to 90%; 31.5–40 min: 90%. The purity of all test compounds is greater than 95%.

### 4.3. General synthetic procedures

#### General procedure A – aminolysis of β-keto ester 8

A mixture of β-keto ester 8 (1 eq.), 4-(dimethylamino)pyridine (0.3 eq.), and respective secondary amine (2 eq.) was heated to 120 °C for the given time. After cooling down to rt, the reaction was terminated by addition of saturated aqueous NaHCO_3_ solution. The mixture was extracted with ethyl acetate. The combined organic layers were washed with brine, dried (Na_2_SO_4_), filtered and the solvent was removed under reduced pressure.

#### General procedure B – NaBH_4_ reduction of β-ketoamides 9

In a dried flask 1-benzoxepine-5-one 9 (1 eq.) was dissolved in methanol_abs_. NaBH_4_ (5 eq.) was slowly added to the clear vigorously stirred solution. After the addition was completed and NaBH_4_ was dissolved, the mixture was stirred at rt. Then, HCl (6.22 M, 10 eq.) was added, the mixture was stirred for 10 min and the pH adjusted to 7 with NaOH (2 M). For workup the aqueous layer was extracted with CH_2_Cl_2_. The combined organic layers were washed with brine, dried (Na_2_SO_4_), filtered and the solvent was removed under reduced pressure.

#### General procedure C – LiAlH_4_ reduction of β-hydroxy amides 10

In a dried Schlenk flask under N_2_ atmosphere carboxamide 10 (1 eq.) was dissolved in THF_abs._ and cooled to 0 °C. LiAlH_4_ (1 M in THF, 3 eq.) was slowly added with a syringe. The mixture was heated to reflux for the time given. For workup, the mixture was allowed to cool to rt. Then the mixture was slowly poured into a large amount of ice cooled water. The mixture was extracted with CH_2_Cl_2_. The combined organic layers were washed with brine, dried (Na_2_SO_4_), filtered and the solvent was removed under reduced pressure.

### 4.4. Synthetic procedures and analytical data

#### Methyl 4-amino-2-hydroxybenzoate (5)


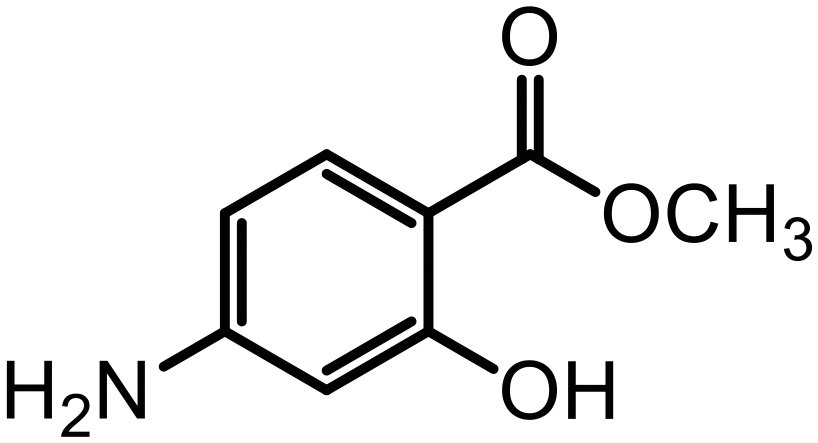
 4-Aminosalicylic acid (4, 2.00 g, 30.6 mmol) was suspended in methanol (40 mL). H_2_SO_4_,_conc._ (8.1 ml, 153 mmol) was carefully added until the resulting precipitate was dissolved. The reaction mixture was then heated to reflux for 12 h. For workup the reaction mixture was neutralized with aqueous solution of NaHCO_3_, transferred into a separation funnel, and extracted with dichloromethane (3 × 30 mL). The combined organic layers were washed with brine (40 mL) and dried (Na_2_SO_4_). The solvent was removed *in vacuo* and the residue was purified by fc (*ø* 8 cm, CH_2_Cl_2_ : CH_3_OH = 100 : 1, 15 cm, 100 mL, *R*_f_ = 0.50). Colorless solid, mp 120–121 °C, yield 3.55 g (76%). C_8_H_9_NO_3_, *M*_r_ = 167.1. MS (EM): *m*/*z* calcd. for C_8_H_10_NO_3_ 168.0661, found 168.0660. ^1^H NMR (DMSO-D_6_): *δ* [ppm] = 3.77 (s, 3H, CH_3_), 5.97 (d, *J* = 2.1 Hz, 1H, 3-H), 6.06–6.16 (m, 3H, NH_2_, 5-H), 7.42 (d, *J* = 8.7 Hz, 1H, 6-H), 10.75 (s, 1H, OH). ^13^C NMR (DMSO-D_6_): *δ* [ppm] = 52.3 (1C, OCH_3_), 100.4 (1C, C-3), 101.5 (1C, C-1), 108.0 (1C, C-5), 131.8 (1C, C-6), 155.0 (1C, C-4), 163.2 (1C, C-2), 170.4 (1C, C=O). IR (neat): *ν̃* [cm^−1^] = 3472 (N–H), 3378 (O–H), 1654 (C=O). Purity (HPLC): 99.4%, *t*_R_ = 14.67 min.

#### Methyl 4-benzamido-2-hydroxybenzoate (6)


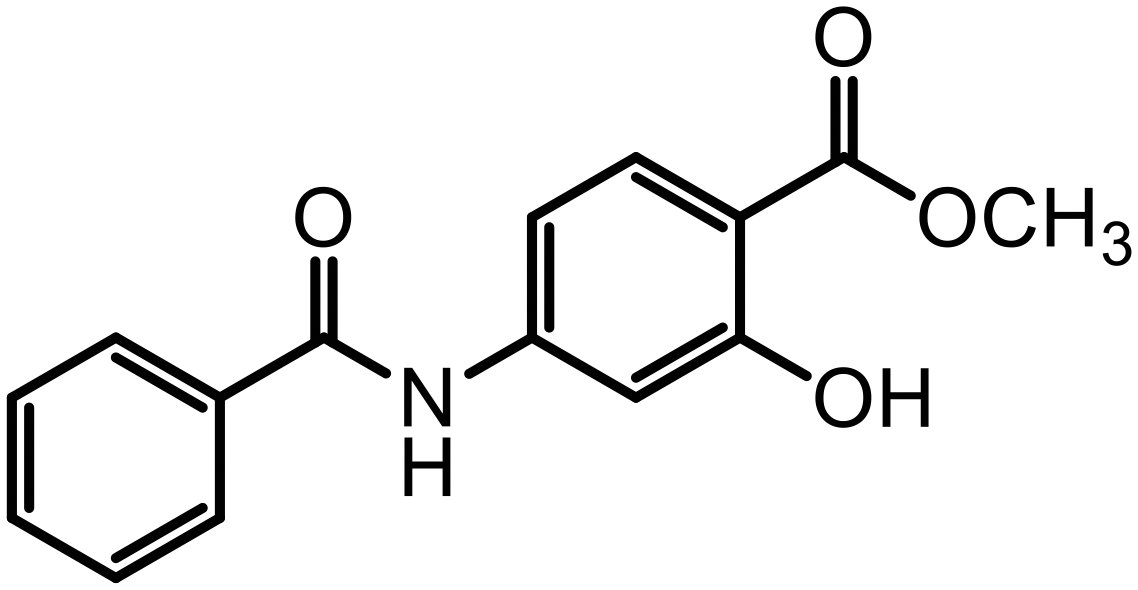
 Under N_2_, benzoyl chloride (7.7 g, 55.5 mmol) was dissolved in THF (30 mL) and cooled to 0 °C. Methyl ester 5 (6.6 g, 39.7 mmol) and triethylamine (4.0 g, 39.7 mmol), both dissolved in THF (20 mL), were slowly added. The reaction mixture was then warmed to rt and stirred for 2 h. The formed solid was separated, washed with THF and dissolved in CH_2_Cl_2_. The residual amounts of product in the THF layer were extracted with CH_2_Cl_2_ (4 × 30 mL) after the mixture was neutralized with aqueous NaHCO_3_ (30 mL). The combined organic layers were washed with brine (40 mL), and dried (NaSO_4_). The solvent was removed *in vacuo* and the residue was purified by fc (*ø* 8 cm, dichloromethane : methanol = 100 : 1, 15 cm, 100 mL, *R*_f_ = 0.75 (cyclohexane : ethyl acetate = 1 : 1)). Colorless solid, mp 168–170 °C, yield 7.94 g (74%). C_15_H_13_NO_4_, *M*_r_ = 271.3. MS (EM): *m*/*z* calcd. for C_15_H_14_NO_4_ 272.0923, found 272.0922. ^1^H NMR (DMSO-D_6_): *δ* [ppm] = 3.87 (s, 3H, O–CH_3_), 7.35 (dd, *J* = 8.8/2.1 Hz, 1H, 5-H), 7.47–7.66 (m, 4H, Ar–H), 7.76 (d, *J* = 8.8 Hz, 1H, 6-H), 7.88–8.00 (m, 2H, Ar–H), 10.49 (s, 1H, NH), 10.62 (s, 1H, O–H). ^13^C NMR (CDCl_3_): *δ* [ppm] = 52.4 (1C, CH_3_), 107.5 (1C, C-3), 108.7 (1C, C-1), 111.1 (1C, C-5), 127.3 (2C, C-2_Bz_ and C-6_Bz_), 129.1 (2C, C-3_Bz_ and C-5_Bz_), 131.1 (1C, C-4_Bz_), 134.7 (1C, C-6), 144.6 (1C, C-4), 162.9 (1C, C-2), 166.0 (1C, NHC=O), 170.3 (1C, C=0). IR (neat): *ν̃* [cm^−1^] = 3390 (N–H), 3100 (O–H), 2965 (OCH_3_), 1742 (C=O), 1671 (O=CNHR). Purity (HPLC): 97.8%, *t*_R_ = 19.49 min.

#### Methyl 4-benzamido-2-[3-(methoxycarbonyl)propoxy]benzoate (7)


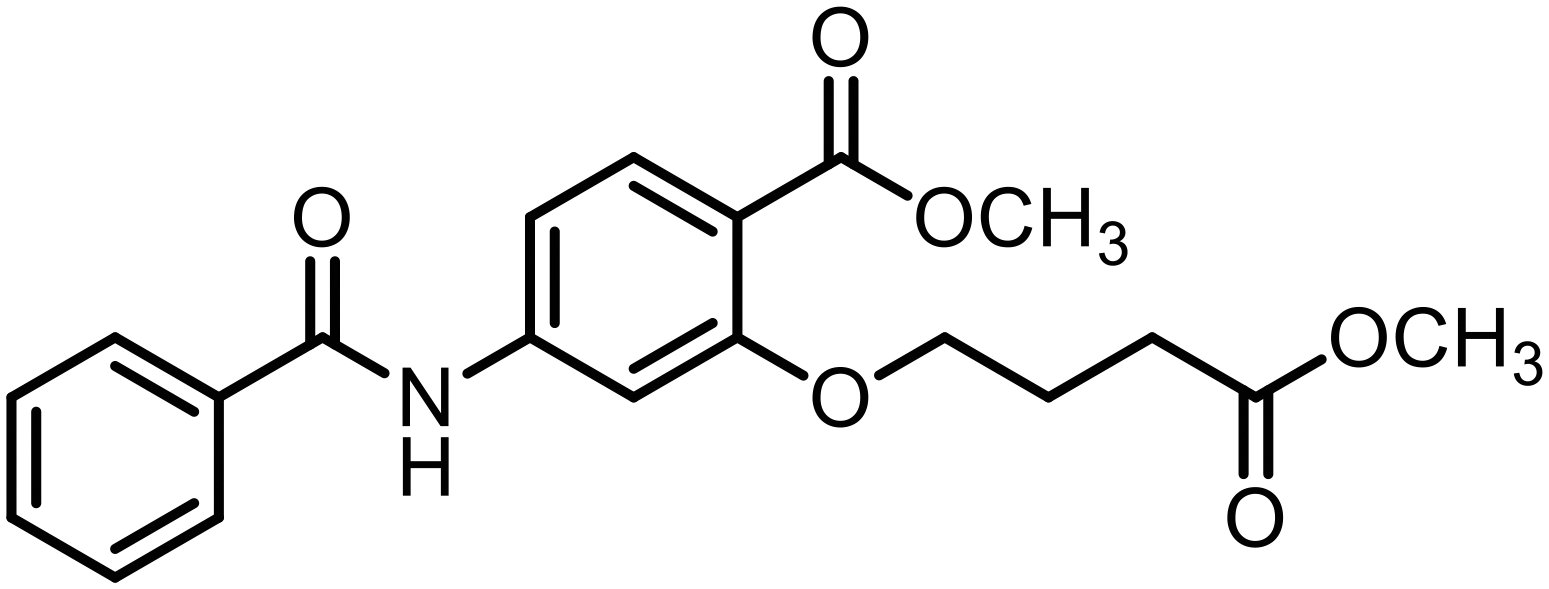
 Benzamide 6 (7.9 g, 30 mmol) was dissolved in DMF (100 mL). K_2_CO_3_ (12.2 g, 88 mmol) was added and the mixture was stirred for 15 min at rt. Methyl 4-bromobutyrate (6.1 g, 33 mmol) was then added and the suspension was stirred vigorously overnight at 80 °C. After cooling to rt, water was poured into the mixture and the mixture was stirred until all of the K_2_CO_3_ was dissolved. The resulting clear solution was extracted with ethyl acetate several times. The combined organic layers were washed with brine, dried (Na_2_SO_4_) and the solvent was removed *in vacuo*. The residue was purified by fc (*ø* 8 cm, cyclohexane : ethyl acetate = 5 : 1, 15 cm, 100 mL, *R*_f_ = 0.30 (cyclohexane : ethyl acetate 2.5 : 1)). Colorless solid, mp 106–108 °C, yield 9.4 g (84%). C_20_H_21_NO_6_, *M*_r_ = 371.4. MS (ESI): *m*/*z* [%] = 370 (M–H, 100). ^1^H NMR (CDCl_3_): *δ* [ppm] = 2.10–2.23 (m, 2H, CH_2_*CH*_*2*_CH_2_), 2.61 (t, 7.4 Hz, CH_2_CH_2_*CH*_*2*_), 3.67 (s, 3H, OCH_3_), 3.86 (s, 3H, OCH_3_), 4.11 (t, 2H, *CH*_*2*_CH_2_CH_2_), 7.0 (dd, *J* = 7.1/4.9 Hz, 1H, 5-H_arom._), 7.45–7.52 (m, 2H, H_Bz_), 7.54–7.59 (m, 1H, H_arom._), 7.72 (d, *J* = 2 Hz, 1H, H_Bz_), 7.81–7.91 (m, 3H, H_Bz_), 7.85–7.89 (m, 1H, H_arom._), 8.10 (s, 1H, NH). ^13^C-NMR (CDCl_3_): *δ* [ppm] = 24.7 (1C, CH_2_*C*H_2_CH_2_), 30.6 (1C, CH_2_CH_2_*C*H_2_), 52.0 (2C, 2 × COO*C*H_3_), 67.8 (1C, *C*H_2_CH_2_CH_2_), 104.6 (1C, C-3), 111.1 (1C, C-5), 115.7 (1C, C-1), 127.3 (2C, C-2_Bz_ and C-6_Bz_), 129.1 (2C, C-3_Bz_ and C-5_Bz_), 132.5 (1C, C-4_Bz_), 133.2 (1C, C-6), 134.7 (C-1-C_6_H_5_), 143.2 (1C C-4), 160.0 (1C, C-2), 166.1 (1C, NHC=O or Ar–*C*=O), 166.3 (1C, NHC=O or Ar–*C*=O), 174.0 (1C, CH2*C*O_2_CH3). IR (neat): *ν̃* [cm^−1^] = 3328 (N–H), 2952 (OCH_3_), 2359, 1697 (O=CNHR). Purity (HPLC): *t*_R_ = 19.34 min, purity 94.4%.

#### Methyl 8-benzamido-5-oxo-2,3,4,5-tetrahydro-1-benzoxepine-4-caboxylate (8)


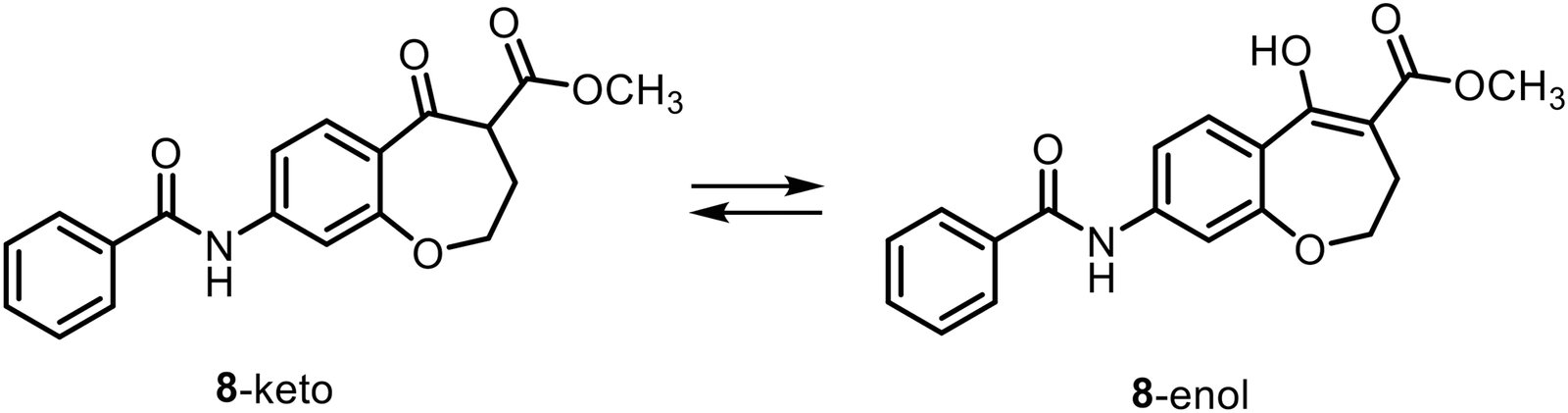
 Under N_2_ atmosphere, diester 7 (3.48 g, 9.37 mmol) was dissolved in THF_abs._ (20 mL) in a 100 mL Schlenk flask. The solution was cooled to 0 °C. With a syringe pump LiHMDS (1 M in THF, 28 mL, 28 mmol) was added slowly (10 mL h^−1^). After the addition was completed, the mixture was warmed to rt and stirred overnight. The reaction was terminated by addition of saturated NaHCO_3_ solution. HCl 20% was added and the pH was adjusted to 7. The mixture was extracted with CH_2_Cl_2_ (8×). The combined organic layers were washed with brine, dried (Na_2_SO_4_) and the solvent was removed *in vacuo*. The resulting residue was purified by fc (*ø* 8 cm, cyclohexane : ethyl acetate = 5 : 1, 15 cm, 100 mL, *R*_f_ = 0.30 (cyclohexane : ethyl acetate 3 : 2)). Colorless solid, mp 146–147 °C, yield 1.63 g (51%). C_19_H_17_NO_5_, *M*_r_ = 339.3. MS (EM): *m*/*z* calcd. for C_19_H_18_NO_5_ 340.1185, found 340.1187. MS (ESI): *m*/*z* (%) = 338 (M–H, 100). ^1^H NMR (CDCl_3_): *δ* [ppm] = 2.33–2.55 (m, 2 × 0.67H, 3-H), 2.65 (t, *J* = 5.3 Hz, 2 × 0.33H, 3-H·), 3.68 (s, 3 × 0.67H, OCH_3_), 3.76 (s, 3 × 0.33H, OCH_3_·), 3.95–4.07 (m, 2 × 0.67H, 2-H and 4-H), 4.29 (t, *J* = 5.3 Hz, 2 × 0.33H, 2-H·), 4.36–4.44 (m, 2 × 0.67H, 2-H), 7.18 (dd, *J* = 8.3/2.3, 0.67H, 7-H), 7.23 (dd, *J* = 8.8/2.2 Hz, 0.33H, 7-H·), 7.36–7.44 (m, 4 × 0.67H, 9-H, 3-H_Bz_, 4-H_Bz_, 5-H_Bz_), 7.45–7.53 (m, 3 × 0.33H, 3-H_Bz_, 4-H_Bz_, 5-H_Bz_), 7.57 (d, *J* = 1.9 Hz, 0.33H, 9-H·), 7.73 (d, *J* = 8.6 Hz, 0.67H, 6-H), 7.76–7.81 (m, 2H, 2-H_Bz_ and 6-H_Bz_ and 2-H_Bz_· and 6 H_Bz_·), 7.90 (d, *J* = 8.8 Hz, 0.33 H, 6-H·), 7.92 (s, 0.33 H, NH·), 8.03 (s, 0.67H, NH), 13.13 (s, 0.33H, OH·). According to the ^1^H NMR spectrum, the ratio of 8-keto : 8-enol tautomers is 67 : 33. ^13^C NMR (CDCl_3_): *δ* [ppm] = 27.3 (1C, C-3·), 29.5 (1C, C-3), 52.3 (3C, OCH_3_·), 52.7 (3C, OCH_3_), 56.3 (1C, C-4), 72.3 (1C, C-2), 74.5 (1C, C-2·), 100.5 (1C, C-4·), 111.4 (1C, C-9), 112.2 (1C, C-9·), 114.1 (1C, C-7·), 114.5 (1C, C-7), 120.9 (1C, C-5a·), 123.8 (1C, C-5a), 127.3 (4C, C-2_Bz_ and C-6_Bz_ and C-2_Bz_· and C-6_Bz_·), 129.1 (4C, C-3_Bz_ and C-5_Bz_ and C-3_Bz_· and C-5_Bz_·), 130.6 (1C, C-6·), 131.3 (1C, C-6), 132.3 (1C, C-4_Bz_·), 132.5 (1C, C-4_Bz_), 134.5 (1C, C-1_Bz_), 134.8 (1C, C-1_Bz_·), 141.4 (1C, C-8·), 143.7 (1C, C-8), 159.1 (1C C-9a·), 163.5 (1C, C-9a), 166.0 (2C, NHC=O and NHC=O·), 166.7 (1C, C5), 170.2 (1C, H_3_C*C*=O), 173.4 (1C, H_3_C*C*=O·), 194.5 (1C, C-5). IR (neat): *ν̃* [cm^−1^] = 2956 (C–H), 1763 (C=O_ester_), 1656 (C=O_amide_). Purity (HPLC): 85.8%, *t*_R_ = 18.13 min.

#### *N*-{5-Oxo-4-[(piperidin-1yl)carbonyl]-2,3,4,5-tetrahydro-1-benzoxepin-8-yl}benzamide (9a)


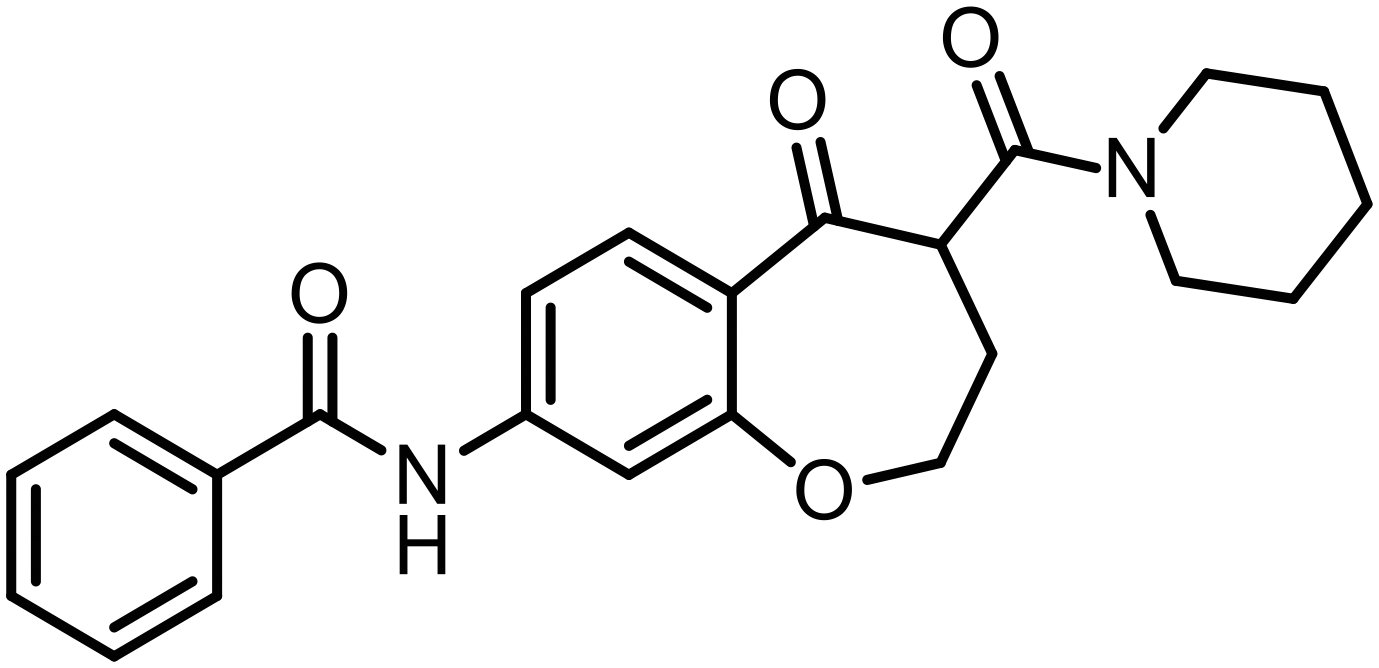
 According to *General procedure A*, a mixture of 8 (2.20 g, 6.48 mmol), piperidine (0.83 g, 9.72 mmol), 4-(dimethylamino)pyridine (0.24 g, 1.94 mmol), and toluene (30 mL) was heated to 130 °C overnight. After workup, the residue was purified by fc (*ø* 4 cm, *h* = 15 cm, cyclohexane : ethyl acetate 1 : 2, *R*_f_ = 0.29 (cyclohexane : ethyl acetate 1 : 1)). Pale yellow solid, mp 137–140 °C, yield 0.834 g (33%). C_23_H_24_N_2_O_4_, *M*_r_ = 392.4. MS (EM): *m*/*z* calcd. for C_23_H_25_N_2_O_4_ 393.1814, found 393.1813. ^1^H NMR (CDCl_3_): *δ* [ppm] = 1.50–1.57 (m, 6H, 3-H, 4-H, 5-H_piperidine_), 2.43 (ddt, *J* = 14.1/11.4/7.1 Hz, 1H, 3-H), 2.57 (dddd, *J* = 13.8/10.6/5.3/3.1 Hz, 1H, 3-H), 3.01–3.07 (m, 2H, 2-H_piperidine_ and 6-H_piperidine_), 3.45–3.54 (m, 1H, 2-H_piperidine_ or 6-H_piperidine_), 3.54–3.64 (m, 1H, 2-H_piperidine_ or 6 H_piperidine_), 3.93 (ddd, *J* = 12.4/11.4/5.3 Hz, 1H, 2-H), 4.12–4.19 (m, 1H, 4-H), 4.55 (ddd, *J* = 12.4/7.0/3.0 Hz, 1H, 2-H), 7.23 (dd, *J* = 8.6/2.1 Hz, 1H, 7-H), 7.40–7.53 (m, 3H, 3-H, 4-H, 5-H_Bz_), 7.54 (d, *J* = 2.0 Hz, 1H, 9-H), 7.67 (d, *J* = 8.5 Hz, 1H, 6-H), 7.78–7.85 (m, 2H 2-H, 6-H_Bz_), 8.10 (s, 1H, NH). ^13^C NMR (CDCl_3_): *δ* [ppm] = 24.7 (1C, C-4_piperidine_), 25.6, 26.1 (2C, C-3 and C-5_piperidine_), 30.9 (1C, C-3), 43.4, 47.0 (2C, C-2 and C-6_piperidine_), 53.6 (1C, C-4), 72.9 (1C, C-2), 111.3, 114.3 (2C, C-7, C-9), 124.0 (1C, C-5a), 127.4 (2C, C-2 and C-6_Bz_), 129.2 (2C, C-3_Bz_ and C-5_Bz_), 130.7 (1C, C-4_Bz_), 132.5 (1C, C-6), 134.6 (1C, C-1_Bz_), 143.5 (1C, C-8), 163.6 (1C, C-9a), 166.1 (1C, NHC=O), 167.0 (1C, 1C, COCH*C*=ONH), 195.8 (1C, C-5). IR (neat): *ν̃* [cm^−1^] = 3306 (NH), 3287 (NH), 2936 (C–H), 2854 (C–H), 1674 (C=O). Purity (HPLC): 93.4%, *t*_R_ = 19.18 min.

#### *N*-{5-Oxo-4-[(4-phenylpiperidin-1-yl)carbonyl]-2,3,4,5-tetrahydro-1-benzoxepin-8-yl}benzamide (9b)


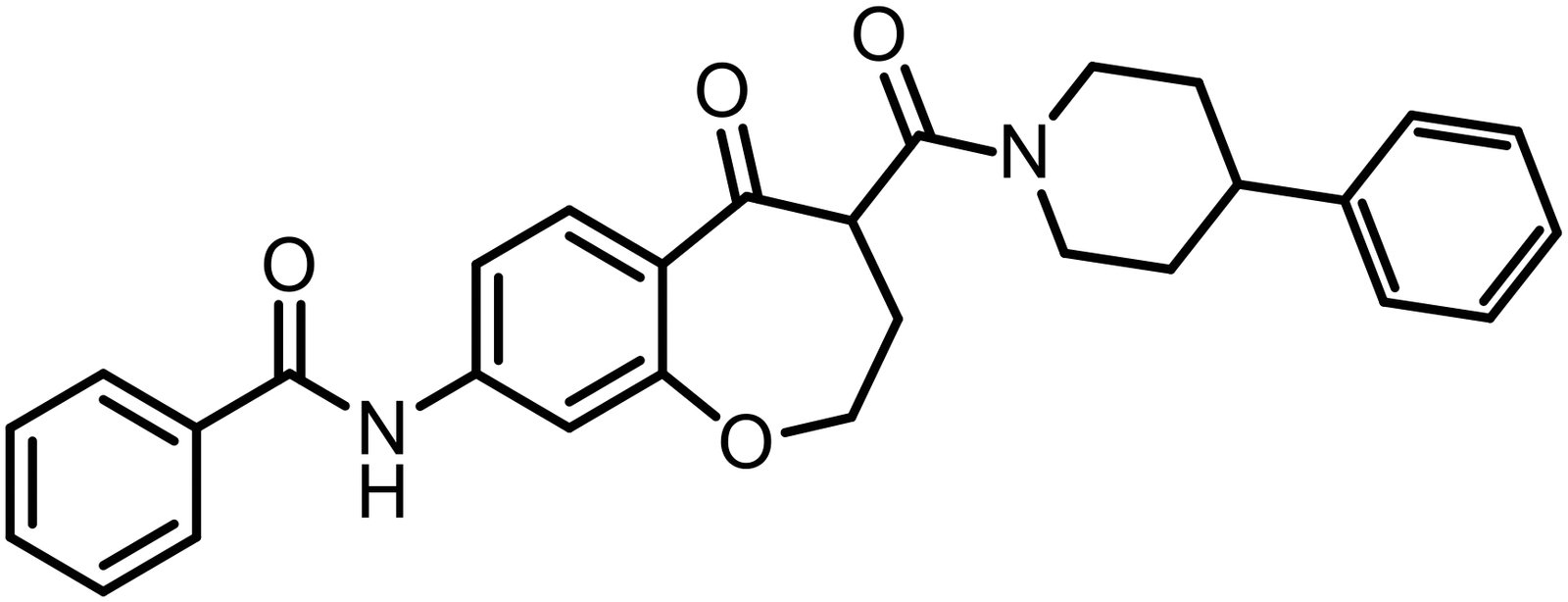
 According to *General procedure A*, a mixture of 8 (0.207 g, 0.61 mmol), 4-phenylpiperidine (0.150 g, 0.93 mmol), 4-(dimethylamino)pyridine (0.044 g, 0.36 mmol), and toluene (25 mL) was heated to reflux for 32 h. After workup, the residue was purified by fc (*ø* 3 cm, *h* = 15 cm, cyclohexane : ethyl acetate 1 : 3.5, *R*_f_ = 0.11 (cyclohexane : ethyl acetate 3 : 2)). Colorless solid, mp 206–207 °C, yield 253 mg (90%). C_29_H_28_N_2_O_4_, *M*_r_ = 468.5. MS (EM): *m*/*z* calcd. for C_29_H_28_N_2_O_4_ 469.2129, found 469.2099. ^1^H NMR (CDCl_3_): *δ* [ppm] = 1.38–1.82 (m, 4H, 3-H, 5-H_piperidine_), 2.29–2.55 (m, 2H, 3-H), 2.53–2.79 (m, 2H, 2-H, 4-H_piperidine_), 2.87 (t, *J* = 11.5 Hz, 0.5H, 6-H_piperidine_), 3.02 (t, *J* = 12.2 Hz, 0.5H, 6-H_piperidine_), 3.46 (d, *J* = 13.6 Hz, 0.5H, 6-H_piperidine_), 3.52 (d, *J* = 13.1 Hz, 0.5H, 6-H_piperidine_), 3.90–3.97 (m, 1H, 2-H), 4.38–4.53 (m, 1H, 4-H), 4.55–4.67 (m, 2H, 2-H_piperidine_ and 2-H), 7.09–7.36 (m, 5H, C_6_H_5_), 7.47–7.66 (m, 5H, 3-H, 4-H, 5-H_Bz_, 7-H, 9-H), 7.74 (dd, *J* = 5.2/1.9 Hz, 1H, 6-H), 7.96 (d, *J* = 8.0 Hz, 2H, 2-H, 6-H_Bz_), 10.54 (s (broad), 1H, NH). ^13^C NMR (CDCl_3_): *δ* [ppm] = 30.8 (1C, C-3), 33.3 (2C, C-3, C-5_piperidine_), 42.3 (1C, C-4_piperidine_), 42.7 (1C, C-2_piperidine_), 46.3 (1C, C-6_piperidine_), 53.6 (1C, C-4), 72.9 (1C, C-2), 111.2 (1C, C-7), 114.8 (1C, C-9), 123.5 (1C, C-5a), 126.8 (1C, C-4-C_6_H_5_), 127.3 (2C, C-2, C-6-C_6_H_5_), 128.5 (2C, C-2, C-6_Bz_), 129.1 (4C, C-3, C-5_Bz_ and C-3, C-5-C_6_H_5_), 130.4 (1C, C-4_Bz_), 132.6 (1C, C-6), 135.2 (1C, C-1_Bz_), 145.1 (1C, C-8), 146.2 (1C, C-1_Bz_), 163.0 (1C, C-9a), 166.8 (1C, NH–C=O), 171.0 (1C, R_2_N–C=O), 196.7 (1C, C-5). IR (neat): *ν̃* [cm^−1^] = 3283 (N–H), 2938 (C–H), 1684 (C=O). Purity (HPLC): 99.5%, *t*_R_ = 21.19 min.

#### *N*-{5-Oxo-4-[(4-phenylpiperazin-1-yl)carbonyl]-2,3,4,5-tetrahydro-1-benzoxepin-8-yl}benzamide (9c)


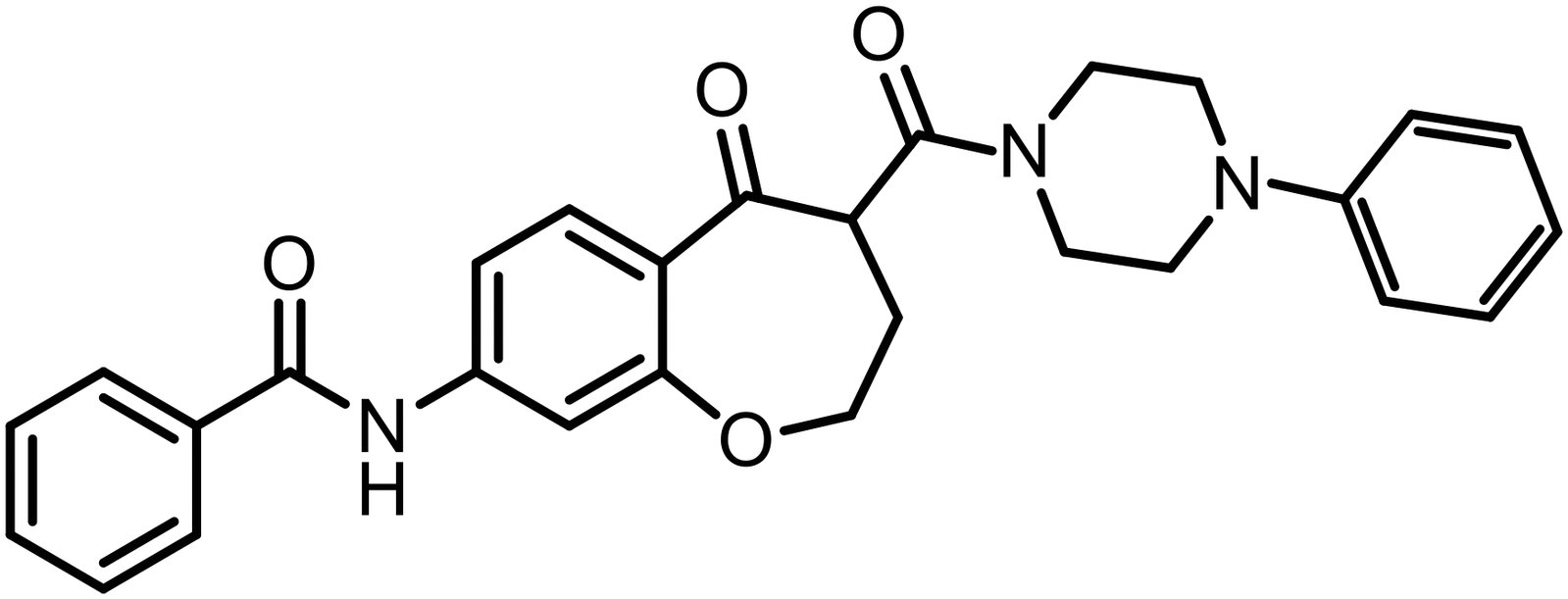
 According to *General procedure A*, a mixture of 8 (3.00 g, 8.85 mmol), 1-phenylpiperazine (2.87 g, 17.7 mmol), 4-(dimethylamino)pyridine (0.324 g, 2.66 mmol), and toluene (50 mL) was heated to reflux for 24 h. After workup, the residue was purified by fc (*ø* 4 cm, *h* = 15 cm, fraction size 65 mL, cyclohexane : ethyl acetate 2 : 1 → 1.5 : 1 → 1 : 1, *R*_f_ = 0.30 (cyclohexane : ethyl acetate 1 : 1)). Yellow solid, mp 112–113 °C, yield 3.02 g (73%). C_28_H_27_N_3_O_4_, *M*_r_ = 469.5. MS (EM): *m*/*z* calcd. for C_28_H_28_N_2_O_4_ 470.2080, found 470.2043. ^1^H NMR (CDCl_3_): *δ* [ppm] = 2.38–2.55 (m, 1H, 3-H), 2.60 (dddd, *J* = 14.0/10.5/5.4/3.5 Hz, 1H, 3-H), 2.91–3.14 (m, 3H, CH_2 piperazine_), 3.71 (ddd, *J* = 13.1/6.9 /3.3 Hz, 1H, CH_2 piperazine_), 3.23–3.35 (m, 2H, CH_2 piperazine_), 3.71 (ddd, *J* = 13.1/6.9/3.3 Hz, 1H, CH_2 piperazine_), 3.85 (ddd, *J* = 12.9/6.9/3.4 Hz, 1H, CH_2 piperazine_), 3.96 (ddd, *J* = 12.3/11.0/5.4 Hz, 1H, 2-H), 4.22 (dd, *J* = 10.5/7.1 Hz, 1H, 4-H), 4.56 (ddd, *J* = 12.3/6.9/3.4 Hz, 1H, 2-H), 6.80–6.87 (m, 3H, 2-H, 4-H and 6-H_N–Ph_), 7.16–7.22 (m, 2H, 2-H- and 6-H_N–Ph_), 7.23 (dd, *J* = 6.4/2.1 Hz, 1H, 7-H), 7.40–7.49 (m, 2H, 3-H and 5-H_Bz_), 7.49–7.56 (m, 1H, 4-H_Bz_), 7.57 (d, *J* = 2.1 Hz, 1H, 9-H), 7.70 (d, *J* = 8.5 Hz, 1H, 6-H), 7.86–7.79 (m, 2H, 2-H and 6-H_Bz_), 8.03 (s, 1H, NH). ^13^C NMR (CDCl_3_): *δ* [ppm] = 30.5 (1C, C-3), 42.3, 45.9, 49.5 (4C, C-2, C-3, C-5, C-6_piperazine_), 53.7 (1C, C-4), 72.8 (1C, C-2), 111.5 (1C, C-9), 114.6 (1C, C-7), 115.9 (2C, C-2, C-6_N–Ph_), 120.7 (1C, C-4_N–Ph_), 123.8 (1C, C-5a), 127.5, 129.1, 129.5 (6C, C-2, C-3, C-5, C-6_Bz_ and C-3, C-5_N–Ph_), 136.8 (1C, C-4_Bz_), 132.6 (C-6), 134.5 (1C, C-1_Bz_), 143.9 (1C, C-8), 151.1 (1C, C-1_N–Ph_), 163.5 (1C, C-9a), 166.3, 167.5 (2C, O=C–NR_2_ and NHC=O), 195.6 (1C, C-5). IR (neat): *ν̃* [cm^−1^] = 3707 (NH), 2970 (C–H), 2866 (C–H), 1635 (C=O). Purity (HPLC): 98.5%, *t*_R_ = 18.8 min.

#### *N*-{5-Oxo-4-[(4-benzylpiperidin-1-yl)carbonyl]-2,3,4,5-tetrahydro-1-benzoxepin-8-yl}benzamide (9d)


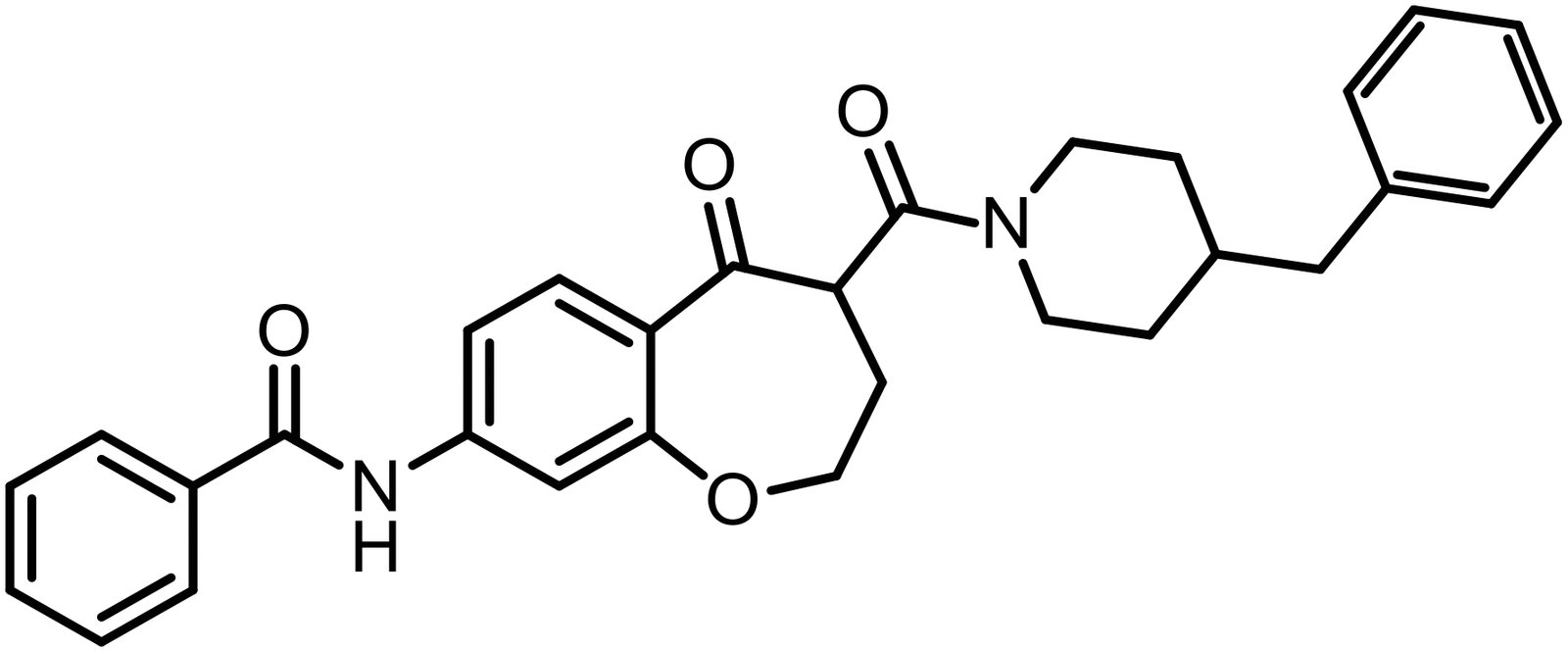
 According to *General procedure A*, a mixture of 8 (1.754 g, 5.17 mmol), 4-benzylpiperidine (1.36 g, 7.75 mmol), 4-(dimethylamino)pyridine (0.190 g, 1.6 mmol), and toluene (50 mL) was heated to reflux for 24 h. After workup, the residue was purified by fc (*ø* 4 cm, *h* = 15 cm, cyclohexane : ethyl acetate 1.5 : 1, *R*_f_ = 0.30 (cyclohexane : ethyl acetate 1 : 1)). Pale yellow solid, mp 110 °C (decomposition), yield 1.95 g (79%). C_30_H_30_N_2_O_4_, *M*_r_ = 482.6. MS (EM): *m*/*z* calcd. for C_30_H_31_N_2_O_4_ 483.2284, found 483.2248. ^1^H NMR (CDCl_3_): *δ* [ppm] = 0.85–1.28 (m, 3H, 3-H and 5-H_piperidine_), 1.42–1.72 (m, 2H, 3-H or 5-H_piperidine_ and 2-H or 6-H_piperidine_), 2.38–2.53 (m, 3H, 4-H_piperidine_ and 3-H), 2.53–2.64 (m, 2H, NC*H*_2_C_6_H_5_), 2.49–2.67 (m, 1H, 6-H_piperidine_), 3.29 (t, *J* = 13.0 Hz, 1H, 6-H_piperidine_), 3.85–4.02 (m, 1H, 2-H), 4.16 (ddd, *J* = 9.3/7.9/1.7 Hz, 1H, 4-H), 4.45–4.67 (m, 2H, 2-H_piperidine_ and 2-H), 7.00–7.16 (m, 3H, 2-H, 4-H, 6-H–C_6_H_5_), 7.16–7.26 (m, 3H, 3-H, 5-H–C_6_H_5_ and 9-H), 7.40–7.49 (m, 2H, 3-H, 5-H_Bz_), 7.50–7.56 (m, 2H, 4-H_Bz_ and 7-H), 7.66 (d, *J* = 8.5 Hz, 0.5H, 6-H), 7.69 (d, *J* = 8.5 Hz, 0.5H, 6-H), 7.77–7.84 (m, 2H, 2-H, 6-H_Bz_), 7.87 (s, 1H, NH). ^13^C NMR (CDCl_3_): *δ* [ppm] = 30.8 (1C, C-3), 31.8 (2C, C-3 and C-5_piperidine_), 38.5 (1C, C-4_piperidine_), 42.8 (1C, *C*H_2_C_6_H_5_), 46.3 (2C, C-2 and C-6_piperidine_), 53.7 (1C, C-4), 72.9 (1C, C-2), 111.3 (1C, C-9), 114.3 (1C, C-7), 124.0 (1C, C-5a), 126.2 (1C, C-4-C_6_H_5_), 127.4, 128.5, 129.1, 129.3 (8C, C-2, C-3, C-5, C-6_Bz_ and C-2, C-3, C-5, C-6-C_6_H_5_), 130.7 (1C, C-4_Bz_), 132.6 (1C, C-6), 134.6, 140.2 (2C, C-1_Bz_ and C-1-C_6_H_5_), 143.5 (1C, C-8), 163.5 (1C, C-9a), 166.1 (1C, NHC=O), 167.0 (R_2_N–C=O), 195.9 (1C, C-5). IR (neat): *ν̃* [cm^−1^] = 3676 (NH), 2970 (C–H), 2920 (C–H), 1674 (C=O_ketone_), 1631 (C=O_amide_). Purity (HPLC): 98.7%, *t*_R_ = 21.51 min.

#### 8-Benzamido-*N*,*N*-diethyl-5-oxo-2,3,4,5-tetrahydro-1-benzoxepine-4-caboxamide (9e)


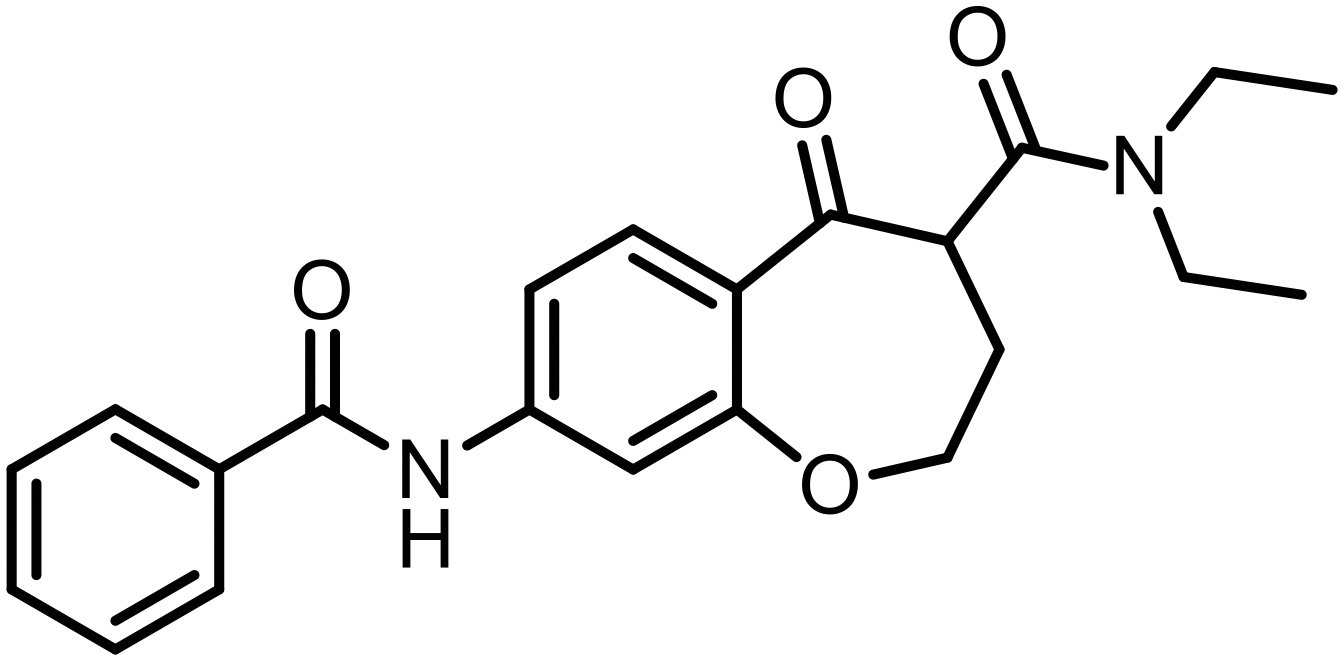
 According to *General procedure A*, a mixture of 8 (0.512 g, 1.5 mmol), 4-(dimethylamino)pyridine (0.073 g, 0.6 mmol), diethylamine (0.219 g, 3 mmol) and toluene (25 mL) was heated to reflux for 16 h. After workup, the residue was purified by fc (*ø* 3 cm, *h* = 15 cm, cyclohexane : ethyl acetate 3.5 : 1 → 2 : 1, *R*_f_ = 0.25). Colorless solid, mp 84–86 °C, yield 0.439 g (77%). C_22_H_24_N_2_O_4_, *M*_r_ = 380.4. MS (EI): *m*/*z* (%) 380 (M, 4), 281 (M–O=CN(CH_3_CH_2_)_2_, 12). ^1^H NMR (CDCl_3_): *δ* [ppm] = 1.02 (t, *J* = 7.1 Hz, 3H, N(CH_2_C*H*_3_)_2_), 1.11 (t, *J* = 7.1 Hz, 3H, N(CH_2_C*H*_3_)_2_), 2.32–2.49 (m, 1H, 3-H), 2.53–2.70 (m, 1H, 3-H), 3.03 (q, *J* = 7.2 Hz, 2H, N(C*H*_2_CH_3_)_2_), 3.25 (dq, *J* = 14.1/7.1 Hz, 1H, 1 × N(C*H*_2_CH_3_)_2_), 3.49 (dq, *J* = 14.1/7.1 Hz, 1H, 1 × N(C*H*_2_CH_3_)_2_), 3.96 (ddd, *J* = 12.3/11.0/5.2 Hz, 1H, 2-H), 4.14 (dd, *J* = 10.5/7.0 Hz, 1H, 4-H), 4.54 (ddd, *J* = 12.3/6.8/3.5 Hz, 1H, 2-H), 7.21 (dd, *J* = 8.9/2.4 Hz, 1H, 7-H), 7.39–7.53 (m, 3H, 3-H_Bz_, 4-H_Bz_, 5-H_Bz_) 7.56 (d, *J* = 2.0 Hz, 1H, 9-H), 7.70 (dd, *J* = 8.5 Hz, 1H, 6-H), 7.78–7.86 (m, 2H, 2-H_Bz_ and 6-H_Bz_), 8.01 (s, 1H, NH). ^13^C NMR (CDCl_3_): *δ* [ppm] = 13.0 (1C, CH_2_*C*H_3_), 14.5 (1C, CH_2_*C*H_3_), 30.8 (1C, C-3), 40.6 (1C, *C*H_2_CH_3_), 42.2 (1C, *C*H_2_CH_3_), 53.6 (1C. C-4), 72.9 (1C, C-2), 111.4 (1C, C-9), 114.3 (1C, C-7), 124.3 (1C, C-5a), 127.3 (2C, C-2_Bz_ and C-6_Bz_), 130.9 (2C, C-3_Bz_ and C-5_Bz_), 132.6 (2C, C-4_Bz_ and C-6), 134.6 (1C, C-1_Bz_), 143.4 (1C, C-8), 163.3 (1C, C-9a), 166.0 (1C, NHC=O), 168.0 (1C, COCH*C*=ONEt_2_), 196.0 (1C, *C*=OCHCONEt_2_). IR (neat): *ν̃* [cm^−1^] = 3298 (NH broad), 2970 (C–H), 2935 (C–H), 1674 (C=O), 1627 (C=O_amide_). Purity (HPLC): 99.9%, *t*_R_ = 18.71 min.

#### *N*-{*cis*-5-Hydroxy-4-[(piperidin-1-yl)carbonyl]-2,3,4,5-tetrahydro-1-benzoxepin-8-yl}benzamide (*cis*-10a) and *N*-{*trans*-5-hydroxy-4-[(piperidin-1-yl)carbonyl]-2,3,4,5-tetrahydro-1-benzoxepin-8-yl}benzamide (*trans*-10a)


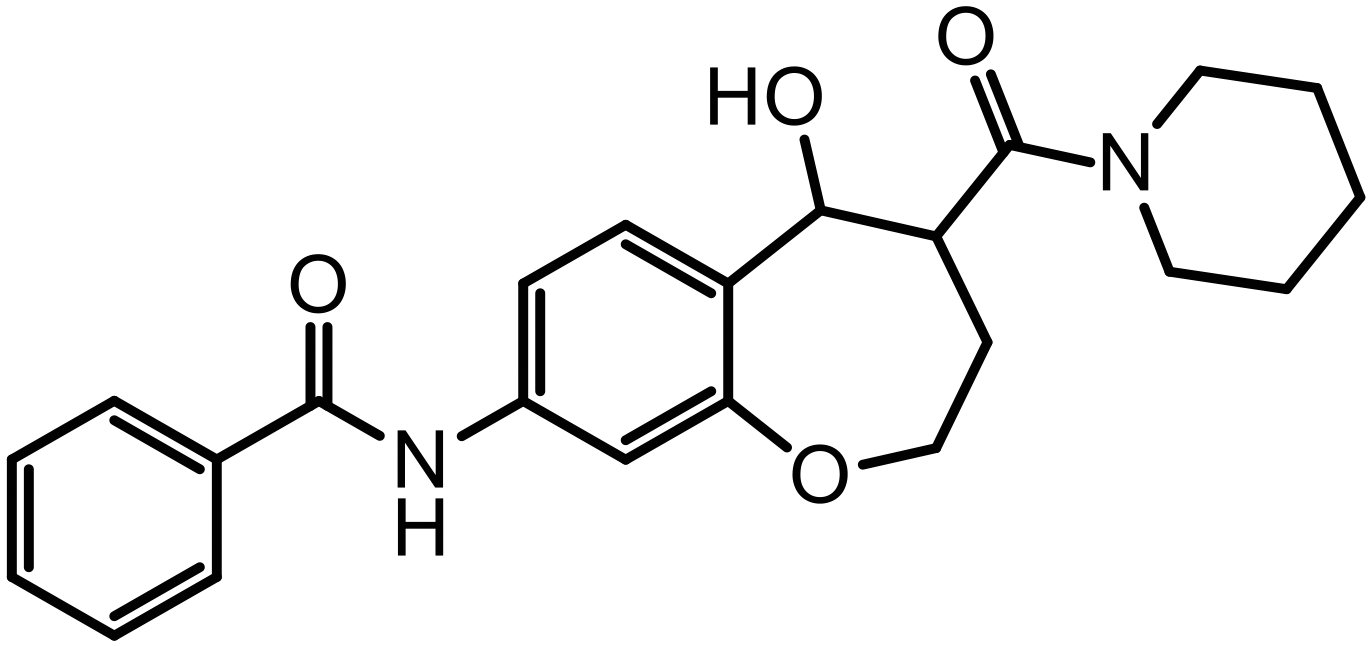
 According to *General procedure B*, a mixture of 9a (0.670 g, 1.71 mmol), NaBH_4_ (0.323 g, 8.54 mmol) and MeOH_._ (50 mL) was stirred at rt for 1 h After workup the residue was purified by fc (*ø* 4 cm, *h* = 15 cm, cyclohexane : ethyl acetate 1 : 2, *R*_f_ = 0.33 (cyclohexane : ethyl acetate 1 : 1)). Colorless solid, mp 118 °C, yield 599 mg (89%). C_23_H_26_N_2_O_4_, *M*_r_ = 394.5. MS (EM): *m*/*z* (%) calcd. for C_23_H_27_N_2_O_4_ 395.1971, found 395.1995. ^1^H NMR (CDCl_3_): *δ* [ppm] = 1.38–1.68 (m, 6H, 3-H, 4-H, 5-H_piperidine_), 1.78–1.86 (m, 0.3H, 3-H·), 1.99–2.05 (m, 0.7H, 3-H), 2.15–2.42 (m, 1H, 3-H), 2.81 (ddd, *J* = 12.2/9.2/3.3 Hz, 0.3H, 4-H·), 3.12 (dd, *J* = 11.9/5.4 Hz, 0.7 H, 4-H), 3.22–3.62 (m, 4.4H, 2-H, 6-H_piperidine_ and 2-H (0.7H)), 3.92 (d, *J* = 3.2 Hz, 0.3H, 2-H_piperidine_·), 4.08 (dt, 10.2/5.0 Hz, 0.3H, 2-H·), 4.34 (dt, *J* = 12.3 /3.9 Hz, 0.7H, 2-H), 4.45 (ddd, *J* = 12.8/9.0/4.9 Hz, 0.3H, 2-H·), 4.89 (2 × s, 0.7H, 5-H), 5.16 (2 × d, *J* = 9.1 Hz, 0.3H, 5-H·), 7.18–7.27 (m, 2H, 7-H, 9-H), 7.37–7.53 (m, 3H, 3-H, 4-H, 5-H_Bz_), 7.59 (dd, *J* = 8.4/0.8 Hz, 0.3H, 2-H or 6-H_Bz_·), 7.75–7.85 (m, 2, 7H, 2-H, 6-H_Bz_, and 6-H). Signals for the OH and NH protons are not seen in the spectrum. Ratio of *cis*-10a : *trans*-40a is 70 : 30. Signals of *trans*-isomer are marked with ·. Ratio of rotamers 50 : 50. ^13^C NMR (CDCl_3_): *δ* [ppm] = 24.7, 25.8, 27.0 (3C, C-3, C-4, C-5_piperidine_), 29.3 (0.7C, C-3), 32.9 (0.3C, C-3·), 41.5 (0.7C, C-4), 43.0, 43.2, 47.1 (2C, C-2, C-6_piperidine_), 47.4 (0.3C, C-4·), 70.8 (0.3C, C-5·), 71.1 (0.7C, C-2), 72.2 (0.3C, C-2·), 74.1 (0.7C, C-5), 112.5, 113.2, 114.6, 115.8 (2C, C-7, C-9), 127.2 (2C, C-2, C-6_Bz_), 129.0 (2C, C-3, C-5_Bz_), 131.6 (1C, C-5a), 132.1 (1C, C-6), 133.5 (1C, C-4_Bz_), 135.1 (1C, C-1_Bz_), 138.0 (0.3C, C-8·), 138.8 (0.7C, C-8), 157.5 (0.7C, C-9a), 161.2 (0.3C, C-9a·), 165.9 (1C, NHC=O), 173.1 (0.3C, O=C–N_pip_·), 173.1 (0.7C, O=C–N_pip_). Ratio of diastereomers 70 : 30. IR (neat): *ν̃* [cm^−1^] = 3286 (OH), 2936 (C–H), 2854 (C–H), 1651 (C=O). Purity (HPLC): 90.9%, *t*_R_ = 17.54 and 17.98 min.

#### *N*-{*cis*-5-Hydroxy-4-[(4-phenylpiperidin-1-yl)carbonyl]-2,3,4,5-tetrahydro-1-benzoxepin-8-yl}benzamide (*cis*-10b) and *N*-{*trans*-5-hydroxy-4-[(4-phenylpiperidin-1-yl)carbonyl]-2,3,4,5-tetrahydro-1-benzoxepin-8-yl}benzamide (*trans*-10b)


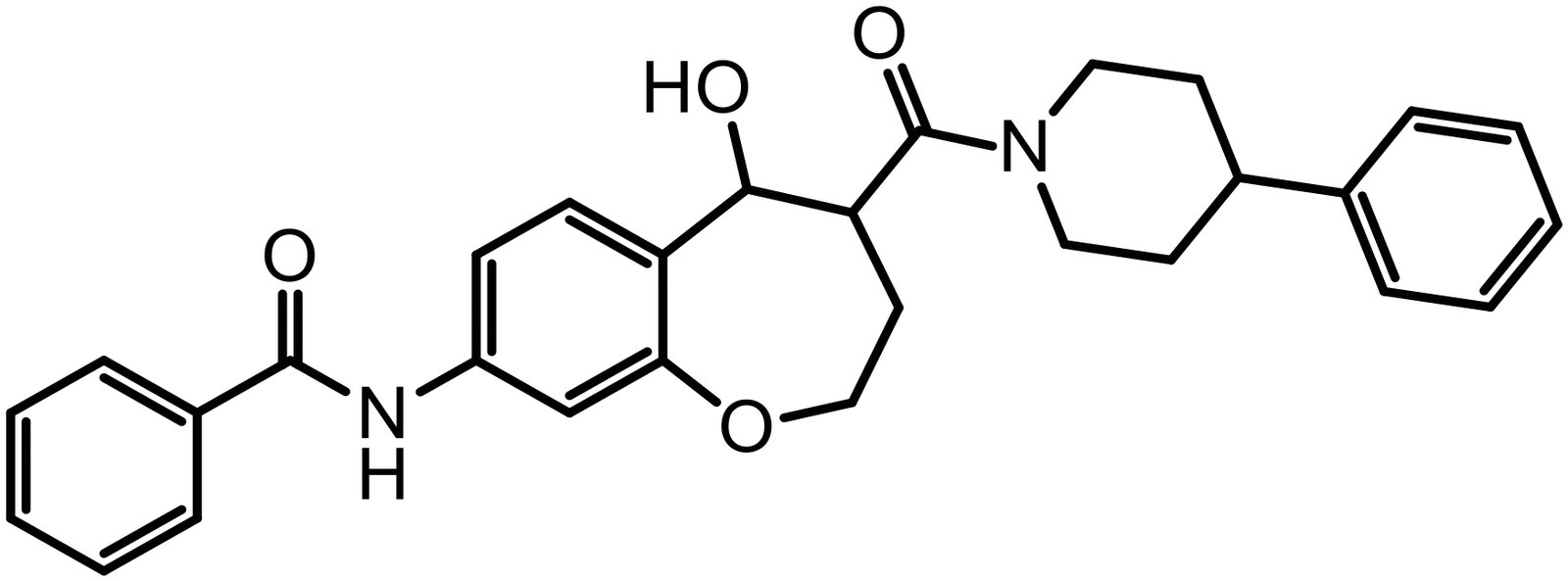
 According to *General procedure B*, a mixture of 9b (1.658 g, 3.54 mmol), NaBH_4_ (0.67 g, 17.7 mmol) and MeOH was stirred at rt for 4 h. After workup, the residue was purified by fc two times (*ø* 5 cm, *h* = 15 cm, cyclohexane : ethyl acetate 1.5 : 1; *ø* 4 cm, *h* = 15 cm, cyclohexane : ethyl acetate 5 : 1 + 0.1% *N*,*N*-dimethylethylamine). Flash chromatography led to fractions with diastereomerically pure products *cis*-10b and *trans*-10b.

*cis*-10b (*R*_f_ = 0.22, cyclohexane : ethyl acetate 1 : 1): colorless solid, mp 112–115 °C, yield 345 mg (21%). C_29_H_30_N_2_O_4_, *M*_r_ = 470.6. MS (EM): *m*/*z* calcd. for C_29_H_31_N_2_O_4_ 471.2284, found 471.2237. ^1^H NMR (CDCl_3_): *δ* [ppm] = 1.47–1.65 (m, 2H, 3-H, 5-H_piperidine_), 1.81–1.92 (m, 2H, 3-H, 5-H_piperidine_), 2.02 (ddd, *J* = 13.9/9.1/4.7 Hz, 1H, 3-H), 2.26–2.48 (m, 1H, 3-H), 2.54–2.84 (m, 2H, 2-H, 4-H_piperidine_), 3.01–3.26 (m, 2H, 6-H_piperidine_ and 4-H), 3.91 (d, *J* = 13.4 Hz, 1H, 6-H_piperidine_), 3.99–4.12 (m, 1H, 2-H), 4.38–4.52 (m, 1H, 2-H), 4.69–4.78 (m, 1H, 2-H_piperidine_), 4.83–4.86 (m, 1H, OH), 4.90 (s, 0.5H, 5-H), 4.94 (s, 0.5H, 5-H) 7.09–7.29 (m, 8H, C_6_H_5_, 6-H, 7-H, 9-H), 7.35–7.50 (m, 3H, 3-H, 4-H, 5-H_Bz_), 7.75–7.82 (m, 2H, 2-H and 6-H_Bz_), 8.00 (s, 1H, NH). Ratio of rotamers is 50 : 50. ^13^C NMR (CDCl_3_): *δ* [ppm] = 29.33 (1C, C-3), 33.0, 34.3 (2C, C-3, C-5_piperidine_), 41.6 (1C, C-4), 42.7, 46.7 (3C, C-2, C-4, C-6_piperidine_), 72.1 (1C, C-2), 74.2 (1C, C-5), 112.8 (1C, C-9), 114.9 (1C, C-7), 126.7 (3C, C-5a and C-2, C-6-C_6_H_5_), 126.9 (1C, C-4-C_6_H_5_), 127.3 (2C, C-2, C-6_Bz_), 128.9 (4C, C-3, C-5 C_6_H_5_ and C-3, C-5_Bz_), 132.1 (1C, C-6), 133.4 (1C, C-4_Bz_), 135.0 (1C, C-1_Bz_), 139.0 (1C, C-8), 144.9 (1C, C-1-C_6_H_5_), 161.1 (1C, C-9a), 166.0 (1C, NHC=O), 174.9 (1C, R_2_N–C=O). IR (neat): *ν̃* [cm^−1^] = 3302 (N–H, O–H), 2920 (C–H), 2851 (C–H), 1667 (C=O). Purity (HPLC): 99.2%, *t*_R_ = 20.33 min.

*trans*-10b (*R*_f_ = 0.11, cyclohexane : ethyl acetate 1 : 1): colorless solid, yield 100 mg (6%). ^1^H NMR (CDCl_3_): *δ* [ppm] = 1.39–1.73 (m, 2H, 3-H, 5-H_piperidine_), 1.78–1.95 (m, 3H, 3-H, 5-H_piperidine_ and 3-H), 2.17–2.43 (m, 1H, 3-H), 2.56–2.77 (m, 2H, 2-H, 4-H_piperidine_), 2.83–2.94 (m, 1H, 6-H_piperidine_), 2.99–3.20 (m, 1H, 4-H), 3.60 (t, *J* = 12.6 Hz, 1H, 2-H), 3.88 (dd, *J* = 16.4/7.4 Hz, 1H, 6-H_piperidine_), 4.36 (dd, *J* = 12.3/3.6 Hz, 1H, 2-H), 4.80 (d, *J* = 13.4 Hz, 1H, 2-H_piperidine_), 5.19 (d, *J* = 9.1 Hz, 1H, 5-H), 5.14–5.21 (m, 6H, C_6_H_5_ and 7-H), 7.37–7.55 (m, 4H, 3-H, 5-H_Bz_, 6-H, 9-H), 7.61 (t, *J* = 7.0 Hz, 1H, 4-H_Bz_), 7.75–7.87 (m 3H, 2-H, 6-H_Bz_ and NH). A signal for the OH proton is not seen in the spectrum. Purity (HPLC): 87.9%, *t*_R_ = 20.24 min.

#### 4.4.1. *N*-{*cis*-5-Hydroxy-4-[(4-phenylpiperazin-1-yl)carbonyl]-2,3,4,5-tetrahydro-1-benzoxepin-8-yl}benzamide (*cis*-10c) and *N*-{*trans*-5-hydroxy-4-[(4-phenylpiperazin-1-yl)carbonyl]-2,3,4,5-tetrahydro-1-benzoxepin-8-yl}benzamide (*trans*-10c)


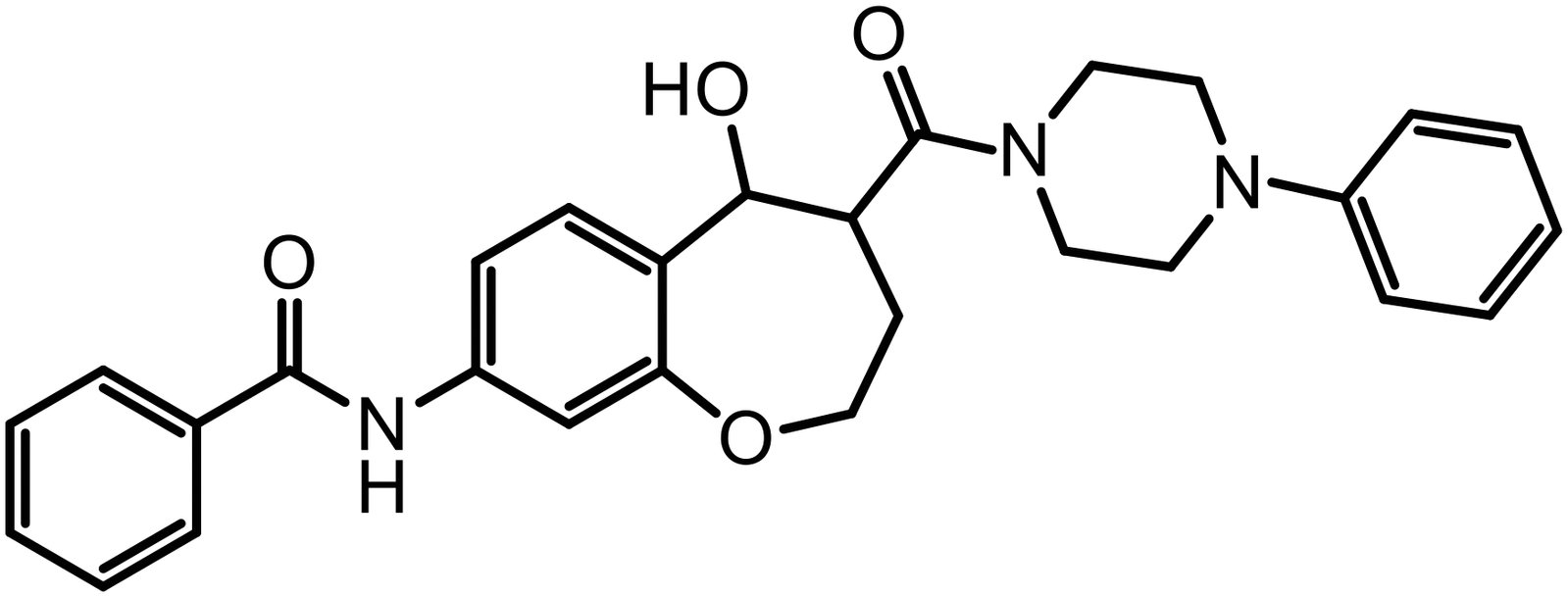
 According to *General procedure B*, a mixture of 9c (0.546 g, 1.16 mmol), NaBH_4_ (0.22 g, 5.81 mmol) and MeOH (100 mL) was stirred at rt for 2 h. After workup, the residue was purified by fc (*ø* 3 cm, *h* = 15 cm, cyclohexane : ethyl acetate 1 : 2 + 0.1% *N*,*N*-dimethylethylamine, *R*_f_ = 0.5). Colorless solid, mp 143 °C, yield 327 mg (60%). C_28_H_29_N_3_O_4_, *M*_r_ = 471.6. MS (EM): *m*/*z* calcd. for C_28_H_30_N_3_O_4_ 472.2236, found 472.2249. ^1^H NMR (CDCl_3_): *δ* [ppm] = 1.43–1.79 (m, 4H, 3-H, 5-H_piperazine_), 1.78–1.92 (m, 0.67H, 3-H), 2.05 (ddd, *J* = 14.2/9.8/5.5 Hz, 0.33H, 3-H·), 2.22–2.49 (m, 1.33H, 3-H· and 4-H), 2.88 (ddd, *J* = 12.4/9.3/3.3 Hz, 0.67H, 3-H), 3.05–3.28 (m, 3H, 2-H, 6-H_piperazine_), 3.49–3.98 (m, 2.34H, 2-H, 6-H_piperazine_ and 2-H (0.67H) and NH (0.67H)), 4.11 (dt, *J* = 12.2/4.9 Hz, 0.33H, 2-H·), 4.37 (dt, *J* = 12.3/3.8 Hz, 0.67H, 2-H), 4.46 (ddd, *J* = 13.0/9.1/4.1 Hz, 0.33H, 2-H·), 4.65 (s (broad), 0.33H, NH·), 4.94 (s, 0.33H, 5-H·), 5.17 (d, *J* = 9.1 Hz, 0.67H, 5-H), 6.80–7.08 (m, 3H, 2-H, 6-H–C_6_H_5_ and 7-H), 7.20–7.31 (m, 3H, 3-H, 5-H–C_6_H_5_ and 9-H), 7.36–7.52 (m, 4H, 3-H, 4-H, 5-H_Bz_, 4-H–C_6_H_5_), 7.58 (d, *J* = 8.5 Hz, 0.33H, 6-H·), 7.75–7.85 (m, 2.67H, 2-H, 6-H_Bz_ and 6-H). A signal for the OH proton is not seen in the spectrum. Ratio of *trans*-10c : *cis*-10c is 67 : 33. Signals of *cis*-10c are marked with ·. ^13^C NMR (CDCl_3_): *δ* [ppm] = 29.3 (0.33C, C-3·), 32.8 (0.67C, C-3), 41.7, 42.0, 46.0, 47.2, 49.7 (4C, C-2, C-3, C-5 C-6_piperazine_), 50.0 (0.67C, C-4), 50.2 (0.33C, C-4·), 71.0 (1C, C-5), 72.1 (0.67C, C-2), 73.9 (0.33C, C-2·), 112.7 (0.33C, C-9·), 113.4 (0.67C, C-9), 114.7 (0.33C, C-7·), 115.9 (0.67C, C-7), 116.9 (2C, C-2, C-6-C_6_H_5_), 121.0 (1C, C-4-C_6_H_5_), 126.5 (1C, C-5a), 127.0, 127.5 (2C, C-2, C-6_Bz_), 129.0, 129.5 (4C, C-3, C-5-C_6_H_5_ and C-3, C-5_Bz_), 131.5 (1C, C-6), 132.1 (1C, C-4_Bz_), 133.4, 135.0 (1C, C-1_Bz_), 138.1 (0.67H, C-8), 138.9 (0.33C, C-8·), 151.0 (1C, C-1-C_6_H_5_), 157.4 (1C, C-9a), 166.0 (0.33C, HNC=O·), 166.0 (0.67C, NHC=O), 173.4 (0.67C, NC=O), 175.0 (0.33C, NC=O·). Ratio of diastereomers is 76 : 33. IR (neat): *ν̃* [cm^−1^] = 3305 (O–H, N–H), 2924 (C–H), 2854 (C–H), 1597 (C=O). Purity (HPLC): 59.8%, *t*_R_ = 16.92 min and 36.8%, *t*_R_ = 17.43 min.

#### *N*-{*cis*-4-[(4-Benzylpiperidin-1-yl)carbonyl]-5-hydroxy-2,3,4,5-tetrahydro-1-benzoxepin-8-yl}benzamide (*cis*-10d) and *N*-{*trans*-4-[(4-benzylpiperidin-1-yl)carbonyl]-5-hydroxy-2,3,4,5-tetrahydro-1-benzoxepin-8-yl}benzamide (*trans*-10d)


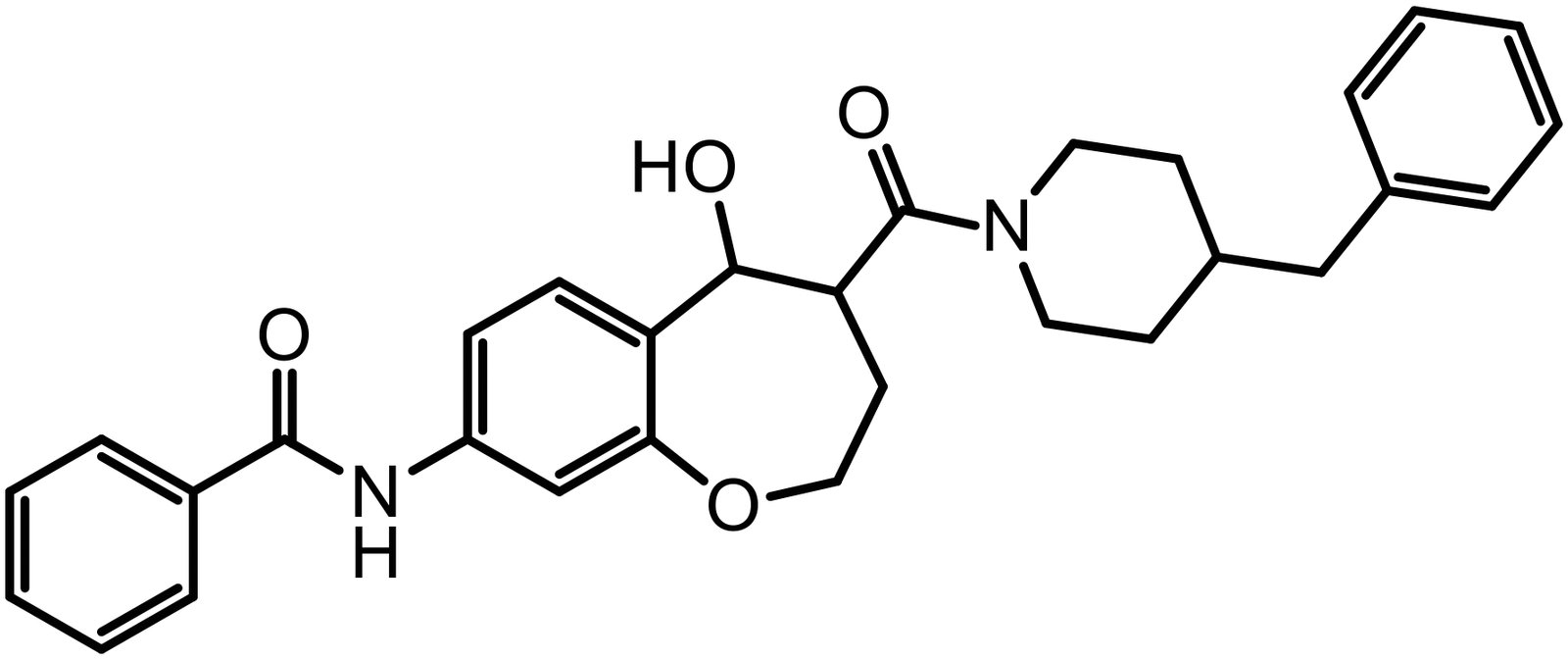
 According to *General procedure B*, a mixture of 9d (2.18 g, 4.53 mmol), NaBH_4_ (0.857 g, 22.6 mmol) and MeOH_abs._ (50 mL) was stirred at rt for 2 h. After workup, the residue was purified by fc (*ø* 3 cm, *h* = 15 cm, cyclohexane : ethyl acetate 1 : 1.2 + 0.1% *N*,*N*-dimethylethylamine, *R*_f_ = 0.12 (cyclohexane : ethyl acetate 1 : 1 + 0.1% *N*,*N*-dimethylethylamine)). Colorless solid, mp 91 °C, yield 3.68 g (81%). C_30_H_32_N_2_O_4_, *M*_r_ = 484.6. MS (EM): *m*/*z* calcd. for C_30_H_33_N_2_O_4_ 485.2440, found 485.2433. ^1^H NMR (CDCl_3_): *δ* [ppm] = 1.00–1.26 (m, 2H, 3-H, 5-H_piperidine_), 1.60–1.88 (m, 3.63H, 3-H, 4-H, 5-H_piperidine_ and 3-H (0.63H)), 1.97–2.07 (m, 0.37H, 3-H·), 2.11–2.39 (m, 1H, 3-H), 2.41–2.57 (m, 3H, C*H*_2_C_6_H_5_ and 2-H_piperidine_), 2.76–2.85 (m, 0.63H, 4-H), 2.86–3.00 (m, 1H, 6-H_piperidine_), 3.06–3.16 (m, 0.37H, 4-H·), 3.50–3.65 (m, 0.63H, 2-H), 3.65–3.82 (m, 1H, 6-H_piperidine_), 3.88 (s (broad), 0.37H, NH·), 4.00–4.12 (m, 0.37H, 2-H·), 4.26–4.38 (m, 0.63H, 2-H), 4.39–4.51 (m, 0.37H, 2-H·), 4.51–4.65 (m, 1H, 2-H_piperidine_), 4.88 (s, 0.14H, 5-H·), 4.92 (s, 0.14H, 5-H·), 5.16 (d, *J* = 9.0 Hz, 0.63H, 5-H), 7.04–7.30 (m, 7H, C_6_H_5_, 7-H, 9-H), 7.36–7.51 (m, 3H, 3-H, 4-H, 5-H_Bz_), 7.57 (d, *J* = 8.9 Hz, 0.33H, 6-H), 7.61 (d, *J* = 8.4 Hz, 0.34H, 6-H) 7.85–7.68 (m, 2.37H, 2-H, 6-H_Bz_ and 6-H·). Signals for NH (0.63H) or OH protons are not seen in the spectrum. Ratio of *trans*-10d : *cis*-10d is 63 : 37. Signals of *cis*-10d are marked with ·. IR (neat): *ν̃* [cm^−1^] = 3230 (O–H), 2920 (C–H), 2862 (C–H), 2843 (C–H), 1601 (C=O). Purity (HPLC): 99.1%, *t*_R_ = 20.69 min and 21.09 min.

#### *cis*-8-Benzamido-*N*,*N*-diethyl-2,3,4,5-tetrahydro-1-benzoxepin-4-carboxamide (*cis*-10e) and *trans*-8-benzamido-*N*,*N*-diethyl-2,3,4,5-tetrahydro-1-benzoxepin-4-carboxamide (*trans*-10e)


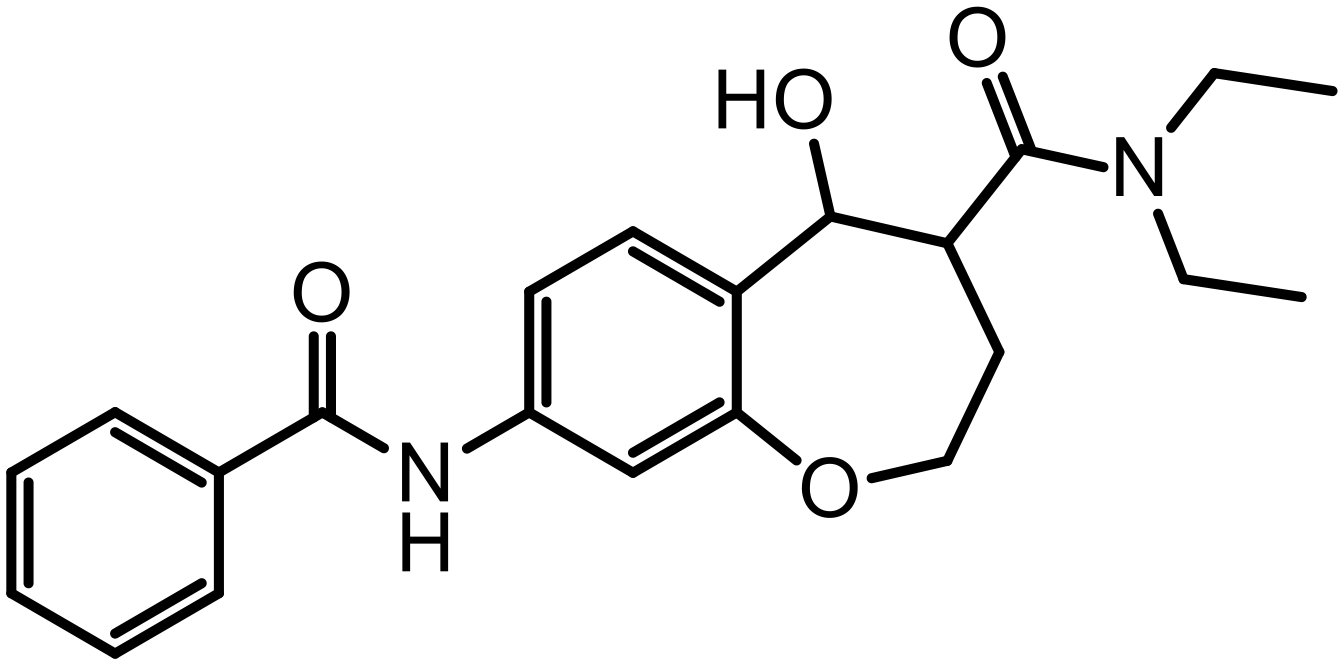
 According to *General procedure B*, a mixture of 9e (0.707 g, 1.86 mmol), NaBH_4_ (0.35 g, 9.3 mmol) and MeOH (25 mL) was stirred at rt for 1 h. After workup the mixture was purified by fc (*ø* 4 cm, *h* = 15 cm, cyclohexane : ethyl acetate 3 : 1, *R*_f_ = 0.19 and 0.17 (cyclohexane : ethyl acetate 3 : 2)) and *cis*-10e and *trans*-10e were isolated in diastereomerically pure form. In addition to the pure products a mixture of diastereomers was isolated 388 mg (54%). Total yield 0.71 g (99%).

##### *cis*-10e

Pale yellow resin, yield 0.238 g (33%). C_22_H_26_N_2_O_4_, *M*_r_ = 382.5. MS (EM): calcd. for C_22_H_27_N_2_O_4_ 383.1971, found 383.1961. ^1^H NMR (CDCl_3_): *δ* [ppm] = 1.12–1.21 (m, 6H, N(CH_2_C*H*_3_)_2_), 2.05–2.15 (m, 2H, 3-H), 2.41–2.57 (m, 1H, 4-H), 3.03–3.16 (m, 1H, N(C*H*_2_CH_3_)_2_), 3.20–3.34 (m, 1H, N(C*H*_2_CH_3_)_2_), 3.33–3.44 (m, 1H, N(C*H*_2_CH_3_)_2_), 3.52 (dq, *J* = 14.2/7.1 Hz, 1H, N(C*H*_2_CH_3_)_2_), 4.12–4.19 (m, 1H, 2-H), 4.53 (ddd, *J* = 12.5/8.8/4.0 Hz, 1H, 2-H), 4.90 (s, 1H, 5-H), 7.24–7.31 (m, 2H, 7-H and 9-H), 7.44–7.60 (m, 3H, 3-H, 4-H, 5-H_Bz_), 7.82–7.90 (m, 3H, 2-H, 6-H_Bz_ and 9-H). Signals for OH and NH protons are not seen in the spectrum. ^13^C NMR (CDCl_3_): *δ* [ppm] = 13.2 (1C, N(CH_2_*C*H_3_)_2_), 15.2 (1C, N(CH_2_*C*H_3_)_2_), 29.6 (1C, C-3), 40.5 (1C, N(*C*H_2_CH_3_)_2_), 41.8 (1C, N(*C*H_2_CH_3_)_2_), 42.5 (1C, C-4), 72.3 (1C, C-5), 74.3 (1C, C-2), 112.8 (1C, C-9), 114.7 (1C, C-7), 126.9 (1C, C-5a), 127.3 (2C, C-2, C-6 Bz), 129.0 (2C, C-3, C-5 Bz), 132.1 (1C, C-6), 133.4 (1C, C-4 Bz), 135.1 (1C, C-1 Bz), 138.9 (1C, C-8), 161.2 (1C, C-9a), 165.9 (1C, NHC=O), 176.1 (1C, CH*C*=ONEt_2_). IR (neat): *ν̃* [cm^−1^] = 3306 (N–H), 2970 (C–H), 2935 (C–H), 1651 (C=O). Purity (HPLC): 97.9%, *t*_R_ = 17.95 min.

##### *trans*-10e

Pale yellow resin, yield 83 mg (12%). C_22_H_26_N_2_O_4_, *M*_r_ = 382.5. MS (EM): calcd. for C_22_H_27_N_2_O_4_ 383.1971, found 383.1966. ^1^H NMR (CDCl_3_): *δ* [ppm] = 1.16 (2 × t, *J* = 7.1 Hz, 6H, N(CH_2_C*H*_3_)_2_), 1.82–1.88 (m, 1H, 3-H), 2.28–2.43 (m, 1H, 4-H), 2.80 (ddd, *J* = 12.3/9.3/3.2 Hz, 1H, 3-H), 3.24–3.40 (m, 3H, N(C*H*_*2*_CH_3_)_2_), 3.52 (dq, *J* = 14.2/7.1 Hz, 1H, N(C*H*_*2*_CH_3_)_2_), 3.64 (td, *J* = 12.1/2.0 Hz, 1H, 2-H), 4.43 (dt, *J* = 12.3/3.8 Hz, 1H, 2-H), 5.22 (d, *J* = 9.3 Hz, 1H, 5-H), 7.32 (dd, *J* = 8.4/2.2 Hz, 1H, 7-H), 7.44–7.58 (m, 4H, 3-H, 4-H, 5-H_Bz_ and 9-H), 7.66 (d, *J* = 8.4 Hz, 1H, 6-H), 7.81 (s (broad), 1H, NH), 7.83–7.90 (m, 2H, 2-H, 6-HBz). A signal for the OH proton is not seen in the spectrum. Purity (HPLC): 97.1%, *t*_R_ = 17.35 min.

#### *cis*-8-Benzylamino-4-[(piperidin-1yl)methyl]-2,3,4,5-tetrahydro-1-benzoxepin-5-ol (*cis*-11a) and *trans*-8-benzylamino-4-(piperidine-1yl)methyl-2,3,4,5-tetrahydro-1-benzoxepin-5-ol (*trans*-11a)


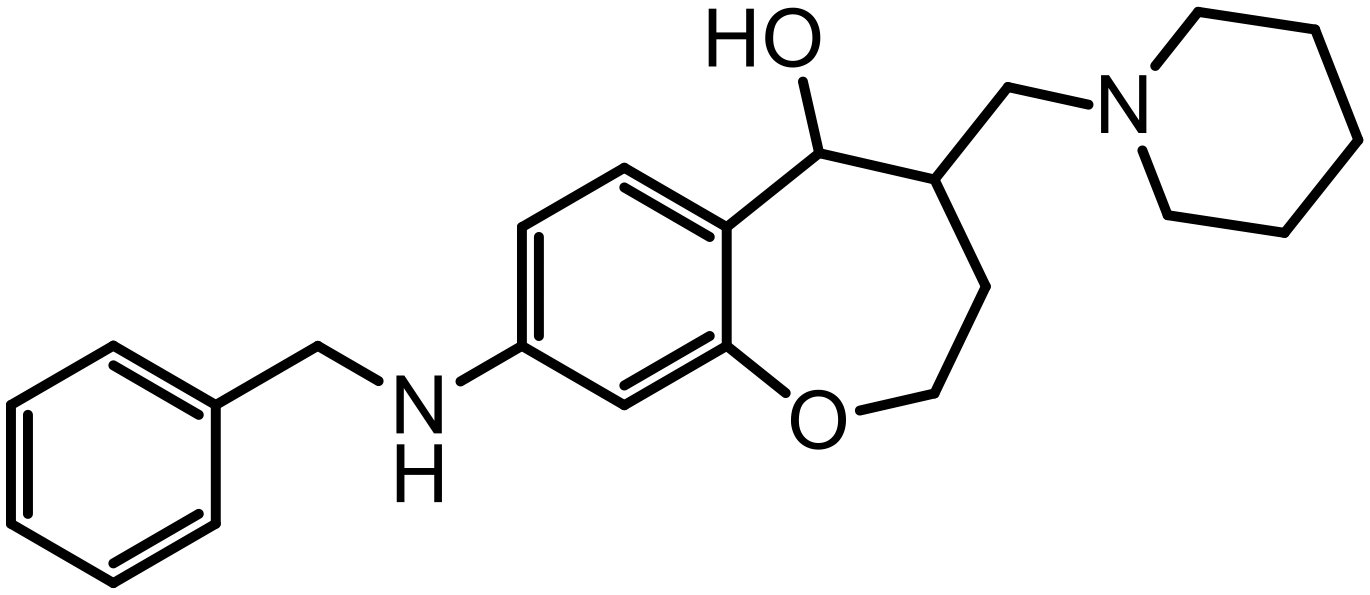
 According to *General procedure C*, a mixture of 10a (0.471 g, 1.20 mmol), LiAlH_4_ (1 M in THF, 6 mL, 6.0 mmol) and THF_abs._ (20 mL) was heated to reflux for 5 h. After workup, the residue was purified by fc (*ø* 4 cm, *h* = 15 cm, cyclohexane : ethyl acetate 1 : 2 + 0.1% *N*,*N*-dimethylethylamine, *R*_f_ = 0.65). Colorless solid, mp 108–112 °C, yield 599 mg (89%). C_23_H_30_N_2_O_2_, *M*_r_ = 366.5. MS (EM): *m*/*z* (%) calcd. for C_23_H_31_N_2_O_2_ 367.2386, found 367.2373. ^1^H NMR (CDCl_3_): *δ* [ppm] = 1.30–1.42 (m, 3H, 3-H (1H), 4-H_piperidine_), 1.42–1.59 (m, 4.7H, 3-H (0.7H) and 3-H, 5-H_piperidine_), 1.64–1.75 (m, 0.3H, 3-H·), 1.77–1.87 (m, 0.3H, 4-H·), 1.97 (d, *J* = 11.4 Hz, 0.7H, CH_2_N), 2.01–2.11 (m, 2H, 2-H, 6-H_piperidine_), 2.26 (dd, *J* = 12.7/2.8 Hz, 0.3H, CH_2_N·), 2.44 (d, *J* = 12.0 Hz, 0.7H, CH_2_N), 2.39–2.57 (m, 2.7H, 4-H (0.7H) and 2-H, 6-H_piperidine_), 2.65 (t, *J* = 12.7 Hz, 0.3H, CH_2_N·), 3.48–3.58 (m, 1H, 2-H), 3.98–4.11 (m, 0.7H, 2-H), 4.18–4.22 (m, 0.3H, 2-H·), 4.23 (s, 2H, NCH_2_-Ph), 4.73 (d, *J* = 9.1 Hz, 0.3H, 5-H·), 5.00 (s, 0.7H, 5-H), 6.22 (d, *J* = 2.4 Hz, 0.3H, 9-H·), 6.25 (d, *J* = 2.4 Hz, 0.7H, 9-H), 6.38 (dd, *J* = 8.3/2.4 Hz, 1H, 7-H), 7.20–7.37 (m, 5.7H, C_6_H_5 Bn_, and 6-H), 7.48 (dd, *J* = 8.4/0.9 Hz, 0.3H, 6-H·). Signals for NH and OH protons are not seen in the spectrum. Ratio of *cis*-11a : *trans*-11a according to ^1^H NMR spectrum is 70 : 30. Signals for *trans*-11a are marked with ·. ^13^C NMR (CDCl_3_): *δ* [ppm] = 24.4 (1C, C-4_piperidine_), 26.4 (2C, C-3, C-5_piperidine_), 32.6 (0.7C, C-3), 34.5 (0.3C, C-3·), 36.9 (0.7C, C-4), 38.0 (0.3C, C-4·), 48.8 (2C, C-2, C-6_piperidine_), 55.5 (1C, NCH_2_-Ph), 60.3 (0.7C, CH_2_N_piperidine_), 65.9 (0.3C, CH_2_N_piperidine_·), 68.5 (0.7C, C-2), 71.8 (0.3C, C-2·), 74.5 (0.7C, C-5), 76.0 (0.3C, C-5·), 105.2 (0.3C, C-9·), 105.7 (0.7C, C-9), 108.6 (1C, C-7), 125.0 (1C, C-5a), 127.5 (1C, C-4_Bn_), 127.9 (2C, C-2, C-6_Bn_), 128.6 (1C, C-6), 128.8 (2C, C-3, C-5_Bn_), 139.5 (1C, C-1_Bn_), 148.6 (1C, C-8), 157.9 (1C, C-9a). Ratio of diastereomers is 70 : 30. IR (neat): *ν̃* [cm^−1^] = 3290 (OH), 2924 (C–H), 2862 (C–H), 1612 (N–H). Purity (HPLC): 94.2%, *t*_R_ = 14.99 and 18.54 min.

#### *cis*-8-Benzylamino-4-[(4-phenylpiperidin-1-yl)methyl]-2,3,4,5-tetrahydro-1-benzoxepin-5-ol (*cis*-11b) and *trans*-8-benzylamino-4-[(4-phenylpiperidin-1-yl)methyl]-2,3,4,5-tetrahydro-1-benzoxepin-5-ol (*trans*-11b)


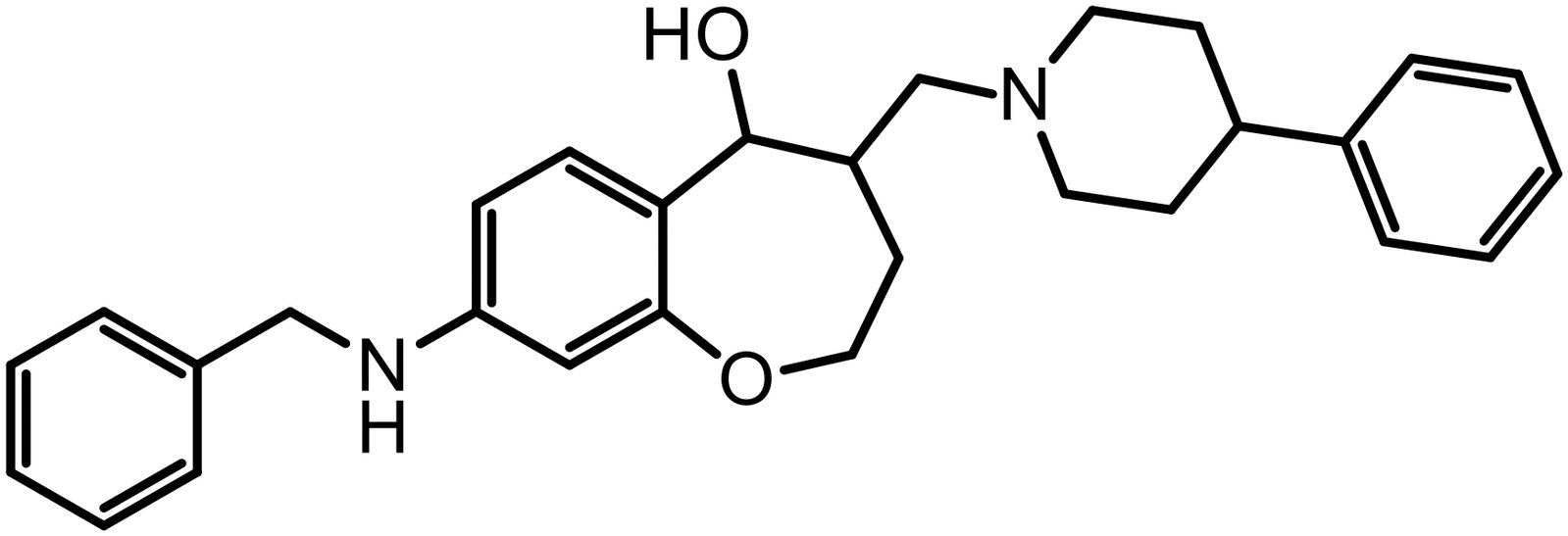
 According to *General procedure C*, a mixture of 10b (162 mg, 0.34 mmol), LiAlH_4_ (1 M in THF, 1.72 mL, 1.72 mmol) and THF_abs._ (1.7 mL) was heated to reflux for 5 h. After workup, the residue was purified by fc (*ø* 3 cm, *h* = 15 cm, cyclohexane : ethyl acetate 2 : 1 + 0.1% *N*,*N*-dimethylethylamine, *R*_f_ = 0.66 (cyclohexane : ethyl acetate 1 : 1 + 0.1% *N*,*N*-dimethylethylamine)). Colorless solid, mp 102–105 °C, yield 48 mg (31%). C_29_H_34_N_2_O_2_, *M*_r_ = 442.6. MS (EM): *m*/*z* calcd. for C_29_H_35_N_2_O_2_ 443.2699, found 443.2690. ^1^H NMR (CDCl_3_): *δ* [ppm] = 1.46–1.98 (m, 7H, 2-H or 6-H_piperidine_ (1H), 3-H, 5-H_piperidine_, 3-H), 1.99–2.22 (m, 2 × 0.8H, CH_2_N and 2-H or 6-H_piperidine_), 2.25–2.29 (0.2H, 4-H_piperidine_·), 2.30–2.36 (0.2H, CH_2_N·), 2.36–2.47 (m, 0.8H, 4-H_piperidine_), 2.48–2.59 (m, 2 × 0.8H, CH_2_N and 4-H), 2.63 (d, *J* = 11 Hz, 0.8H, 2-H or 6-H_piperidine_), 2.75 (t, *J* = 11.9 Hz, 0.2H, CH_2_N·), 2.82–2.95 (m, 0.2H, 4-H·), 3.02 (d, *J* = 10 Hz, 0.2H, 2-H or 6-H_piperidine_·), 3.26 (d, *J* = 10.6 Hz, 1H, 2-H or 6-H_piperidine_), 3.46–3.52 (m, 0.2H, 2-H or 6-H_piperidine_·), 3.57 (t, *J* = 11.2 Hz, 1H, 2-H), 3.91 (s (broad), 1H, NH), 4.06 (dt, *J* = 12.3/3.8 Hz, 0.8H, 2-H), 4.12–4.18 (m, 0.2H, 2-H·), 4.24 (s, 2H, N–CH_2_–Ph), 4.77 (d, *J* = 9.7 Hz, 0.2 H, 5-H·), 5.02 (s, 1H, 0.8H, 5-H), 6.24–6.30 (m, 1H, 9-H), 6.36 (dd, *J* = 8.2/2.3 Hz, 1H, 7-H), 7.04–7.35 (m, 10.8H, C_6_H_5_, C_6_H_5 Bn_ and 6-H), 7.50 (d, *J* = 8.5 Hz, 0.2H, 6-H·). A signal for the OH proton is not seen in the spectrum. Ratio of *cis*-11b : *trans*-11b according to ^1^H NMR spectrum is 80 : 20. Signals for *trans*-11b are marked with ·. IR (neat): *ν̃* [cm^−1^] = 3379 (N–H), 2939 (C–H), 2916 (C–H), 2870 (C–H), 1616 (N–H). Purity (HPLC): 95.2%, *t*_R_ = 18.09 min and 20.27 min.

In addition to the mixture of diastereomers, one fraction contained pure diastereomer *cis*-11b. *cis*-11b: C_29_H_34_N_2_O_2_, *M*_r_ = 442.6. MS (EM): *m*/*z* calcd. for C_29_H_35_N_2_O_2_ 443.2699, found 443.2713. ^1^H NMR (CDCl_3_): *δ* [ppm] = 1.43–1.75 (m, 5H, 3-H, 5-H_piperidine_, and 3-H), 1.81 (td, *J* = 11.8/2.3 Hz, 1H, 2-H_piperidine_), 1.91–2.13 (m, 3H, CH_2_N, 6-H_piperidine_ and 3-H), 2.29–2.39 (m, 1H, 4-H_piperidine_), 2.40–2.50 (m, 2H, CH_2_N and 4-H), 2.55 (d, *J* = 11.1 Hz, 1H, 6-H_piperidine_), 3.18 (d, *J* = 11.3 Hz, 1H, 2-H_piperidine_), 3.49 (t, *J* = 11.0 Hz, 1H, 2-H), 3.98 (dt, *J* = 12.3/4.0 Hz, 1H, 2-H), 4.16 (s, 2H, N–CH_2_–Ph), 4.94 (s, 1H, 5-H), 6.18 (d, *J* = 2.4 Hz, 1H, 9-H), 6.30 (dd, *J* = 8.3/2.4 Hz, 1H, 7-H), 7.02–7.27 (m, 11H, C_6_H_5 Bn_, C_6_H_5_, 6-H). Signals for Oh and NH protons are not seen in the spectrum. ^13^C NMR (CDCl_3_): *δ* [ppm] = 32.6 (1C, C-3), 33.5, 34.2 (2C, C-3, C-5_piperidine_), 37.2 (1C, C-4), 42.6 (1C, C-4_piperidine_), 48.8 (1C, N–CH_2_–Ph), 53.7 (1C, C-2_piperidine_), 56.2 (1C, C-6_piperidine_), 60.0 (1C, CH_2_N), 68.7 (1C, C-2), 74.6 (1C, C-5), 105.7 (1C, C-9), 108.7 (1C, C-7), 124.9 (1C, C-5a), 126.5 (1C, C-4_Bn_), 127.0 (2C, C-2, C-6_Bn_), 127.5 (1C, C-4-C_6_H_5_), 127.5 (4C, C-3, C-5_Bn_ and C-3, C-5-C_6_H_5_), 128.6, 128.9 (2C, C-2, C-6-C_6_H_5_), 128.6 (1C, C-6), 138.5 (1C, C-1_Bn_), 146.1 (1C, C-1-C_6_H_5_), 148.7 (1C, C-2), 158.0 (1C, C-9a).

#### *trans*-8-Benzylamino-4-[(4-phenylpiperidin-1-yl)methyl]-2,3,4,5-tetrahydro-1-benzoxepin-5-ol (*trans*-11b)


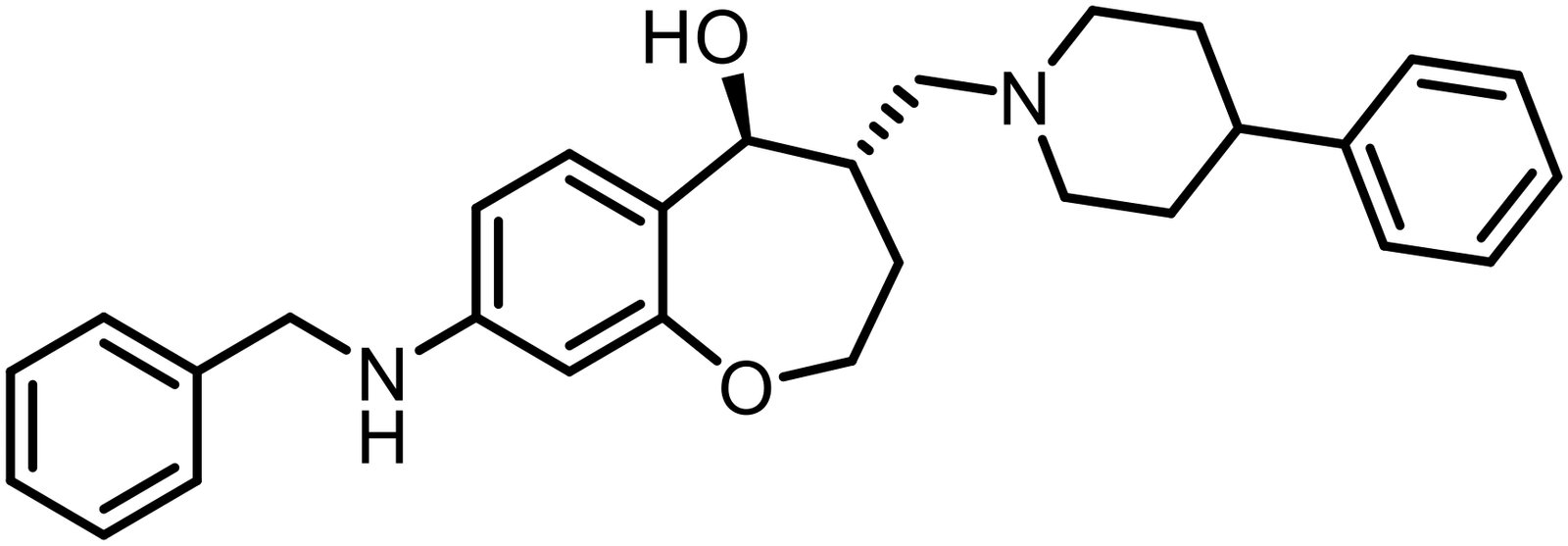
 According to *General procedure C*, a mixture of *trans*-10b (194 mg, 0.41 mmol), LiAlH_4_ (1 M in THF, 2 mL, 2 mmol) and THF_abs._ was heated to reflux for 5 h. After workup, the residue was purified by fc (*ø* 3 cm, cyclohexane : ethyl acetate 5 : 1 + 0.1% *N*,*N*-dimethylethylamine *R*_f_ = 0.84 (cyclohexane : ethyl acetate 1 : 1 + 0.1% *N*,*N*-dimethylethylamine)) and a diastereomerically pure fraction of *trans*-11b was isolated. Pale yellow resin, yield 162 mg (89%). C_29_H_34_N_2_O_2_, *M*_r_ = 442.6. ^1^H NMR (CDCl_3_): *δ* [ppm] = 1.56–1.96 (m, 8H, 2-H or 6-H, 3-H, 5-H_piperidine_, and 3-H), 2.14–2.27 (m, 1H, 4-H_piperidine_), 2.33 (dd, *J* = 12.7/2.7 Hz, 1H, CH_2_N), 2.42–2.52 (m, 1H, 4-H), 2.75 (t, *J* = 12.6 Hz, 1H, CH_2_N), 3.02 (d, *J* = 11.0 Hz, 1H, 2-H or 6-H_piperidine_), 3.26 (d, *J* = 10.0 Hz, 1H, 2-H or 6-H_piperidine_), 3.56 (ddd, *J* = 12.9/10.9/2.2 Hz, 1H, 2-H), 4.19–4.25 (m, 1H, 2-H), 4.24 (s, 2H, CH_2 Bn_), 4.76 (d, *J* = 9.2 Hz, 1H, 5-H), 6.23 (d, *J* = 2.4 Hz, 1H, 9-H), 6.39 (dd, *J* = 8.4/2.4 Hz, 1H, 7-H), 7.08–7.40 (10H, C_6_H_5 Bn_ and C_6_H_5_), 7.49 (dd, *J* = 8.4/0.8 Hz, 1H, 6-H). Signals for NH or OH protons are not seen in the spectrum. Purity (HPLC): 83.9%, *t*_R_ = 19.09 min.

#### *cis*-8-Benzylamino-4-[(4-phenylpiperazin-1yl)methyl]-5-hydroxy-2,3,4,5-tetrahydro-1-benzoxepin-5-ol (*cis*-11c) and *trans*-8-benzylamino-4-[(4-phenylpiperazin-1yl)methyl]-5-hydroxy-2,3,4,5-tetrahydro-1-benzoxepin-5-ol (*trans*-11c)


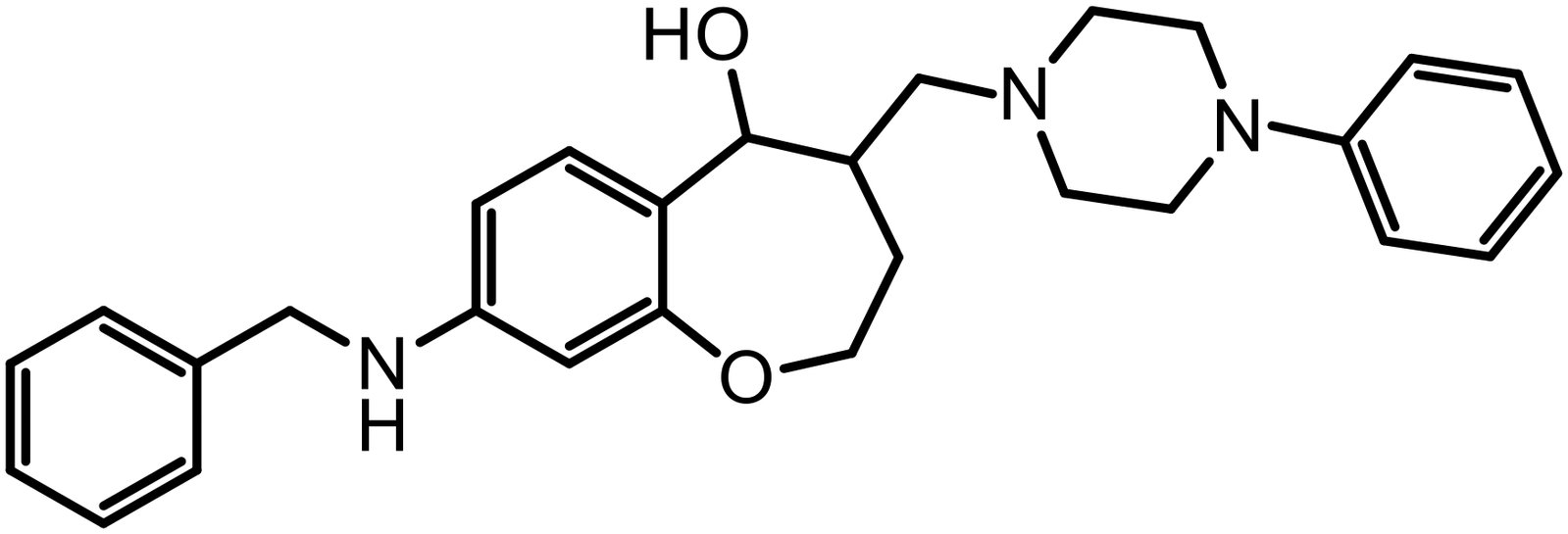
 According to *General procedure C*, a mixture of 10c (3.05 g, 6.48 mmol), LiAlH_4_ (1 M, 19 mL, 19 mmol) and THF_abs._ (30 mL) was heated to reflux for 4 h. After workup, the residue was purified by fc (*ø* 5 cm, *h* = 15 cm, cyclohexane : ethyl acetate 3 : 1 + 0.1% *N*,*N*-dimethylethylamine, *R*_f_ = 0.18 (cyclohexane : ethyl acetate 2 : 1 + 0.1% *N*,*N*-dimethylethylamine)). Colorless solid, mp 120–122 °C, yield 2.24 g (78%). C_28_H_33_N_3_O_2_, *M*_r_ = 443.6. MS (EM): *m*/*z* calcd. for C_28_H_34_N_3_O_2_ 444.2651, found 444.2639. ^1^H NMR (CDCl_3_): *δ* [ppm] = 1.44–1.84 (4H, CH_2 piperazine_), 1.84–2.01 (0.45H, 3-H·), 2.04–2.21 (m, 0.45H, CH_2_N·), 2.36 (2 × d, *J* = 12.5 Hz, 2 × 0.55H, CH_2_N), 2.45–2.55 (m, 1H, 3-H), 2.59 (t, *J* = 13.2 Hz, 0.45H, CH_2_N·), 2.68/2.83 (m, 1.55H, 3-H (0.55H) and 4-H), 3.05–3.29 (m, 4H, CH_2 piperazine_), 3.54–3.70 (m, 1H, 2-H), 3.99–4.10 (m, 0.55H, 2-H), 4.17–4.23 (m, 0.45H, 2-H·), 4.24 (s, 2H, N–CH_2 Bn_), 4.76, (d, *J* = 9.2 Hz, 0.55H, 5-H), 4.98 (s, 0.45H, 5-H·), 6.25 (dd, *J* = 9.9/2.4 Hz, 1H, 7-H), 6.34–6.39 (m, 1H, 9-H), 6.77–6.89 (m, 3H, 2-H, 4-H, 6-H_Ph_), 7.16–7.33 (m, 7.45H, C_6_H_5 Bn_, 3-H, 5-H_Ph_ and 6-H·), 7.44 (d, *J* = 8.0 Hz, 0.55H, 6-H). Signals for OH or NH protons are not seen in the spectrum. Ratio of *trans*-11c : *cis*-11c according to ^1^H NMR spectrum is 55 : 45. Signals for *cis*-11c are marked with ·. ^13^C NMR (CDCl_3_): *δ* [ppm] = 27.4 (1C, C-3), 32.4 (0.55C, C-4), 34.1 (0.45C, C-4·), 37.2 (0.55C, N–CH_2 Bn_), 38.00 (0.45C, N–CH_2 Bn_·), 48.7 (2 × 0.55C, C_piperazine_), 49.5 (2 × 0.45C, C_piperazine_·), 53.2 (2 × 0.45C, C_piperazine_·), 53.7 (2 × 0.55C, C_piperazine_), 59.9 (1C, CH_2_N), 69.1 (0.55C, C-2), 71.4 (0.45C, C-2·), 74.4, 76.2 (1C, C-5), 105.2 (0.45C, C-9·), 105.7 (0.55C, C-9), 108.6 (1C, C-7), 116.5 (2C, C-2, C-6_Ph_), 120.33 (1C, C-4_Ph_), 125.5 (1C, C-5a), 127.5 (1C, C-4_Bn_), 127.8 (3C, C-2, C-6_Bn_ and C-1_Bn_), 128.8 (1C, C-6), 128.9 (2C, C-3, C-5_Bn_), 129.4 (2C, C-3, C-5_Ph_), 139.4 (1C, C-8), 148.8 (1C, C-1_Ph_), 151.4 (0.45C, C-9a·), 158.1 (0.55C, C-9a). Ratio of diastereomers is 55 : 45. IR (neat): *ν̃* [cm^−1^] = 3240 (O–H, N–H), 3059 (C–H_arom._), 3028 (C–H_arom._) 2924 (C–H). Purity (HPLC): 95.6%, *t*_R_ = 17.80 min and 20.19 min.

#### *cis*-8-Benzylamino-4-[(4-benzylpiperidin-1yl)methyl]-2,3,4,5-tetrahydro-1-benzoxepin-5-ol (*cis*-11d) and *trans*-8-benzylamino-4-[(4-benzylpiperidin-1yl)methyl]-2,3,4,5-tetrahydro-1-benzoxepin-5-ol (*trans*-11d)


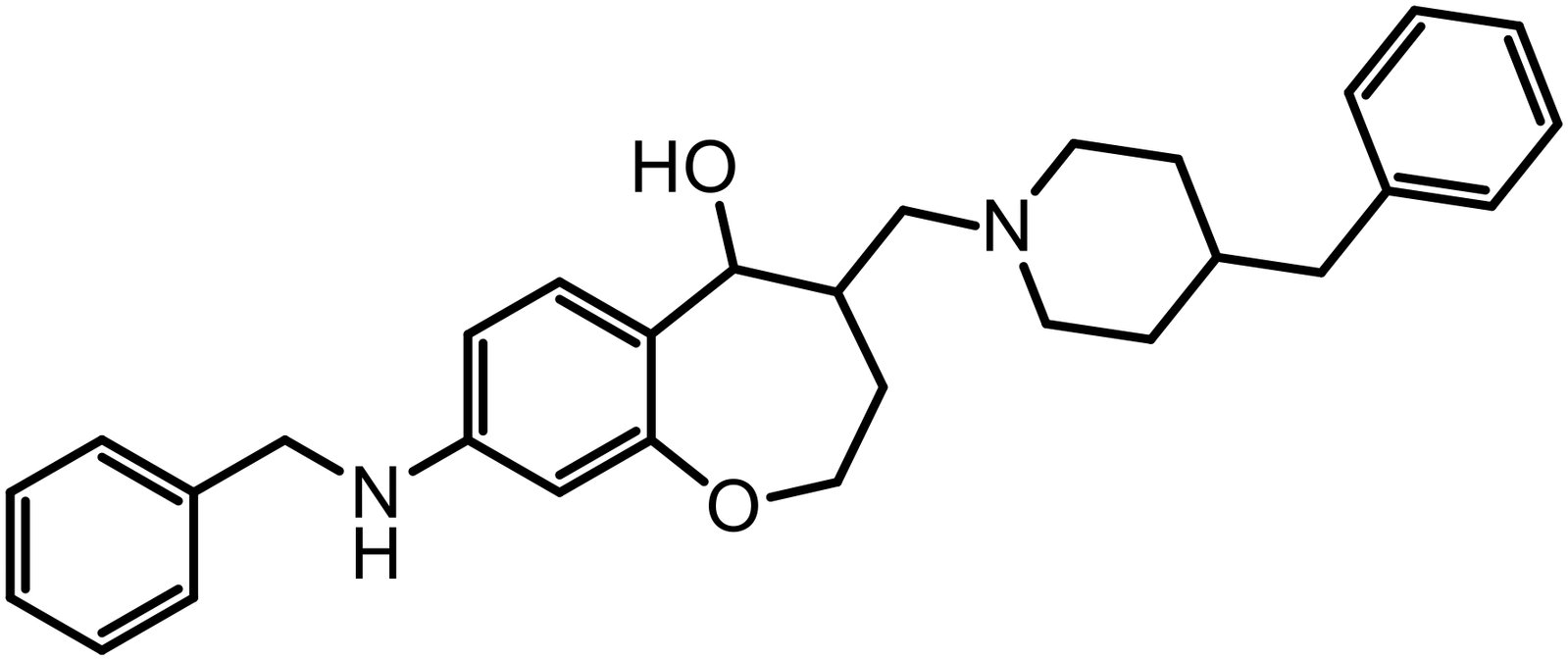
 According to *General procedure C*, a mixture of 10d (1.86 g, 3.84 mmol), LiAlH_4_ (1 M, 15 mL, 15 mmol) and THF_abs._ (100 mL) was heated to 80 °C for 6 h. After workup, the residue was purified by fc (*ø* 5 cm, *h* = 15 cm, cyclohexane : ethyl acetate + 0.1% *N*,*N*-dimethylethylamine 4 : 1 → 3 : 1→ 2.5 : 1, *R*_f_ = 0.67 (cyclohexane : ethyl acetate 1 : 1 + 0.1% *N*,*N*-dimethylethylamine)). Colorless resin, yield 0.926 g (53%). C_30_H_36_N_2_O_2_, *M*_r_ = 456.6. MS (EM): *m*/*z* calcd. for C_30_H_37_N_2_O_2_ 457.2855, found 457.2902. ^1^H NMR (CDCl_3_): *δ* [ppm] = 1.09–1.30 (m, 2H, 3-H, 5-H_piperidine_), 1.14–1.72 (m, 5H, 2-H (1H), 3-H, 4-H, 5-H_piperidine_ and 3-H), 1.77–1.90 (m, 1H, 6-H_piperidine_), 1.93–2.00 (m, 0.8H, CH_2_N), 2.05–2.17 (m, 0.8H, 3-H), 2.22–2.29 (m, 0.2H, CH_2_N·), 2.40–2.51 (m, 4.6H, C*H*_2_C_6_H_5_, 4-H, CH_2_N (0.8H) and 6-H_piperidine_ (0.8H)), 2.53–2.62 (m, 0.2H, 3-H·), 2.68 (t, *J* = 11.4 Hz, 0.2H, CH_2_N·), 2.86 (d, *J* = 13.0 Hz, 0.2H, 6-H_piperidine_·), 3.03–3.16 (m, 1H, 2-H_piperidine_), 3.42–3.58 (m, 0.8H, 2-H), 3.63–3.70 (m, 0.2H, 2-H·), 3.88 (s (broad), 1H, NH), 4.04 (dt, *J* = 12.3/4.0 Hz, 0.8H, 2-H), 4.16–4.23 (m, 0.2H, 2-H·), 4.24 (s, 2H, NHC*H*_*2*_C_6_H_5_), 4.73 (d, *J* = 9.5 Hz, 0.2H, 5-H·), 4.99 (s, 0.8H, 5-H), 6.22 (d, *J* = 2.4, 0.2H, 9-H·), 6.24 (d, *J* = 2.4 Hz, 0.8H, 9-H), 6.35–6.40 (m, 1H, 7-H), 7.06 (d, *J* = 6.9 Hz, 2H, 2-H, 6-H–C_6_H_5_), 7.09–7.16 (m, 1H, 4-H–C_6_H_5_), 7.16–7.33 (m, 7.8H, 3-H, 5-H–C_6_H_5_, C_6_H_5 Bn_ and (0.8H) 6-H), 7.48 (d, *J* = 9.0 Hz, 0.2H, 6-H·). A signal for OH protons is not seen in the spectrum. Ratio of *cis*-11d : *trans*-11d according to ^1^H NMR spectrum is 80 : 20. Signals of *trans*-11d are marked with ·. ^13^C NMR (CDCl_3_): *δ* [ppm] = 32.5, 32.6 (2C, C-3, C-5_piperidine_), 33.0 (0.8C, C-3), 34.4 (0.2C, C-3·), 37.1 (1C, C-4), 38.0 (1C, C-4_piperidine_), 43.3 (1C, CH-*C*H_2_C_6_H_5_), 48.8 (1C, NH*C*H_2_C_6_H_5_), 52.5, 53.3, 55.7 (2C, C-2, C-6_piperidine_), 59.9 (0.8C, CH_2_N), 65.3 (0.2C, CH_2_N·), 68.6 (0.8C, C-2), 71.8 (0.2C, C-2·), 74.5 (0.8C, C-5), 76.1 (0.2C, C-5·), 105.2 (0.2C, C-9·), 105.7 (0.8C, C-9), 108.6 (1C, C-7), 125.0 (1C, C-5a), 126.1 (1C, C-4-C6H5), 127.4 (0.2C, C-4_Bn_·), 127.5 (0.8C, C-4_Bn_), 127.8 (0.2C, C-6·), 127.9 (0.8C, C-6), 128.4, 128.6, 128.8, 129.3 (8C, C-2, C-3, C-5, C-6-C_6_H_5_ and C-2, C-3, C-5, C-6_Bn_), 139.5 (0.8C, 1-C_6_H_5_), 139.7 (0.2C, C-1-C_6_H_5_·), 140.8 (1C, C-1_Bn_), 148.4 (0.2C, C-8·), 148.6 (0.8C, C-8), 157.9 (0.8C, C-9a), 158.5 (0.2C, C-9a·). Ratio of diastereomers is 80 : 20. IR (neat): *ν̃* [cm^−1^] = 3352 (N–H), 3082 (C–H_arom._), 3059 (C–H_arom._), 3024 (C–H_arom._), 2916 (C–H), 2846 (C–H), 1616 (N–H). Purity (HPLC): 77.3%, *t*_R_ = 18.83 min and 20.2% *t*_R_ = 20.95 min.

#### *cis*-8-Amino-4-[(piperidin-1-yl)methyl]-2,3,4,5-tetrahydro-1-benzoxepin-5-ol (*cis*-12a) and *trans*-8-amino-4-[(piperidin-1-yl)methyl]-2,3,4,5-tetrahydro-1-benzoxepin-5-ol (*trans*-12a)


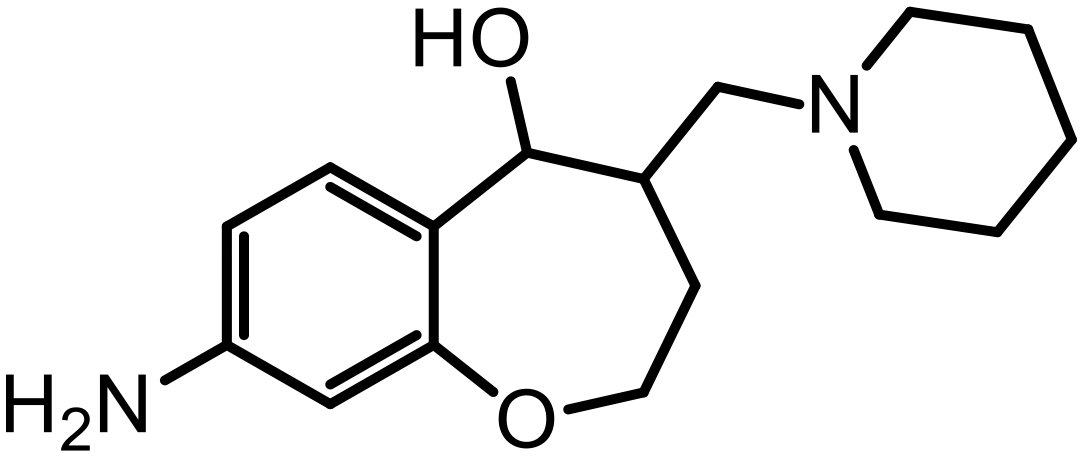
 Under N_2_, benzylamine 11a (0.387 g, 1.06 mmol) was dissolved in MeOH_abs._ (30 mL) and Pd/C (39 mg, 10% w/w) was added. The mixture was purged with H_2_ and stirred vigorously under H_2_ atmosphere (balloon) for 1 d at rt. Then, Pd/C was filtered off through Celite® and the solvent was removed under reduced pressure. The residue was purified by fc (*ø* 3 cm, *h* = 15 cm, cyclohexane : ethyl acetate 2 : 1 + 0.1% *N*,*N*-dimethylethylamine, *R*_f_ = 0.41). Pale yellow solid, mp >115 °C (decomposition), yield 132 mg (45%). C_16_H_24_N_2_O_2_, *M*_r_ = 276.4. MS (EM): *m*/*z* (%) calcd. for C_16_H_25_N_2_O_2_ 277.1837, found 277.1867. ^1^H NMR (CDCl_3_): *δ* [ppm] = 1.30–1.44 (m, 2H, 4-H_piperidine_), 1.45–1.60 (m, 5H, 3-H, 5-H_piperidine_ and 3-H), 1.63–1.75 (m, 0.37H, 3-H·), 1.76–1.90 (m, 0.37H, 4-H·), 1.93–2.00 (m, 0.63H, CH_2_N), 2.01–2.18 (m, 3.63H, 2-H, 6-H_piperidine_ and 3-H), 2.27 (dd, *J* = 12.7/2.7 Hz, 0.37H, CH_2_N·), 2.42 (t, *J* = 12.1 Hz, 0.63H, CH_2_N), 2.37–2.59 (m, 1.63H, 2-H_piperidine_, 4-H (0.63H)), 2.66 (dd, *J* = 12.5/11.6 Hz, 0.37H, CH_2_N·), 3.42–3.67 (m, 1H, 2-H), 4.06 (dt, *J* = 12.0/4.0 Hz, 0.63H, 2-H), 4.22 (dt, *J* = 12.2/3.9 Hz, 0.37H, 2-H·), 4.73 (d, *J* = 9.2 Hz, 0.37H, 5-H·), 5.00 (s, 0.63H, 5-H), 6.26 (d, *J* = 2.4 Hz, 0.37H, 9-H·), 6.27 (d, *J* = 2.3 Hz, 0.63H, 9-H), 6.41 (dd, *J* = 8.3/2.3 Hz, 0.63H, 7-H), 6.41 (dd, *J* = 8.3/2.2 Hz, 0.37H, 7-H·) 7.26 (dd, *J* = 8.2/0.8 Hz, 0.63H, 6-H), 7.46 (dd, *J* = 8.3/0.9 Hz, 0.37H, 6-H·). Signals for OH or NH protons are not seen in the spectrum. Ratio of *cis*-12a : *trans*-12a according to ^1^H NMR spectrum is 63 : 37. Signals of *trans*-12a are marked with ·. ^13^C NMR (CDCl_3_): *δ* [ppm] = 24.3 (1C, C-4_piperidine_), 26.1 (0.37C, C-3_piperidine_·), 26.4 (2 × 0.63C, C-3, C-5_piperidine_), 32.6 (0.63C, C-3), 34.4 (0.37C, C-3·), 36.8 (2C, C-2, C-6_piperidine_), 37.9 (0.37C, C-5_piperidine_·), 55.14 (1C, C-4), 60.24 (0.63C, CH_2_N), 65.7 (0.37C, CH_2_N·), 68.4 (0.63C, C-2), 71.8 (0.37C, C-2·), 74.5 (0.63C, C-5), 76.0 (0.37C, C-5·), 107.7 (0.37C, C-9·), 108.2 (0.63C, C-9), 110.8 (1C, C-7), 126.2 (1C, C-5a), 128.6 (1C, C-6), 146.3 (0.37C, C-8·), 146.5 (0.63C, C-8), 157.7, 158.4 (1C, C-9a). Ratio of diastereomers is 63 : 37. IR (neat): *ν̃* [cm^−1^] = 3449 (N–H), 3348 (N–H), 3209 (O–H), 2931 (C–H). Purity (HPLC): 89.1%, *t*_R_ = 2.86 min and 3.42 min.

#### *cis*-8-Amino-4-[(4-phenylpiperidin-1-yl)methyl]-2,3,4,5-tetrahydro-1-benzoxepin-5-ol (*cis*-12b) and *trans*-8-amino-4-[(4-phenylpiperidin-1yl)methyl]-2,3,4,5-tetrahydro-1-benzoxepin-5-ol (*trans*-12b)


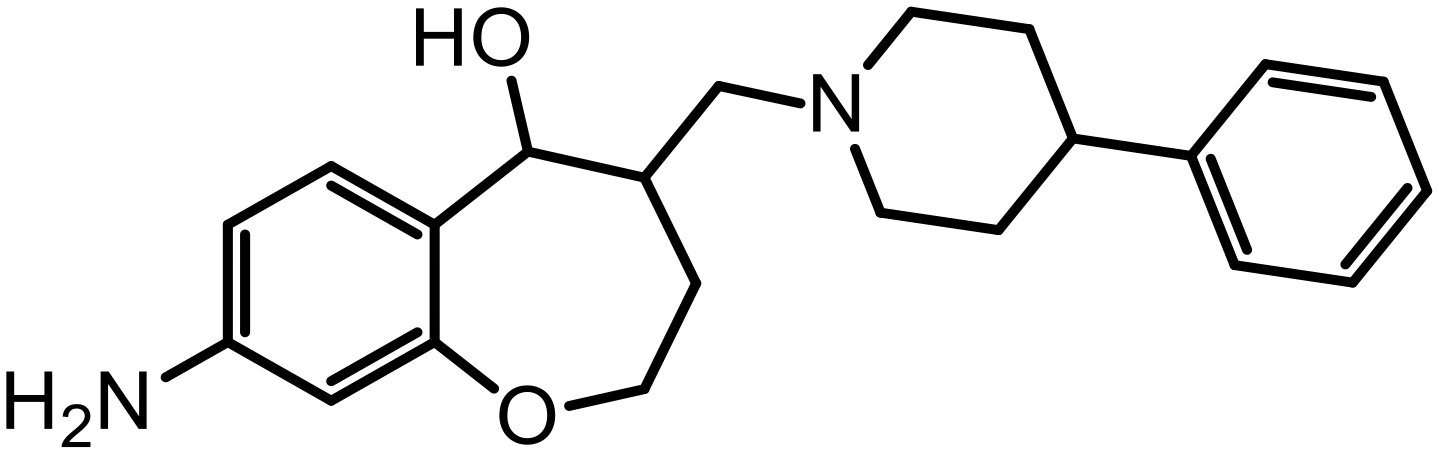
 As described for the synthesis of 12a, a mixture of 11b (0.601 g, 1.36 mmol), Pd/C (0.61 g, 10% w/w), and MeOH (50 mL) was stirred under H_2_ (balloon) for 2 d at rt. After filtration through Celite®, the solvent was removed under reduced pressure and the residue was purified by fc (*ø* 4 cm, *h* = 15 cm, cyclohexane : ethyl acetate 2 : 1 + 0.1% *N*,*N*-dimethylethylamine, *R*_f_ = 0.50 (cyclohexane : ethyl acetate 2 : 1 + 0.1% *N*,*N*-dimethylethylamine)). Pale yellow solid, mp 118–121 °C, yield 259 mg (47%). C_22_H_28_N_2_O_2_, *M*_r_ = 352.5. MS (EM): *m*/*z* calcd. for C_22_H_29_N_2_O_2_ 353.2229, found 353.2242. MS (EI): *m*/*z* (%) 352 (M, 8), 174 (CH_2_–NC_5_H_9_–C_6_H_5_, 100). ^1^H NMR (CDCl_3_): *δ* [ppm] = 1.43–1.91 (m, 7.55H, 2-H, 3-H, 5-H, 6-H_piperidine_, 3-H (1.55H)), 2.01–2.04 (m, 0.45H, 3-H·), 2.04–2.09 (m, 0.55H, 4-H_piperidine_), 2.34 (dd, *J* = 12.7/2.7 Hz, 0.55H, CH_2_N), 2.39–2.49 (m, 1H, 4-H_piperidine_· and 4-H), 2.49–2.57 (m, 2 × 0.45H, CH_2_N· and 4-H·), 2.60 (d, *J* = 10.9 Hz, 0.45H, 6-H_piperidine_·), 2.75 (t, *J* = 12.1 Hz, 0.55H, CH_2_N), 3.02 (d, *J* = 10.0 Hz, 0.55H, 6-H_piperidine_), 3.26 (d, *J* = 10.6 Hz, 1H, 2-H_piperidine_), 3.48–3.62 (m, 1.45H, CH_2_N, 2-H), 4.07 (dt, *J* = 12.6/4.1 Hz, 0.45H, 2-H·), 4.23 (dt, *J* = 12.1/3.9 Hz, 0.55H, 2-H), 4.77 (d, *J* = 9.3 Hz, 0.55H, 5-H), 5.02 (s, 0.45H, 5-H·), 6.27 (d, *J* = 2.4 Hz, 0.55H, 9-H), 6.29 (d, *J* = 2.3 Hz, 0.45H, 9-H·), 6.42 (dd, *J* = 10.0/2.0 Hz, 1H, 7-H), 7.01–7.34 (m, 5.45 H, C_6_H_5_ and 6-H·), 7.48 (d, *J* = 8.3 Hz, 0.55 Hz, 6-H). Signals for NH or OH protons are not seen in the spectrum. Ratio of *trans*-12b : *cis*-12b according to ^1^H NMR spectrum is 55 : 45. Signals of *cis*-12b are marked with ·. ^13^C NMR (CDCl_3_): *δ* [ppm] = 32.6 (0.55C, C-3), 33.2 (0.45C, C-3·), 33.5, 34.2 (2 × 0.55C, C-3, C-5_piperidine_), 33.9, 34.4 (2 × 0.45C, C-3, C-5_piperidine_·), 37.1 (0.55C, C-4), 38.1 (0.45C, C-4·), 42.6 (1C, C-4_piperidine_), 52.9 (0.45C, C-2_piperidine_·), 53.7 (0.55, C-2_piperidine_), 56.2 (0.55 C, C-6_piperidine_), 59.9 (0.55C, CH_2_N), 60.6, 65.5 (2 × 0.45C, CH_2_N· and C-6_piperidine_·), 68.5 (0.55C, C-2), 72.1 (0.45C, C-2·), 74.6 (0.55C, C-5), 76.1 (0.45C, C-5·), 107.8 (0.45C, C-9·), 108.2 (0.55C, C-9), 110.0 (0.45C, C-7·), 110.8 (0.55C, C-7), 126.1 (1C, C-4-C_6_H_5_), 126.5 (1C, C-6), 127.0 (2C, C-3, C-5-C_6_H_5_), 127.9 (1C, C-5a), 128.6 (2C, C-2, C-6-C_6_H_5_), 146.0 (0.45C, C-1-C_6_H_5_·), 146.1 (0.55C, C–C_6_H_5_), 146.5 (0.45C, C-8·), 146.6 (0.55C, C-8), 157.8 (0.55C, C-9a), 158.5 (0.45C, C-9a·). Ratio of diastereomers is 55 : 45. IR (neat): *ν̃* [cm^−1^] = 3646 (N–H, O–H), 3327 (N–H, O–H), 3026 (C=C), 2927 (C–H). Purity (HPLC): 94.7%, *t*_R_ = 11.97 min.

#### *cis*-8-Amino-4-[(4-phenylpiperazin-1yl)methyl]-2,3,4,5-tetrahydro-1-benzoxepin-5-ol (*cis*-12c) and *trans*-8-amino-4-[(4-phenylpiperazin-1yl)methyl]-2,3,4,5-tetrahydro-1-benzoxepin-5-ol (*trans*-12c)


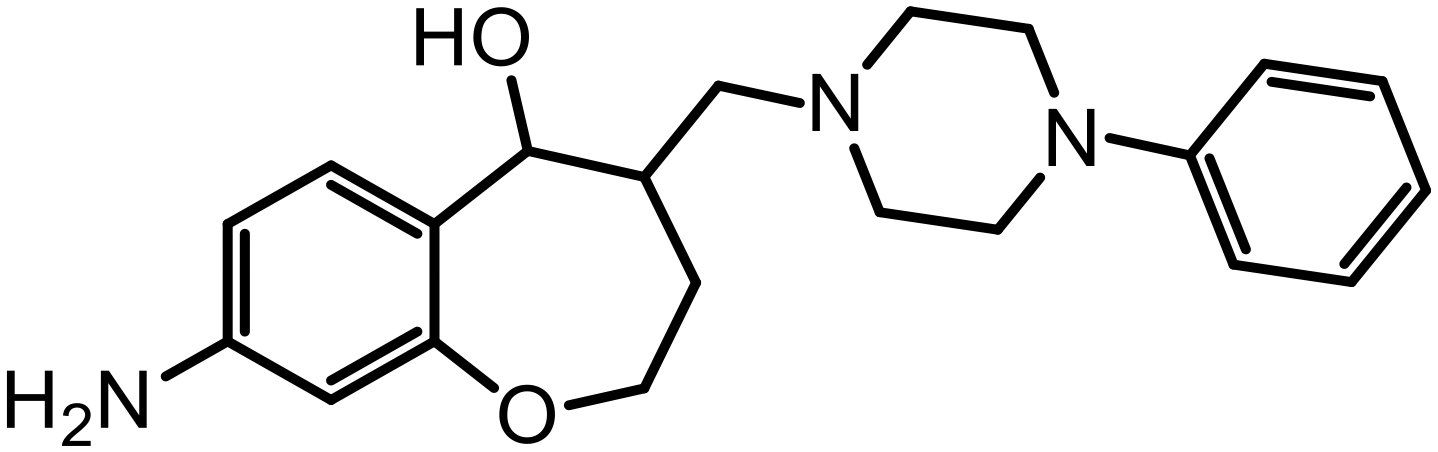
 Under N_2_, a mixture of 11c (0.60 g, 1.35 mmol), ammonium formate (0.102 g, 1.62 mmol), Pd/C (10% w/w, 0.06 g) and MeOH_abs._ (25 mL) was heated to 60 °C for 4 h. After filtration through Celite®, the solvent was removed under reduced pressure and the residue was purified by fc (*ø* 4 cm, *h* = 15 cm, cyclohexane : ethyl acetate 2 : 1 + 0.1% *N*,*N*-dimethylethylamine, *R*_f_ = 0.08). Pale yellow resin, yield 169 mg (36%). C_21_H_27_N_3_O_2_, *M*_r_ = 353.5. MS (EM): *m*/*z* calcd. for C_21_H_28_N_3_O_2_ 354.2182 found 354.2232. ^1^H NMR (CDCl_3_): *δ* [ppm] = 1.41–1.91 (m, 4H, CH_2 piperazine_), 1.86–1.92 (m, 0.45H, 3-H·), 2.04–2.30 (m, 0.45 CH_2_N·), 2.43 (d, *J* = 12.7 Hz, 1H, CH_2_N), 2.48–2.59 (m, 0.55H, CH_2_N), 2.63–3.04 (m, 2.55H, 3-H and 4-H), 3.08–3.37 (m, 4H, CH_2 piperazine_), 3.61 (t, *J* = 10.1 Hz, 1H, 2-H), 3.98–4.11 (m, 0.55H, 2-H), 4.15–4.29 (m, 0.45H, 2-H·), 4.76 (d, *J* = 9.2 Hz, 0.55H, 5-H), 5.02 (s, 0.45H, 5-H·), 6.21–6.36 (m, 1H, 7-H), 6.31–6.46 (m, 1H, 9-H), 6.78–6.96 (m, 3H, 2-H, 4-H, 6-H_Ph_), 7.15–7.25 (m, 2.55H, 3-H, 5-H_Ph_ and 6-H), 7.41 (d, *J* = 8.3 Hz, 0.45H, 6-H·). Signals for NH and OH protons are not seen in the spectrum. Ratio of *trans*-12c : *cis*-12c according to ^1^H NMR spectrum is 55 : 45. Signals of *cis*-12c are marked with ·. ^13^C NMR (CDCl_3_): *δ* [ppm] = 32.4 (0.55C, C-3), 34.1 (0.45C, C-3·), 37.2 (0.55C, C-4), 37.9 (0.45C, C-4·), 49.4 (2 × 0.45C, C_piperazine_·), 49.6 (1.1C, C_piperazine_), 53.4 (2C, C_piperazine_), 59.8 (0.55C, CH_2_N), 64.7 (0.45C, CH_2_N·), 68.8 (0.45C, C-2·), 71.6 (0.55C, C-2), 74.6 (0.55C, C-5), 76.1 (0.45C, C-5·), 107.8 (0.45C, C-9·), 108.3 (0.55C, C-9), 110.0 (0.45C, C-7·), 110.8 (0.55C, C-7), 116.5 (2C, C-2, C-6_Ph_), 120.3 (1C, C-4_Ph_), 125.9, 127.5 (1C, C-5a), 128.0, 128.7 (1C, C-6), 129.4 (2C, C-3, C-5_Ph_), 146.8 (1C, C-8), 151.6 (1C, C-1_Ph_), 157.9, 158.6 (1C, C-9a). Ratio of diastereomers is 55 : 45. IR (neat): *ν̃* [cm^−1^] = 3707 (N–H), 2920 (C–H), 2866 (C–H). Purity (HPLC): 93.1%, *t*_R_ = 7.93 min and 8.82 min.

#### *cis*-8-Amino-4-[(4-benzylpiperidin-1-yl)methyl]-2,3,4,5-tetrahydro-1-benzoxepin-5-ol (*cis*-12d) and *trans*-8-amino-4-[(benzylpiperidin-1-yl)methyl]-2,3,4,5-tetrahydro-1-benzoxepin-5-ol (*trans*-12d)


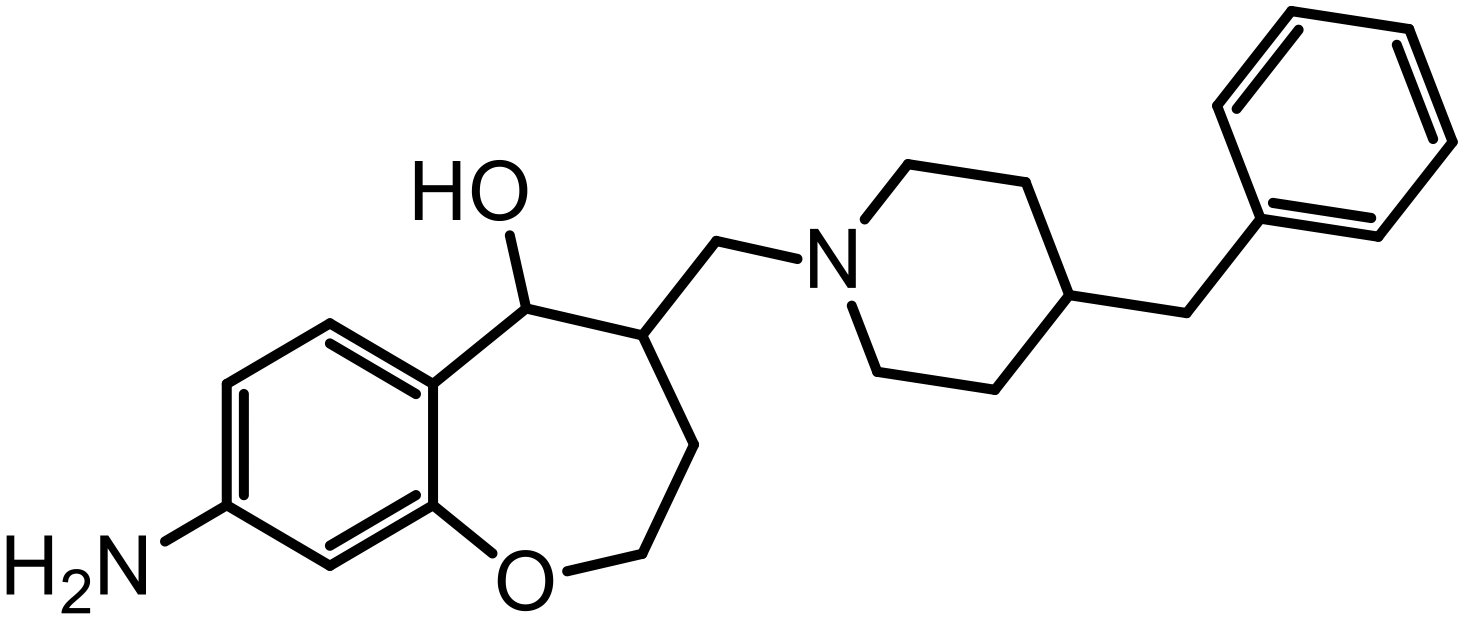
 A mixture of 11d (0.566 g, 1.24 mmol), Pd/C (0.06 g, 10% w/w), ammonium formate (0.28 g, 4.44 mmol) and MeOH was heated to reflux for 9 h. After filtration through Celite®, the solvent was removed under reduced pressure. The residue was purified by fc (*ø* 4 cm, *h* = 15 cm, cyclohexane : ethyl acetate + 0.1% *N*,*N*-dimethylethylamine 4 : 1 → 3 : 1 *R*_f_ = 0.40 (cyclohexane : ethyl acetate 1 : 1 + 0.1% *N*,*N*-dimethylethylamine)).

Yellow resin, yield 96 mg (21%). C_23_H_30_N_2_O_2_, *M*_r_ = 366.5. MS (EM): calcd. for C_23_H_31_N_2_O_2_ 367.2386, found 367.2365. ^1^H NMR (CDCl_3_): *δ* [ppm] = 1.30–1.90 (m, 9H, 2-H, 3-H, 4-H, 5-H, 6-H_piperidine_ and 3-H), 2.07–2.20 (m, 1H, 4-H), 2.25 (dd, *J* = 12.7/2.6 Hz, 0.5H, CH_2_N), 2.37–2.60 (m, 2.5H, C*H*_2_C_6_H_5_ and 6-H_piperidine_), 2.68 (t, *J* = 11.0 Hz, 0.5H, CH_2_N), 2.86 (d, *J* = 11.1 Hz, 0.5H, 6-H_piperidine_), 3.10 (d, *J* = 12.1 Hz, 1H, 2-H_piperidine_), 3.41–3.62 (m, 1.5H, CH_2_N and 2-H), 3.79–3.93 (m, 0.5H, 2-H), 4.01–4.09 (m, 0.5H, 2-H), 4.21 (dt, *J* = 12.1/3.8 Hz, 0.5H, 2-H), 4.73 (d, *J* = 9.2 Hz, 0.5H, 5-H), 5.00 (s, 0.5H, 5-H), 6.26 (d, *J* = 2.4 Hz, 0.5H, 9H), 6.27 (d, *J* = 2.3 Hz, 0.5 H, 9-H), 6.41 (dd, *J* = 8.2/2.5 Hz, 1H, 7-H), 7.06 (d, *J* = 7.4 Hz, 2H, 2-H, 6-H–C_6_H_5_), 7.09–7.16 (m, 1H, 4-H–C_6_H_5_), 7.18–7.31 (2.5H, 3-H, 5-H–C_6_H_5_ and 6-H), 7.46 (dd, *J* = 8.3/0.8 Hz, 0.5H, 6-H). Signals for OH and NH protons are not be seen in the spectrum. Ratio of *cis*-12d : *trans*-12d according to ^1^H NMR spectrum is 50 : 50. IR (neat): *ν̃* [cm^−1^] = 3352 (N–H), 2920 (C–H), 2851 (C–H), 2808 (C–H), 1616 (N–H). Purity (HPLC): 87.2%, *t*_R_ = 13.03 min and 13.57 min.

#### *cis*-8-Amino-4-[(4-benzylpiperidin-1-yl)methyl]-2,3,4,5-tetrahydro-1-benzoxepin-5-ol (*cis*-12d)

As described for the synthesis of 12a, a mixture of 11d (0.250 g, 0.55 mmol), Pd/C (0.025 g, 10% w/w) and MeOH was stirred under H_2_ atmosphere (balloon) at rt for 24 h. After filtration through Celite®, the solvent was removed under reduced pressure. The residue was purified by fc (*ø* 3 cm, *h* = 20 cm, *R*_f_ = 0.40 (cyclohexane : ethyl acetate 1 : 1 + 0.1% *N*,*N*-dimethylethylamine)). A diastereomerically pure product (*cis*-12d) could be isolated through purification by fc.

##### *cis*-12d

Yellow resin, yield 92 mg (46%). C_23_H_30_N_2_O_2_, *M*_r_ = 366.5. ^1^H NMR (CDCl_3_): *δ* [ppm] = 1.13–1.85 (m, 8H, 3-H, 4-H, 5-H, 6-H_piperidine_ and 3-H), 1.96 (d, *J* = 11.6 H, 1H, 2-H_piperidine_), 2.04–2.22 (m, 1H, 4-H), 2.14–2.53 (m, 3H, 6-H_piperidine_ and CH_2_C_6_H_5_), 3.10 (d, *J* = 11.5 Hz, 1H, 2-H_piperidine_), 3.37–3.67 (m, 3H, 2-H and CH_2_N), 4.05 (dt, *J* = 12.3/4.0 Hz, 1H, 2-H), 5.00 (s, 1H, 5-H), 6.27 (d, *J* = 2.3 Hz, 1H, 9-H), 6.40 (dd, *J* = 8.2/2.3 Hz, 1H, 7-H), 7.04–7.16 (m, 3H, 2-H, 4-H, 6-H–C_6_H_5_), 7.17–7.29 (m, 3H, 3-H, 5-H–C_6_H_5_ and 6-H). Signals for NH and OH are not seen in the spectrum. ^13^C NMR (CDCl_3_): *δ* [ppm] = 32.4, 32.6 (2C, C-3, C-5_piperidine_), 33.0 (1C, C-3), 37.0 (1C, C-4), 38.0 (1C, C-4_piperidine_), 43.3 (1C, *C*H_2_C_6_H_5_) 53.3 (1C, C-2 or C-6_piperidine_), 55.7 (1C, C-2 or C-6_piperidine_), 59.8 (1C, CH_2_N), 68.4 (1C, C-2), 74.5 (1C, C-5), 108.2 (1C, C-9), 110.8 (1C, C-7), 126.1 (1C, C-6), 126.1 (1C, C-5a), 128.4 (2C, C-2, C-6-C_6_H_5_), 128.5 (1C, C-4-C_6_H_5_), 129.3 (2C, C-3, C-5-C_6_H_5_), 140.8 (1C, C-1-C_6_H_5_), 146.6 (1C, C-8), 157.8 (1C, C-9a). Purity (HPLC): 96.3%, *t*_R_ = 12.62 min.

#### *N*-{*cis*-5-Hydroxy-4-[(4-phenylpiperidin-1-yl)methyl]-2,3,4,5-tetrahydro-1-benzoxepin-8-yl}formamide (*cis*-13b) and *N*-{*trans*-5-hydroxy-4-[(4-phenylpiperidin-1-yl)methyl]-2,3,4,5-tetrahydro-1-benzoxepin-8-yl}formamide (*trans*-13b)


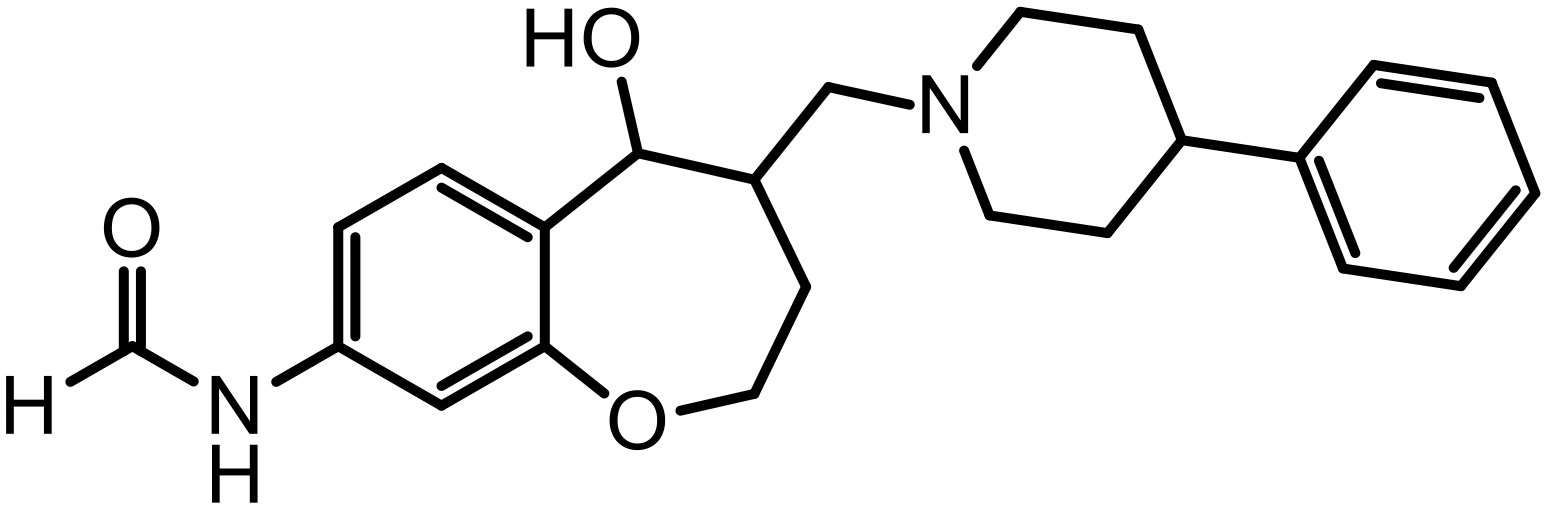
 Formic acid (40 mg, 0.85 mmol) was added to Ac_2_O (72 mg, 0.71 mmol) at 0 °C. The mixture was heated to 55 °C for 2 h. After cooling to rt, a solution of primary amine 12b (100 mg, 0.28 mmol) and THF_abs._ (10 mL) was added and the mixture was stirred for 2 h at 40 °C. For workup, saturated aqueous NaHCO_3_ solution (10 mL) was added and the mixture was extracted with CH_2_Cl_2_. The combined organic layers were washed with brine, dried (Na_2_SO_4_), filtered and the solvent was removed under reduced pressure. The residue was purified by fc two times (*ø* 3 cm, *h* = 15 cm, cyclohexane : ethyl acetate 1 : 1 + 0.1% *N*,*N*-dimethylethylamine, *ø* 3 cm, *h* = 15 cm, cyclohexane : ethyl acetate 1.25 : 1 + 0.1% *N*,*N*-dimethylethylamine, *R*_f_ = 0.20 (cyclohexane : ethyl acetate 1 : 1 + 0.1% *N*,*N*-dimethylethylamine)). Yellow resin, yield 37 mg (37%). C_23_H_28_N_2_O_3_, *M*_r_ = 380.5. MS (EM): *m*/*z* calcd. for C_23_H_29_N_2_O_3_ 381.2178, found 381.2218. ^1^H NMR (CDCl_3_): *δ* [ppm] = 1.44–1.66 (m, 1H, 3-H), 1.63–1.97 (m, 5H, 2-H, 3-H, 5-H_piperidine_), 2.02–2.12 (m, 1.6H, CH_2_N, 6-H_piperidine_), 2.14–2.30 (m, 1.2H, 3-H and 6-H_piperidine_·), 2.38–2.53 (2H, CH_2_N and 4-H_piperidine_), 2.53–2.71 (m, 1.8H, 4-H and 6-H_piperidine_), 2.80 (ddd, *J* = 13.1/12.3/3.4 Hz, 0.2H, CH_2_N·), 3.03 (d, *J* = 10.8 Hz, 0.2H, 6-H_piperidine_·), 3.29 (d, *J* = 10.8 Hz, 1H, 2-H_piperidine_), 3.56 (ddd, *J* = 13.4/7.8/3.3 Hz, 1H, 2-H), 4.10–4.21 (m, 0.8H, 2-H), 4.29 (dd, *J* = 12.5/9.1 Hz, 0.2H, 2-H·), 4.38 (d, *J* = 9.4 Hz, 0.2H, 5-H·), 5.10 (s, 0.8H, 5-H), 6.65 (dd, *J* = 7.4/2.3 Hz, 0.8H, 7-H), 6.79 (dt, *J* = 8.2/3.1 Hz, 0.2H, 7-H·), 7.06–7.32 (m, 6H, C_6_H_5_ and 9-H), 7.51 (dd, *J* = 13.4/8.0 Hz, 0.8H, 6-H), 7.64–7.77 (m, 0.2H, 6-H·), 8.29 (d, *J* = 1.8Hz, 0.5H, O=CH), 8.61 (2 × d, *J* = 11.4, 0.5H, O=CH). A signal for NH and OH protons is not seen in the spectrum. Ratio of *cis*-13b : *trans*-13b according to ^1^H NMR spectrum is 80 : 20. Signals of *trans*-13b are marked with ·. Ratio of rotamers is 50 : 50. ^13^C NMR (CDCl_3_): *δ* [ppm] = 32.3, 32.5 (1C, C-3), 33.5, 34.2 (2C, C-3, C-5_piperidine_), 36.50, 36.7 (1C, C-4), 42.2 (0.2C, C-4_piperidine_·), 42.5 (0.8C, C-4_piperidine_), 52.8 (0.2C, C-2_piperidine_·), 53.6 (0.8C, C-2_piperidine_), 56.2 (1C, C-6_piperidine_), 59.8 (1C, CH_2_N), 68.5 (1C, C-2), 74.4 (1C, C-5), 111.8, 113.2, 114.0, 115.3 (2C, C-7, C-9), 126.6 (1C, C-4-C_6_H_5_), 126.0 (2C, C-3, C-5-C_6_H_5_), 128.2, 129.1 (1C, C-6), 128.7 (2C, C-2, C-6-C_6_H_5_), 132.8, 133.1 (1C, C-5a), 136.6, 136.8 (1C, C-8), 146.8 (1C, C-_6_H_5_), 157.1, 157.7 (1C, C-9a), 159.0, 162.3 (1C, C=O). Ratio of diastereomers is 80 : 20. IR (neat): *ν̃* [cm^−1^] = 3255 (N–H, OH), 3182 (N–H, OH), 3059 (CH_ar_), 2927 (C–H), 2854 (C–H), 1681 (C=O). Purity (HPLC): 22.4%, *t*_R_ = 14.58 min and 71.1%, *t*_R_ = 15.12 min.

#### *N*-{*cis*-[(4-Benzylpiperidin-1-yl)methyl]-5-hydroxy-2,3,4,5-tetrahydro-1-benzoxepin-8-yl}formamide (*cis*-13d) and *N*-{*trans*-[(4-benzylpiperidin-1-yl)methyl]-5-hydroxy-2,3,4,5-tetrahydro-1-benzoxepin-8-yl}formamide (*trans*-13d)


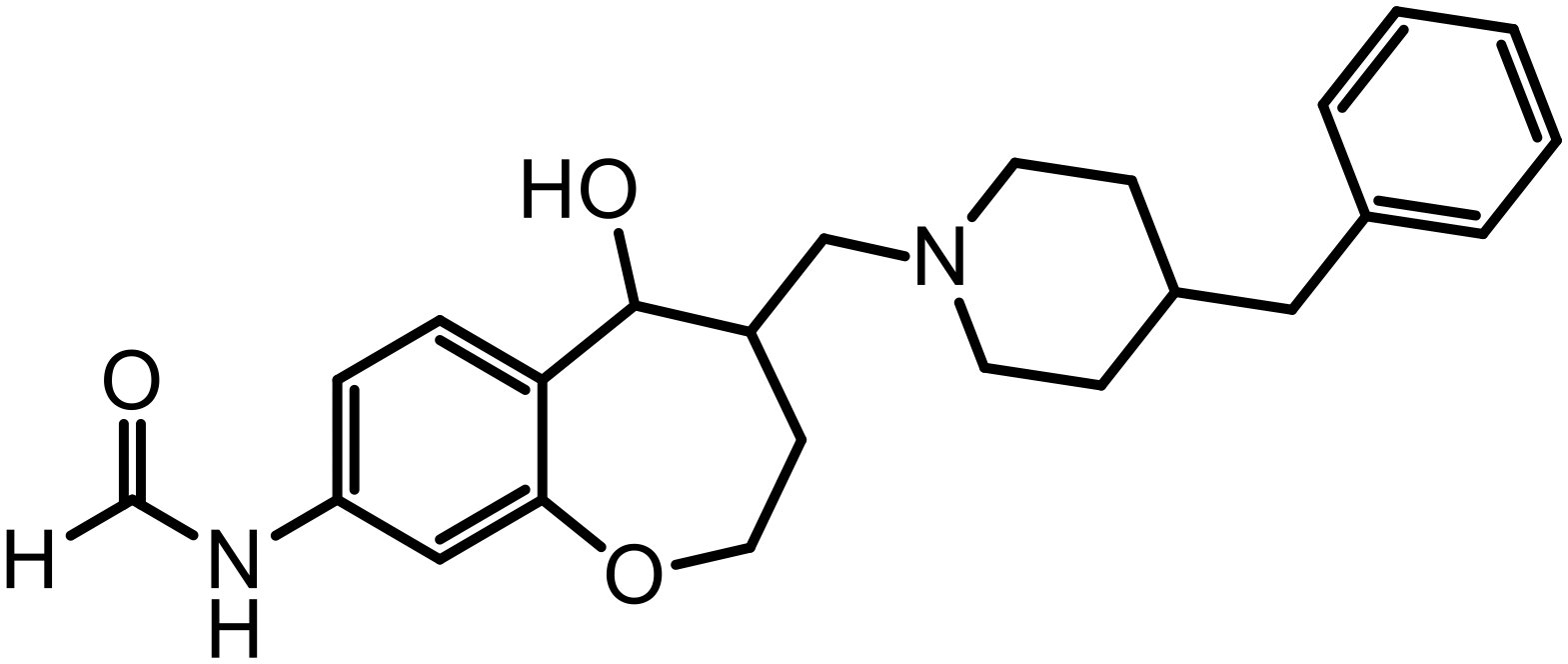
 As described for the synthesis of 13b, a mixture of Ac_2_O (0.06 g, 0.61 mmol) and formic acid (0.03 g, 0.74 mmol) was heated to 55 °C for 1 h. After cooling to rt a solution of primary amine 12d (90 mg, 0.25 mmol) and THF_abs._ (15 mL) was added and the mixture was heated for 2 h at 40 °C. For workup, saturated aqueous NaHCO_3_ solution (15 mL) was added and the mixture was extracted with CH_2_Cl_2_. The combined organic layers were washed with brine, dried (Na_2_SO_4_), filtered and the solvent was removed under reduced pressure. The residue was purified by fc two times (*ø* 3 cm, *h* = 15 cm, cyclohexane : ethyl acetate 3 : 1 + 0.1% *N*,*N*-dimethylethylamine, *ø* 2 cm, *h* = 15 cm, petrol ether : ethyl acetate 3 : 1 + 0.1% *N*,*N*-dimethylethylamine, *R*f = 0.18 (cyclohexane : ethyl acetate 1 : 1 + 0.1% *N*,*N*-dimethylethylamine)). Yellow resin, yield 22 mg (23%). C_24_H_30_N_2_O_3_, *M*_r_ = 394.2. MS (EM): *m*/*z* calcd. for C_24_H_31_N_2_O_3_ 395.2335, found 395.2333. ^1^H NMR (CDCl_3_): *δ* [ppm] = 0.58–2.11 (m, 7.6H, 2-H, 3-H, 4-H, 5-H-6-H_piperidine_ and 3-H), 2.12–2.37 (m, 0.4H, 2-H or 6-H_piperidine_· and 3-H·), 2.39–2.54 (m, 3H, 4-H and C*H*_2_C_6_H_5_), 2.63–2.78 (m, 1H, 2-H or 6-H_piperidine_), 2.82–3.00 (m, 2H, CH_2_N), 3.02–3.14 (m, 1H, 2-H or 6-H_piperidine_), 3.20–3.39 (m, 1H, 2-H_piperidine_), 3.46–3.58 (m, 1H, 2-H), 4.16–4.36 (m, 1H, 2-H), 4.80 (2 × d, *J* = 9.0 Hz, 0.8H, 5-H), 5.06 (s, 0.1H, 5-H·), 5.27 (s, 0.1H, 5-H·), 5.84 (s (broad), 0.2H, NH·), 5.96 (s, 0.8H, NH), 6.68–6.56 (m, 0.8H, 7-H), 6.76 (d, *J* = 8.6 Hz, 0.2H, 7-H·), 6.94 (d, *J* = 8.2 Hz, 1H, 9-H), 6.99–7.48 (m, 5H, C_6_H_5_), 7.53 (d, *J* = 7.8 Hz, 0.2H, 6-H·), 7.68 (2 × d, *J* = 8.3 Hz, 0.8H, 6-H), 8.31 (1H, OH), 8.58 (d, *J* = 11.4 Hz, 0.5H, CH=O), 8.60 (d, *J* = 4.1 Hz, 0.5H, CH=O). Ratio of *trans*-13d : *cis*-13d = 80 : 20. Signals of *cis*-13d are marked with ·. IR (neat): *ν̃* [cm^−1^] = 2920 (C–H), 2851 (C–H), 1682 (C=O). Purity (HPLC): 90.7%, *t*_R_ = 15.87 min and 15.87 min.

#### *N*-{*cis*-5-Hydroxy-4-[(piperidin-1yl)methyl]-2,3,4,5-tetrahydro-1-benzoxepin-8-yl}acetamide (*cis*-14a) and *N*-{*trans*-5-hydroxy-4-[(piperidin-1yl)methyl]-2,3,4,5-tetrahydro-1-benzoxepin-8-yl}acetamide (*trans*-14a)


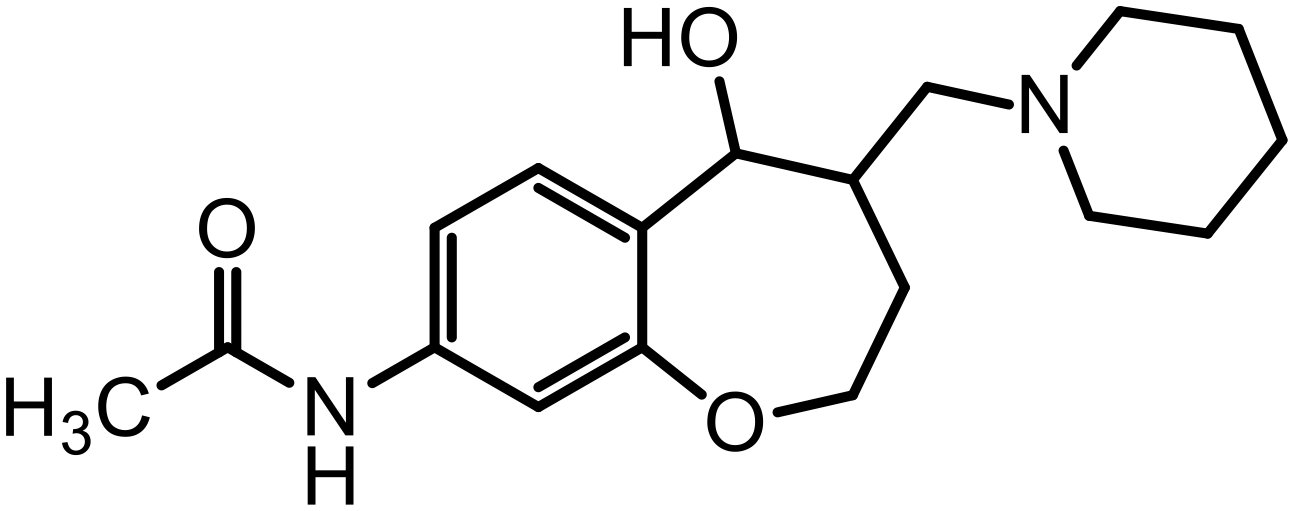
 Primary amine 12a (105 mg, 0.38 mmol) was dissolved in CH_2_Cl_2_ (10 mL). Ac_2_O (60 mg, 0.59 mmol) was added and the mixture was stirred at rt for 3 h. The solvent was removed under reduced pressure. MeOH (10 mL) and K_2_CO_3_ (0.1 g, 13.1 mmol) were added and the mixture was stirred for additional 2 h. Then, water (20 mL) and CH_2_Cl_2_ (20 mL) were added, the layers were separated and the aqueous layer was extracted with CH_2_Cl_2_. The combined organic layers were dried (Na_2_SO_4_), filtered and the solvent was removed under reduced pressure. The residue was purified by fc (*ø* 3 cm, *h* = 15 cm, cyclohexane : ethyl acetate 2 : 1 + 0.1% *N*,*N*-dimethylethylamine → 1 : 1 + 0.1% *N*,*N*-dimethylethylamine, *R*_f_ = 0.48 and 0.40). Yellow solid, mp decomposition >120 °C, yield 0.071 g (59%). C_18_H_26_N_2_O_3_, *M*_r_ = 318.4. MS (EM): calcd. for C_18_H_27_N_2_O_3_ 318.2022, found 319.1977. ^1^H NMR (CDCl_3_): *δ* [ppm] = 1.10–1.45 (m, 2H, 4-H_piperidine_), 1.46–1.62 (m, 4H, 3-H, 5-H_piperidine_), 1.63–1.88 (m, 3H, 3-H, 4-H), 1.94–2.20 (m, 1.6H, 2-H and 6-H_piperidine_), 2.07 (2 × s, 3H, CH_3_), 2.29 (dd, *J* = 12.7/2.6 Hz, 2 × 0.4H, CH_2_N·), 2.32–2.41 (m, 0.6H, CH_2_N), 2.46–2.60 (m, 2H, 2-H, 6-H_piperidine_), 2.64–2.73 (m, 0.6H, CH_2_N), 3.43–3.56 (m, 1H, 2-H), 3.81–3.90 (m, 0.4H, 2-H_piperidine_·), 4.09 (dt, *J* = 12.3/3.9 Hz, 0.4H, 2-H·), 4.25 (dt, *J* = 12.1/3.6 Hz, 0.6H, 2-H), 4.79 (d, *J* = 9.3 Hz, 0.6H, 5-H), 5.06 (s, 0.4H, 5-H·), 7.07 (dd, *J* = 8.4/2.1 Hz, 0.6H, 7-H·), 7.13–7.24 (m, 1.4H, 7-H and 9-H), 7.42 (d, *J* = 8.0 Hz, 0.4H, 6-H·), 7.52 (s, 0.6H, NH·), 7.58 (s, 0.4H, NH·), 7.61 (d, *J* = 8.4 Hz, 0.6H, 6-H). A signal for the OH proton is not seen in the spectrum. Ratio of *trans*-14a : *cis*-14a according to ^1^H NMR spectrum is 60 : 40. Signals of *cis*-14a are marked with ·. IR (neat): *ν̃* [cm^−1^] = 3302 (N–H), 3263 (N–H), 3109 (C–H_arom._), 2924 (C–H), 1666 (C=O). Purity (HPLC): 95.1%, *t*_R_ = 8.81 and 10.21 min.

#### *N*-{*cis*-5-Hydroxy-4-[(4-phenylpiperdin-1-yl)methyl]-2,3,4,5-tetrahdro-1-benzoxepin-8-yl}acetamide (*cis*-14b) and *N*-{*trans*-5-hydroxy-4-[(4-phenylpiperdin-1-yl)methyl]-2,3,4,5-tetrahdro-1-benzoxepin-8-yl}acetamide (*trans*-14b)


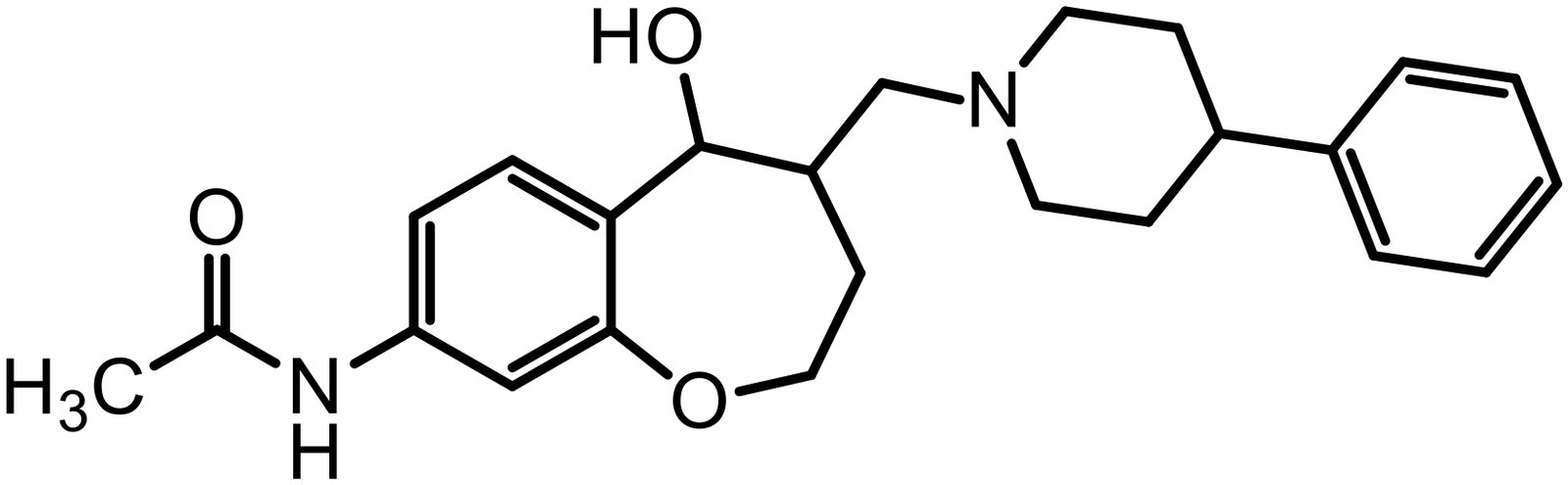
 Ac_2_O (45 mg, 0.44 mmol) was added to a solution of primary amine 12b (103 mg, 0.29 mmol) in CH_2_Cl_2_ (15 mL). The mixture was stirred at rt for 1 h. Then, saturated aqueous NaHCO_3_ solution was added (15 mL) and it was stirred for 10 min. The layers were separated and the aqueous layer was extracted with CH_2_Cl_2_. The combined organic layers were washed with brine, dried (Na_2_SO_4_), filtered and the solvent was removed under reduced pressure. The residue was purified by fc (*ø* 3 cm, *h* = 15 cm, *n*-hexane : ethyl acetate 2 : 1 + 0.1% *N*,*N*-dimethylethylamine, *R*_f_ = 0.27 (cyclohexane : ethyl acetate 2 : 1 + 0.1% *N*,*N*-dimethylethylamine)).

Intermediate {8-acetamido-4-[(4-phenylpiperidin-1-yl)methyl]-2,3,4,5-tetrahydro-1-benzoxepin-5-yl}acetate was isolated as pale-yellow resin, yield 79 mg (60%). C_26_H_32_N_2_O_4_, *M*_r_ = 436.5. MS (EI): *m*/*z* (%) 436 (M, 7), 393 (M–H_3_CCO, 9). ^1^H NMR (CDCl_3_): *δ* [ppm] = 1.52–1.78 (m, 6H, 3-H, 5-H_piperidine_ and 3-H), 1.90–2.04 (m, 2H, CH_2_N), 1.98 (s, 3 × 0.7H, CH_3_CO_2_), 2.00 (s, 3 × 0.3H, CH_3_CO_2_·), 2.09 (s, 3H, CH_3_CON), 2.14 (dd, *J* = 13.7/6.0 Hz, 2H, 2-H, 6-H_piperidine_), 2.20–2.30 (m, 1H, 4-H_piperidine_), 2.35–2.45 (m, 1H, 4-H), 2.67 (d, *J* = 10.9 Hz, 0.3H, 2-H or 6-H_piperidine_·), 2.81–2.87 (m, 2 × 0.7H, 2-H, 6-H_piperidine_), 2.92 (d, *J* = 11.0 Hz, 0.3H, 2-H or 6-H_piperidine_·), 3.86–3.93 (m, 0.3H, 2-H·), 3.96 (dd, *J* = 12.0/5.5 Hz, 0.7H, 2-H), 4.18–4.22 (m, 0.3H, 2-H·), 4.26 (dt, *J* = 14.8/4.4 Hz, 0.7H, 2-H), 5.86 (d, *J* = 5.1 Hz, 0.3H, 5-H·), 6.02 (s, 0.7H, 5-H), 6.96–7.26 (7.3H, C_6_H_5_, 6-H·, 7-H and 9-H), 7.29 (d, *J* = 8.3 Hz, 0.7H, 6-H). A signal for NH proton is not seen in the spectrum. Ratio of *cis*-acetate : *trans*-acetate according to ^1^H NMR spectrum is 70 30. Signals of *trans*-acetate are marked with ·. ^13^C NMR (CDCl_3_): *δ* [ppm] = 21.5 (0.7C, *C*H_3_CO_2_), 21.7 (0.3C, *C*H_3_CO_2_·), 24.9 (1C, *C*H_3_CON), 29.6 (1C, C-3), 31.1, 33.8 (1.7C, C-3, C-5_piperidine_), 35.5 (0.3C C-3 or C-5_piperidine_·), 38.1 (1C, C-4), 43.0 (1C, C-4_piperidine_), 94.9, 55.2 (2C, C-2, C-6_piperidine_), 59.3 (0.3C, CH_2_N·), 61.2 (0.7C, CH_2_N), 69.3 (0.3C, C-2·), 72.3 (0.7C, C-2), 75.5 (1C, C-5), 112.8 (0.7C, C-9), 113.1 (0.3C, C-9·), 114.1 (0.7C, C-7), 114.3 (0.3C, C-7·), 122.2 (1C, C-5a), 126.3 (1C, C-4_Ph_), 129.1 (2C, C-3, C-5_Ph_), 128.6 (2C, C-2, C-6_Ph_), 132.9 (0.7C, C-6), 133.2 (0.3C, C-6·), 138.9 (1C, C-8), 146.7 (1C, C-1_Ph_), 159.6 (0.3C, C-9a·), 160.7 (0.7C, C-9a), 168.4, 170.7 (2C, H3C*C*O_2_, H_3_CCON). Ratio of diastereomers is 70 : 30. Purity (HPLC): 97.8%, *t*_R_ = 16.72 min.

A mixture of intermediate {8-acetamido-4-[(4-phenylpiperidin-1-yl)methyl]-2,3,4,5-tetrahydro-1-benzoxepin-5-yl}acetate (79 mg, 0.18 mmol), K_2_CO_3_ (100 mg, 0.71 mmol) and CH_3_OH (20 mL) was stirred at rt for 1 h. Then, saturated aqueous NaHCO_3_ (20 mL) solution was added and the mixture was extracted with CH_2_Cl_2_. The combined organic layers were dried (Na_2_SO_4_), filtered and the solvent was removed under reduced pressure. The purity was so high that a fc purification was not required. *R*_f_ = 0.25 (cyclohexane : ethyl acetate 2 : 1 + 0.1% *N*,*N*-dimethylethylamine). Colorless solid, mp 118 °C, yield 69 mg (60% over two steps). C_24_H_30_N_2_O_3_, *M*_r_ = 394.5. MS (EM): *m*/*z* calcd. for C_24_H_31_N_2_O_3_ 395.2335, found 395.2333. ^1^H NMR (CDCl_3_): *δ* [ppm] = 1.47–1.97 (m, 7.7H, 2-H, 3-H, 5-H, 6-H_piperidine_ and 3-H), 2.10 (s, 3H, CH_3_), 2.17–2.30 (m, 0.7H, 3-H), 2.32–2.53 (m, 2H, 4-H and 4-H_piperidine_), 2.55–2.67 (m, 1.7H, CH_2_N (2 × 0.7H) and 6-H_piperidine_·), 2.80 (t, *J* = 12.0 Hz, 0.3H, CH_2_N·), 3.04 (d, *J* = 11.4 Hz, 0.3H, 6-H_piperidine_·), 3.29 (d, *J* = 10.9 Hz, 1H, 2-H_piperidine_), 3.48–3.65 (m, 1.3H, 2-H and CH_2_N·), 4.12 (dt, *J* = 12.4/4.0 Hz, 0.7H, 2-H), 4.28 (dt, *J* = 12.2/3.7 Hz, 0.3H, 2-H·), 4.83 (d, *J* = 9.5 Hz, 0.3H, 5-H·), 5.10 (s, 0.7H, 5-H), 7.07 (dd, *J* = 8.3 /2.1 Hz, 0.3H, 7-H·), 7.10–7.28 (m, 6, 7H, C_6_H_5_, 9-H and 7-H), 7.47 (d, *J* = 8.1 Hz, 0.7H, 6-H), 7.65 (d, *J* = 8.5H, 0.3H, 6-H·). Signals for NH or OH protons are not seen in the spectrum. Ratio of *cis*-14b : *trans*-14b according to ^1^H NMR spectrum is 70 : 30. Signals of *trans*-14b are marked with ·. ^13^C NMR (CDCl_3_): *δ* [ppm] = 24.9 (1C, CH_3_), 29.88 (0.3C, C-3·), 32.5 (0.7C, C-3), 33.1, 33.4, 33.8, 34.1 (2C, C-3, C-5_piperidine_), 34.4 (0.3C, C-4·), 36.7 (0.7C, C-4), 37.7 (0.3C, C-4_piperidine_·), 42.5 (0.7C, C-4_piperidine_), 52.8, 53.6, 56.2 (2C, C-2, C-6_piperidine_), 59.8 (0.7C, CH_2_N), 65.3 (0.3C, CH_2_N·), 68.4 (0.7C, C-2), 71.9 (0.3C, C-2·), 74.4 (0.7C, C-5), 75.9 (0.3C, C-5), 112.7 (0.3C, C-9·), 113.0 (0.7C, C-9), 115.1 (0.7C, C-7), 115.2 (0.3C, C-7·), 126.6, 127.3, 128.11 (3C, C-4_Ph_, C-5a, C-6), 127.0 (2C, C-3, C-5_Ph_), 128.7 (2C, C-2, C-6_Ph_), 137.5 (0.3C, C-8·), 137.9 (0.7C, C-8), 145.8 (1C, C-1_Ph_), 157.1 (1C, C-9a), 168.4 (1C, C=O). Ratio of diastereomers is 70 : 30. IR (neat): *ν̃* [cm^−1^] = 3734 (NH, OH), 3672 (NH, OH), 3255 (O–H), 2924 (C–H), 1667 (C=O). Purity (HPLC): 97.6%, *t*_R_ = 15.35 min and 15.95 min.

#### *N*-{*cis*-5-Hydroxy-4-[(4-phenylpiperazin-1yl)methyl]-2,3,4,5-tetrahydro-1-benzoxepin-8-yl}acetamide (*cis*-14c) and *N*-{*trans*-5-hydroxy-4-[(4-phenylpiperazin-1yl)methyl]-2,3,4,5-tetrahydro-1-benzoxepin-8-yl}acetamide (*trans*-14c)


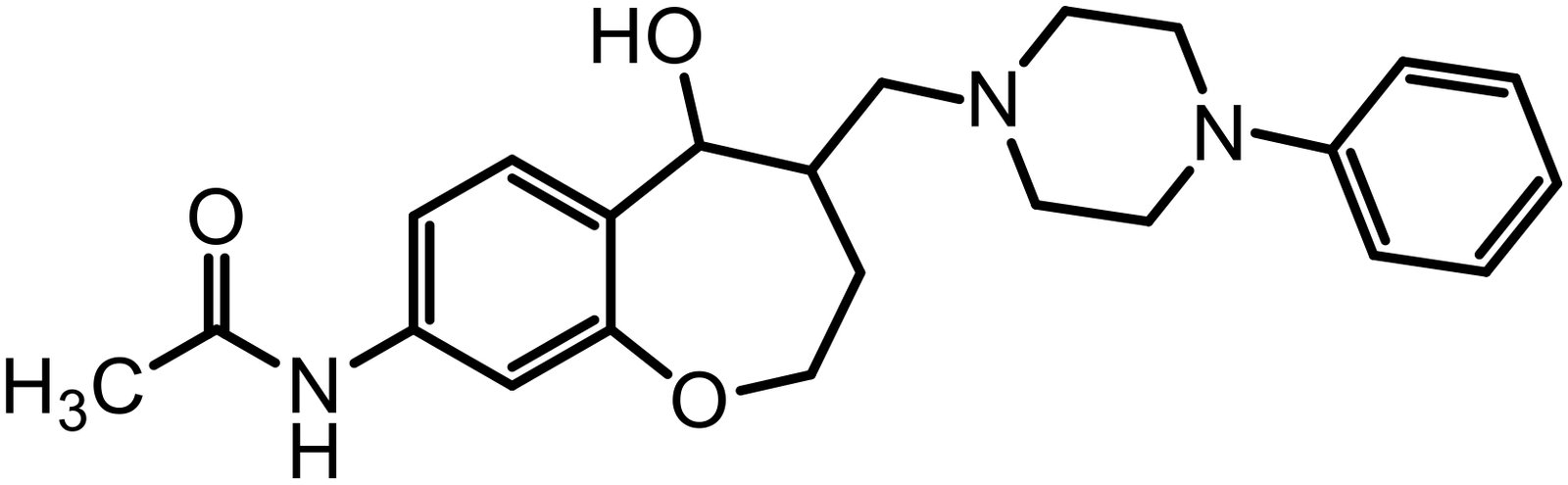
 Ac_2_O (0.05 g, 0.48 mmol) was added to a solution of primary amine 12c (99 mg, 0.28 mmol) in CH_2_Cl_2_ (10 mL) and the mixture was stirred at rt for 2 h. Then, the solvent was removed under reduced pressure and the residue was dissolved in MeOH_abs._ (20 mL). K_2_CO_3_ (0.10 g, 0.7 mmol) was added to this solution and the mixture was stirred for 40 min. For workup water (10 mL) was added and the mixture was extracted with CH_2_Cl_2_. The combined organic layers were dried (Na_2_SO_4_), filtered and the solvent was removed under reduced pressure. The residue was purified by fc (*ø* 3.5 cm, *h* = 15 cm, cyclohexane : ethyl acetate 4 : 1 → 3 : 1 → 2 : 1 + 0.1% *N*,*N*-dimethylethylamine, *R*_f_ = 0.14 (cyclohexane : ethyl acetate 1 : 1 + 0.1% *N*,*N*-dimethylethylamine)). Colorless resin, yield 40 mg (35%). C_23_H_29_N_3_O_3_, *M*_r_ = 395.5. MS (EM): *m*/*z* calcd. for C_23_H_30_N_3_O_3_ 396.2287, found 396.2287. ^1^H NMR (CDCl_3_): *δ* [ppm] = 1.16–1.36 (m, 2H, CH_2 piperazine_), 1.43–1.81 (m, 2H, CH_2 piperazine_), 1.85–1.93 (m, 0.33H, 3-H·), 2.09 (2 × s, 3H, CH_3_), 2.27–2.36 (m, 0.67H, CH_2_N), 2.39 (dd. *J* = 12.6/2.5 Hz, 1H, CH_2_N), 2.47–2.61 (m, 1.33H, 3-H and CH_2_N·), 2.67–2.76 (m, 0.67H, 4-H), 2.76–2.90 (m, 1H, 3-H, 4-H·), 3.09–3.27 (m, 3.67H, CH_2 piperazine_), 3.47–3.67 (m, 1H, 2-H), 3.82–3.93 (m, 0.33, CH_2 piperazine_·), 4.11 (dt, *J* = 12.3/4.0 Hz, 0.33H, 2-H·), 4.27 (dt, *J* = 12.2/3.8 Hz, 0.67H, 2-H), 4.83 (d, *J* = 9.3 Hz, 0.67H, 5-H), 5.07 (s, 0.33H, 5-H·), 6.78–6.90 (m, 3H, 3-H, 5-H_Ph_ and 9-H), 7.07 (dd, *J* = 8.4 /2.1 Hz, 0.67H, 7-H), 7.13–7.25 (m, 3.33H, 2-H, 4-H, 6-H_Ph_ and 7-H·), 7.41 (d, *J* = 8.3 Hz, 0.33H, 6-H·), 7.60 (d, *J* = 8.3 Hz, 0.67H, 6-H). Signals for NH or OH protons are not seen in the spectrum. Ratio of *trans*-14c : *cis*-14c according to ^1^H NMR spectrum is 67 : 33. Signals of *cis*-14c are marked with ·. ^13^C NMR (CDCl_3_): *δ* [ppm] = 23.7, 24.9 (1C, H_3_C), 30.3 (0.67C, C-3), 31.6 (0.33C, C-3·), 34.3 (0.67C, C-4), 37.5 (0.33C, C-4·), 49.4 (1.33C, C_piperazine_), 49.7 (0.67C, C_piperazine_·), 53.3, 53.7 (2C, C_piperazine_), 59.1 (0.33C, CH_2_N·), 64.8 (0.67C, CH_2_N), 71.8 (1C, C-2), 74.5 (0.33C, C-5·), 75.9 (0.67C, C-5), 112.9 (1C, C-9), 113.0 (1C, C-5a), 115.3 (1C, C-7), 116.5 (2C, C-2, C-6_Ph_), 120.4 (1C, C-4_Ph_), 127.3 (1C, C-6), 129.4 (2C, C-3, C-5_Ph_), 142.5 (1C, C-8), 150.9 (1C, C-1_Ph_), 158.1 (1C, C-9a), 172.5 (1C, H_3_C*C*=O). Ratio of diastereomers 67 : 33. IR (neat): *ν̃* [cm^−1^] = 3302 (N–H), 2927 (C–H), 1667 (C=O). Purity (HPLC): 96.8%, *t*_R_ = 14.97 min and 15.62 min.

#### *N*-{*cis*-4-[(Benzylpiperidin-1-yl)methyl]-5-hydroxy-2,3,4,5-tetrahydro-1-benzoxepin-8-yl}acetamide (*cis*-14d) and *N*-{*trans*-4-[(benzylpiperidin-1-yl)methyl]-5-hydroxy-2,3,4,5-tetrahydro-1-benzoxepin-8-yl}acetamide (*trans*-14d)


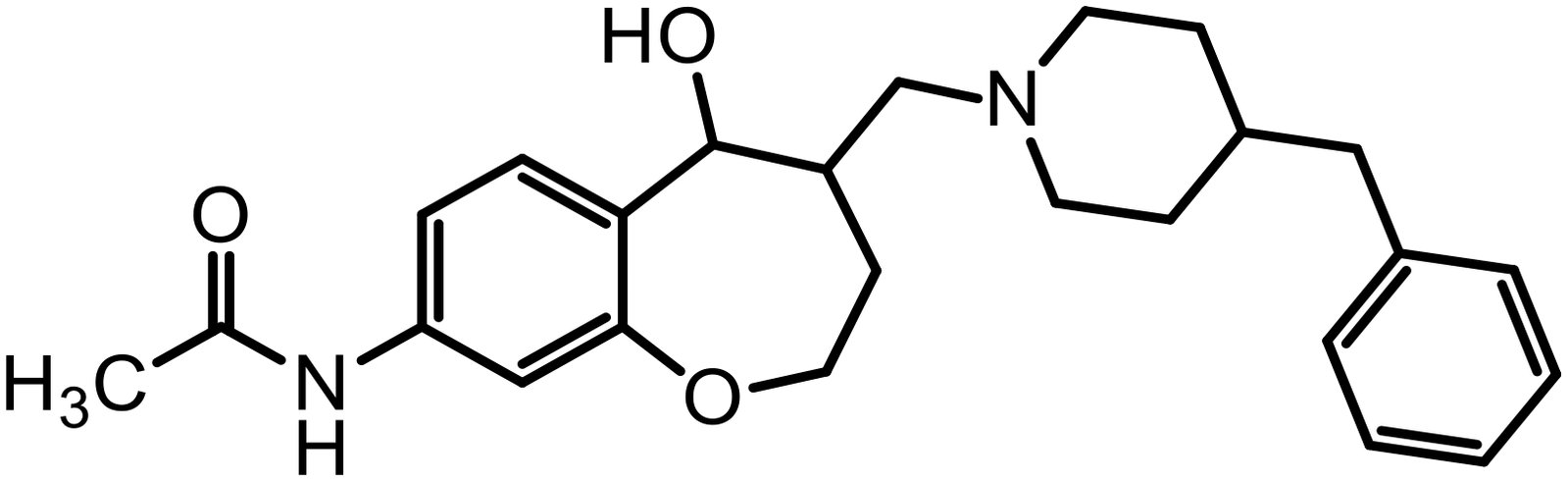
 As described for the synthesis of 14a, a mixture of primary amine 12d (56 mg, 0.15 mmol), Ac_2_O (23 mg, 0.23 mmol) and CH_2_Cl_2_ (12 mL) was stirred at rt for 2 h. The solvent was removed under reduced pressure and the residue was dissolved in MeOH (20 mL). K_2_CO_3_ (0.25 g, 1.8 mmol) was added and the mixture was stirred at rt for 30 min. For workup, saturated aqueous NaHCO_3_ solution (40 mL) was added and the mixture was extracted with CH_2_Cl_2_. The combined organic layers were washed with brine, filtered (Na_2_SO_4_) and the solvent was removed under reduced pressure. The residue was purified by fc (*ø* 3 cm, *h* = 15 cm, cyclohexane : ethyl acetate 2 : 1 + 0.1% *N*,*N*-dimethylethylamine, *R*_f_ = 0.25 (cyclohexane : ethyl acetate 1 : 1 + 0.1% *N*,*N*-dimethylethylamine)). Yellow resin, yield 41 mg (65%). C_25_H_32_N_2_O_3_, *M*_r_ = 408.5. MS (EM): *m*/*z* calcd. for C_25_H_33_N_2_O_3_ 409.2491, found 409.2484. ^1^H NMR (CDCl_3_): *δ* [ppm] = 1.03–1.91 (m, 8.25H, 2-H, 3-H, 4-H, 5-H, 6-H_piperidine_ and 3-H (1.25H)), 1.97 (d, *J* = 12.3 Hz, 0.75H, CH_2_N), 2.10 (s, 3H, CH_3_), 2.13–2.21 (m, 0.75H, 3-H), 2.28 (d, *J* = 9.9 Hz, 0.25H, CH_2_N·), 2.34–2.43 (m, 1.75H, CH_2_N and 4-H), 2.46 (d, *J* = 7.1 Hz, 2H, C*H*_2_C_6_H_5_), 2.55 (d, *J* = 7.9 Hz, 0.75H, 6-H_piperidine_), 2.71 (t, *J* = 12.1 Hz, 0.25H, CH_2_N·), 2.83–3.00 (m, 0.25H, 6-H_piperidine_·), 3.03–3.16 (m, 1H, 2-H_piperidine_), 3.51 (t, *J* = 11.4 Hz, 1H, 2-H), 4.10 (dt, *J* = 12.3/3.8 Hz, 0.75H, 2-H), 4.26 (dt, *J* = 7.4/3.3 Hz, 0.25H, 2-H·), 4.79 (d, *J* = 9.1 Hz, 0.25H, 5-H·), 5.06 (s, 0.75H, 5-H), 7.04–7.10 (m, 4H, 2-H, 6-H–C_6_H_5_, 7-H and 9-H), 7.11–7.28 (m, 3H, 3-H, 3-H, 5-H–C_6_H_5_), 7.44 (d, *J* = 8.1 Hz, 0.75H, 6-H), 7.63 (d, *J* = 8.5 Hz, 0.25H, 6-H·). Signals for NH or OH protons are not seen in the spectrum. Ratio of *cis*-14d : *trans*-14d according to ^1^H NMR spectrum is 75 : 25. Signals of *trans*-14d are marked with ·. IR (neat): *ν̃* [cm^−1^] = 3302 (N–H), 3263 (O–H), 2924 (C–H), 1667 (C=O). Purity (HPLC): 96.5%, *t*_R_ = 15.80 min and 16.39 min.

#### *N*-{*cis*-5-Hydroxy-4-[(piperidin-1-yl)methyl]-2,3,4,5-tetrahydro-1-benzoxepin-8-yl}isobutyramide (*cis*-15a) and *N*-{*trans*-5-hydroxy-4-[(piperidin-1-yl)methyl]-2,3,4,5-tetrahydro-1-benzoxepin-8-yl}isobutyramide (*trans*-15a)


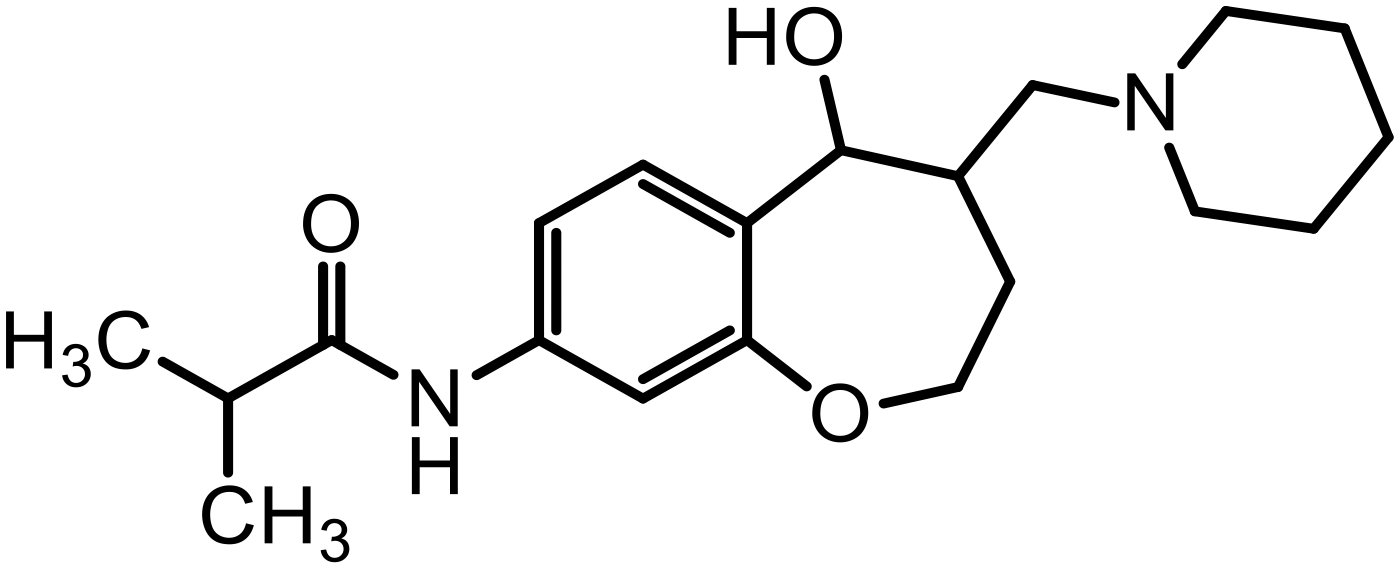
 Isobutyric anhydride (0.30 g, 1.9 mmol) was added to a solution of primary amine 12a (0.110 g, 0.40 mmol) in MeOH_abs._ (20 mL). The mixture was stirred for 3 h at 80 °C. After cooling down the mixture was stirred overnight for 12 h. Then, water (20 mL) was added and the mixture was extracted with CH_2_Cl_2_. The CH_2_Cl_2_ layer was evaporated under reduced pressure. The residue was dissolved in MeOH (20 mL), K_2_CO_3_ (0.10 g, 13.1 mmol) was added and the mixture was stirred for 2 h. For workup, water (20 mL) was added and the mixture was extracted with CH_2_Cl_2_. The combined organic layers were washed with brine, dried (Na_2_SO_4_), filtered and the solvent was removed under reduced pressure. The residue was purified by fc (*ø* 2 cm, *h* = 20 cm, cyclohexane : ethyl acetate 3 : 1 + 0.1% *N*,*N*-dimethylethylamine, *R*_f_ = 0.30 (cyclohexane : ethyl acetate 1 : 1 + 0.1% *N*,*N*-dimethylethylamine)). Colorless resin, yield 100 mg (72%). C_20_H_30_N_2_O_3_, *M*_r_ = 346.5. MS (EM): *m*/*z* calcd. for C_20_H_31_N_2_O_3_ 347.2335, found 347.2340. MS (EI). *m*/*z* (%) 346 (M, 4), 98 (M–CH_2_–N(CH_2_)_5_, 100). ^1^H NMR (CDCl_3_): *δ* [ppm] = 1.19 (2 × d, *J* = 6.9 Hz, 6H, CH(C*H*_3_)_2_), 1.28–1.45 (m, 2H, 4-H_piperidine_), 1.46–1.64 (m, 5.8H, 3-H, 5-H_piperidine_, 4-H (0.6H) and 3-H (2 × 0.6H)), 1.65–1.85 (m, 1.2H 3-H·(2 × 0.4H), 4-H·), 1.93–2.12 (m, 2.2H, 2-H or 6-H_piperidine_ and CH_2_N (2 × 0.6H)), 2.12–2.24 (m, 0.6H, (CH_3_)_2_C*H*), 2.29 (dd, *J* = 12.7/2.5 Hz, 0.4H, CH_2_N·), 2.32–2.61 (m, 3.4H, 2-H, 6-H_piperidine_, and (CH_3_)_2_C*H*·), 2.67 (t, 0.4H, CH_2_N·), 3.50 (dd, *J* = 16.7/8.0 Hz, 1H, 2-H), 4.10 (dt, *J* = 12.3/3.9 Hz, 0.6H, 2-H), 4.26 (dt, *J* = 12.1/3.7 Hz, 0.4H, 2-H·), 4.80 (d, *J* = 9.1 Hz, 0.4H, 5-H·), 5.07 (s, 0.6H, 5-H), 7.04 (d, *J* = 8.7 Hz, 1H, NH), 7.10 (dd, *J* = 8.4/2.0 Hz, 0.4H, 7-H·), 7.15–7.28 (m, 1.6H, 7-H, 9-H), 7.45 (d, *J* = 8.2 Hz, 0.6H, 6-H), 7.63 (d, *J* = 8.3 Hz, 0.4H, 6-H·). Signals for OH protons are not seen in the spectrum. Ratio of *cis*-15a : *trans*-15a according to ^1^H NMR spectrum is 60 : 40. Signals of *trans*-15a are marked with ·.^13^C NMR (CDCl_3_): *δ* [ppm] = 19.8 (2C, (CH(*C*H_3_)_2_), 24.3 (1C, C-4_piperidine_), 26.0, 26.4, 27.1 (2C, C-3, C-5_piperidine_), 32.6 (0.6C, C-3), 34.4 (0.4C, C-3·), 36.5 (1C, (CH_3_)_2_*C*H), 36.9, 37.5 (2C, C-3, C-5_piperidine_), 55.1 (1C, C-4), 60.1 (0.6C, CH_2_N), 65.7 (0.4C, CH_2_N·), 68.3 (0.6C, C-2), 71.9 (0.4C, C-2·), 74.5 (0.6C, C-5), 75.9 (0.4C, C-5·), 112.6 (0.4C, C-9·), 113.9 (0.6C, C-9), 115.2 (1C, C-7), 127.3 (0.4C, C-6·), 128.0 (0.6C, C-6), 132.2, 133.9 (1C, C-5a), 137.1 (0.4C, C-8·), 137.9, 0.6C, C-8), 157.0 (0.6C, C-9a), 157.8 (0.4C, C-9a·), 175.4 (1C, C=O). Ratio of diastereomers is 60 : 40. IR (neat): *ν̃* [cm^−1^] = 3290 (O–H), 2927 (C–H), 2850 (C–H), 2808 (C–H), 1667 (C=O). Purity (HPLC): 96.0%, *t*_R_ = 12.49 min and 13.24 min.

During the fc purification (described above) of the crude product diastereomerically pure *trans*-15a could be isolated. C_20_H_30_N_2_O_3_, *M*_r_ = 346.5. MS (EM): *m*/*z* calcd. for C_20_H_31_N_2_O_3_ 347.2335, found 347.2327. ^1^H NMR (CDCl_3_): *δ* [ppm] = 1.18 (d, *J* = 6.9 Hz, 6H, CH(C*H*_3_)_2_), 137–1.44 (m, 2H, 4-H_piperidine_), 1.45–1.64 (4H, 3-H, 5-H_piperidine_), 1.64–1.88 (m, 3H, 3-H, 4-H), 2.29 (dd, *J* = 12.7/2.6 Hz, 1H, CH_2_N), 2.31–2.37 (m, 2H, 2-H, 6-H_piperidine_), 2.42 (septet, *J* = 13.5/6.9 Hz, 1H, (CH_3_)_2_C*H*), 2.45 (m, 2H, 2-H, 6-H_piperidine_), 2.69 (t, *J* = 12.3 Hz, 1H, CH_2_N), 3.47–3.56 (m, 1H, 2-H), 4.26 (dt, *J* = 12.1/3.7 Hz, 1H, 2-H), 4.80 (d, *J* = 9.1 Hz, 1H, 5-H), 7.03 (s, 1H, NH), 7.10 (dd, *J* = 8.5/2.1 Hz, 1H, 7-H), 7.24 (d, *J* = 2.0 Hz, 1H, 9-H), 7.63 (d, *J* = 8.5 Hz, 1H, 6-H). A signal of the OH proton is not seen in the spectrum. ^13^C NMR (CDCl_3_): *δ* [ppm] = 19.8 (2C, CH(*C*H_3_)_2_), 24.3 (1C, C-4_piperidine_), 26.0 (2C, C-3, C-5_piperidine_), 34.3 (1C, C-3), 36.8 (1C, (H_3_C)_2_*C*H), 37.7 (2C, C-2, C-6_piperidine_), 56.4 (1C, C-4), 65.7 (1C, CH_2_N), 71.9 (1C, C-2), 75.9 (1C, C-5), 112.7 (1C, C-9), 115.2 (1C, C-7), 127.2 (1C, C-6), 137.6 (1C, C-5a), 146.1 (1C, C-8), 157.8 (1C, C-9a), 175.4 (1C, C=O). Purity (HPLC): 96.2%, *t*_R_ = 13.15 min.

#### *N*-{*cis*-5-Hydroxy-4-[(4-phenylpiperidin-1-yl)methyl]-2,3,4,5-tetrahydro-1-benzoxepin-8-yl}isobutyramide (*cis*-15b) and *N*-{*trans*-5-Hydroxy-4-[(4-phenylpiperidin-1-yl)methyl]-2,3,4,5-tetrahydro-1-benzoxepin-8-yl}isobutyramide (*trans*-15b)


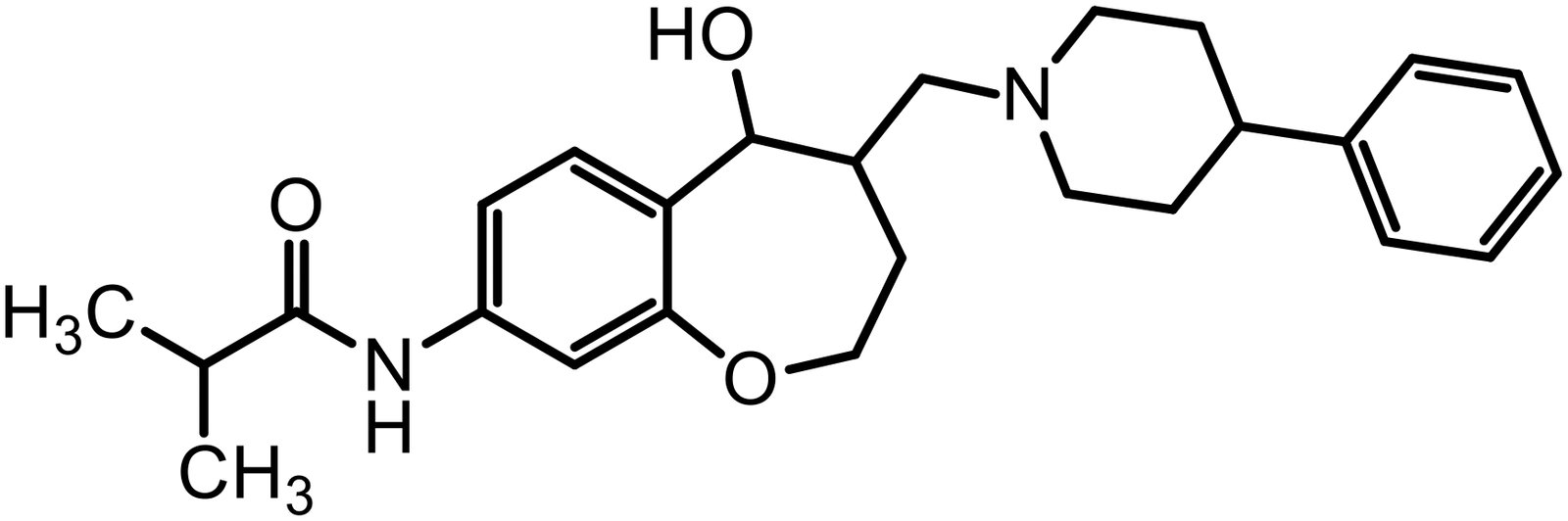
 Isobutyric anhydride (63 mg, 1.2 mmol) was added to a solution of primary amine 12b (0.118 g, 0.34 mmol) in MeOH (15 mL) and the mixture was heated to 80 °C for 20 min. After cooling to rt saturated aqueous NaHCO_3_ solution (20 mL) was added and the mixture was extracted with CH_2_Cl_2_. The solvent was removed under reduced pressure. Then, the residue was dissolved in MeOH (20 mL) and K_2_CO_3_ (100 mg, 0.71 mmol) was added. The mixture was stirred for 2 h at rt. For workup, saturated aqueous NaHCO_3_ solution (20 mL) was added and the mixture was extracted with CH_2_Cl_2_. The combined organic layers were dried (Na_2_SO_4_), filtered and the solvent was removed under reduced pressure. The residue was purified by fc (*ø* 3 cm, *h* = 15 cm, cyclohexane : ethyl acetate 3.5 : 1 + 0.1% *N*,*N*-dimethylethylamine, *R*_f_ = 0.71 (cyclohexane : ethyl acetate 1 : 1 + 0.1% *N*,*N*-dimethylethylamine)). Colorless solid, mp 79 °C, yield 51 mg (36%). C_26_H_34_N_2_O_3_, *M*_r_ = 422.6. MS (EM): *m*/*z* calcd. for C_26_H_35_N_2_O_3_ 423.2648, found 423.2632. ^1^H NMR (CDCl_3_): *δ* [ppm] = 1.18 (d, *J* = 6.8 Hz, 6H, CH(C*H*_3_)_2_), 1.32–1.95 (m, 7.83H, 2-H, 3-H, 5-H, 6-H (0.83H)_piperidine_ and 3-H), 2.16–2.29 (m, 1H, 4-H_piperidine_), 2.33–2.57 (m, 3.34H, CH_2_N (1.17H), 6-H_piperidine_· (0.17H), (CH_3_)_2_C*H*, 4-H), 2.78 (t, *J* = 10.5 Hz, 0.83H, CH_2_N), 3.03 (d, *J* = 11.1 Hz, 0.83H, 6-H_piperidine_), 3.16 (d, *J* = 5.3 Hz, 0.17H, 6H_piperidine_·), 3.26 (d, *J* = 10.7 Hz, 1H, 2-H_piperidine_), 3.54 (t, *J* = 11.5 Hz, 1H, 2-H), 4.31–4.23 (m, 1H, 2-H), 4.83 (d, *J* = 9.4 Hz, 0.83H, 5-H), 5.02 (s, 0.17H, 5-H·), 7.02 (s, 1H, NH), 7.08–7.35 (m, 7H, C_6_H_5_, 7-H, 9-H), 7.65 (d, *J* = 8.4 Hz, 0.83H, 6-H), 7.80 (d, *J* = 7.1 Hz, 0.17H, 6-H·). A signal for the OH proton is not seen in the spectrum. Ratio of *trans*-15b : *cis*-15b according to ^1^H NMR spectrum is 83 : 17. Signals of the *cis*-15b are marked with ·. ^13^C NMR (CDCl_3_): *δ* [ppm] = 19.83 (2C, CH(*C*H_3_)_2_), 33.2, 33.6, 33.9, 34.4 (5C, *C*H(CH_3_)_2_, C-3, C-5_piperidine_ and C-3 and C-4), 42.6 (1C, C-4_piperidine_), 52.8 (1C, C-2_piperidine_), 56.2 (1C, C-6_piperidine_), 65.3 (1C, CH_2_N), 71.2 (0.17C, C-2·), 71.9 (0.83C, C-2), 74.0 (0.17C, C-5·), 75.9 (0.83C, C-5), 110.3, 112.6, 115.1 (2C, C-7, C-9), 126.1 (0.17C, C-4_Ph_·), 126.6 (0.83C, C-4_Ph_), 127.2, 127.5, 128.6, 128.7 (6C, C-2, C-3, C-5, C-6_Ph_, C-5a and C-6), 136.8 (1C, C-8), 145.9 (1C, C-1_Ph_), 157.9 (1C, C-9a), 175.2 (1C, C=O). Ratio of diastereomers is 83 : 17. IR (neat): *ν̃* [cm^−1^] = 3302 (N–H), 2924 (C–H), 2870 (C–H), 1667 (C=O). Purity (HPLC): 93.9%, *t*_R_ = 17.12 min and 17.53 min.

#### *N*-{*cis*-4-[(4-Benzylpiperidin-1yl)methyl]-5-hydroxy-2,3,4,5-tetrahydro-1-benzoxepin-8-yl}isobutyramide (*cis*-15d) and *N*-{*trans*-4-[(4-benzylpiperidin-1yl)methyl]-5-hydroxy-2,3,4,5-tetrahydro-1-benzoxepin-8-yl}isobutyramide (*trans*-15d)


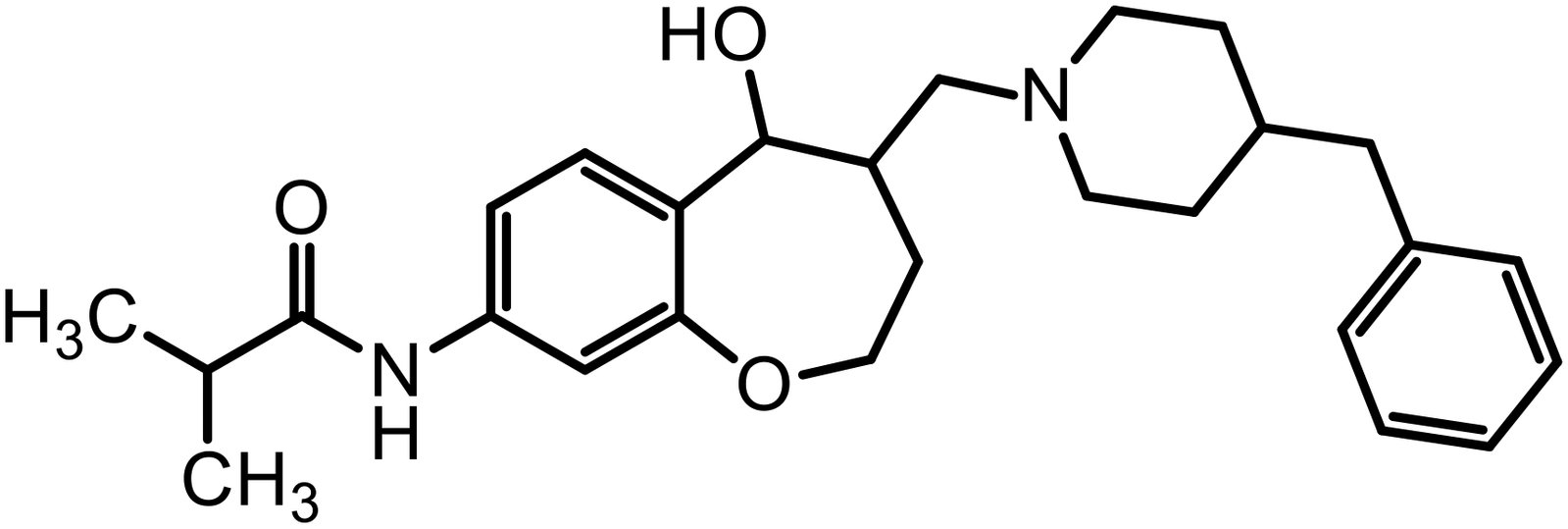
 A solution of primary amine 12d (44 mg, 0.12 mmol), isobutyric anhydride (23 mg, 0.14 mmol) and CH_3_OH (15 mL) was stirred at 80 °C for 20 min. Then, saturated aqueous NaHCO_3_ solution was added (10 mL) and the mixture was extracted with CH_2_Cl_2_. The organic layers were combined and the solvent was removed under reduced pressure. The residue was dissolved in MeOH (15 mL), K_2_CO_3_ (100 mg, 0.72 mmol) was added and the mixture was stirred for 12 h at rt. For workup, saturated aqueous NaHCO_3_ solution was added and the mixture was extracted with CH_2_Cl_2_. The combined organic layers were dried (Na_2_SO_4_), filtered and the solvent was removed under reduced pressure. The residue was purified by fc (*ø* 3.5 cm, *h* = 15 cm, cyclohexane ethyl acetate + 0.1% *N*,*N*-dimethylethylamine 5 : 1 → 4.5 : 1 → 3 : 1 → 2.5 : 1, *R*_f_ = 0.47 (cyclohexane : ethyl acetate 1 : 1 + 0.1% *N*,*N*-dimethylethylamine)). Colorless resin, yield 15 mg (28%). C_27_H_36_N_2_O_3_, *M*_r_ = 436.6. MS (EM): *m*/*z* calcd. for C_27_H_37_N_2_O_3_ 437.2804, found 437.2809. ^1^H NMR (CDCl_3_): *δ* [ppm] = 1.18 (d, *J* = 6.8 Hz, 6H, CH(C*H*_3_)_2_), 1.36–1.95 (m, 8.2H, 2-H, 3-H, 4-H, 5-H, 6-H_piperidine_ and 3-H (1.2H)), 1.96 (dd, *J* = 10.2/1.8 Hz, 0.8H, CH_2_N), 2.09–2.21 (m, 0.8H, 3-H), 2.28 (dd, *J* = 10.3/2.4 Hz, 0.2H, CH_2_N·), 2.34–2.49 (m, 3.8H, CH_2_C_6_H_5_, CH_2_N (0.8H), 4-H), 2.34–2.49 (m, 1.8H, (CH_3_)_2_C*H* and 2-H or 6-H_piperidine_ (0.8H)), 2.64–2.82 (m, 0.2H, CH_2_N·), 2.91–3.03 (m, 0.2H, 2-H or 6-H_piperidine_·), 3.06–3.15 (m, 1H, 2-H, 6-H_piperidine_), 3.49 (t, *J* = 11.4 Hz, 1H, 2-H), 4.09 (dt, *J* = 12.4/3.9 Hz, 0.8H, 2-H), 4.14–4.32 (m, 0.2H, 2-H·), 4.79 (d, *J* = 8.9 Hz, 0.2H, 5-H·), 5.06 (s, 0.8H, 5-H), 7.03–7.25 (m, 7H, 7-H, 9-H and C_6_H_5_), 7.43 (d, *J* = 8.1 Hz, 0.8H, 6-H), 7.61 (t, *J* = 8.6 Hz, 0.2H, 6-H·). Signals for NH or OH protons are not seen in the spectrum. Ratio of *cis*-15d : *trans*-15d according to ^1^H NMR spectrum is 80 : 20. Signals of *trans*-15d are marked with ·. ^13^C NMR (CDCl_3_): *δ* [ppm] = 19.84 (2C, CH(*C*H_3_)_2_), 29.9 (1C, *C*H(CH_3_)_2_), 32.4, 32.5 (2C, C-3, C-5_piperidine_), 33.0 (1C, C-3), 36.7 (0.8C, C-4), 37.0 (0.2C, C-4·), 37.9 (0.8C, C-4_piperidine_), 39.3 (0.2C, C-4_piperidine_·), 43.5 (1C, *C*H_2_C_6_H_5_), 53.2, 55.2, (2C, C-2, C-6_piperidine_), 59.6 (0.8C, CH_2_N), 65.3 (0.2C, CH_2_N·), 66.6 (0.2C, C-2·), 68.3 (0.8C, C-2), 74.5 (1C, C-5), 113.0 (1C, C-9), 115.2 (1C, C-7), 126.1 (1C, C-4-C_6_H_5_), 128.0, 128.4 (2C, C-2, C-6-C_6_H_5_), 128.7 (1C, C-5a), 129.3 (2C, C-3, C-5-C_6_H_5_), 132.1 (1C, C-6), 137.9 (1C, C-8), 140.7 (1C, C-1-C_6_H_5_), 156.8 (1C, C-9a), 175.4 (1C, C=O). Ratio of diastereomers is 80 : 20. IR (neat): *ν̃* [cm^−1^] = 3302 (N–H), 3279 (O–H), 2924 (C–H), 2851 (C–H), 1670 (C=O). Purity (HPLC): 64.7%, *t*_R_ = 17.13 min and 17.66 min.

#### *N*-{*cis*-5-Hydroxy-4-[(4-phenylpiperidin-1-yl)methyl]-2,3,4,5-tetrahydro-1-benzoxepin-8-yl}benzamide (*cis*-16b)


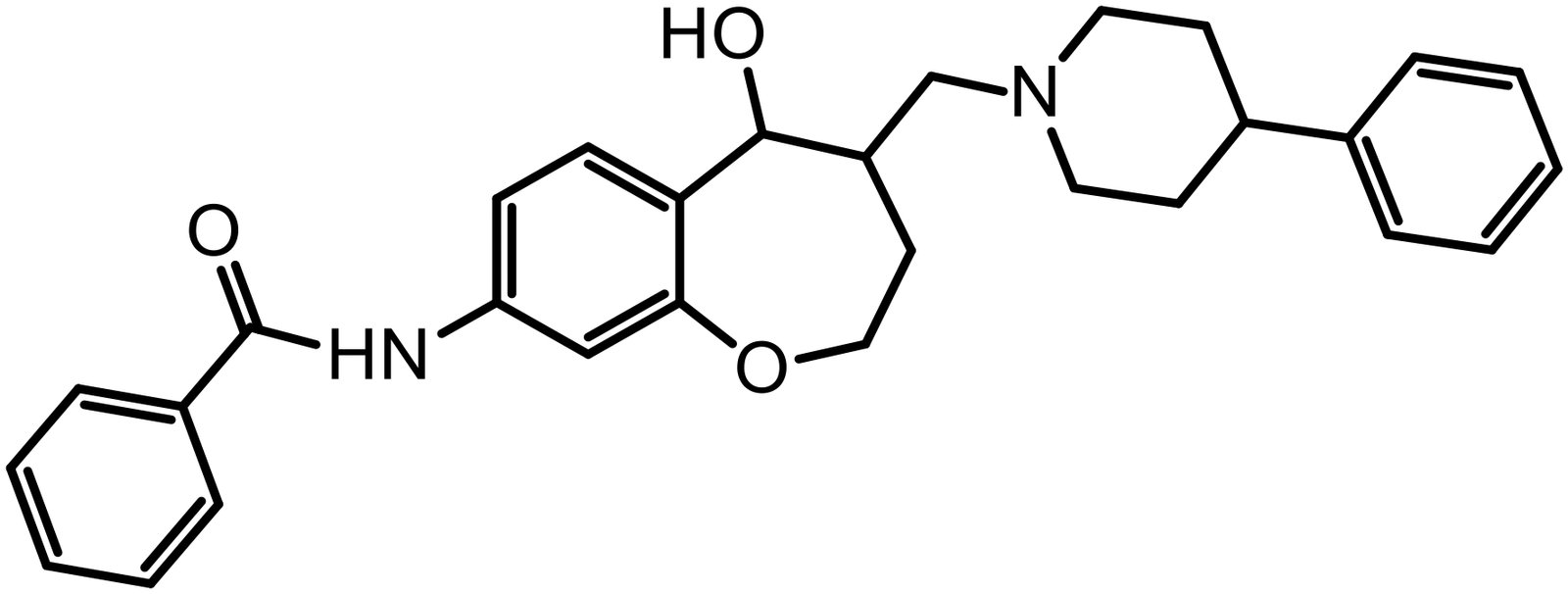
 A mixture of primary amine 12b (100 mg, 0.28 mmol) and NEt_3_ (29 mg, 0.28 mmol), dissolved in THF_abs._ (5 mL), was added dropwise to a solution of benzoyl chloride (47 mg, 0.34 mmol) in THF_abs._ (5 mL). The mixture was stirred at rt for 1 h. Then, saturated aqueous NaHCO_3_ solution was added and the mixture was extracted with CH_2_Cl_2_. The organic layers were combined and the solvent was removed under reduced pressure. The residue was dissolved in MeOH (10 mL), NaOH pellets (0.2 g, 4.5 mmol) were added and the mixture was stirred at rt for 12 h. For workup, saturated aqueous NaHCO_3_ solution was added and the mixture was extracted with CH_2_Cl_2_. The combined organic layers were washed with brine, dried (Na_2_SO_4_), filtered and the solvent was removed under reduced pressure. The residue was purified by fc (*ø* 3 cm, *h* = 15 cm, cyclohexane : ethyl acetate 3.5 : 1 + 0.1% *N*,*N*-dimethylethylamine, *R*_f_ = 0.30 (cyclohexane : ethyl acetate 1 : 1 + 0.1% *N*,*N*-dimethylethylamine)). A diastereomerically pure product was obtained. Colorless solid, mp 112 °C, yield 75 mg (59%). C_29_H_32_N_2_O_3_, *M*_r_ = 456.6. MS (EM): *m*/*z* calcd. for C_29_H_33_N_2_O_3_ 457.2491, found 457.2508. ^1^H NMR (CDCl_3_): *δ* [ppm] = 1.55–1.68 (m, 1H, 3-H), 1.69–2.03 (m, 5H, 2-H, 3-H, 5-H_piperidine_), 2.04–2.18 (m, 2H, CH_2_N and 6-H_piperidine_), 2.21–2.31 (m, 1H, 3-H), 2.42–2.61 (m, 2H, CH_2_N and 4-H_piperidine_), 2.61–2.76 (m, 2H, 6-H_piperidine_ and 4-H), 3.35 (d, *J* = 11.1 Hz, 1H, 2-H_piperidine_), 3.63 (t, *J* = 11.5 Hz, 1H, 2-H), 4.20 (dt, *J* = 12.3/3.7 Hz, 1H, 2-H), 5.18 (s, 1H, 5-H), 7.10–7.70 (m, 11H, C_6_H_5_, 3-H, 4-H, 5-H_Bz_, 6-H, 7-H, 9-H), 7.86 (d, *J* = 7.2 Hz, 2H, 2-H, 6-H_Bz_), 7.91 (s, 1H, NH). A signal for the OH proton is not seen in the spectrum. ^13^C NMR (CDCl_3_): *δ* [ppm] = 32.5 (1C, C-3), 33.5, 34.2 (2C, C-3, C-5_piperidine_), 36.7 (1C, C-4), 42.5 (1C, C-4_piperidine_), 53.6 (1C, C-2_piperidine_), 56.2 (1C, C-6_piperidine_), 59.8 (1C, CH_2_N), 68.4 (1C, C-2), 74.6 (1C, C-5), 113.4 (1C, C-9), 115.6 (1C, C-7), 126.5 (1C, C-4-C_6_H_5_), 127.0 (2C, C-2, C-6_Bz_), 127.2 (1C, C-6), 128.1, 128.7 (4C, C-3, C-5_Bz_ and C-3, C-5-C_6_H_5_), 129.0 (2C, C-2, C-6-C_6_H_5_), 132.1 (1C, C-5a), 132.6 (1C, C-4_Bz_), 135.2 (1C, C-1_Bz_), 137.9 (1C, C-8), 145.9 (1C, 1C-C_6_H_5_), 157.2 (1C, C-9a), 165.9 (1C, C=O). IR (neat): *ν̃* [cm^−1^] = 3703, 3680 (N–H), 2925 (C–H), 2866 (C–H), 1651 (C=O). Purity (HPLC): 92.9%, *t*_R_ = 18.08 min.

#### *N*-Benzyl-4-[(4-phenylpiperidin-1-yl)methyl]-2,3-dihydro-1-benzoxepin-8-amine (17b)


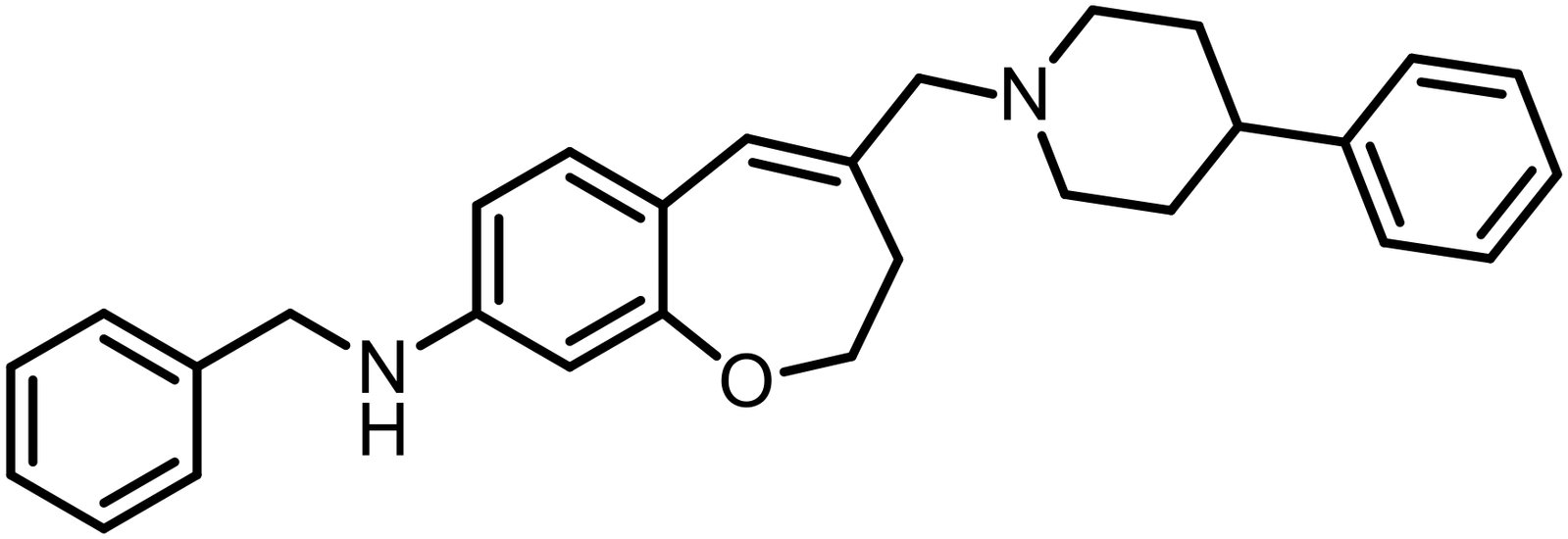
 H_2_SO_4_ conc. (0.07 g, 0.68 mmol) was added to a solution of γ-amino alcohol 11b (100 mg, 0.226 mmol) in THF_abs._ (10 mL). The mixture was stirred for 24 h at rt. For workup saturated NaHCO_3_ solution was added until the pH reached 7. The mixture was extracted with CH_2_Cl_2_ (3×). The combined organic layers were dried (Na_2_SO_4_), filtered and the solvent was removed *in vacuo*. The resulting residue was purified by fc three times (*ø* 3 cm, cyclohexane : ethyl acetate 4 : 1 + 0.1% *N*,*N*-dimethylethylamine, 15 cm, 30 mL, *ø* 3 cm, cyclohexane : ethyl acetate 4 : 1 + 0.1% *N*,*N* dimethylethylamine, 15 cm, 30 mL, *ø* 3 cm, cyclohexane : ethyl acetate 6 : 1 + 0.1% *N*,*N*-dimethylethylamine, *R*_f_ = 0.77 (cyclohexane : ethyl acetate 2 : 1 + 0.1% *N*,*N*-dimethylethylamine)). Yellow resin, yield 18.5 mg (19%). C_29_H_32_N_2_O, *M*_r_ = 424.6. MS (EM): *m*/*z* calcd. for C_29_H_33_N_2_O 425.2593, found 425.2604. ^1^H NMR (CDCl_3_): *δ* [ppm] = 1.11–1.31 (m, 2H, 3-H and 5-H_piperidine_), 1.62–1.86 (m, 2H, 3-H and 5-H_piperidine_), 1.86–1.99 (m, 3H, 2-H and 6-H_piperidine_), 2.36–2.49 (m, 1H, 4-H_piperidine_), 2.60 (t, *J* = 4.4 Hz, 2H, 3-H), 2.88–2.98 (3H, 2-H or 6-H_piperidine_ and N*C*H_2_C), 4.03 (s, 1H, BnN*H*), 4.15 (t, *J* = 4.8 Hz, 2H, OCH_2_), 4.24 (s, 2H, NC*H*_*2*_), 6.09 (s, 1H, 5-H), 6.16 (d, *J* = 2.1 Hz, 1H, 9-H), 6.20 (dd, *J* = 8.3/2.3 Hz, 1H, 7-H), 6.88 (d, *J* = 8.3 Hz, 1H, 6-H), 7.03–7.37 (m, 10H, 2 × C_6_H_5_). ^13^C NMR (CDCl_3_): *δ* [ppm] = 33.8 (2C, C-3 and C-5_piperidine_), 36.0 (1C, C-3), 43.1 (1C, C-4_piperidine_), 48.4 (1C, *C*H_2 Bn_), 54.6 (2C, C-2 and C-6_piperidine_), 68.3 (1C, N*C*H_2._), 69.4 (1C, C-2), 103.5 (1C, C-9), 107.7 (1C, C-7), 116.1 (1C, C-5a), 126.3 (1C, C-4_Bn_), 127.1 (2C, C-2 and C-6_Bn_), 127.5 (1C, C-4-C_6_H_5_), 127.8 (2C, C-3 and C-5_Bn_), 128.6 (2C, C-3-C_6_H_5_ and C-5-C_6_H_5_), 128.9 (2C, C-2 and C-6-C_6_H_5_), 129.3 (1C, C-5), 134.1 (1C, C-6), 135.0 (1C, C-1_Bn_), 139.4 (1C, C-4), 146.8 (1C, C-1-C_6_H_5_), 148.2 (1C, C-8), 160.3 (1C, C-9a). IR (neat): *ν̃* [cm^−1^] = 3363 (N–H), 2796 (CH_2_), 2750 (CH_2_), 1600 (s, C=C). Purity (HPLC): 92.3%, *t*_R_ = 20.00 min.

#### *N*-Benzyl-4-[(4-benzylpiperidin-1-yl)methyl]-2,3-dihydro-1-benzoxepin-8-amine (17d)


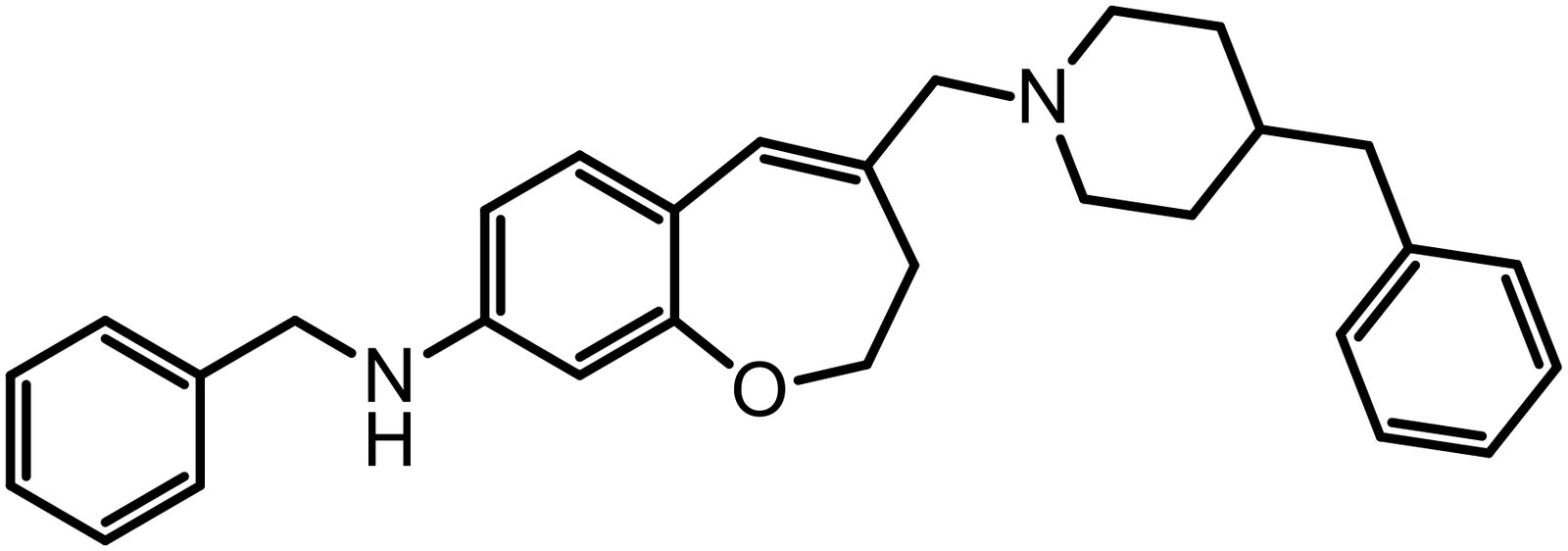
 H_2_SO_4 conc._ (0.5 mL, 9.4 mmol) was added to a solution of γ-amino alcohol 11d (134 mg, 0.29 mmol) in THF. The mixture was stirred for 24 h at rt. The transformation was terminated by addition of NaOH (10 M, 2 mL) and water (5 mL). The mixture was extracted with CH_2_Cl_2_. The combined organic layers were washed with brine, dried (Na_2_SO_4_), filtered and the solvent was removed under reduced pressure. The residue was purified by fc (*ø* 3 cm, *h* = 15 cm, cyclohexane : ethyl acetate 5 : 1 + 0.1% *N*,*N*-dimethylethylamine, *R*_f_ = 0.19). Pale yellow crystals, mp 110 °C, yield 87 mg (68%). C_30_H_34_N_2_O, *M*_r_ = 438.6. MS (EM): *m*/*z* calcd. for C_30_H_35_N_2_O 439.2749, found 439.2753. ^1^H NMR (CDCl_3_): *δ* [ppm] = 1.10–1.31 (m, 2H, 3-H, 5-H_piperidine_), 1.39–1.47 (m, 1H, 4-H_piperidine_), 1.48–1.59 (m, 2H, 3-H, 5-H_piperidine_), 1.67–1.78 (m, 2H, 2-H, 6-H_piperidine_), 2.45 (d, *J* = 7.0 Hz, 2H, C*H*_2_C_6_H_5_), 2.55 (t, *J* = 4.6 Hz, 2H, 3-H), 2.75 (d, *J* = 11.7 Hz, 2H, 2-H, 6-H_piperidine_), 2.87 (s, 2H, CH_2_N_piperidine_), 3.99 (s (broad), 1H, NH), 4.12 (d, *J* = 5.1 Hz, 2H, 2-H), 4.24 (d, *J* = 5.2 Hz, 2H, Ph–C*H*_2_NH), 6.04 (s, 1H, 5-H), 6.15 (d, *J* = 2.4 Hz, 1H, 9-H), 6.19 (dd, *J* = 8.3/2.5 Hz, 1H, 7-H), 6.85 (d, *J* = 8.3 Hz, 1H, 6-H), 7.03–7.15 (m, 3H, 2-H, 4-H, 6-H–C_6_H_5_), 7.17–7.23 (m, 3H, 3-H, 5-H C_6_H_5_ and 4-H_Bn_), 7.25–7.30 (m, 4H, 2-H, 3-H, 5-H, 6-H_Bn_). ^13^C NMR (CDCl_3_): *δ* [ppm] = 30.4 (2C, C-3, C-5_piperidine_), 35.9 (1C, C-3), 38.3 (1C, C-4_piperidine_), 43.5 (1C, *C*H_2_C_6_H_5_), 48.4 (1C, Ph–CH_2_NH), 54.1 (2C, C-2, C-6_piperidine_), 68.2 (1C, CH_2_N_piperidine_), 69.4 (1C, C-2), 103.5 (1C, C-9), 107.7 (1C, C-7), 116.1 (1C, C-5a), 125.9 (1C, C-4-C_6_H_5_), 126.8 (1C, C-5), 127.5 (1C, C-4_Bn_), 127.7 (2C, C-2, C-6_Bn_ or C-3, C-5_Bn_), 128.4 (2C, C-3, C-5-C_6_H_5_), 128.9 (2C, C-2, C-6_Bn_ or C-3, C-5_Bn_), 129.4 (2C, C-2, C-6-C_6_H_5_), 134.1 (1C, C-6), 135.1 (1C, C-1_Bn_), 139.4 (1C, C-1-C_6_H_5_), 141.1 (1C, C-4), 148.2 (1C, C-8), 160.2 (1C, C-9a). IR (neat): *ν̃* [cm^−1^] = 3024 (C–H_arom._), 2920 (C–H), 1612 (N–H). Purity (HPLC): 98.1%, *t*_R_ = 20.55 min.

#### 4-[(4-Phenylpiperidin-1-yl)methyl]-2,3-dihydro-1-benzoxepin-8-amine (18b)


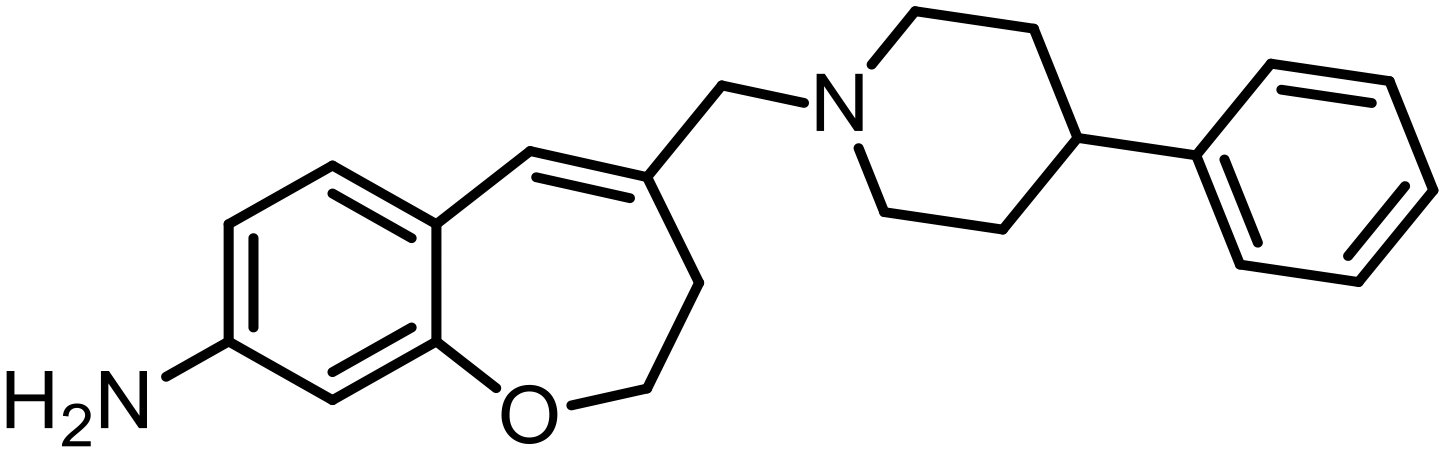
 A solution of γ-amino alcohol 12b (0.10 g, 0.284 mmol) in THF_abs._ (10 mL) and H_2_SO_4 conc_. (0.05 ml, 0.9 mmol) was stirred at rt for 24 h. After workup, the combined organic layers were dried (Na_2_SO_4_), filtered and the solvent was removed *in vacuo*. The resulting residue was purified by fc (*ø* 3 cm, *h* = 15 cm, cyclohexane : ethyl acetate 2.5 : 1 + 0.1% *N*,*N*-dimethylethylamine, *R*_f_ = 0.28 (cyclohexane : ethyl acetate 2 : 1 + 0.1% *N*,*N*-dimethylethylamine)). Yellow resin, yield 0.062 g (65%). C_22_H_26_N_2_O, *M*_r_ = 334.2. MS (EM): *m*/*z* = calcd. for C_22_H_27_N_2_O 335.2123, found 335.2150. ^1^H NMR (CDCl_3_): *δ* [ppm] = 1.62–1.81 (m, 4H, 3-H and 5-H_piperidine_), 1.86–2.02 (m, 2H, 2-H and 6-H_piperidine_), 2.42 (tt, *J* = 11.5/4.4 Hz, 1H, 4-H_piperidine_), 2.61 (t, *J* = 4.7 Hz, 2H, 3-H), 2.97–2.88 (m, 4H, 2-H and 6-H_piperidine_, and NCH_2_), 3.95 (s, NH_2_), 4.16 (t, *J* = 4.7 Hz, 2H, 2-H), 6.10 (s, 1H, 5-H), 6.21 (d, *J* = 2.4 Hz, 1H, 9-H), 6.24 (dd, *J* = 8.1/2.4 Hz, 7-H), 6.87 (d, *J* = 8.2 Hz, 1H, 6-H), 7.08–7.28 (m, 5H, C_6_H_5_). ^13^C NMR (CDCl_3_): *δ* [ppm] = 33.8 (2C, C-3 and C-5_piperidine_), 36.0 (1C, C-3), 43.1 (1C, C-4_piperidine_), 54.6 (2C, C-2 and C-6_piperidine_), 68.3 (1C, NCH_2_), 69.4 (1C, C-2), 106.1 (1C, C-9), 109.6 (1C, C-7), 117.1 (1C, C-5a), 126.3 (1C, C-4-C_6_H_5_), 126.9 (1C, C-1-C-_6_H_5_), 127.1128.6 (4C, C-2, C-3, C-5, C-6-C_6_H_5_), 134.2 (1C, C-6), 135.6 (1C, C-5), 146.5, 146.8 (2C, C-4 and C-8), 160.2 (1C, C-9a). IR (neat): *ν̃* [cm^−1^] = 3367 (m, N–H), 2796 (C–H), 2750 (C–H), 1612 (C=C). Purity (HPLC): 97.5%, *t*_R_ = 14.00 min.

#### 4-[(4-Benzylpiperidin-1-yl)methyl]-2,3-dihydro-1-benzoxepin-8-amine (18d)


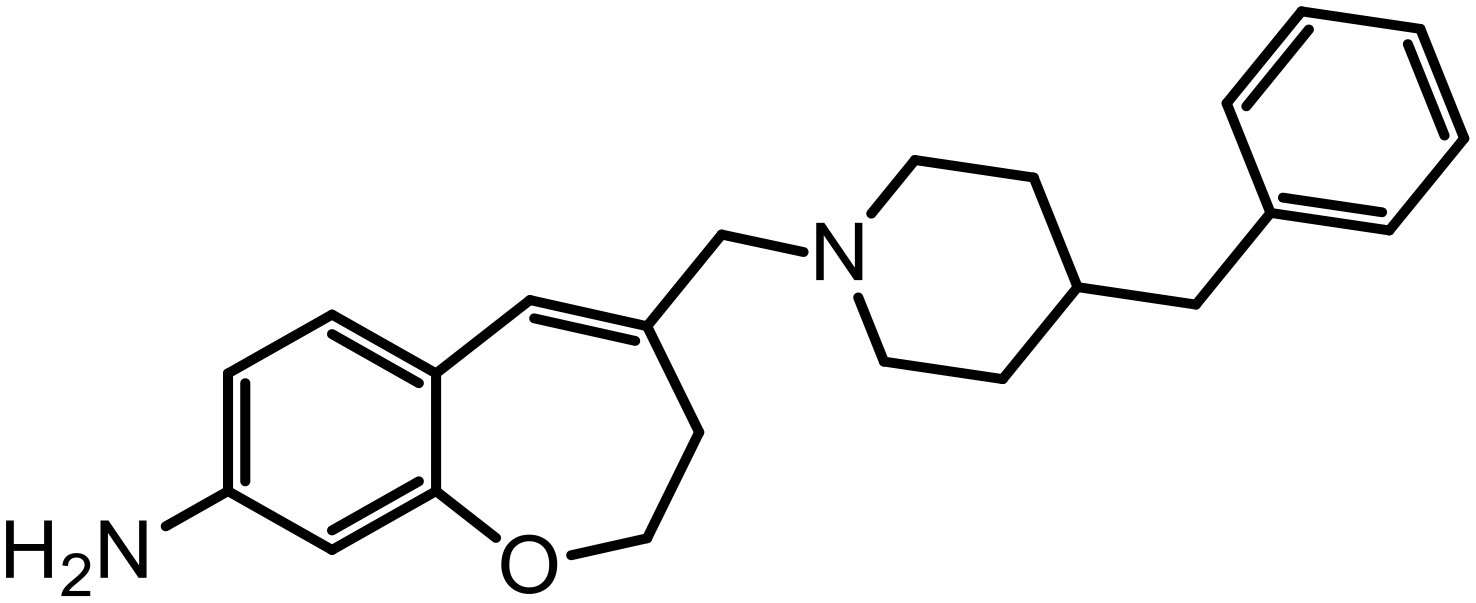
 A solution of γ-amino alcohol 12d (0.086 g, 0.24 mmol), H_2_SO_4 conc._ (0.5 mL, 9.4 mmol) and THF_abs._ was stirred at rt for 24 h. For workup a saturated aqueous NaHCO_3_ solution was added (10 mL) and the mixture was extracted with CH_2_Cl_2_. The combined organic layers were washed with brine, dried (Na_2_SO_4_), filtered and the solvent was removed under reduced pressure. The residue was purified three times by fc (*ø* 3 cm, *h* = 15 cm, cyclohexane : ethyl acetate 4 : 1 + 0.1% *N*,*N*-dimethylethylamine → 3 : 1 + 0.1% *N*,*N*-dimethylethylamine, *ø* 3 cm, *h* = 15 cm; cyclohexane : ethyl acetate 4 : 1 + 0.1% *N*,*N*-dimethylethylamine; *ø* 2 cm, *h* = 15 cm, cyclohexane : ethyl acetate 5 : 1 + 0.1% *N*,*N*-dimethylethylamine, *R*_f_ = 0.20). Yellow resin, yield 11 mg (13%). C_23_H_28_N_2_O, *M*_r_ = 348.5. MS (EM): calcd. for C_23_H_29_N_2_O 349.2280, found 349.2285. ^1^H NMR (CDCl_3_): *δ* [ppm] = 1.10–1.32 (m, 2H, 3-H, 5-H_piperidine_), 1.40–1.48 (m, 1H, 4-H_piperidine_), 1.49–1.59 (m, 2H, 3-H, 5-H_piperidine_), 1.62–1.79 (m, 2H, 2-H, 6-H_piperidine_), 2.46 (d, *J* = 7.1 Hz, 2H, C*H*_2_C_6_H_5_), 2.56 (t, *J* = 4.8 Hz, 2H, 3-H), 2.75 (d, *J* = 11.7 H, 2H, 2-H, 6-H_piperidine_), 2.88 (s, 2H, CH_2_N), 3.58 (s (broad), 2H, NH_2_), 4.12 (t, *J* = 4.3 Hz, 2H, 2-H), 6.05 (s, 1H, 5-H), 6.19 (d, *J* = 2.3 Hz, 1H, 9-H), 6.22 (dd, *J* = 8.1/2.4 Hz, 1H, 7-H), 6.84 (d, *J* = 8.1 Hz, 1H, 6-H), 7.02–7.14 (m, 3H, 2-H, 4-H, 6-H–C_6_H_5_), 7.16–7.25 (m, 2H, 3-H, 5-H–C_6_H_5_). IR (neat): *ν̃* [cm^−1^] = 3371 (N–H), 3024 (C–H_arom._), 2850 (C–H), 1616 (N–H). Purity (HPLC): 97.3%, *t*_R_ = 14.73 min.

#### *N*-{4-[(4-Phenylpiperidin-1-yl)methyl]-2,3-dihydro-1-benzoxepin-8-yl}formamide (19b)


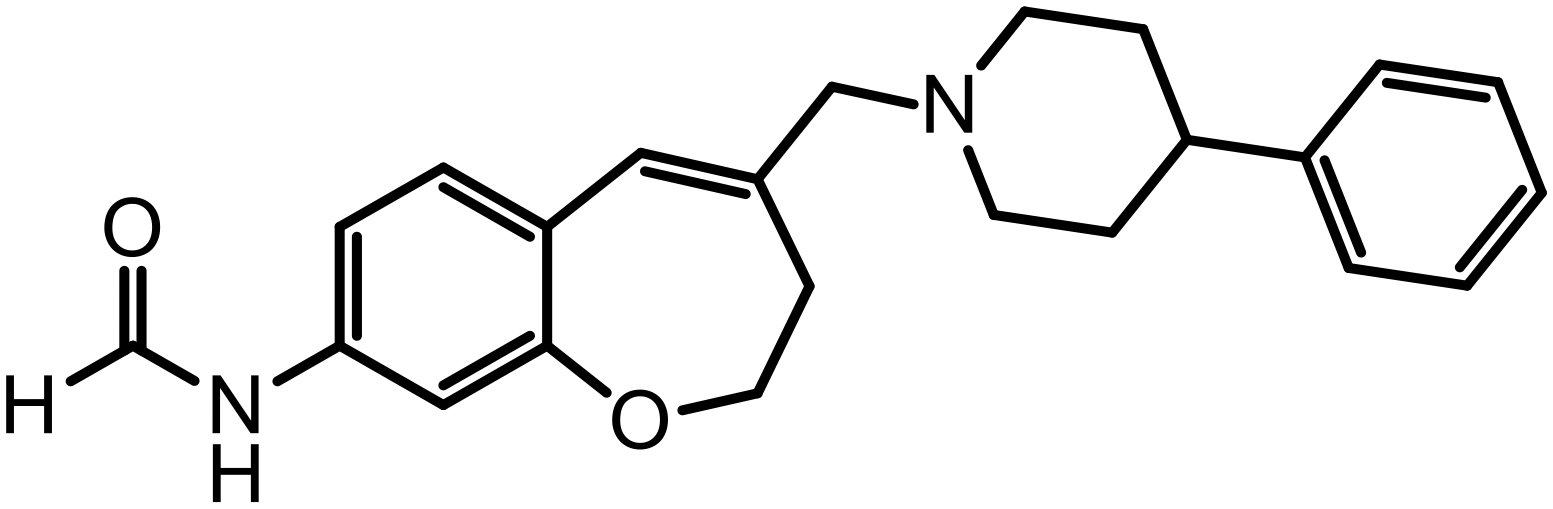
 Formic acid (0.024 g, 0.52 mmol) was slowly added to γ-amino alcohol 12b (123 mg, 0.35 mmol) and the mixture was stirred for 25 min. The reaction was terminated by the addition of water (50 mL) and NaOH (10 M, 1 mL). Ethyl acetate was added, the layers were separated and the aqueous layer was extracted with CH_2_Cl_2_. The combined organic layers were dried (Na_2_SO_4_), filtered and the solvent was removed under reduced pressure. The residue was purified by fc (*ø* 3 cm, *h* = 15 cm, *n*-hexane : ethyl acetate 2 : 1 + 0.1% *N*,*N*-dimethylethylamine, *R*_f_ = 0.25). Colorless solid, decomposition >120 °C, yield 86 mg (64%). C_23_H_26_N_2_O_2_, *M*_r_ = 362.5. MS (EM): *m*/*z* calcd. for C_23_H_27_N_2_O_2_ 363.2073, found 363.2058. MS (EI): *m*/*z* (%) = 362 (M, 21), 203 (M–4-phenylpiperidine, 8). ^1^H NMR (CDCl_3_): *δ* [ppm] = 1.63–1.81 (m, 4H, 3-H, 5-H_piperidine_), 1.91–2.01 (m, 2H, 2-H, 6-H_piperidine_), 2.37–2.50 (m, 1H, 4-H_piperidine_), 2.62–2.70 (m, 2H, 3-H), 2.92 (d, *J* = 11.3 Hz, 2H, 2-H, 6-H_piperidine_), 2.99 (s, 2H, CH_2_NR_2_), 4.14–4.22 (m, 2H, 2-H), 6.19 (s, 1H, 5-H), 6.52–6.68 (m, 1H, 7-H), 6.99 (s, 0.5H, NH), 7.02–7.08 (m, 1.5H, 9-H and 0.5 × NH), 7.11–7.30 (m, 6H, C_6_H_5_ and 6-H), 8.29 (d, *J* = 1.7 Hz, 0.5H, NHC*H*=O), 8.61 (d, *J* = 11.4 Hz, 0.5H, NHC*H*=O). Ratio of rotamers is 50 : 50. ^13^C NMR (CDCl_3_): *δ* [ppm] = 33.8, 35.9 (3C, C-3, C-5_piperidine_ and C-3), 43.0 (1C, C-4_piperidine_), 54.6 (2C, C-2, C-6_piperidine_), 68.0 (1C, CH_2_NR_2_), 69.5 (1C, C-2), 109.9 (0.5C, C-9), 111.2 (1C, C-6), 112.4 (0.5C, C-9), 113.9 (1C, C-7), 123.0 (1C, C-5a), 125.96 (1C, C-4_Ph_), 126.3 (1C, C-5), 127.1, 128.6 (4C, C-2, C-3, C-5, C-6_Ph_), 135.9 (1C, C-4), 146.7 (1C, C-1_Ph_), 158.9 (0.5C, NHCH=O), 159.4, 159.9 (2C, C-9a and C-2), 162.0 (0.5C, NHCH=O). IR (neat): *ν̃* [cm^−1^] = 2970 (C–H), 2920 (C–H), 1685 (C=O). Purity (HPLC): 99.2%, *t*_R_ = 16.93 min.

### 4.5. Receptor binding studies

#### 4.5.1. General procedures for the binding assays

The test compound solutions were prepared by dissolving approximately 10 μmol (usually 2–4 mg) of test compound in DMSO so that a 10 mM stock solution was obtained. To obtain the required test solutions for the assay, the DMSO stock solution was diluted with the respective assay buffer. The filtermats were presoaked in 0.5% aqueous polyethylenimine solution for 2 h at rt before use. All binding experiments were carried out in duplicates in the 96 well multiplates. The concentrations given are the final concentration in the assay. Generally, the assays were performed by addition of 50 μL of the respective assay buffer, 50 μL of test compound solution in various concentrations (10^−5^, 10^−6^, 10^−7^, 10^−8^, 10^−9^ and 10^−10^ mol L^−1^), 50 μL of the corresponding radioligand solution and 50 μL of the respective receptor preparation into each well of the multiplate (total volume 200 μL). The receptor preparation was always added last. During the incubation, the multiplates were shaken at a speed of 500–600 rpm at the specified temperature. Unless otherwise noted, the assays were terminated after 120 min by rapid filtration using the harvester. During the filtration, each well was washed five times with 300 μL of water. Subsequently, the filtermats were dried at 95 °C. The solid scintillator was melted on the dried filtermats at a temperature of 95 °C for 5 min. After solidifying of the scintillator at rt, the trapped radioactivity in the filtermats was measured with the scintillation analyzer. Each position on the filtermat corresponds to one well of the multiplate and was measured for 5 min with the [^3^H]-counting protocol. The overall counting efficiency was 20%. The IC_50_ values were calculated with the program GraphPad Prism® 3.0 (GraphPad Software, San Diego, CA, USA) by non-linear regression analysis. Subsequently, the IC_50_ values were transformed into *K*_i_ values using the equation of Cheng and Prusoff.^49^ The *K*_i_ values are given as mean value ± SEM from three independent experiments.

#### 4.5.2. Assay to determine the affinity towards the ifenprodil binding site of GluN2B subunit-containing NMDA receptors^30^

The competitive binding assay was performed with the radioligand [^3^H]ifenprodil (60 Ci mmol^−1^; BIOTREND, Cologne, Germany). The thawed cell membrane preparation from the transfected L(tk-) cells (about 20 μg protein) was incubated with various concentrations of test compounds, 5 nm [^3^H]ifenprodil, and TRIS/EDTA-buffer (5 mm TRIS/1 mm EDTA, pH 7.5) at 37 °C. The non-specific binding was determined with 10 μm unlabeled ifenprodil. The *K*_d_ value of ifenprodil is 7.6 nM.^30,50^

#### 4.5.3. Further radioligand receptor binding studies

The assays to determine the affinity towards σ_1_ receptors,^31–33^ σ_2_ receptors,^31–33^ and towards the PCP binding site of NMDA receptors,^34,35^ were conducted as reported in literature. Details of the receptor binding studies are given in the SI.

### 4.6. Physicochemical and *in vitro* pharmacokinetic parameters of test compounds

log *D*_7.4_ values were recorded using the micro shake flask method reported in ref. 40 and 41. Plasma protein binding was recorded by HPAC.^40,42,43^ Metabolic stability was determined by incubation with mouse liver microsomes.^40,44^ Details are given in the SI.

### 4.7. Molecular modeling

Modeling of the receptor–ligand complex was performed using a homology model of the *Homo sapiens* amino-terminal domain (ATD) of the GluN1a/GluN2B NMDA receptor. The crystal structure of the *Rattus norvegicus* GluN1a/GluN2B NMDA receptor in complex with ifenprodil (PDB ID: 4PE5),^23^ obtained from the Protein Data Bank has been selected based on sequence identity and the quality of the resolved loop regions, as determined using the RCSB Protein Data Bank sequence similarity search. A total of 100 homology models of the heterodimer-spanning residues D25–M396 of GluN1a and R23–M390 of GluN2B, were generated using MODELLER v10.7.^51^ The ligand ifenprodil was retained from the template structure, and the default ligand restraints provided by MODELLER were applied. The 10 models with the lowest atomic normalized distance-dependent statistical potential scores were selected for further evaluation. Their stereochemical quality and overall structural reliability were assessed using the MolProbity and QMEAN web servers, respectively.^52,53^ The final model was selected based on its energetic and stereochemical quality, together with the orientation of the binding-pocket residues relative to the crystal structure.

Ligand structures were prepared and protonated at pH 7.4 using Open Babel v3.0.0 (ref. 54) and protein using PROPKA3 (ref. 55) method with the CHARMM force field implemented with PDB2PQR v3.7.1.^56^ All docking experiments were performed with GOLD v2025.3.0 (ref. 57) by using default setting and CHEMPLP as primary scoring function with 150 docking iterations allowing flipping of ring corners and pyramidal nitrogen atoms. Residues within a distance of 12 Å around the piperidine nitrogen was defined as binding site. Docking poses were selected depending on the frequency of biding mode and proper geometry of protonated piperidine nitrogen. Receptor–ligand complexes were subsequently energy-minimized in MOE v2024.0601 (Molecular Operating Environment) using the AmberEHT force field. Minimization was restricted to the ligand and side chains of binding-pocket residues and was performed until an RMS gradient of 0.01 kcal mol^−1^ Å^−1^ was reached, prior to calculating protein-ligand interactions with LigandScout v4.5.^58^

## Conflicts of interest

The authors declare no conflict of interest.

## Abbreviations

AMPA: 2-Amino-3-(5-hydroxy-3-methylisoxazoly-4-yl)propionic acid
DMAP: 4-(Dimethylamino)pyridine
GABA: γ-Aminobutyric acid
HPAC: High performance affinity chromatography
5-HT: 5-Hydroxytryptamine
LC-MS: Liquid chromatography coupled with mass spectrometry
LiHMDS: Lithium hexamethyldisilazide
LTD: Long-term depression
LTP: Long-term potentiation
MD: Molecular dynamics
NADPH: Nicotinamide adenine dinucleotide phosphate hydride
NAM: Negative allosteric modulator
NMDA: *N*-Methyl-d-aspartate
NMR: Nuclear magnetic resonance
PCP: 1-(1-Phenylcyclohexyl)piperidine (phencyclidine)
PPB: Plasma protein binding
SD: Standard deviation
SEM: Standard error of the mean
THF: Tetrahydrofuran

## Supplementary Material

MD-017-D6MD00252H-s001

## Data Availability

The data supporting this article have been included as part of the supplementary information (SI). Supplementary information: the SI contains experimental procedures for receptor binding studies, log *D*_7.4_ value determination, plasma protein binding and metabolic stability as well as ^1^H and ^13^C NMR spectra of prepared compounds. See DOI: https://doi.org/10.1039/d6md00252h.
