# Supplementary material for: Negative allosteric modulation of NMDA receptors with GluN2B subunit: synthesis of γ-amino alcohols embedded in a 1-benzoxepine scaffold
Source: RSC Med Chem. 2026 Aug 4;17(9):4347–75. doi: 10.1039/d6md00252h (PMC13474310; doi:10.1039/d6md00252h)
Supplement: Supplementary file 1 [file MD-017-D6MD00252H-s001.pdf]

Supplementary Information (SI) for RSC Medicinal Chemistry.
This journal is © The Royal Society of Chemistry

Supporting information

Negative allosteric modulation of NMDA receptors with GluN2B subunit: Synthesis of □-amino alcohols embedded in a 1-benzoxepine scaffold

Felicitas Wendling,[a] Yuan Chang-Halabi,[a,b] Judith Schmidt,[a] Dirk Schepmann,[a] Julian A. Schreiber,[a] W. Felix Zhu,[a,b] Marcel Bermudez,[a,b] Bernhard Wünsch[a,b *]

a University of Münster, Institute of Pharmaceutical and Medicinal Chemistry, Corrensstraße 48, D-48149 Münster, Germany,

Tel.: +49-251-8333311; Email: wuensch@uni-muenster.de

b GRK 2515, Chemical biology of ion channels (Chembion), Universität Münster, Correnssstr. 48, D-48149 Münster, Germany

Content page

1. Receptor binding studies S2

2. Determination of $\log D_{7.4}$ values S6

3. Determination of plasma protein binding S8

4. Determination of metabolic stability *in vitro* S10

5. Molecular modelling S12

6. References S13

7. ^{1}H and ^{13}C NMR spectra of synthesized compounds S14

1. Receptor binding studies

1.1. Materials

Guinea pig brains and rat livers were commercially available (Envigo, Horst, Netherlands). Pig brains were a donation of the local slaughterhouse (Coesfeld, Germany). Homogenizers: Elvehjem Potter (B. Braun Biotech International, Melsungen, Germany) and Soniprep® 150 (MSE, London, UK). Centrifuges: Cooling centrifuge model Eppendorf 5427R (Eppendorf, Hamburg, Germany) and High-speed cooling centrifuge model Sorvall® RC-5C plus (Thermo Fisher Scientific, Langenselbold, Germany). Multiplates: standard 96 well multiplates (Diagonal, Muenster, Germany). Shaker: self-made device with adjustable temperature and tumbling speed (scientific workshop of the institute). Harvester: MicroBeta® FilterMate 96 Harvester. Filter: Printed Filtermat Typ A and B. Scintillator: Meltilex® (Typ A or B) solid state scintillator. Scintillation analyzer: MicroBeta® Trilux (all Perkin Elmer LAS, Rodgau-Jügesheim, Germany).

1.2. Cell culture and preparation of membrane homogenates from GluN2B cells

Mouse L(tk-) cells stably transfected with the dexamethasone-inducible eukaryotic expression vectors pMSG GluN1a, pMSG GluN2B (1:5 ratio) were grown in Modified Earl's Medium (MEM) containing 10% of standardized FBS Superior (Biochrom AG, Berlin, Germany). The expression of the NMDA receptor at the cell surface was induced after the cell density of the adherent growing cells had reached approximately 90% of confluency. For the induction, the original growth medium was replaced by growth medium containing 4 μM dexamethasone and 4 μM ketamine (final concentration). After 24 h, the cells were rinsed with phosphate buffered saline solution (PBS, Biochrom AG, Berlin, Germany), harvested by mechanical detachment and pelleted (10 min, 1,200 x g).

For the binding assay, the cell pellet was resuspended in PBS solution and the number of cells was determined using a Scepter® cell counter (MERCK Millipore, Darmstadt, Germany). Subsequently, the cells were lysed by sonication (4 °C, 6 × 10 s cycles with breaks of 10 s). The resulting cell fragments were centrifuged with a high performance cool centrifuge (23,500 x g, 4 °C). The supernatant was discarded and the pellet was resuspended in a defined volume of PBS yielding cell

fragments of approximately 500,000 cells/mL. The suspension of membrane homogenates was sonicated again (4 °C, 2 × 10 s cycles with a break of 10 s) and stored at -80 °C.

1.3. Preparation of membrane homogenates from guinea pig brain

5 guinea pig brains were homogenized with the potter (500-800 rpm, 10 up and down strokes) in 6 volumes of cold 0.32 M sucrose. The suspension was centrifuged at 1,200 x g for 10 min at 4 °C. The supernatant was separated and centrifuged at 23,500 x g for 20 min at 4 °C. The pellet was resuspended in 5-6 volumes of buffer (50 mM TRIS, pH 7.4) and centrifuged again at 23,500 x g (20 min, 4 °C). This procedure was repeated twice. The final pellet was resuspended in 5-6 volumes of buffer and frozen (-80 °C) in 1.5 mL portions containing about 1.5 mg protein/mL.

1.4. Preparation of membrane homogenates from rat liver

Two rat livers were cut into small pieces and homogenized with the potter (500-800 rpm, 10 up and down strokes) in 6 volumes of cold 0.32 M sucrose. The suspension was centrifuged at 1,200 x g for 10 min at 4 °C. The supernatant was separated and centrifuged at 31,000 x g for 20 min at 4 °C. The pellet was resuspended in 5-6 volumes of buffer (50 mM TRIS, pH 8.0) and incubated at rt for 30 min. After the incubation, the suspension was centrifuged again at 31,000 x g for 20 min at 4 °C. The final pellet was resuspended in 5-6 volumes of buffer and stored at -80 °C in 1.5 mL portions containing about 2 mg protein/mL.

3.5. Preparation of membrane homogenates from pig brain cortex

Fresh pig brain cortex was homogenized with the potter (500-800 rpm, 10 up and down strokes) in 6 volumes of cold 0.32 M sucrose. The suspension was centrifuged at 1,200 x g for 10 min at 4 °C. The supernatant was separated and centrifuged at 31,000 x g for 20 min at 4 °C. The pellet was resuspended in 5-6 volumes of TRIS/EDTA buffer (5 mM TRIS/1 mM EDTA, pH 7.5) and centrifuged again at 31,000 x g (20 min, 4 °C). The final pellet was resuspended in 5-6 volumes of buffer and frozen (-80 °C) in 1.5 mL portions containing about 0.8 mg protein/mL.

1.7. Protein determination

The protein concentration was determined by the method of Bradford,[1] modified by Stoscheck.[2] The Bradford solution was prepared by dissolving 5 mg of Coomassie Brilliant Blue G 250 in 2.5 mL of EtOH (95 %, v/v). 10 mL deionized H_2O and 5 mL phosphoric acid (85 %, m/v) were added to this solution, the mixture was stirred and filled to a total volume of 50 mL with deionized water. The calibration was carried out using bovine serum albumin as a standard in 9 concentrations (0.1, 0.2, 0.4, 0.6, 0.8, 1.0, 1.5, 2.0 and 4.0 mg /mL). In a 96 well standard multiplate, 10 μL of the calibration solution or 10 μL of the membrane receptor preparation were mixed with 190 μL of the Bradford solution, respectively. After 5 min, the UV absorption of the protein-dye complex at λ = 595 nm was measured with a plate reader (Tecan Genios®, Tecan, Crailsheim, Germany).

1.8. General procedures for the binding assays

The test compound solutions were prepared by dissolving approximately 10 μmol (usually 2-4 mg) of test compound in DMSO so that a 10 mM stock solution was obtained. To obtain the required test solutions for the assay, the DMSO stock solution was diluted with the respective assay buffer. The filtermats were presoaked in 0.5 % aqueous polyethylenimine solution for 2 h at rt before use. All binding experiments were carried out in duplicates in the 96 well multiplates. The concentrations given are the final concentration in the assay. Generally, the assays were performed by addition of 50 μL of the respective assay buffer, 50 μL of test compound solution in various concentrations (10^{-5}, 10^{-6}, 10^{-7}, 10^{-8}, 10^{-9} and 10^{-10} mol/L), 50 μL of the corresponding radioligand solution and 50 μL of the respective receptor preparation into each well of the multiplate (total volume 200 μL). The receptor preparation was always added last. During the incubation, the multiplates were shaken at a speed of 500-600 rpm at the specified temperature. Unless otherwise noted, the assays were terminated after 120 min by rapid filtration using the harvester. During the filtration, each well was washed five times with 300 μL of water. Subsequently, the filtermats were dried at 95 °C. The solid scintillator was melted on the dried filtermats at a temperature of 95 °C for 5 min. After solidifying of the scintillator at rt, the trapped radioactivity in the filtermats was measured with the scintillation analyzer. Each position on the filtermat corresponding to one well of the multiplate was measured for 5 min with the [^{3}H]-counting protocol. The overall counting efficiency was 20 %. The IC_{50} values

were calculated with the program GraphPad Prism® 3.0 (GraphPad Software, San Diego, CA, USA) by non-linear regression analysis. Subsequently, the IC_{50} values were transformed into K_i values using the equation of Cheng and Prusoff.[3] The K_i values are given as mean value ± SEM from three independent experiments.

1.9. Performance of the σ$_1$ receptor assay

The assays was performed with the radioligand [^{3}H]-(+)-pentazocine (22.0 Ci/mmol; Perkin Elmer). The thawed membrane preparation of guinea pig brain (about 100 μg of the protein) was incubated with various concentrations of test compounds, 2 nM [^{3}H]-(+)-pentazocine, and TRIS buffer (50 mM, pH 7.4) at 37 °C. The non-specific binding was determined with 10 μM unlabeled (+)-pentazocine. The K_d value of (+)-pentazocine is 2.9 nM.[4]

1.10. Performance of the σ$_2$ receptor assay

The assays were performed with the radioligand [^{3}H]di-*o*-tolylguanidine (specific activity 50 Ci/mmol; ARC, St. Louis, MO, USA). The thawed rat liver membrane preparation (about 100 µg protein) was incubated with various concentrations of the test compound, 3 nM [^{3}H]di-*o*-tolylguanidine and buffer containing (+)-pentazocine (500 nM (+)-pentazocine in TRIS buffer (50 mM TRIS, pH 8.0)) at rt. The non-specific binding was determined with 10 μM non-labeled di-*o*-tolylguanidine. The K_d value of di-*o*-tolylguanidine is 17.9 nM.[5]

1.11. Performance of the PCP binding assay

The assay was performed with the radioligand [^{3}H]-(+)-MK-801 (22.0 Ci/mmol; Perkin Elmer). The thawed membrane preparation of pig brain cortex (about 100 μg of the protein) was incubated with various concentrations of test compounds, 2 nM [^{3}H]-(+)-MK-801, and TRIS/EDTA buffer (5 mM TRIS/1 mM EDTA, pH 7.5) at rt. The non-specific binding was determined with 10 μM unlabeled (+)-MK-801. The K_d value of [^{3}H](+)-MK-801 is 2.26 nM.[6]

2. Determination of logD$_{7.4}$ values[7,8]

Instruments and parameters for LC-MS standard analysis (in general, if not stated otherwise)

UPLC-UV/MS (Agilent Technologies): degasser: 1260 HiP (G4225A); pump: 1260 Bin Pump (G1212B); autosampler: 1260 HiP ALS (G1367E); column oven: 1290 TCC (G1316C), 30 °C; UV/Vis detector: 1260 VWD (G1314F); MS-detector: 6120 Quadrupole LC/MS (G1978B). MS source: multimode source (G1978B); ESI mode; SIM mode (m/z given for each compound). Data acquisition and settings were performed with OpenLab CDS (ChemStation Edition, Agilent). Guard column: Zorbax Eclipse Plus-C18 (Agilent, Waldbronn, Germany) (2.1 mm x 12.5 mm, 5.0 μm particle size). Main column: Zorbax SB-C18 (Agilent, Waldbronn, Germany) (2.1 mm x 50 mm, 1.8 μm particle size). Spray chamber: vaporizer temperature: 200 °C; drying gas: 12 L/min; nebulizer pressure: 40 psi; capillary voltage: 3000 V; corona current: 4 μA; charging voltage: 2000 V; fragmentor voltage: 100 V; drying gas temperature: 250 °C. 2 mL safe lock tubes (Eppendorf), 2 mL LC-MS vials (Agilent).

LC-MS standard method (LC parameters in general, if not stated otherwise)

Eluents: solvent A: H_2O/CH_3CN 95:5 + 0.1 % formic acid; solvent B: H_2O/CH_3CN 5:95 + 0.1 % formic acid; gradient elution (A %): 0 – 2.5 min: gradient from 100 % to 0 %, 2.5 – 3.5 min: 0 %, 3.5 – 4.0 min: gradient from 0 % to 100 %, 4.0 – 8.0 min: 100 %. Change valve position: after 1.0 min the valve was switched from "waste" to "MS source". Flow rate: 0.4 mL/min. Injection volume: 1.0 μL to 100 μL (given for each compound, 1.0 μL if not stated otherwise).

Chemicals, solvents and stock solutions

3-Morpholinopropanesulfonic acid (MOPS) (Fisher Chemical, 372.5 mg, 8.9 mM) and MOPS sodium salt (Sigma-Aldrich, 513.4 mg, 11.1 mM) were dissolved in dist. H_2O (200 mL) to prepare a 20 mM buffer solution with pH 7.4. A mixture of n-octanol (Sigma Aldrich) and MOPS buffer (20 mM, pH 7.4) in the ratio 1:1 was stirred overnight at room temperature (500 rpm) to saturate both liquids with each other. Afterwards, the aqueous and organic layers were separated.

10 mM stock solutions of the test compounds in DMSO (MERCK-Schuchardt, Hohenbrunn, Germany) were prepared by dissolving an exactly weighted amount of the test compound and adding the calculated amount of DMSO. Depending on the lipophilicity, either the 10 mM stock solution was used directly or the stock solution was diluted 1:100 with MOPS buffer to a concentration of 100 μM.

General procedure

In order to determine the $logD_{7.4}$ value, the micro shake flask method was used.[1,2] To create physiological conditions a buffer with pH 7.4 was used to analyze the lipophilicity ($logD_{7.4}$). The $logD_{7.4}$ value was determined by using three different volume ratios of buffer and n-octanol (1:1, 2:1, 1:2).

Standard procedure: The 10 mM DMSO stock solution of the test compound (7.5 μL) was added to three different volumes of MOPS buffer (750 μL, 1000 μL, 500 μL) in 2 mL Eppendorf tubes. Afterwards, the tubes were filled up to 1500 μL with n-octanol (750 μL, 500 μL, 1000 μL). Each ratio was produced as a triplicate. The tubes were vortexed at rt and centrifuged at 4 °C with 16,000 rpm for 2 min.

An aliquot of the aqueous layer was analyzed by LC-MS standard method. For matrix-matched calibration to calculate $logD_{7.4}$ value, the samples were diluted with MOPS buffer within a range of 1.56 nM to 1.0 μM or 39 nM to 10 μM. All samples were measured once.

3. Determination of plasma protein binding[9,10]

Instruments and parameters for LC-MS PPB analysis (in general, if not stated otherwise)

UPLC-UV/MS (Agilent Technologies): degasser: 1260 HiP (G4225A); pump: 1260 Bin Pump (G1212B); autosampler: 1260 HiP ALS (G1367E); column oven: 1290 TCC (G1316C), 30 °C; UV/Vis detector: 1260 VWD (G1314F); MS-detector: 6120 Quadrupole LC/MS (G1978B). MS source: multimode source (G1978B); ESI mode; SIM mode (*m*/*z* given for each compound). Data acquisition and settings were performed with OpenLab CDS (ChemStation Edition, Agilent). Guard column: Chiralpak® HSA HPLC guard column (2.0 x 10 mm, 5.0 μm particle size). Main column: Chiralpak® HSA HPLC column (Daicel, Eschborn, Germany) (2.0 x 50 mm, 5.0 μm particle size). Spray chamber: vaporizer temperature: 200 °C; drying gas: 12 L/min; nebulizer pressure: 40 psi; capillary voltage: 3000 V; corona current: 4 µA; charging voltage: 2000 V; fragmentor voltage: 100 V; drying gas temperature: 250 °C. 2 mL safe lock tubes (Eppendorf), 2 mL LC-MS vials (Agilent).

LC-MS PPB method (LC parameters in general, if not stated otherwise)

Eluents: solvent A: NH_4OAc buffer (50 mM, pH 7.4); solvent B: *iso*propanol; isocratic elution (A %): 96 %. Flow rate: 0.3 mL/min. Stop time: 3.0 min (*D*-glucose, metronidazole, paracetamol, ramipril, salbutamol, sulfamethoxazole), 8.0 min (propranolol), 10 min (phenytoin), 15 min (haloperidol, imipramine), 60 min (chlorpromazine, test compounds). Injection volume: 1.0 µL to 100 µL (given for each compound, 1.0 µL if not stated otherwise).

Chemicals, solvents and stock solutions

The pH value of an aqueous solution of NH_4OAc (50 mM, Acros Organics, Schwerte, Germany) was adjusted to pH 7.4 with NH_3. 2.0 mM solutions of the reference and the test compounds were prepared from the 10 mM DMSO stock solutions by diluting 1:5 with DMSO (MERCK-Schuchardt, Hohenbrunn, Germany). The final concentration of 20 µM was prepared by further dilution 1:100 with eluent NH_4OAc (50 mM, pH 7.4) : *iso*propanol = 96:4.

Method PA (general procedure)

In order to determine the PPB, a high performance affinity chromatography (HPAC) was performed. The dead time was determined with *D*-glucose. The following compounds with known plasma protein binding were used as reference compounds: metronidazole, paracetamol, salbutamol, sulfamethoxazole, ramipril, propranolol, phenytoin, haloperidol, imipramine and chlorpromazine. The retention times of these compounds and the test compound were measured three times by LC-MS PPB method in SIM mode.

4. Determination of metabolic stability *in vitro*[7,11]

Preparation of mouse liver microsomes

Frozen livers (-80 °C) from male C57BL/6 mice were received from *Prof. Dr. Martina Düfer* from the Institute of Pharmaceutical and Medicinal Chemistry (WWU Münster).

At first, the frozen livers were warmed up at 37 °C for a few min and washed with 1.15 % (*m*/*v*) KCl solution at 4 °C. After cutting the livers into small pieces, the livers were homogenized in an Elvehjem-Potter (10 strokes, 3 s, 800 rpm) with cold phosphate buffer (pH 7.4, 0.1 M, 1.0 mL PBS/g liver) containing sodium EDTA (0.5 mM). PBS (pH 7.4, 0.1 M, 3.0 mL PBS/g liver), cooled on ice, was added and the resulting suspension was centrifuged at 9,000 g for 20 min at 4 °C. The supernatant was centrifuged again at 40,000 g for 90 min at 4 °C. The obtaining microsome pellet was dissolved in PBS (pH 7.4, 0.1 M). Aliquots of 1.0 mL were filled in safe lock Eppendorf tubes and stored at -80 °C.

Instruments and parameters for LC-MS standard analysis (in general, if not stated otherwise)

UPLC-UV/MS (Agilent Technologies): degasser: 1260 HiP (G4225A); pump: 1260 Bin Pump (G1212B); autosampler: 1260 HiP ALS (G1367E); column oven: 1290 TCC (G1316C), 30 °C; UV/Vis detector: 1260 VWD (G1314F); MS-detector: 6120 Quadrupole LC/MS (G1978B). MS source: multimode source (G1978B); ESI mode; SIM mode (*m*/*z* given for each compound). Data acquisition and settings were performed with OpenLab CDS (ChemStation Edition, Agilent). Guard column: Zorbax Eclipse Plus-C18 (Agilent, Waldbronn, Germany) (2.1 mm x 12.5 mm, 5.0 μm particle size). Main column: Zorbax SB-C18 (Agilent, Waldbronn, Germany) (2.1 mm x 50 mm, 1.8 μm particle size). Spray chamber: vaporizer temperature: 200 °C; drying gas: 12 L/min; nebulizer pressure: 40 psi; capillary voltage: 3000 V; corona current: 4 μA; charging voltage: 2000 V; fragmentor voltage: 100 V; drying gas temperature: 250 °C. 2 mL safe lock tubes (Eppendorf), 2 mL LC-MS vials (Agilent).

LC-MS standard method (LC parameters in general, if not stated otherwise)

Eluents: solvent A: H_2O/CH_3CN 95:5 + 0.1 % formic acid; solvent B: H_2O/CH_3CN 5:95 + 0.1 % formic acid; gradient elution (A %): 0 – 2.5 min: gradient from 100 % to 0 %, 2.5 – 3.5 min: 0 %,

3.5 – 4.0 min: gradient from 0 % to 100 %, 4.0 – 8.0 min: 100 %. Change valve position: after 1.0 min the valve was switched from ”waste“ to ”MS source“. Flow rate: 0.4 mL/min. Injection volume: 1.0 μL to 100 μL (given for each compound, 1.0 μL if not stated otherwise).

Chemicals, solvents and stock solutions

NADPH Na_4 (Carl Roth, Karlsruhe, Germany) and UDPGA Na_3 (Sigma Aldrich) were dissolved in phosphate buffer (PBS, 0.1 M, pH 7.4, Sigma Aldrich, Darmstadt, Germany) to prepare a 2.0 mg/mL solution, respectively. $MgCl_2$ (Honeywell Specialty Chemicals, Seelze, Germany) was dissolved in bidist. H_2O to a 0.05 M solution. 1.0 mM solutions of the test compounds were prepared from the 10 mM DMSO stock solutions by diluting 1:10 with DMSO (MERCK-Schuchardt, Hohenbrunn, Germany).

Phase I Metabolism

NADPHNa$_4$ (2.0 mg/mL in 0.1 M PBS, 50 μL), $MgCl_2$ (0.05 M in H_2O, 50 μL) and phosphate buffer (PBS, 0.1 M, 76.8 μL) were mixed in an Eppendorf tube. The test compound (1.0 mM in DMSO, 1.2 μL) and mouse liver microsomes (MLM, 22 μL) were added. Instead of test compound, imipramine (1.0 mM in DMSO, 1.2 μL) was incubated with mouse liver microsomes as positive control. The metabolic stability of imipramine using this procedure is well known (20 % of parent compound after 90 min incubation). The prepared samples were incubated at 37 °C for 90 min at 900 rpm at the thermomixer (Eppendorf). The incubation was stopped by adding CH_3CN/CH_3OH 1:1 (400 μL) to the samples and ice-cooling for 10 min to precipitate the proteins. The samples were centrifuged at 4 °C for 15 min at 16,000 rpm. An aliquot of the supernatant was measured by LC-MS standard method. An “empty sample” (without test compound, PBS was added to replace the missing volume) was prepared in the same way. Additionally, “blanks” (without NADPH Na_4, PBS was added to replace the missing volume) were prepared according to the same procedure. The test compound (10 mM in DMSO, 1.2 μL) was added after precipitating the proteins under ice-cooling for 10 min.

5. Molecular modelling

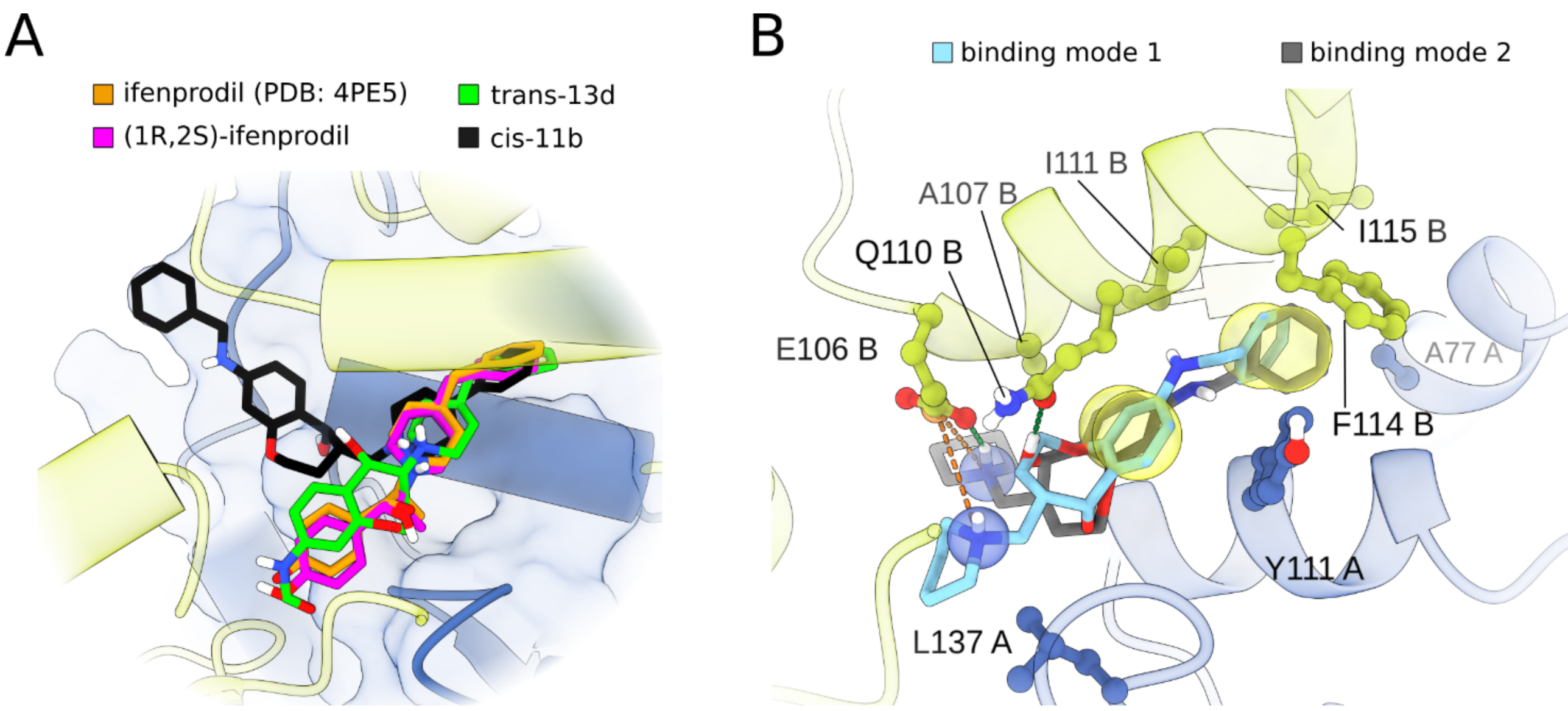

Figure S1. Comparison of binding modes derived by molecular docking.

6. References

[1] Bradford, M. M. A rapid and sensitive method for the quantitation of microgram quantities of protein utilizing the principle of protein-dye binding, *Anal. Biochem.* **1976,** *72*, 248–254.

[2] Stoscheck, C. Quantification of protein, *Methods Enzymol.* **1990,** *182*, 50–68.

[3] Cheng, Y.-C.; Prusoff, W. H. Relationship between the inhibition constant (KI) and the concentration of inhibitor which causes 50 per cent inhibition (I50) of an enzymatic reaction, *Biochem. Pharmacol.* **1973,** *22*, 3099–3108.

[4] DeHaven-Hudkins, D. L.; Fleissner, L. C.; Ford-Rice, F. Y. Characterization of the binding of [3H](+)-pentazocine to σ recognition sites in guinea pig brain, *Eur. J. Pharmacol. Mol. Pharmacol.* **1992,** *227*, 371–378.

[5] Mach, R. H.; Smith, C. R.; Childers, S. R. Ibogaine possesses a selective affinity for σ2 receptors, *Life Sci.* **1995,** *57*, PL57–PL62.

[6] Utech, T., PhD thesis, University of Freiburg 2003.

[7] F. Börgel, F. Galla, K. Lehmkuhl, D. Schepmann, S. M. Ametamey, B. Wünsch. Pharmacokinetic properties of enantiomerically pure GluN2B selective NMDA receptor antagonists with 3-benzazepine scaffold. *J. Pharm. Biomed. Anal. A* **2019**, *172*, 214 - 222.

[8] F. Galla, C. Bourgeois, K. Lehmkuhl, D. Schepmann, M. Soeberdt, T. Lotts, C. Abels, S. Ständer, B. Wünsch. Effects of polar κ receptor agonists designed for the periphery on ATP-induced Ca^{2+} release from keratinocytes. *MedChemComm* **2016**, *7*, 317 - 326. DOI 10.1039/C5MD00414D.

[9] V. Butsch, F. Börgel, F. Galla, K. Schwegmann, S. Hermann, M. Schäfers, B. Riemann, B. Wünsch, S. Wagner. Design, (Radio)Synthesis, and in Vitro and in Vivo evaluation of highly selective and potent matrix metalloproteinase 12 (MMP-12) inhibitors as radiotracers for positron emission tomography. *J. Med. Chem.* **2018**, *61*, 4115 - 4134. DOI: 10.1021/acs.jmedchem.8b00200.

[10] C. P. Konken, K. Heßling, I. Thale, S. Schelhaas, J. Dabel, S. Maskri, E. Bulk, T. Budde, O. Koch, A. Schwab, M. Schäfers, B. Wünsch, Imaging of the calcium activated potassium channel 3.1 ($K_{Ca}3.1$) *in vivo* using a senicapoc-derived PET tracer. *Arch. Pharm.* **2022**, e2200388. DOI: 10.1002/ardp.202200388.

[11] C. Wiese, E. Große Maestrup, F. Galla, D. Schepmann, A. Hiller, S. Fischer, F.-A. Ludwig, W. Deuther-Conrad, C. K. Donat, P. Brust, L. Büter, U. Karst, B. Wünsch. Comparison of in

silico, electrochemical, in vitro and in vivo metabolism of a homologous series of (radio)fluorinated□□$_1$ receptor ligands designed for positron emission tomography. *ChemMedChem* **2016**, *11*, 2445 - 2458. DOI 10.1002/cmdc.201600366.

7. ^{1}H and ^{13}C NMR spectra of synthesized compounds

^{1}H and ^{13}C NMR spectrum (DMSO-D_6) of ester **5**

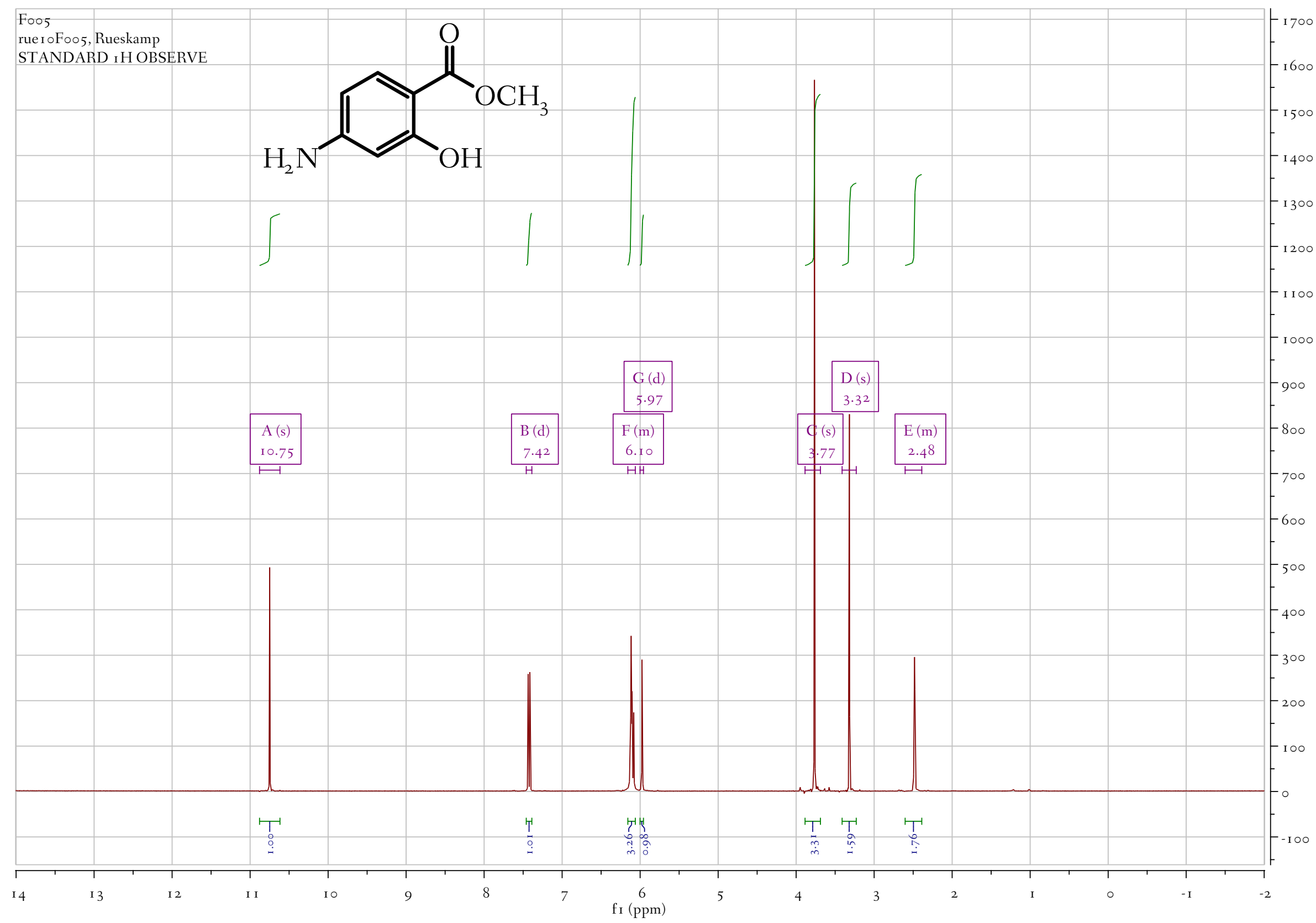

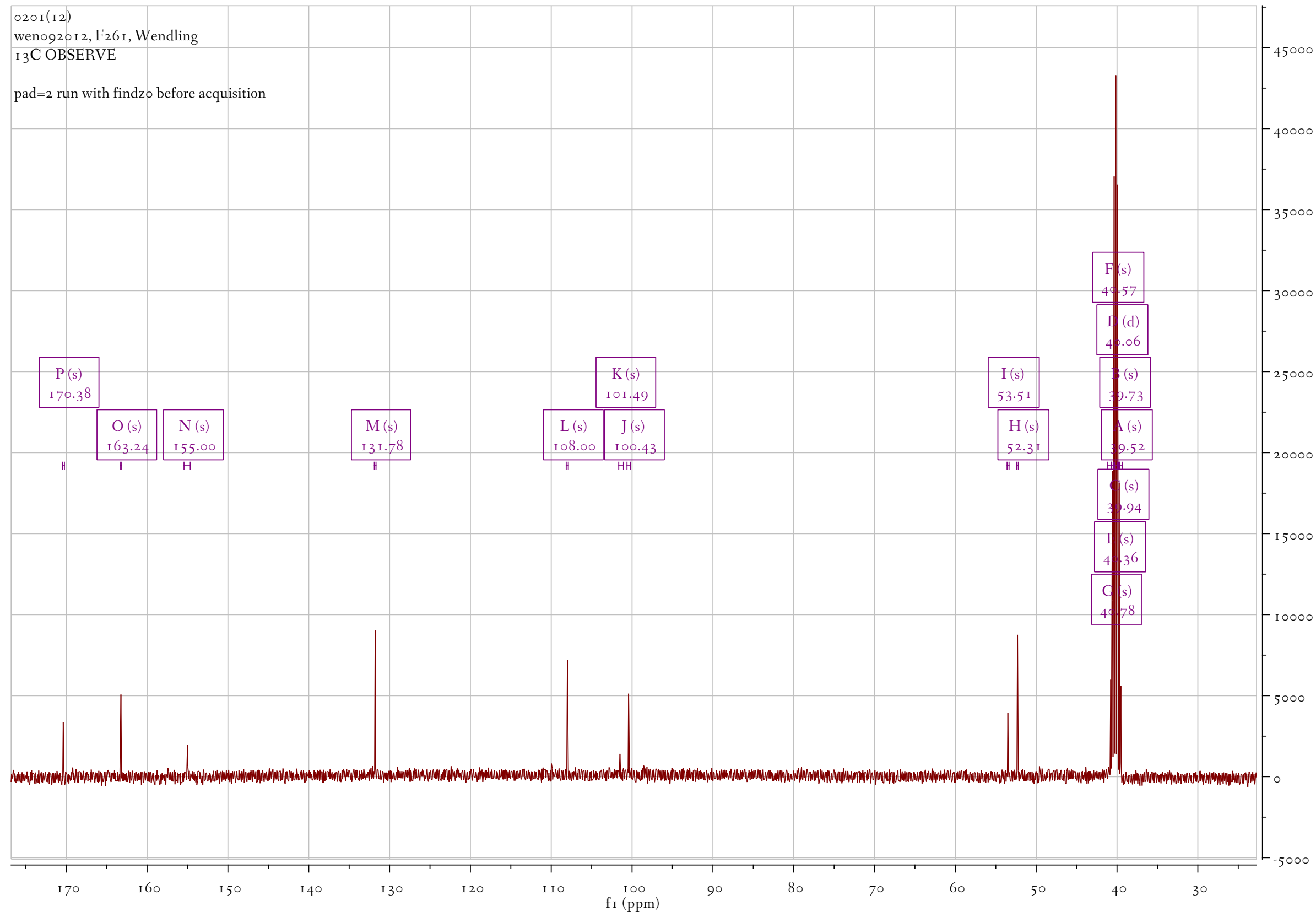

¹H NMR spectrum (DMSO-D$_6$) and ¹³C NMR spectrum (CDCl$_3$) of benzamide **6**

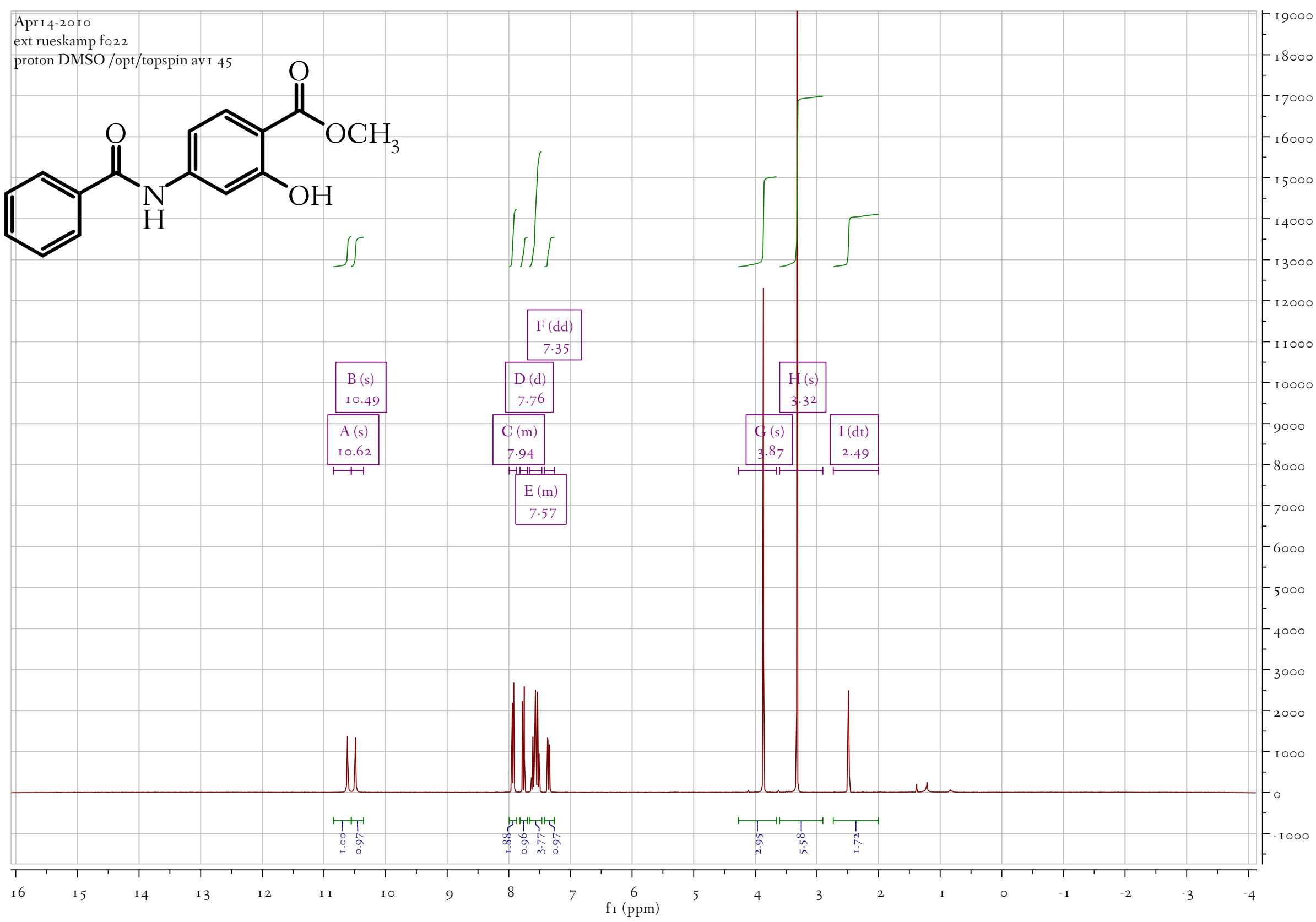

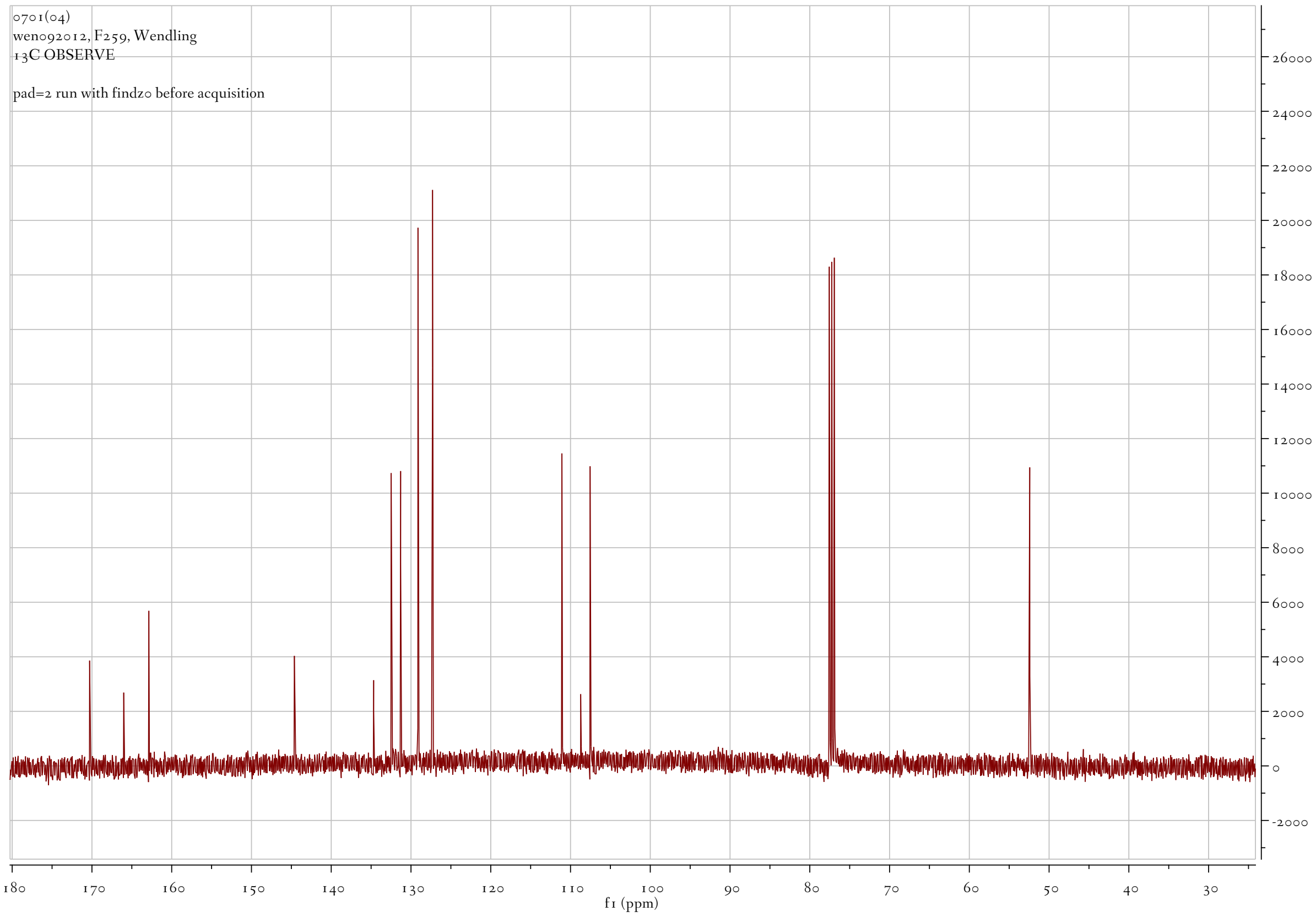

^{1}H and ^{13}C NMR spectrum ($CDCl_3$) of diester **7**

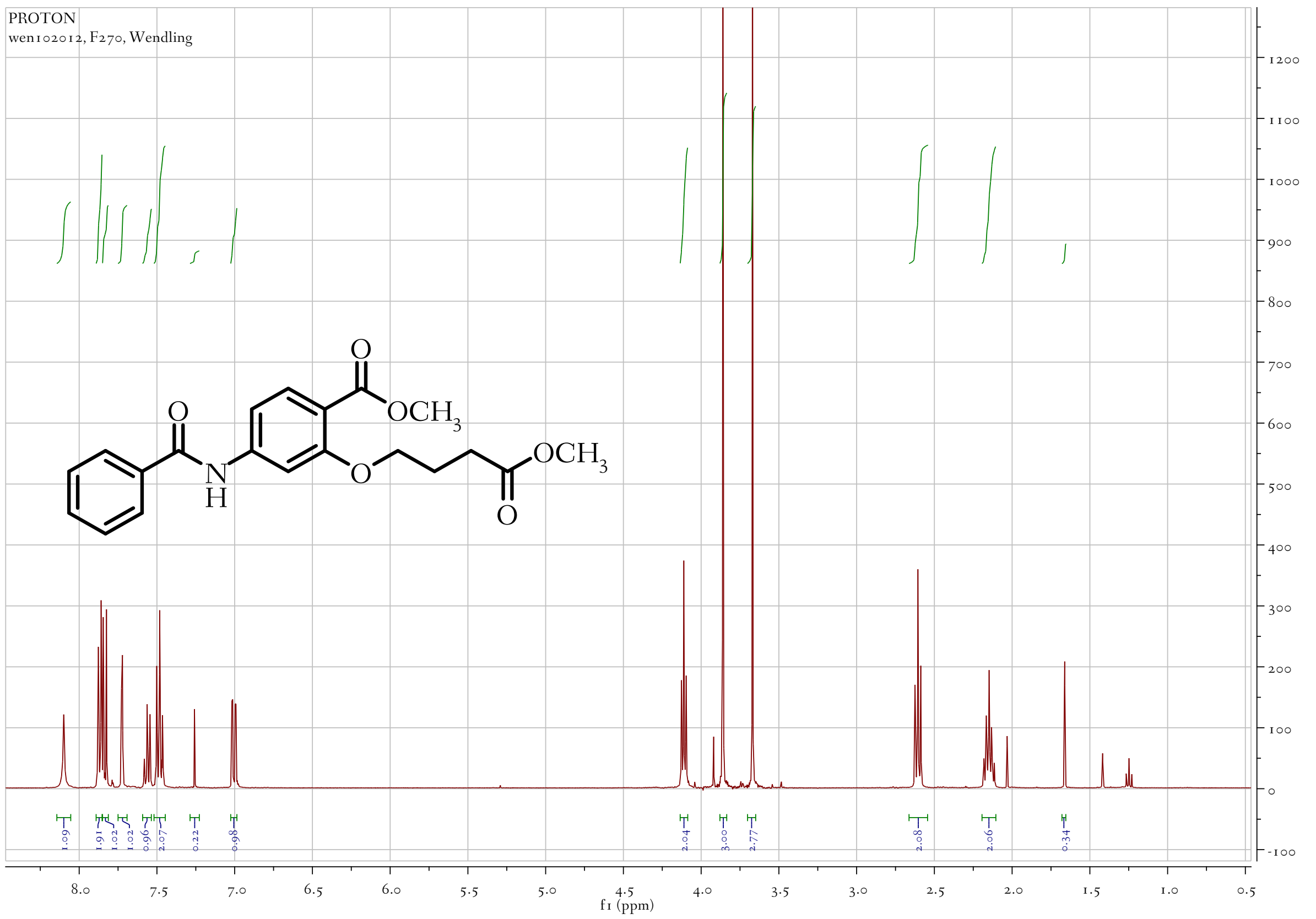

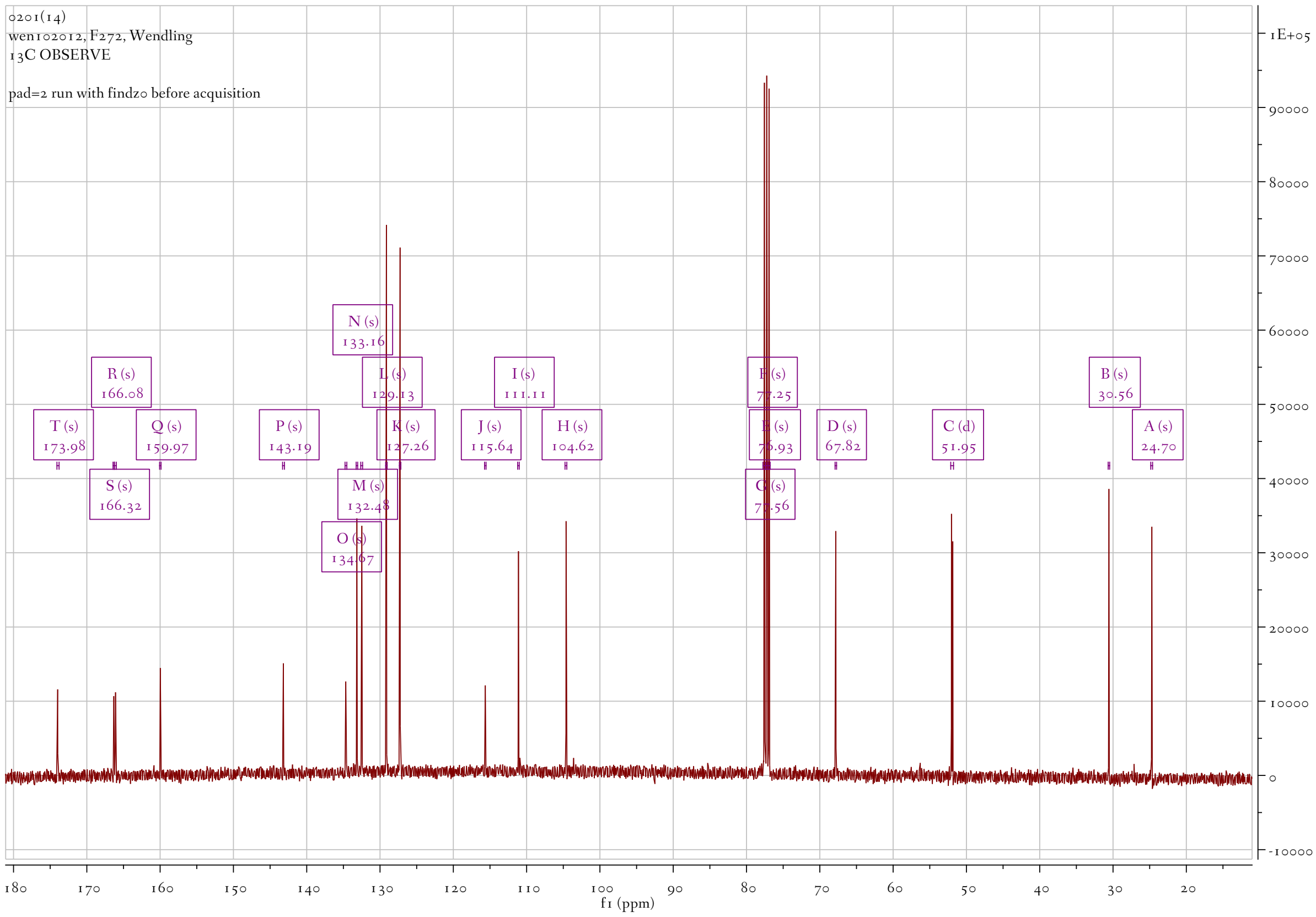

^1H and ^{13}C NMR spectrum ($CDCl_3$) of 1-benzoxepine **8**

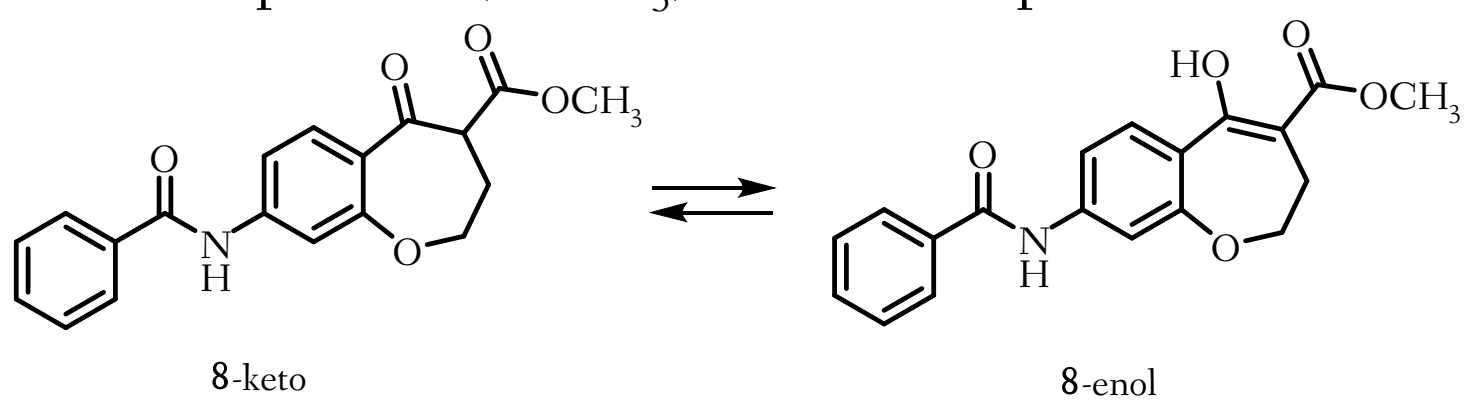

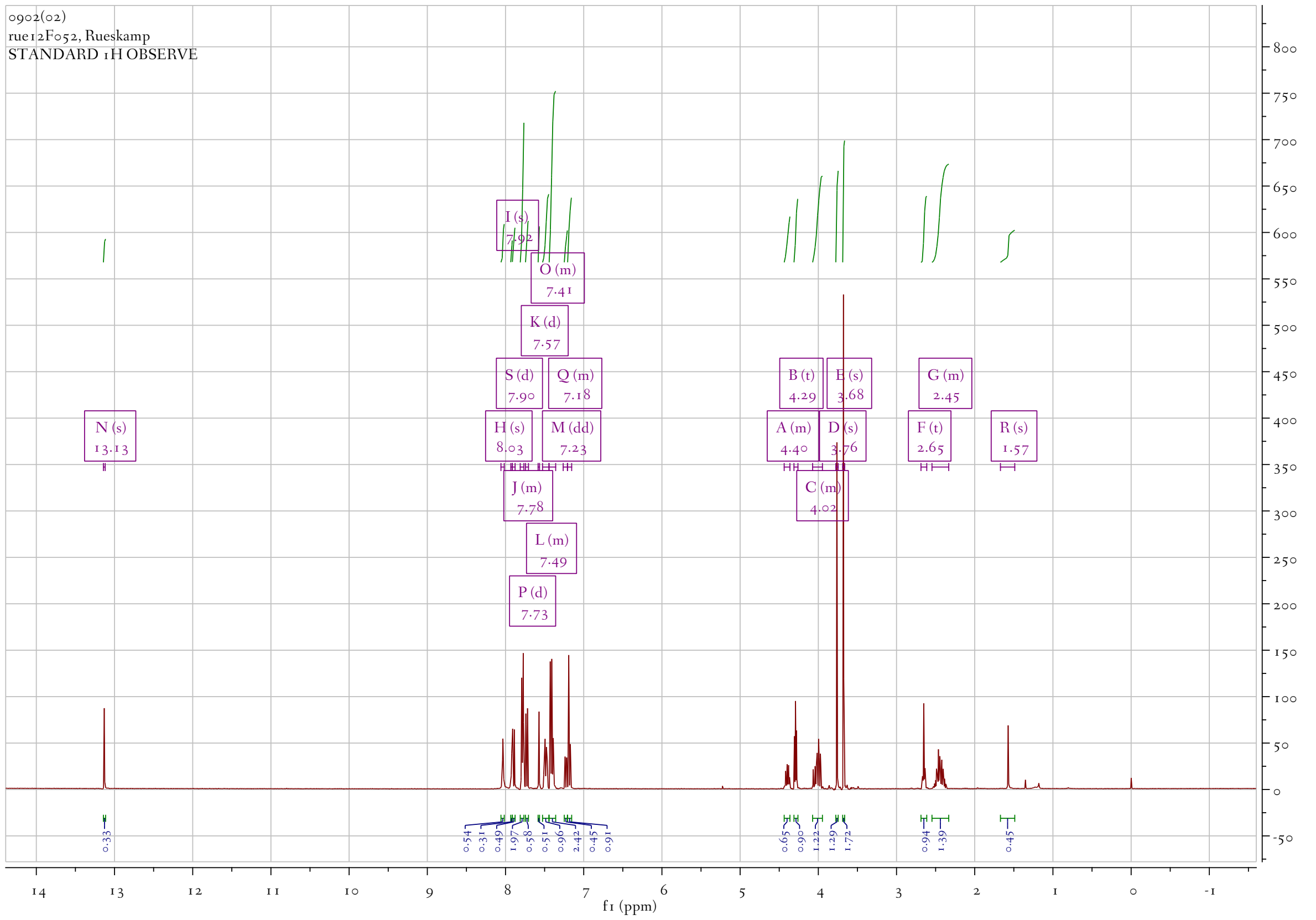

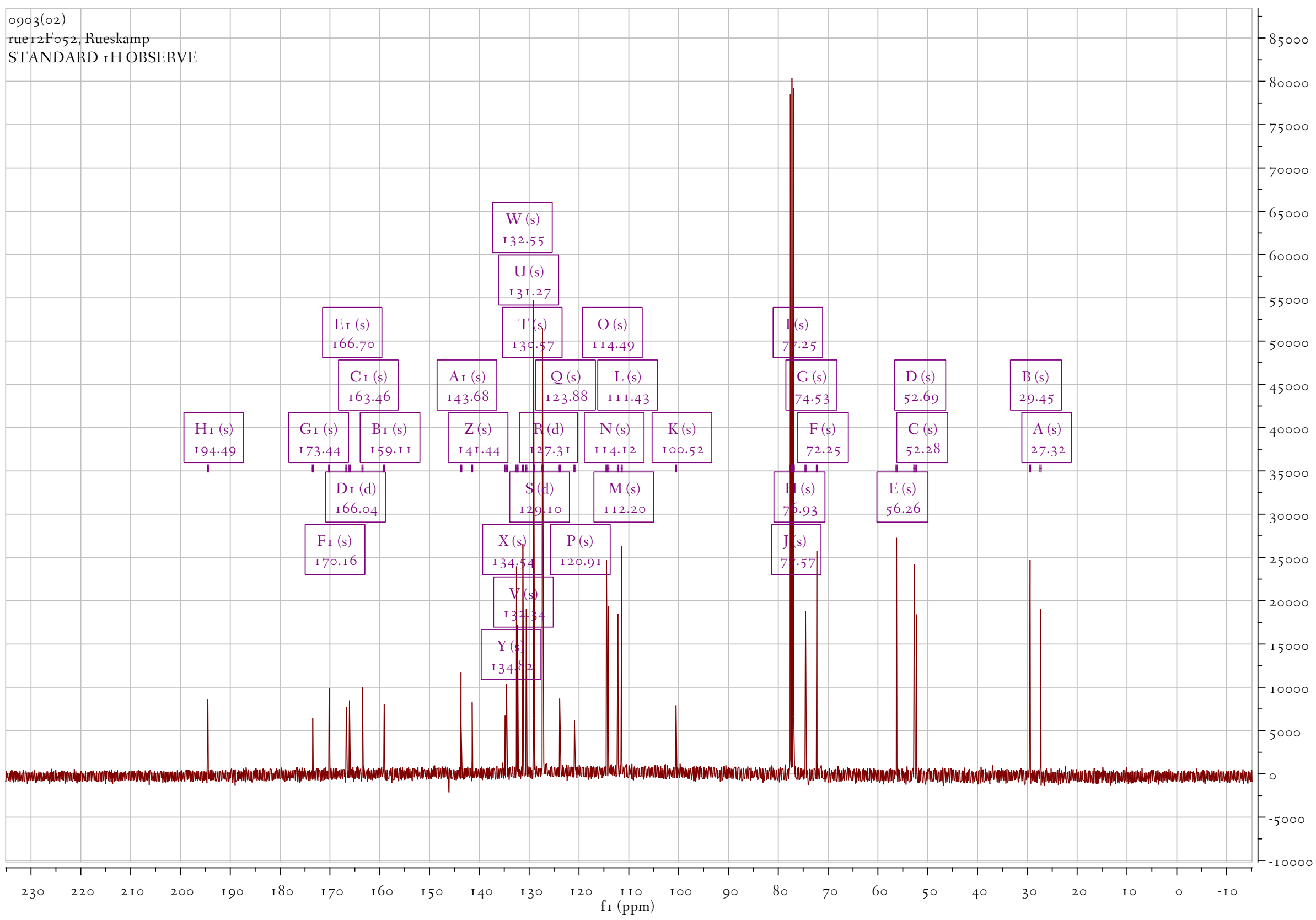

^{1}H and ^{13}C NMR spectrum ($CDCl_3$) of amide **9a**

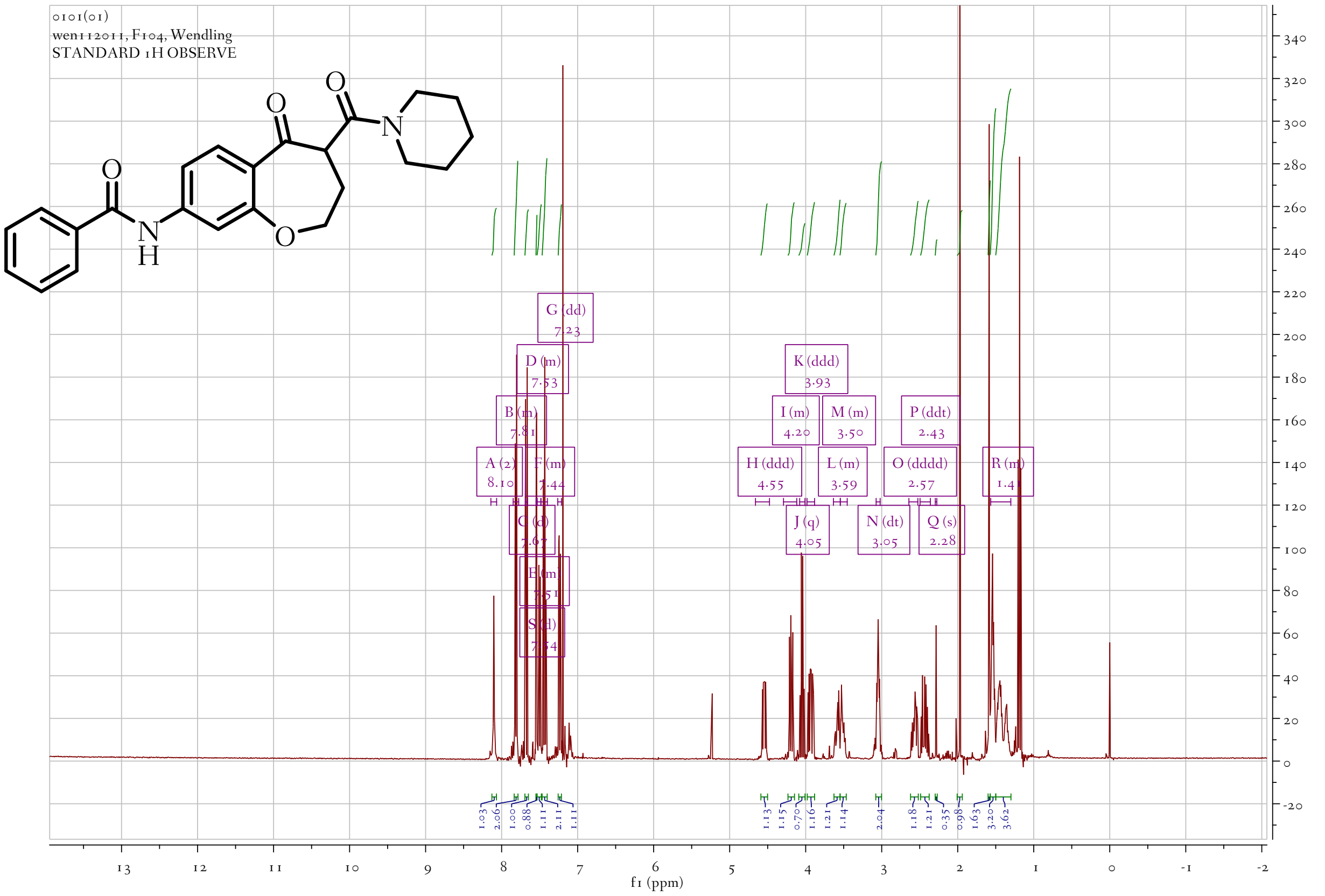

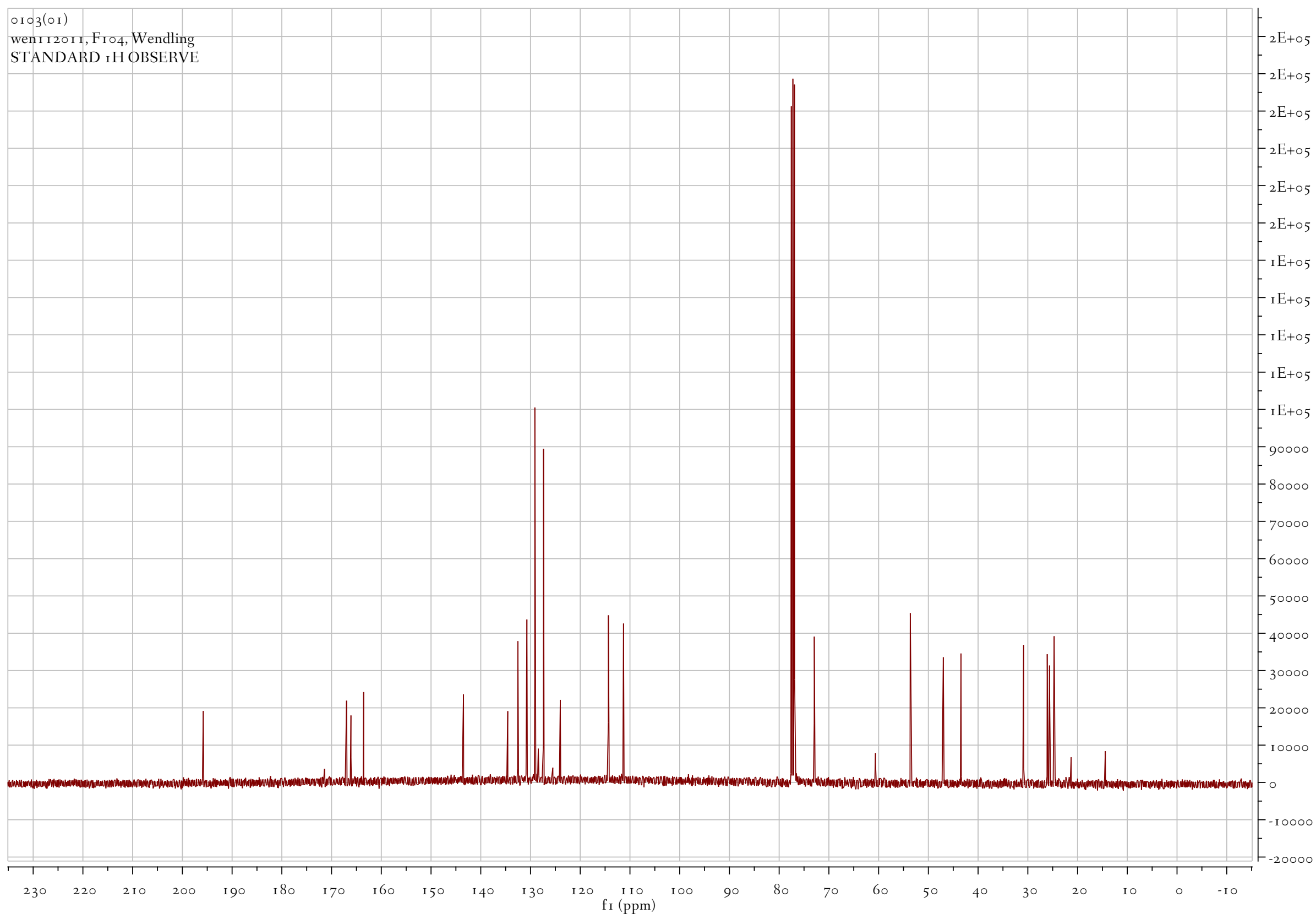

^{1}H and ^{13}C NMR spectrum ($CDCl_3$) of amide **9b**

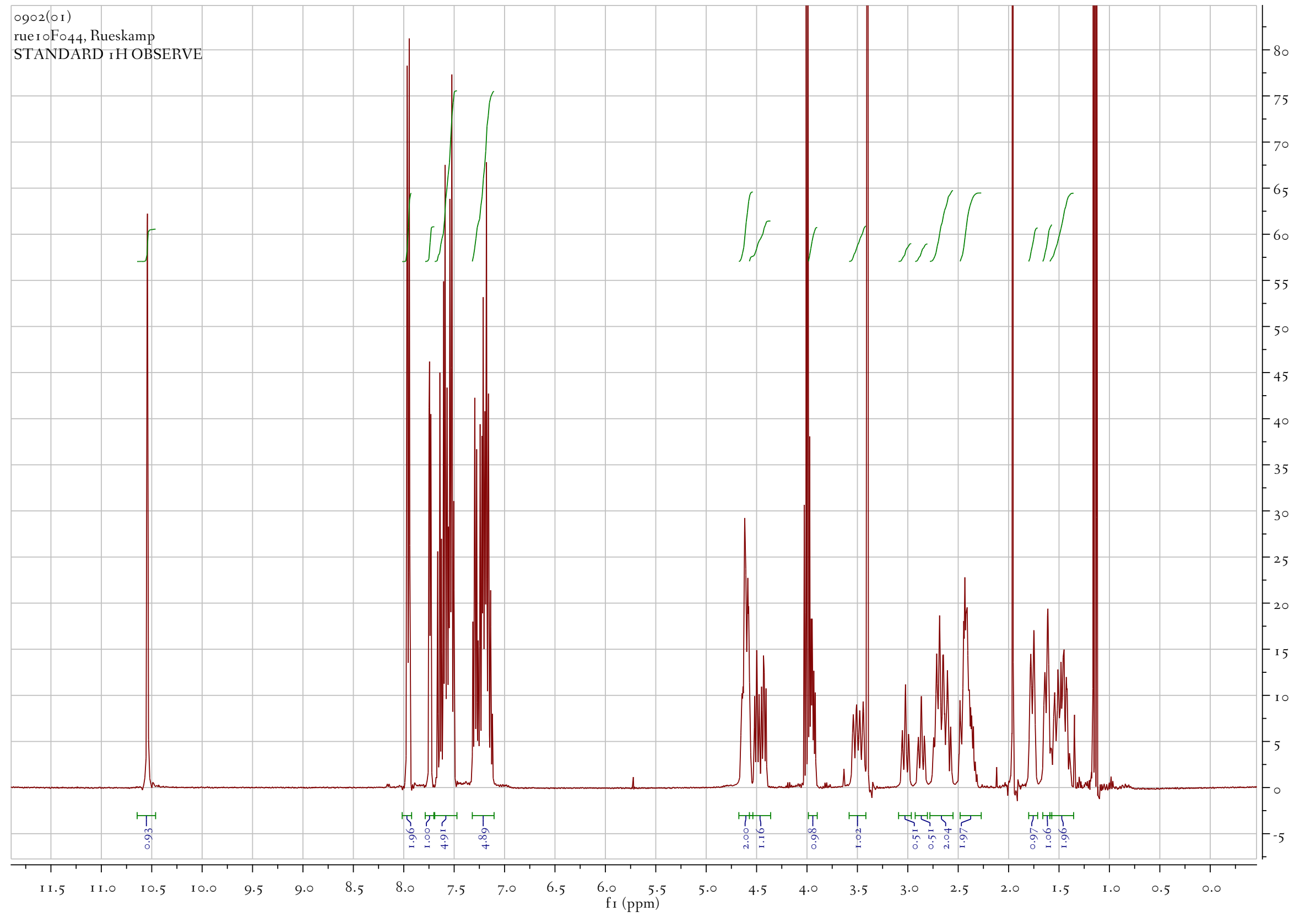

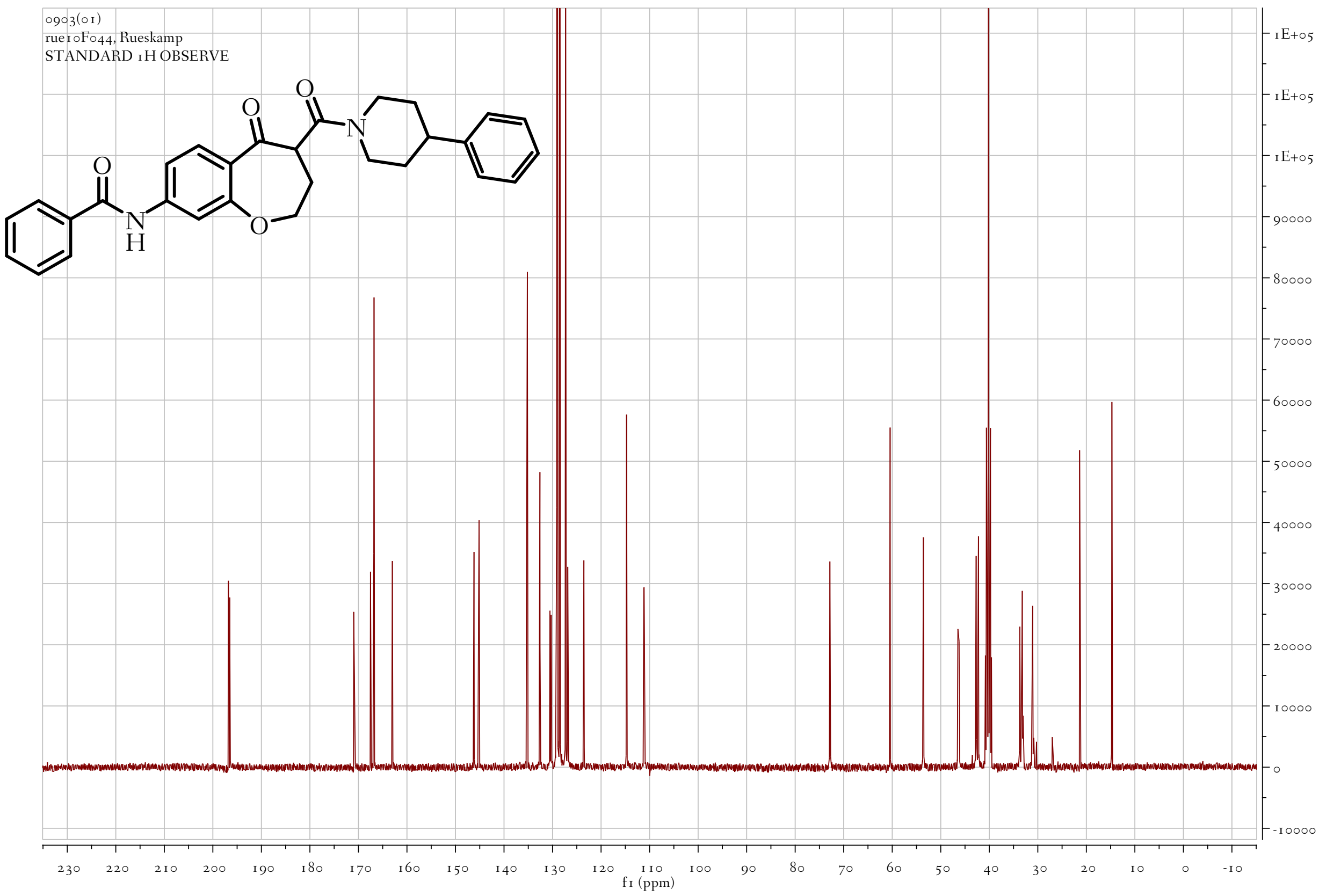

^{1}H and ^{13}C NMR spectrum ($CDCl_3$) of amide **9c**

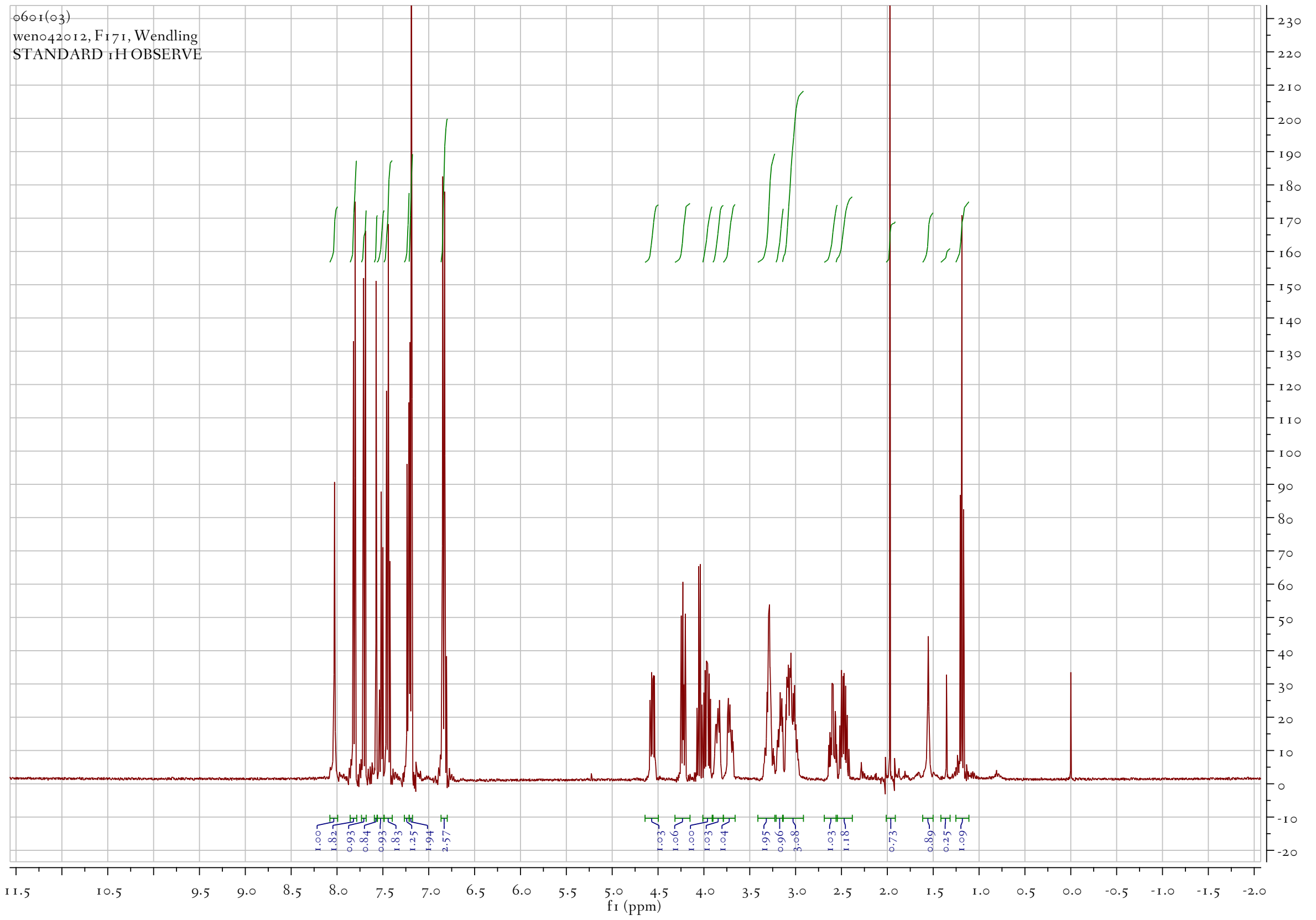

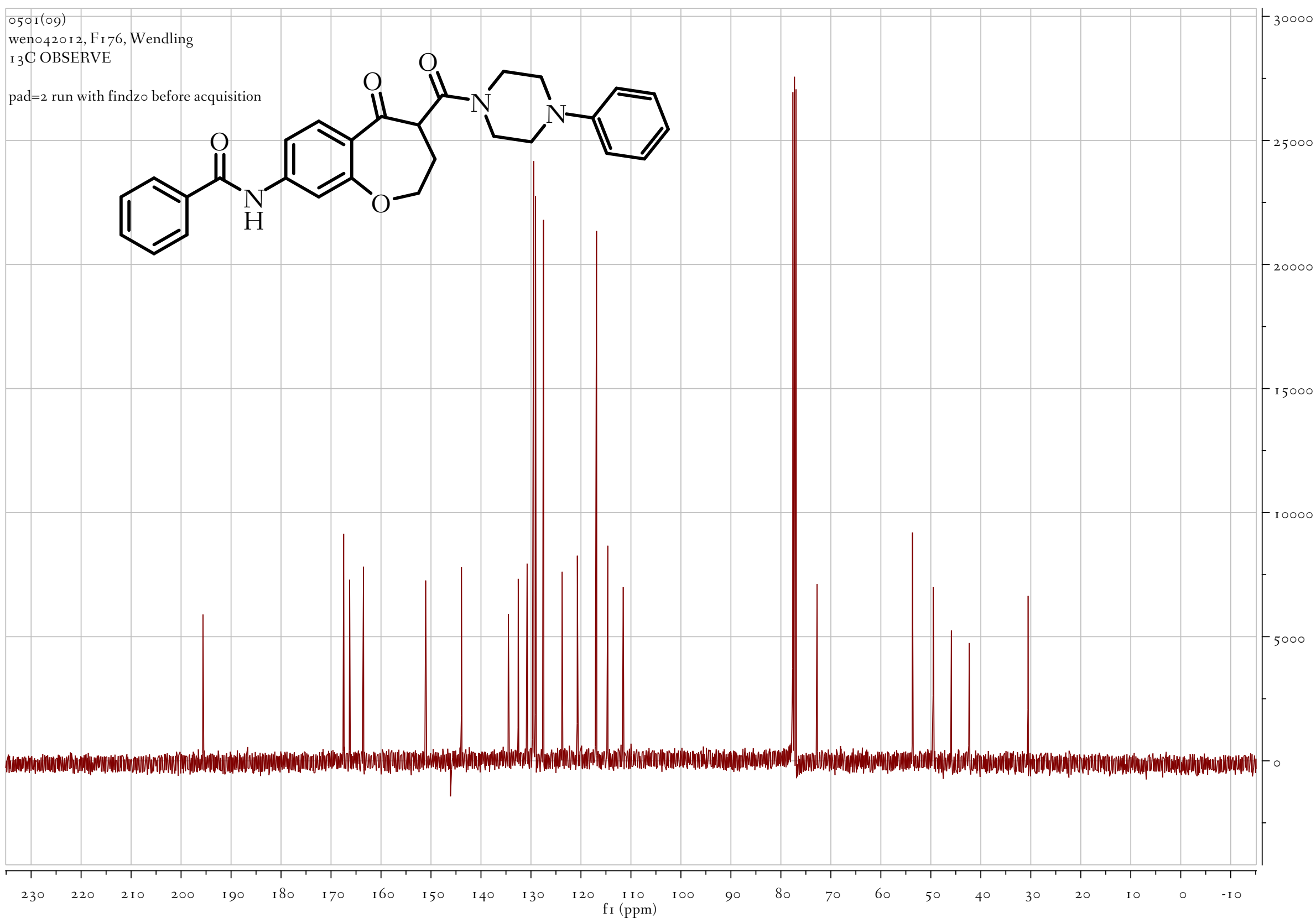

^{1}H and ^{13}C NMR spectrum ($CDCl_3$) of amide **9d**

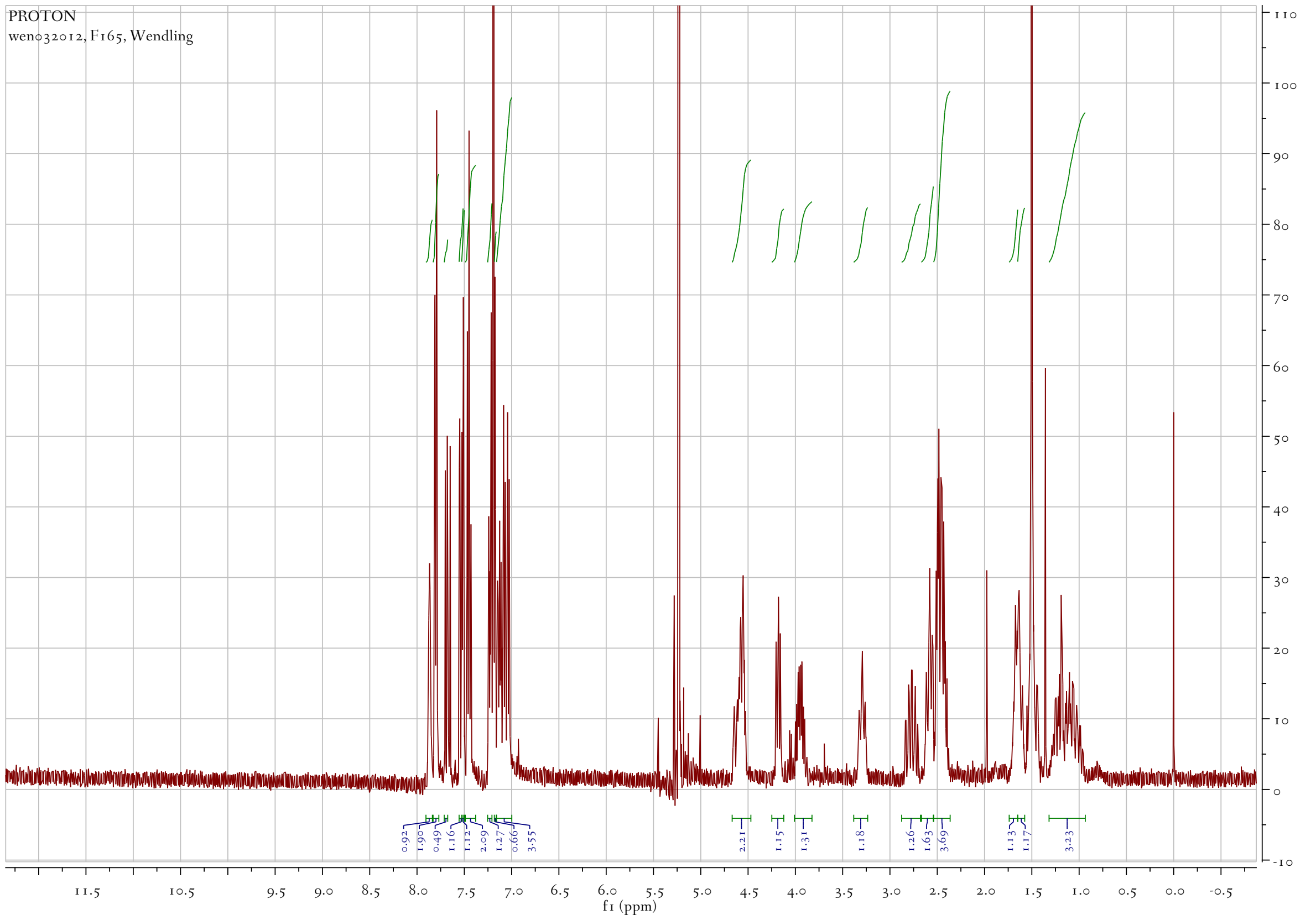

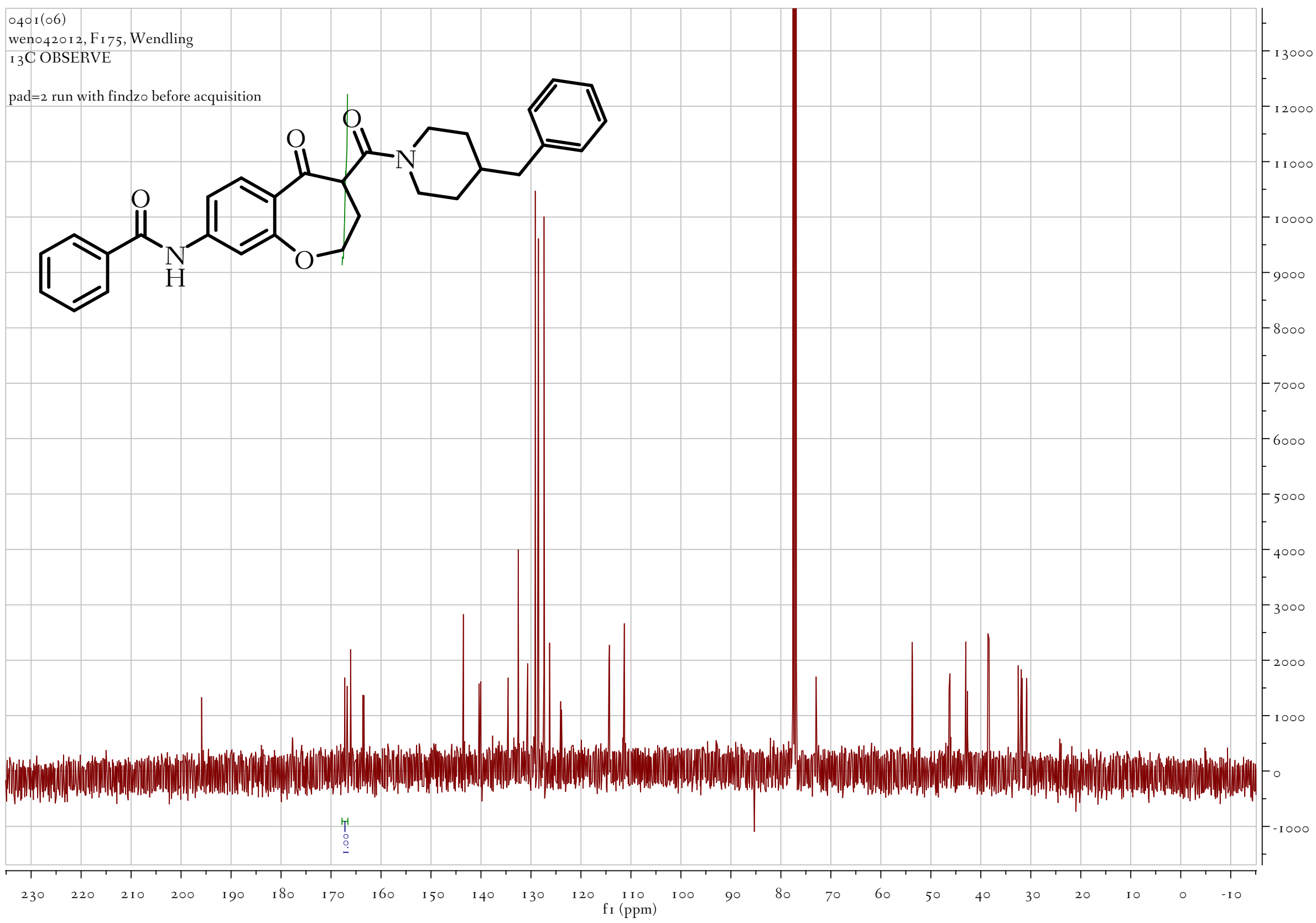

^{1}H and ^{13}C NMR spectrum ($CDCl_3$) of diethylamide **9e**

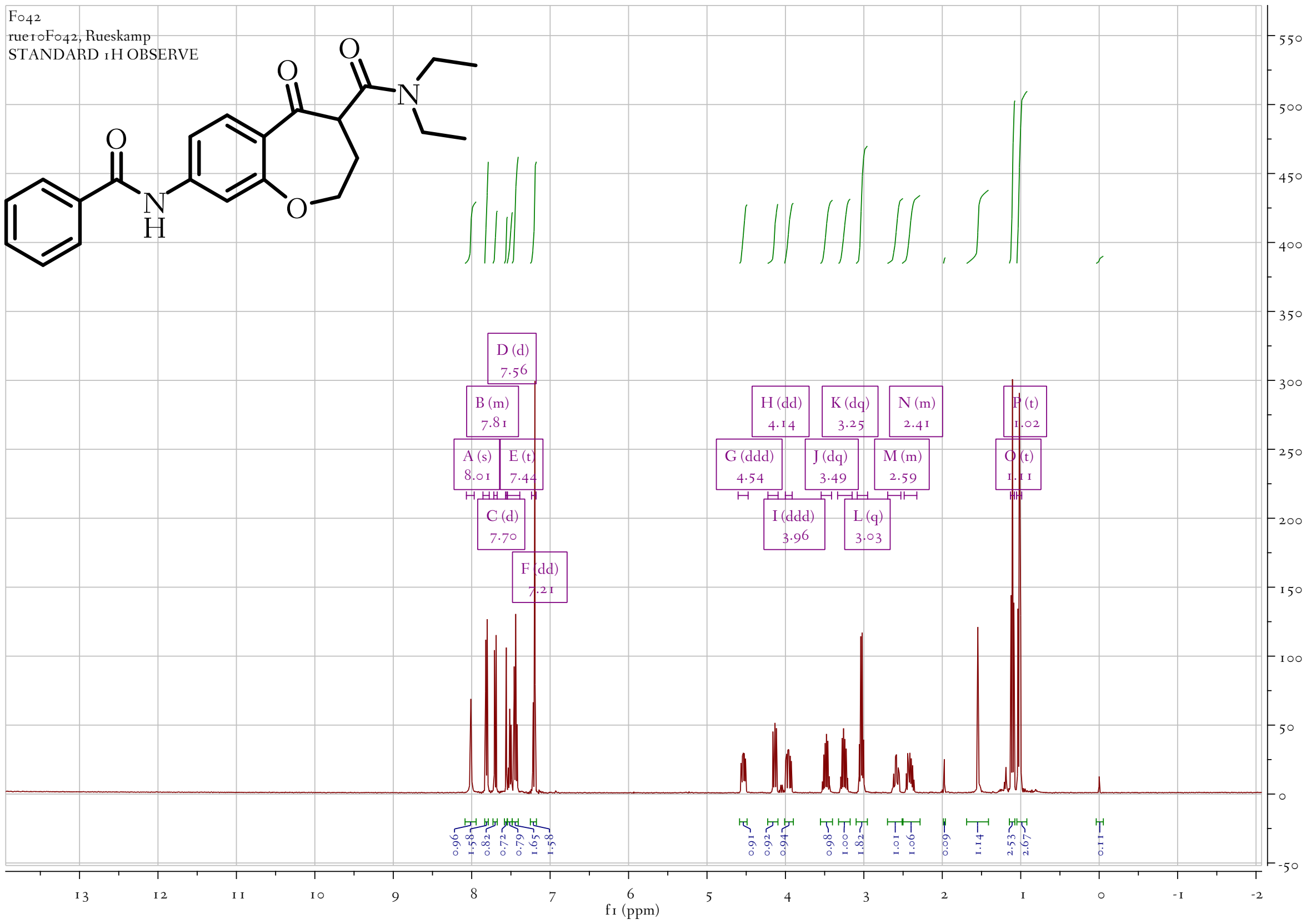

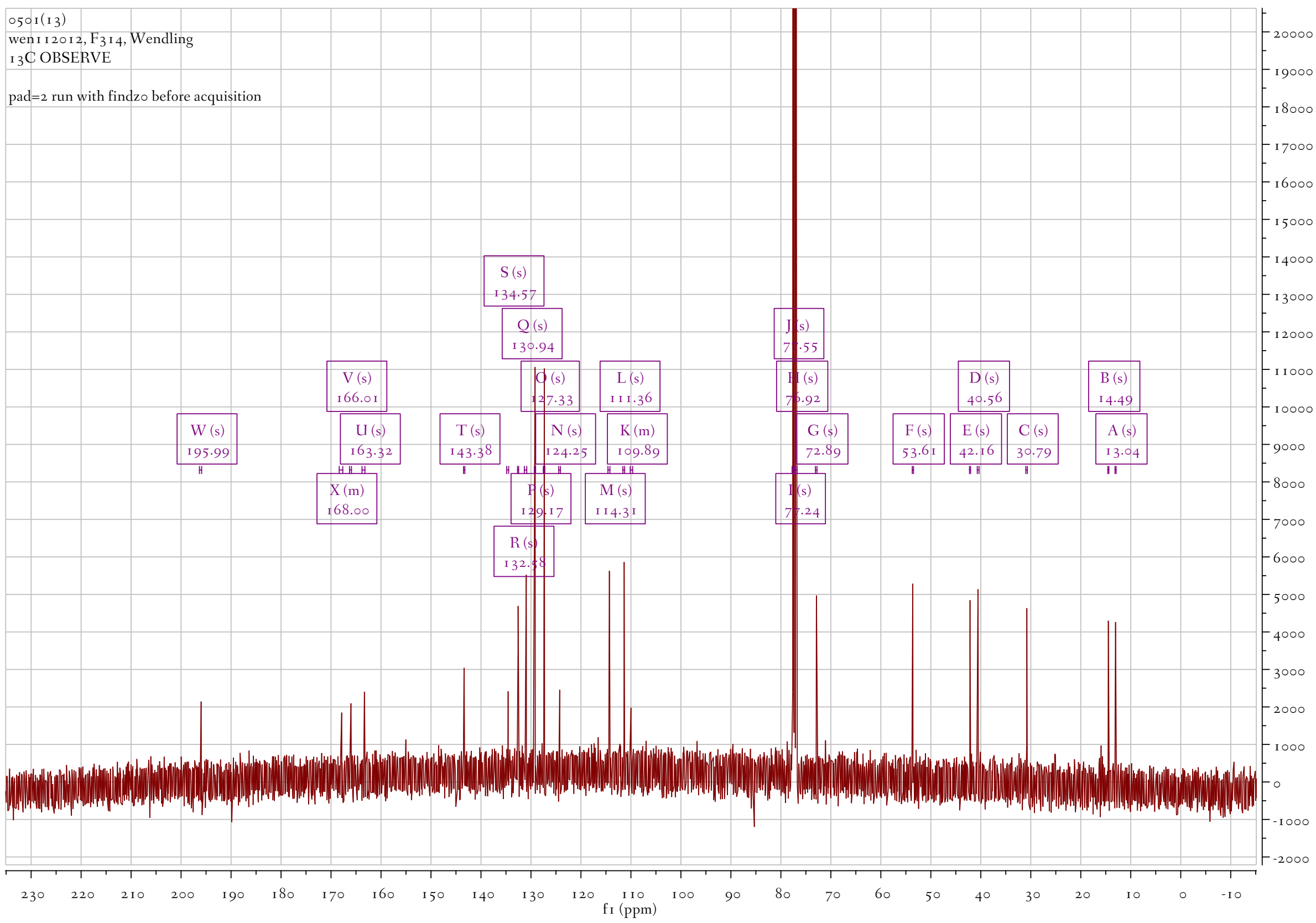

¹H and ¹³C NMR spectrum ($CDCl_3$) of b-hydroxy amide **10a**

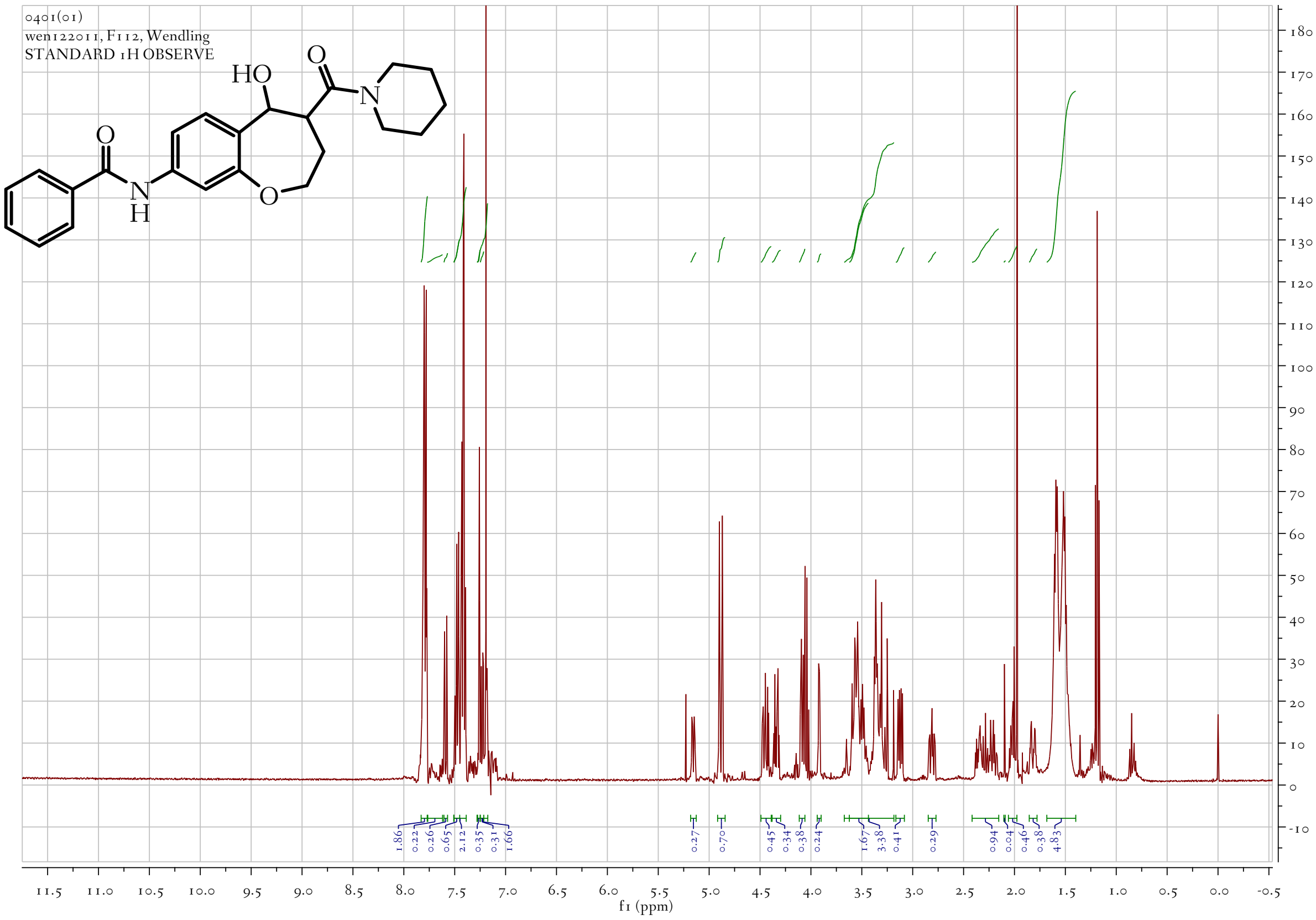

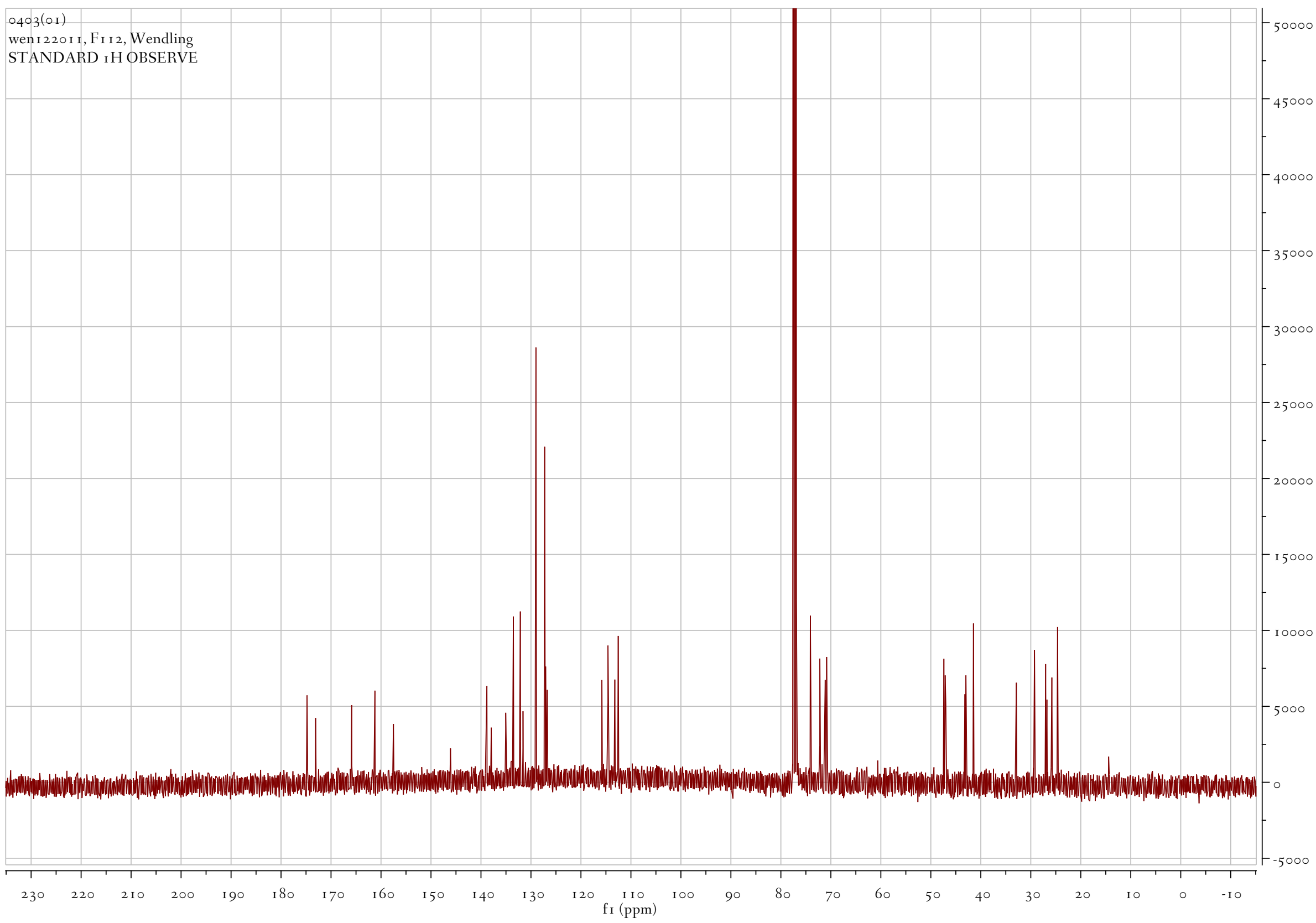

^{1}H and ^{13}C NMR spectrum ($CDCl_3$) of b-hydroxy amide *cis*-**10b**

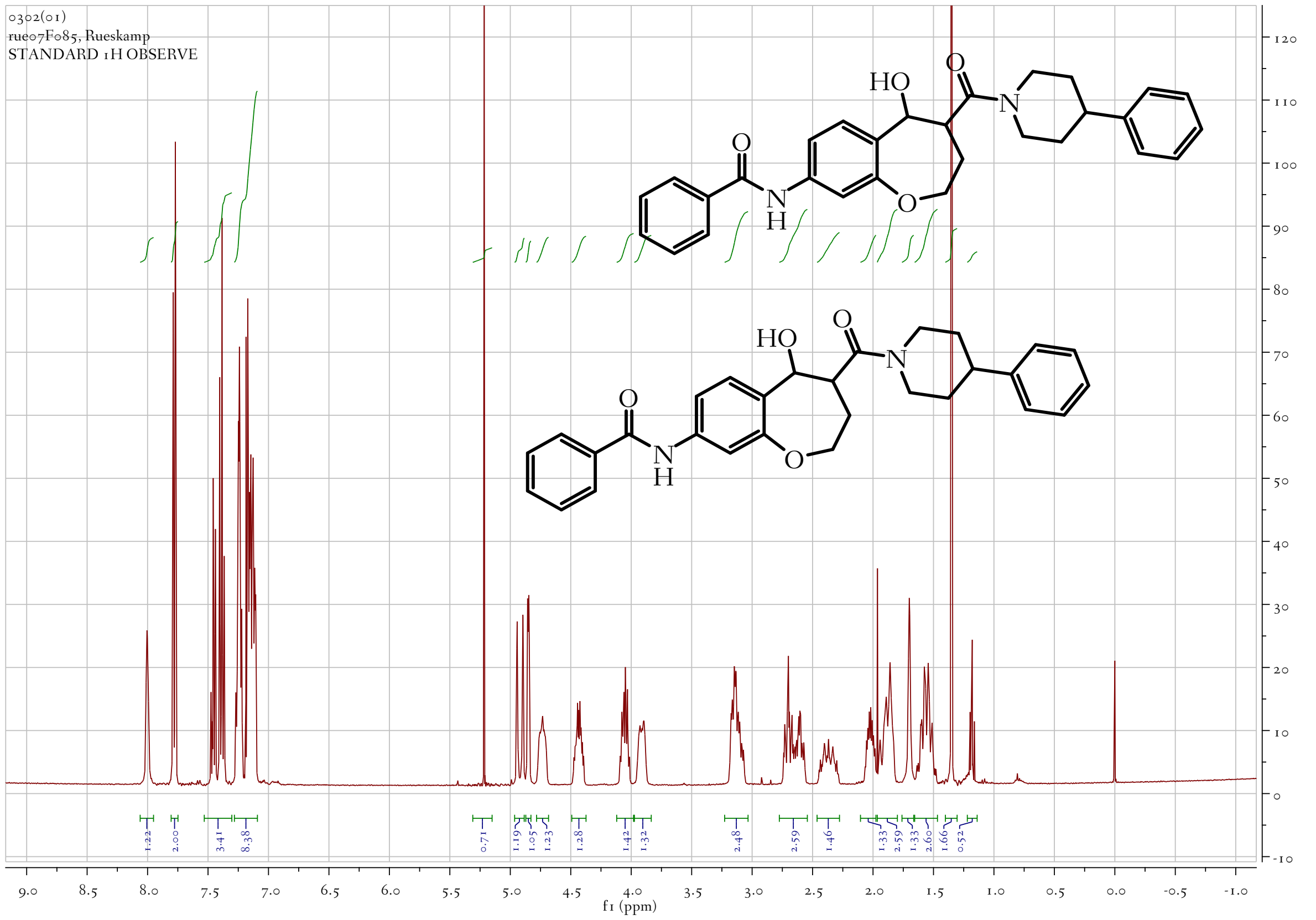

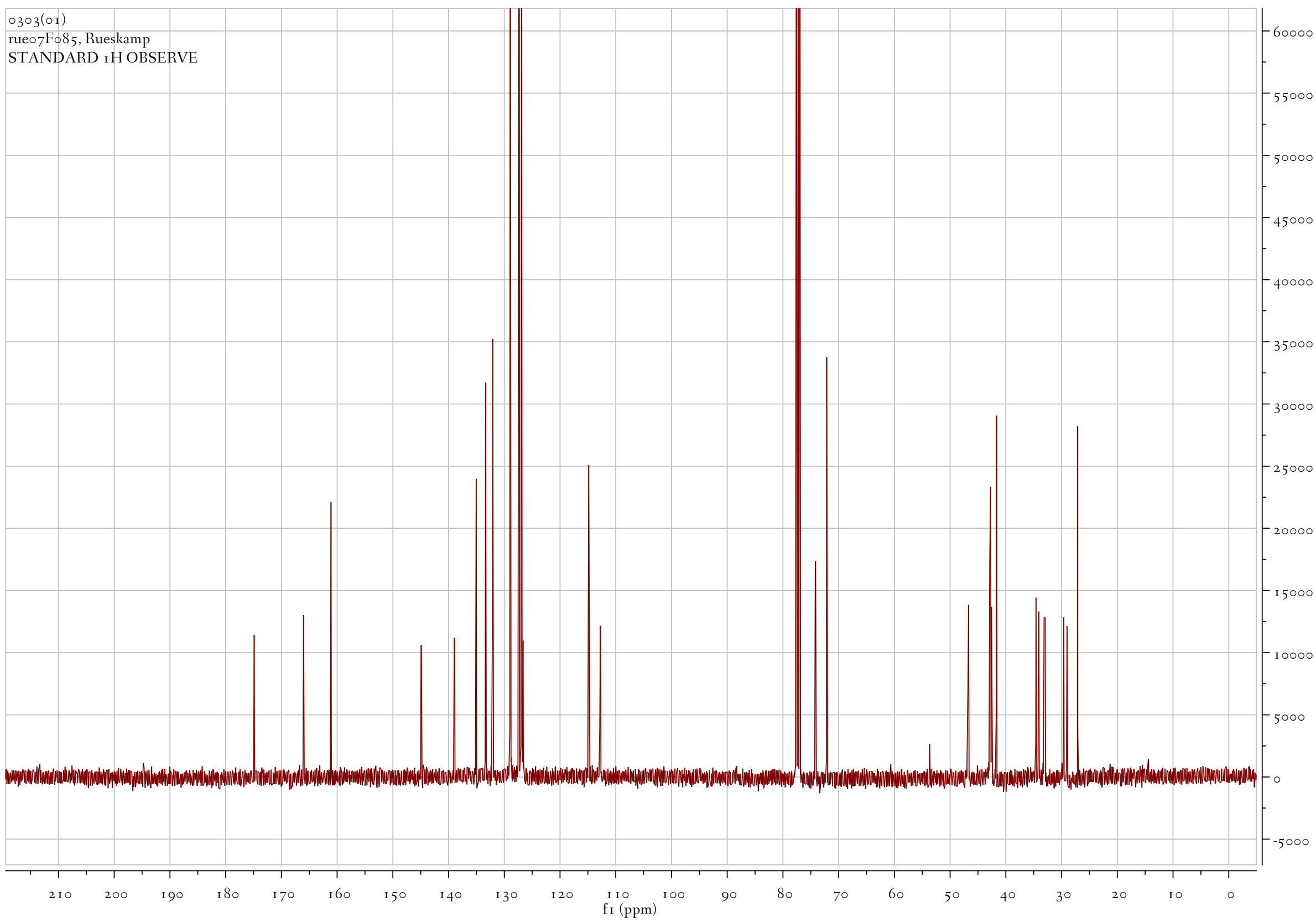

^{1}H NMR spectrum ($CDCl_3$) of b-hydroxy amide *trans*-**10b**

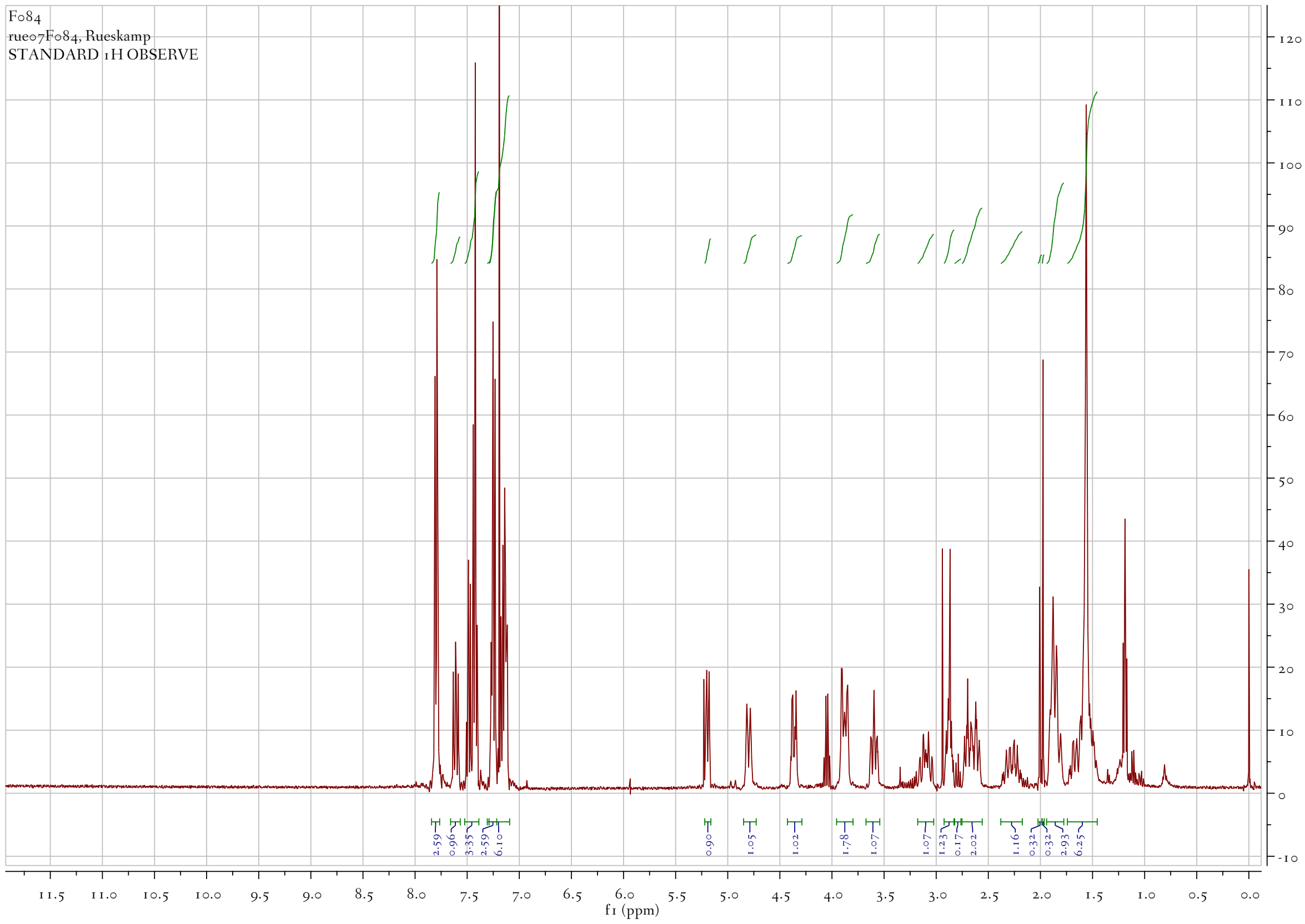
F084
rue07F084, Rueskamp
STANDARD 1H OBSERVE
f1 (ppm)

^{1}H and ^{13}C NMR spectrum ($CDCl_3$) of b-hydroxy amide **10c**

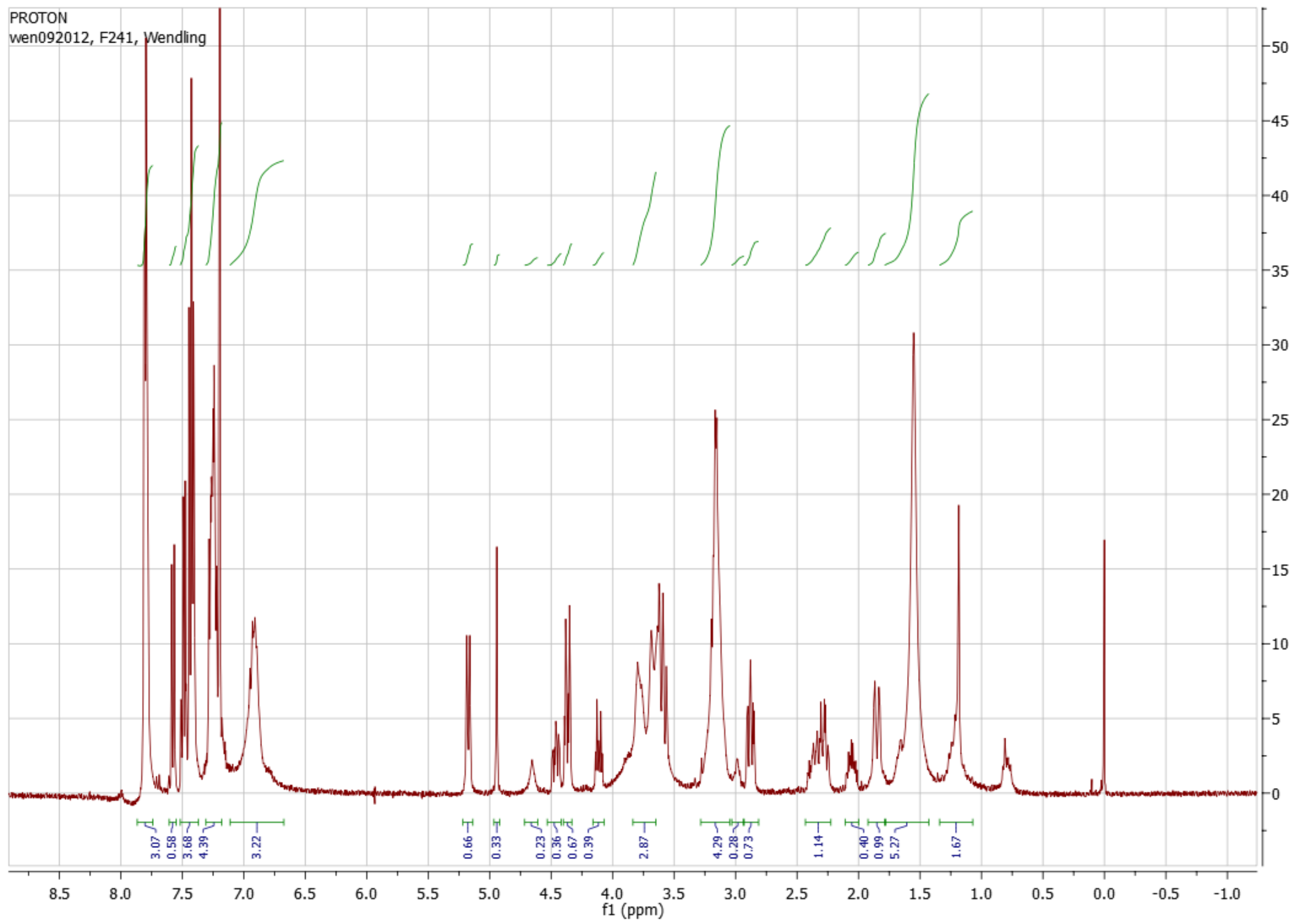

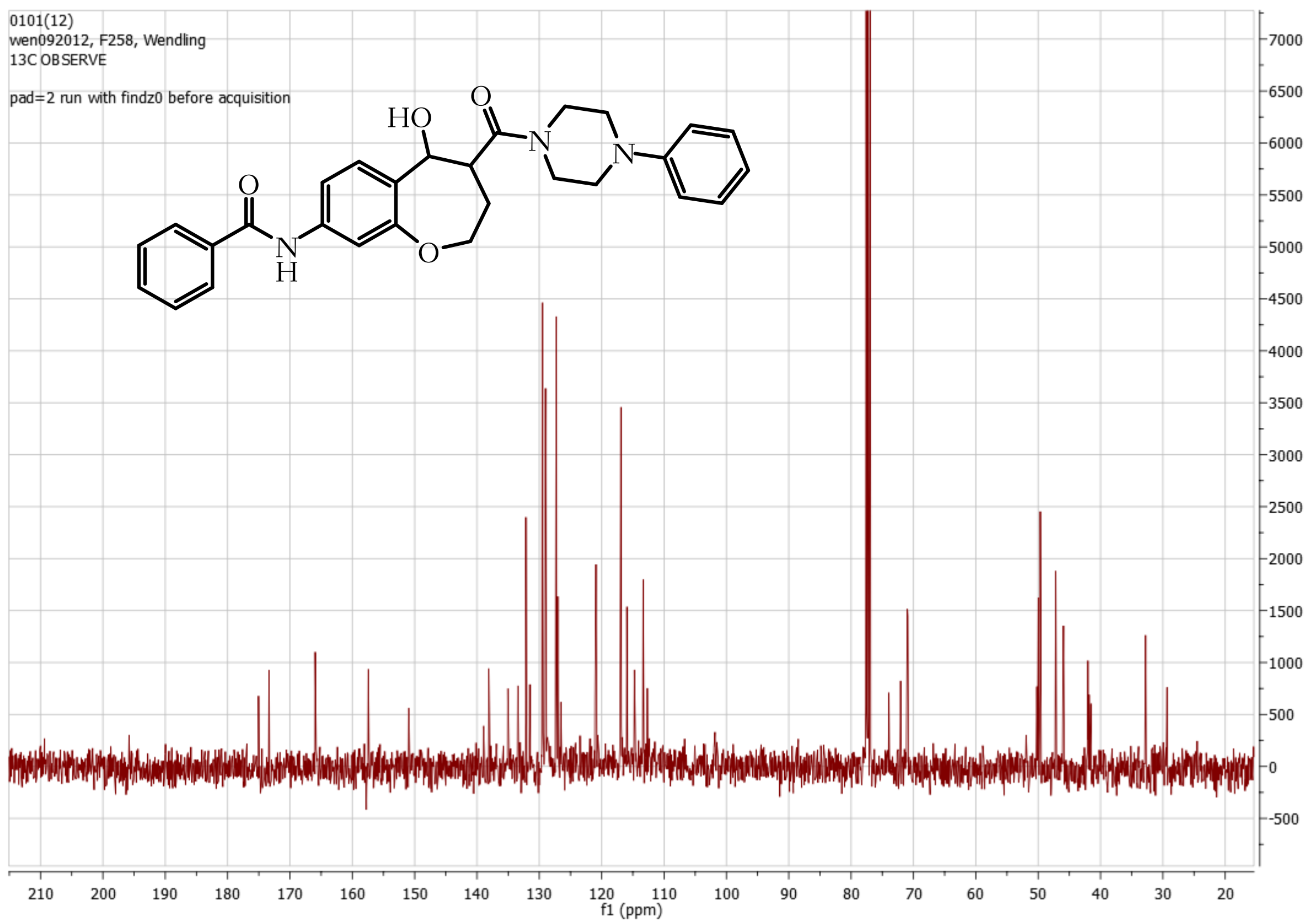

^{1}H NMR spectrum ($CDCl_3$) of b-hydroxy amide **10d**

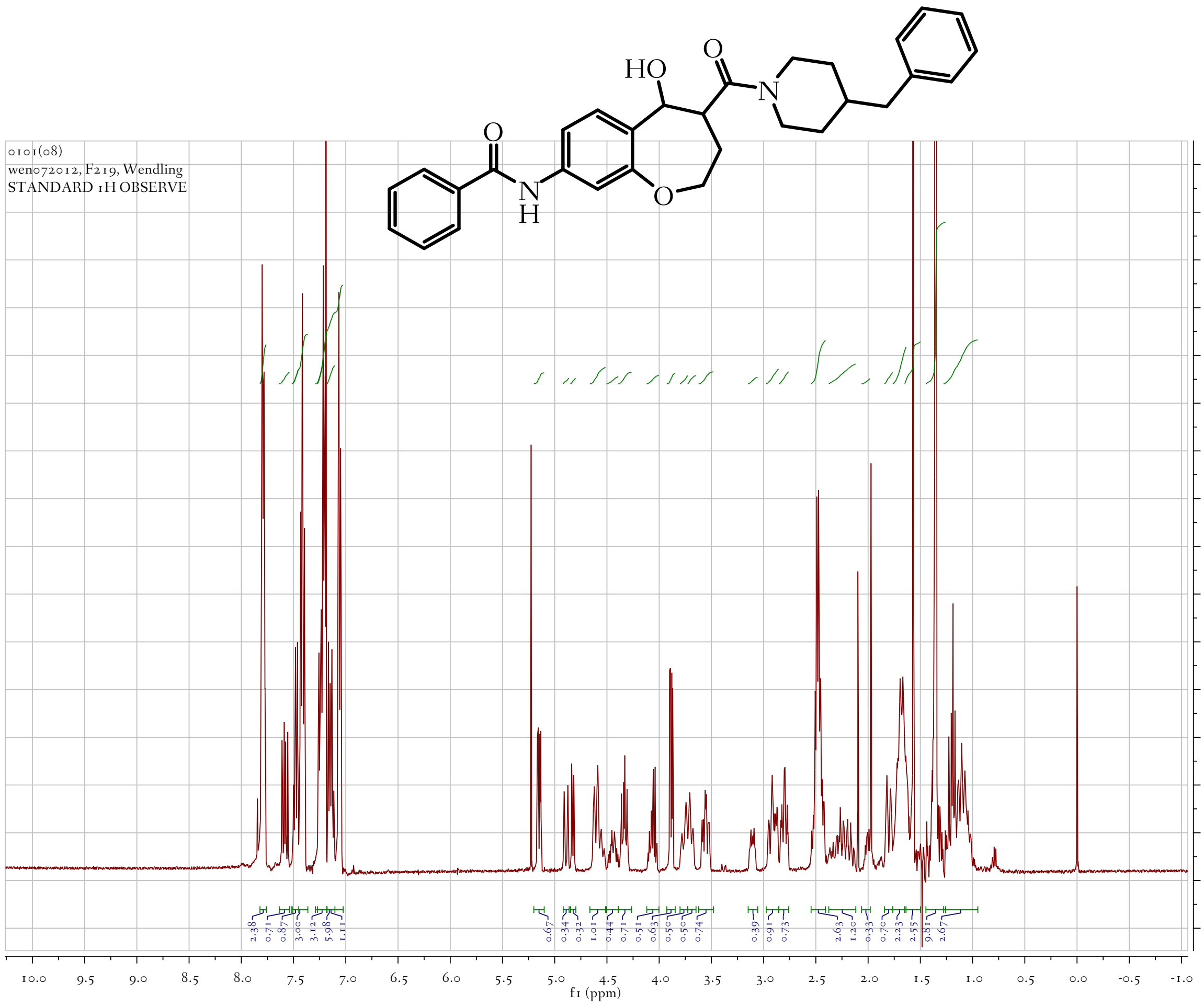

^{1}H and ^{13}C NMR spectrum ($CDCl_3$) of b-hydroxy amide *cis*-**10e**

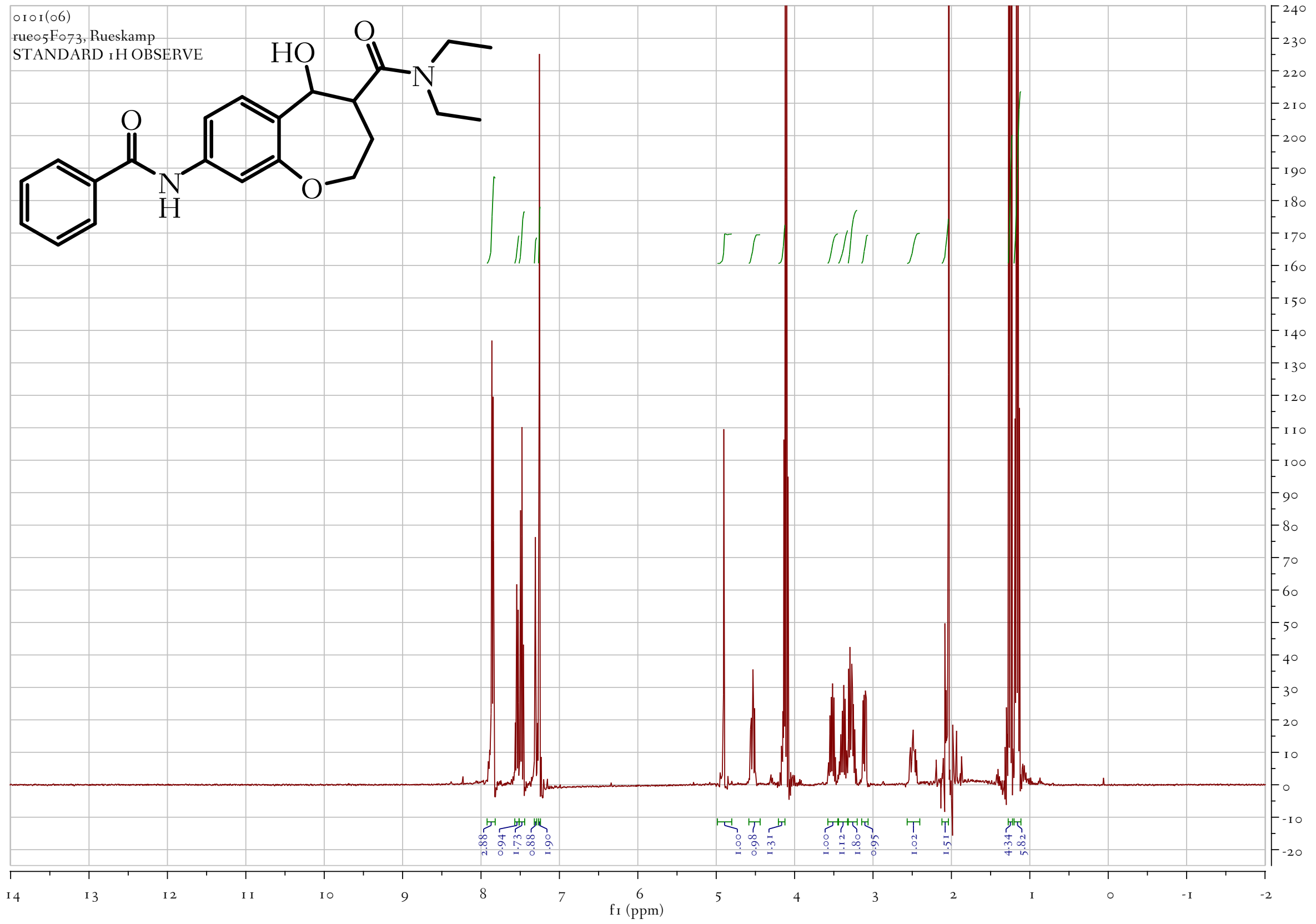

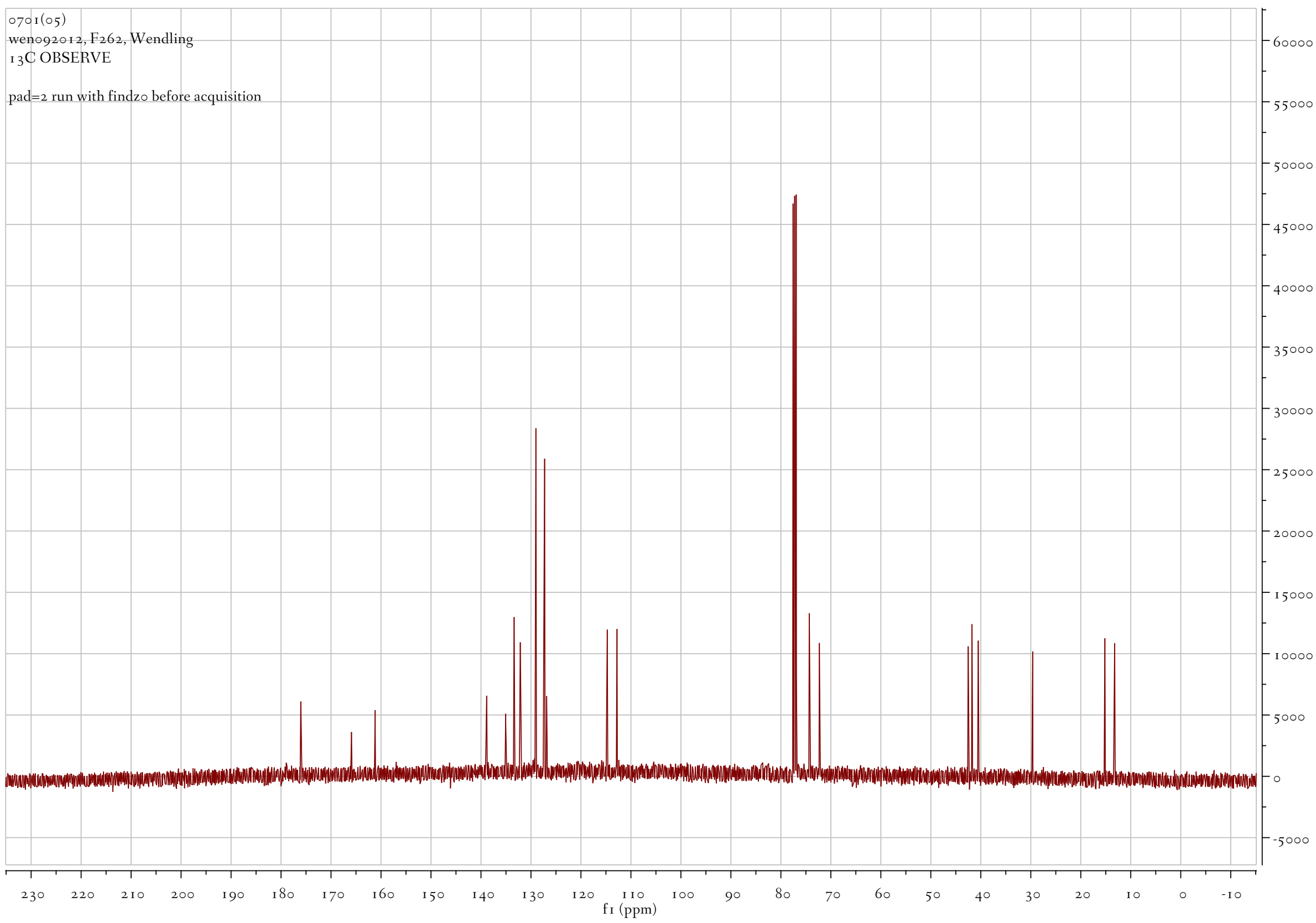

^{1}H NMR spectrum ($CDCl_3$) of b-hydroxy amide *trans*-**10e**

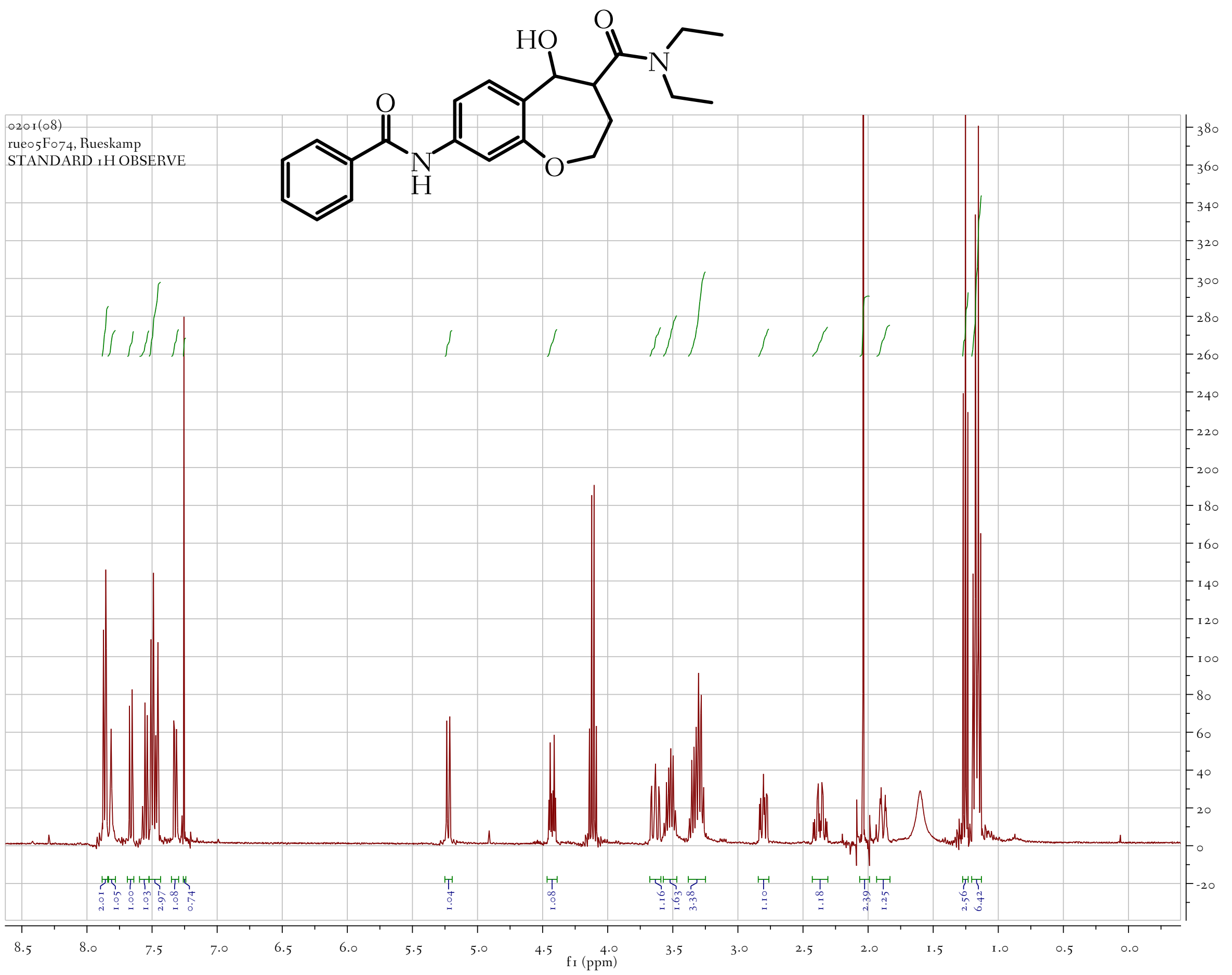

^{1}H and ^{13}C NMR spectrum ($CDCl_3$) of g-amino alcohol **11a**

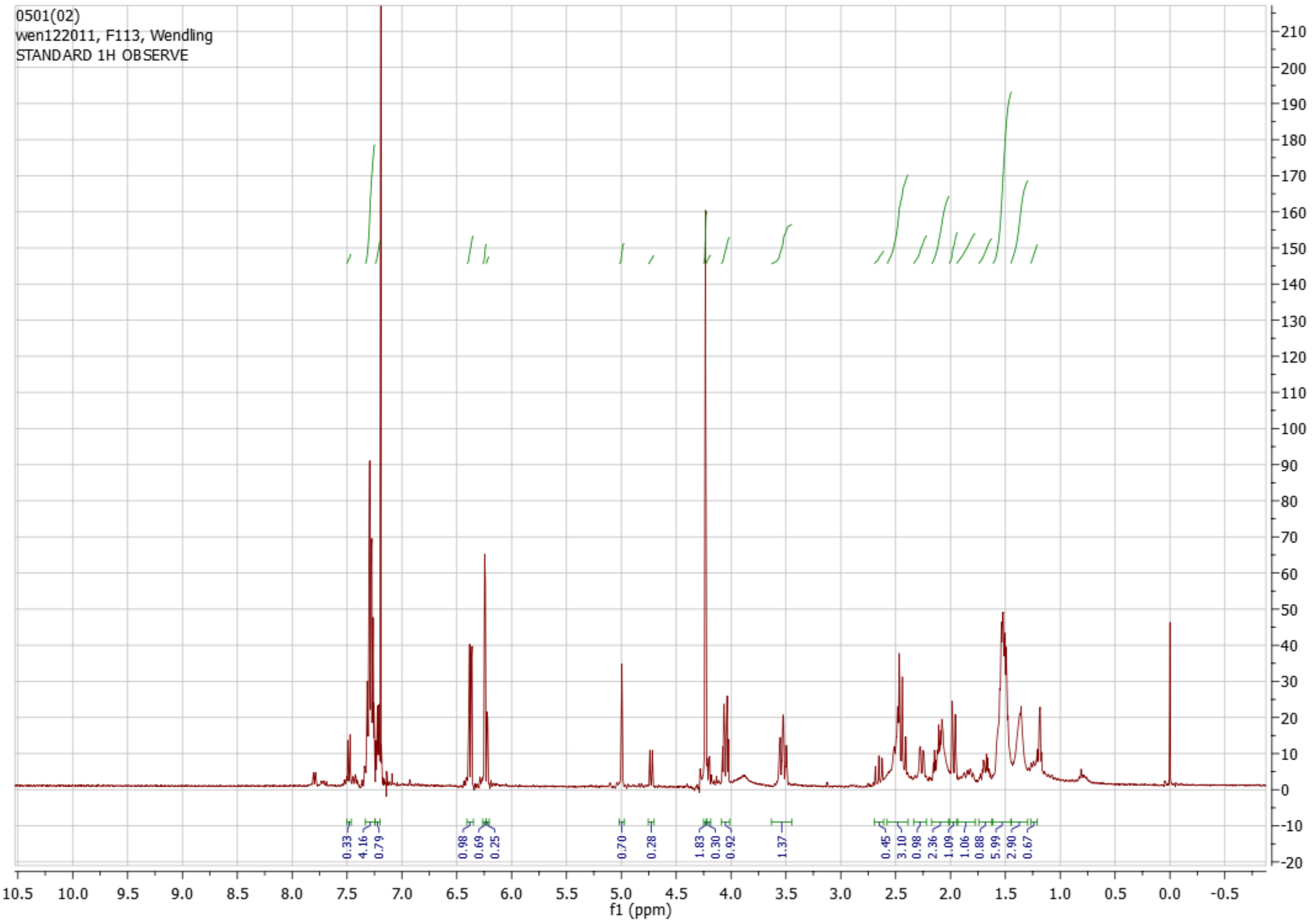

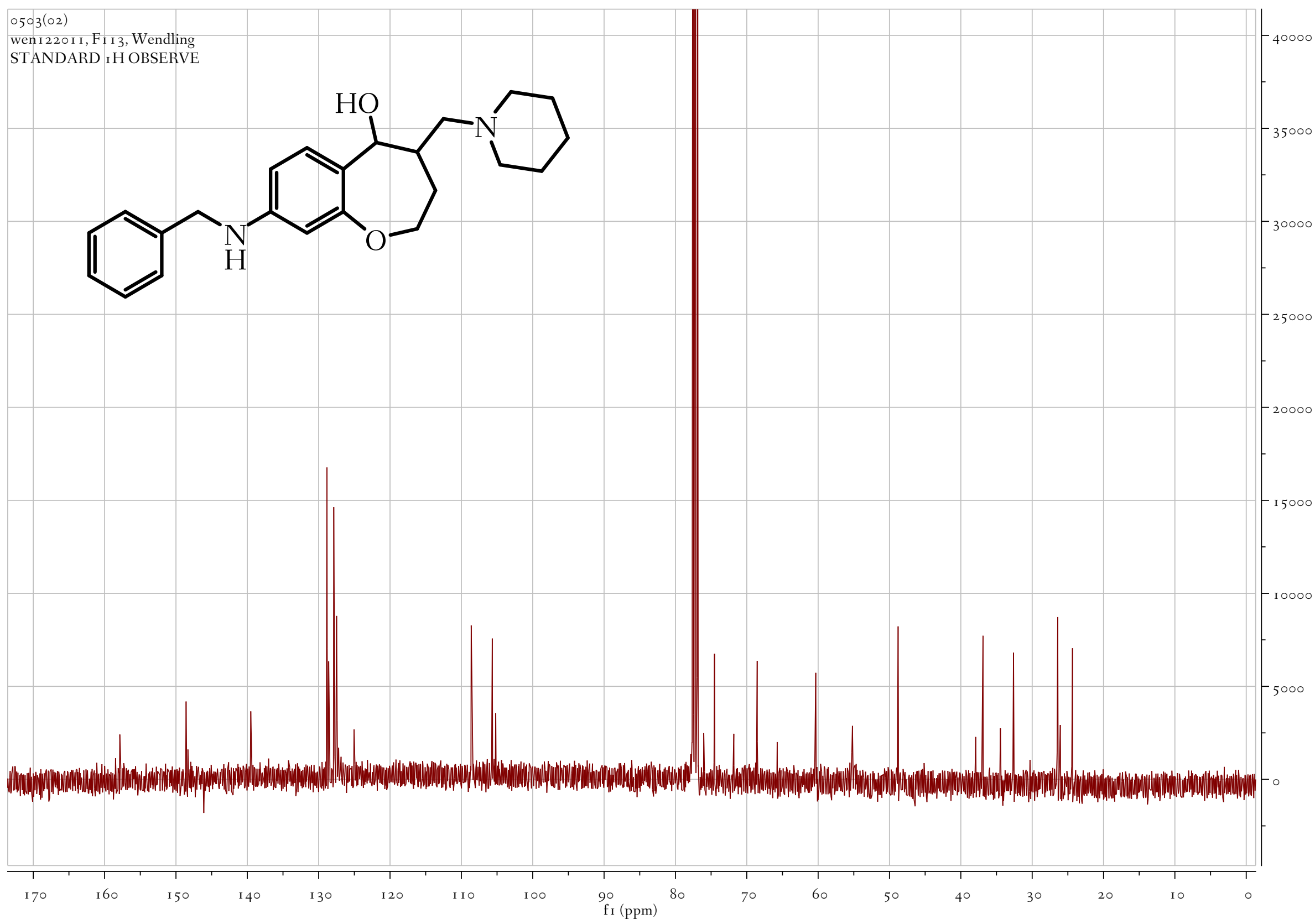

^{1}H NMR spectrum ($CDCl_3$) of g-amino alcohol **11b**

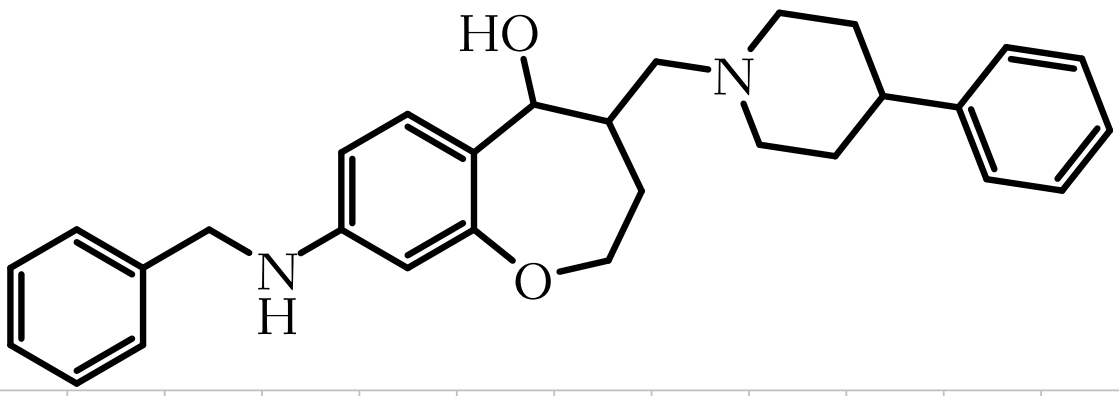

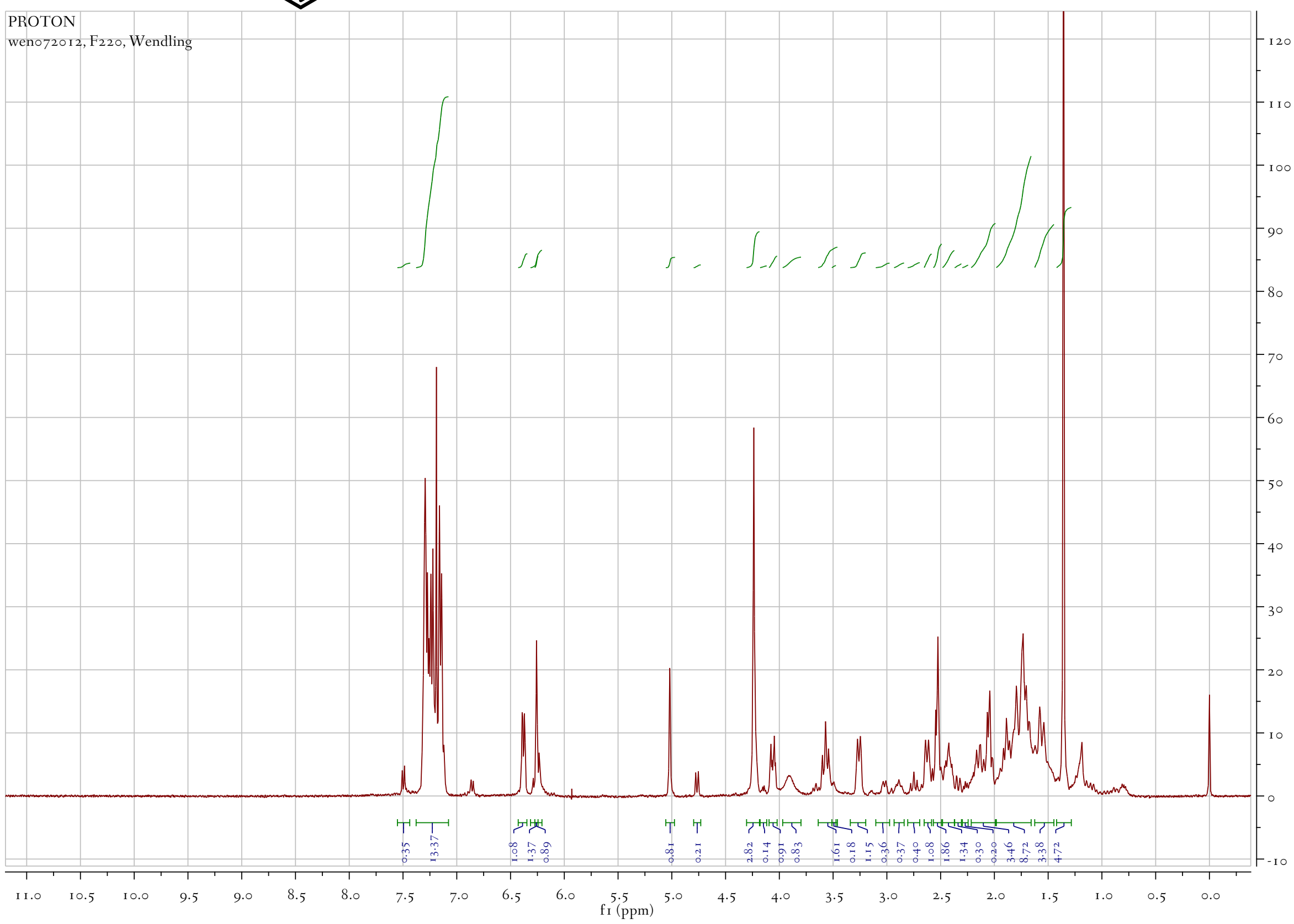

^{1}H and ^{13}C NMR spectrum ($CDCl_3$) of g-amino alcohol *cis*-**11b**

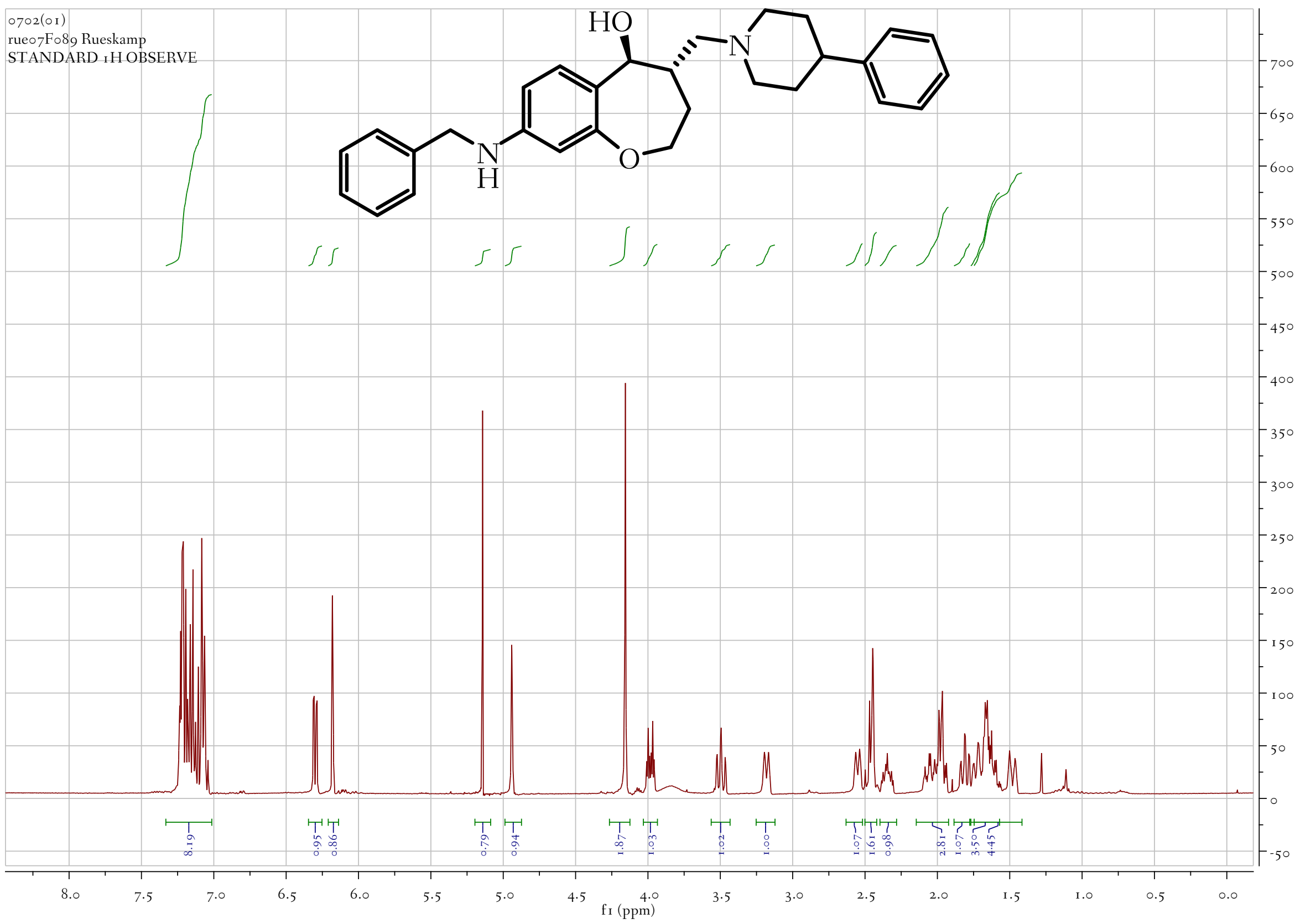

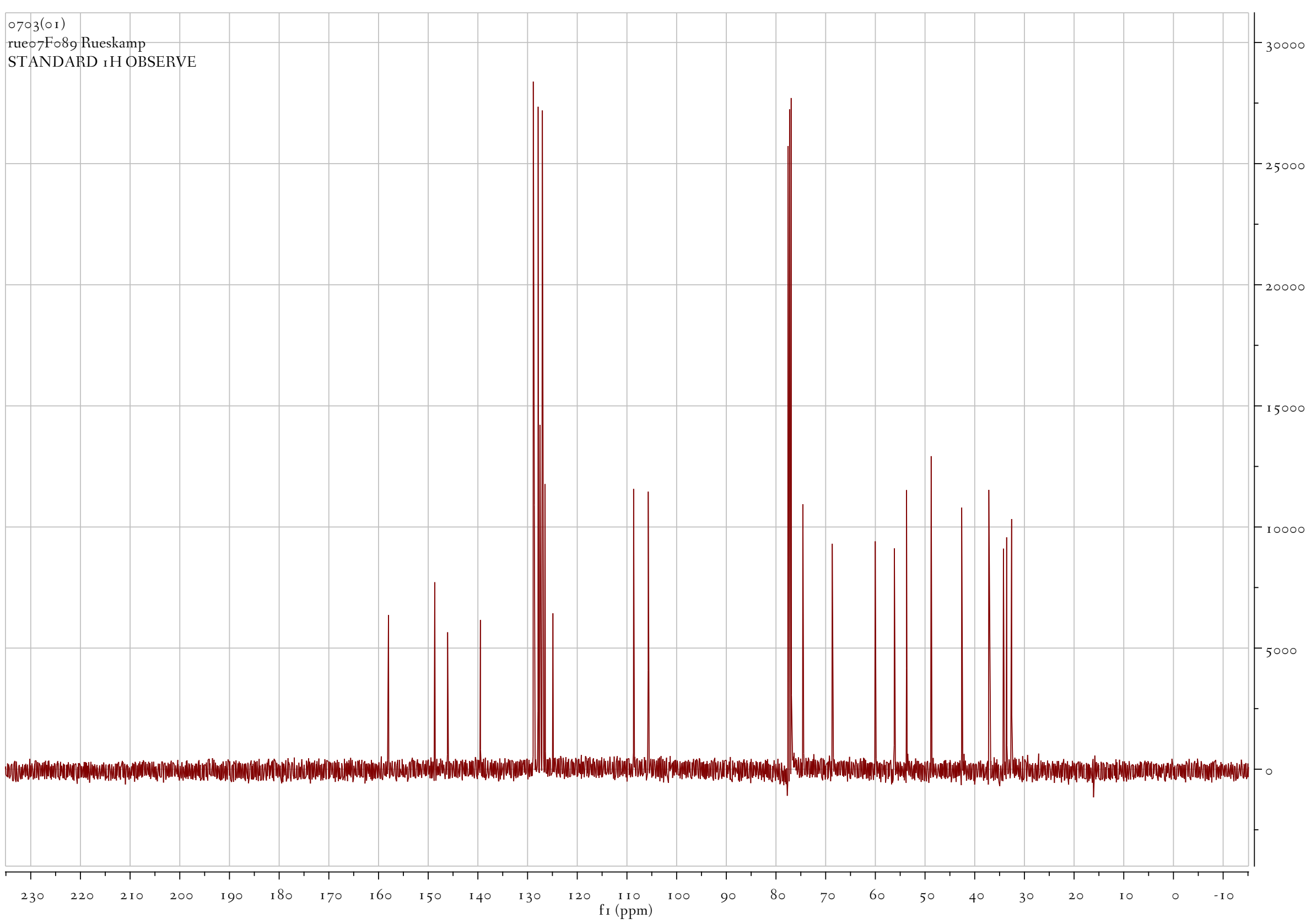

¹H NMR spectrum ($CDCl_3$) of g-amino alcohol *trans*-**11b**

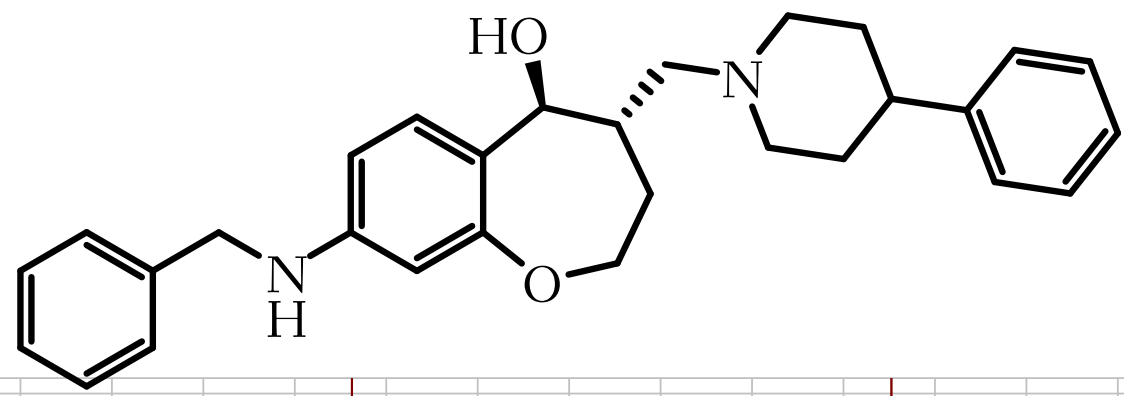

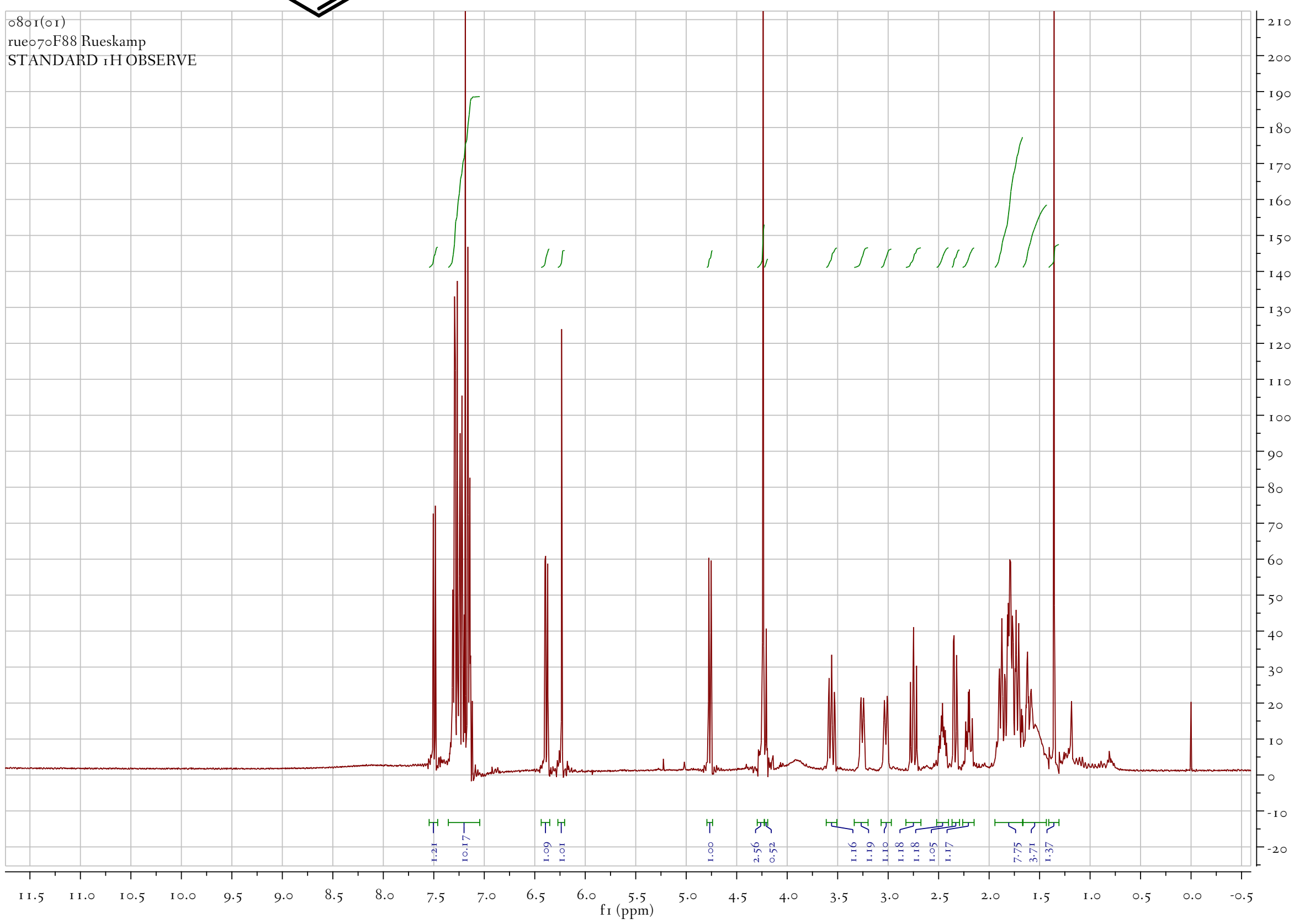

^{1}H and ^{13}C NMR spectrum ($CDCl_3$) of g-amino alcohol **11c**

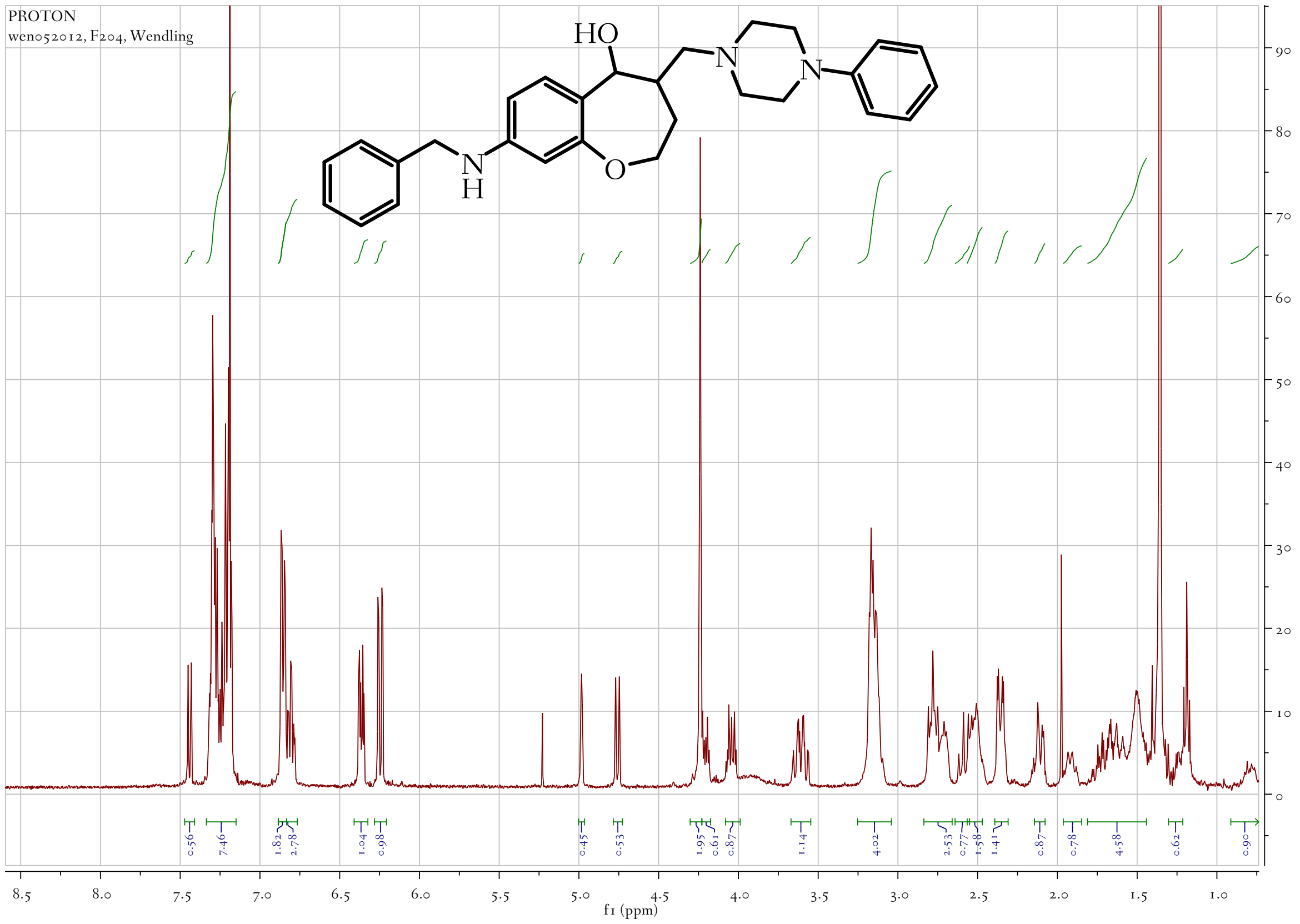

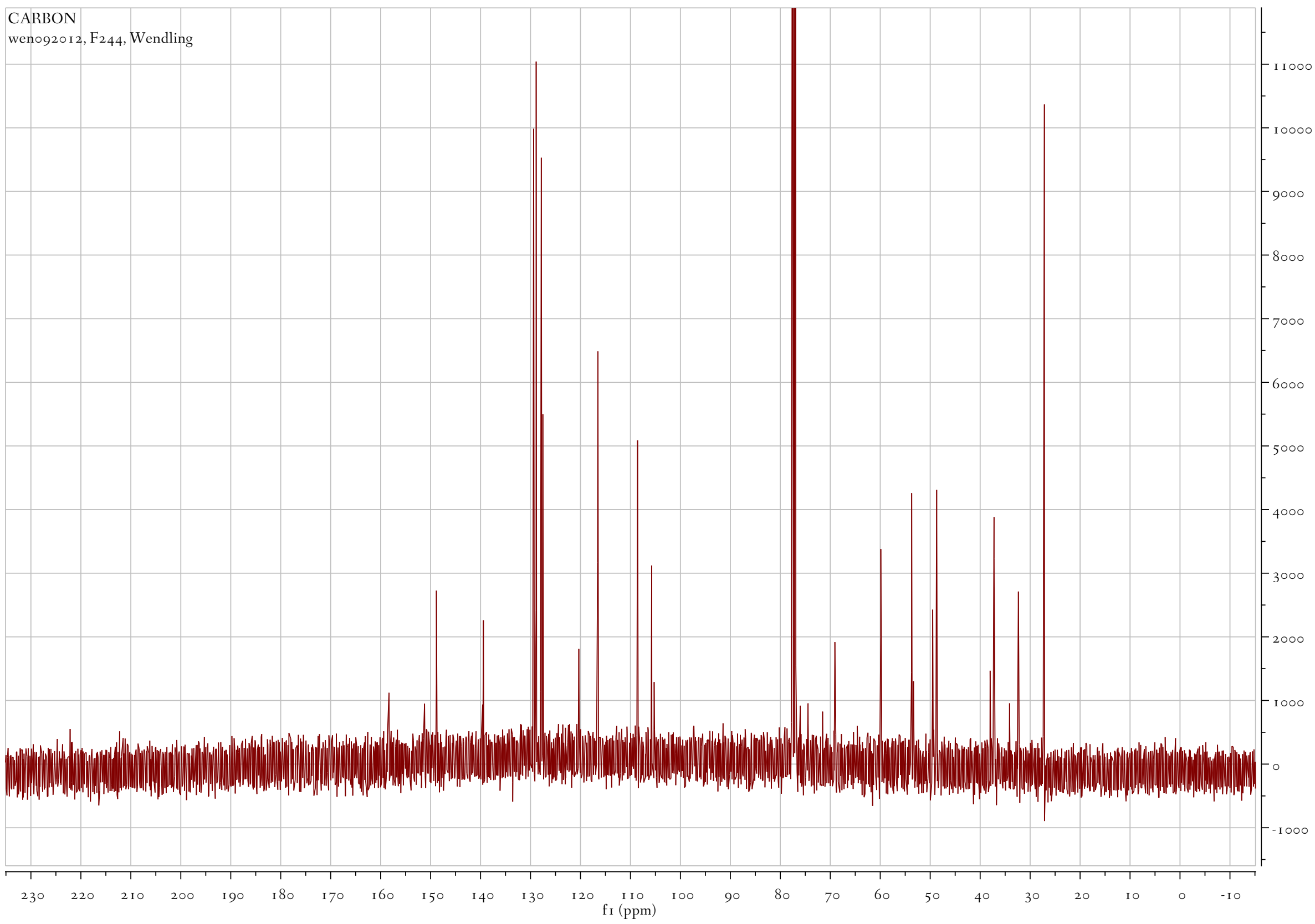

^{1}H and ^{13}C NMR spectrum ($CDCl_3$) of g-amino alcohol **11d**

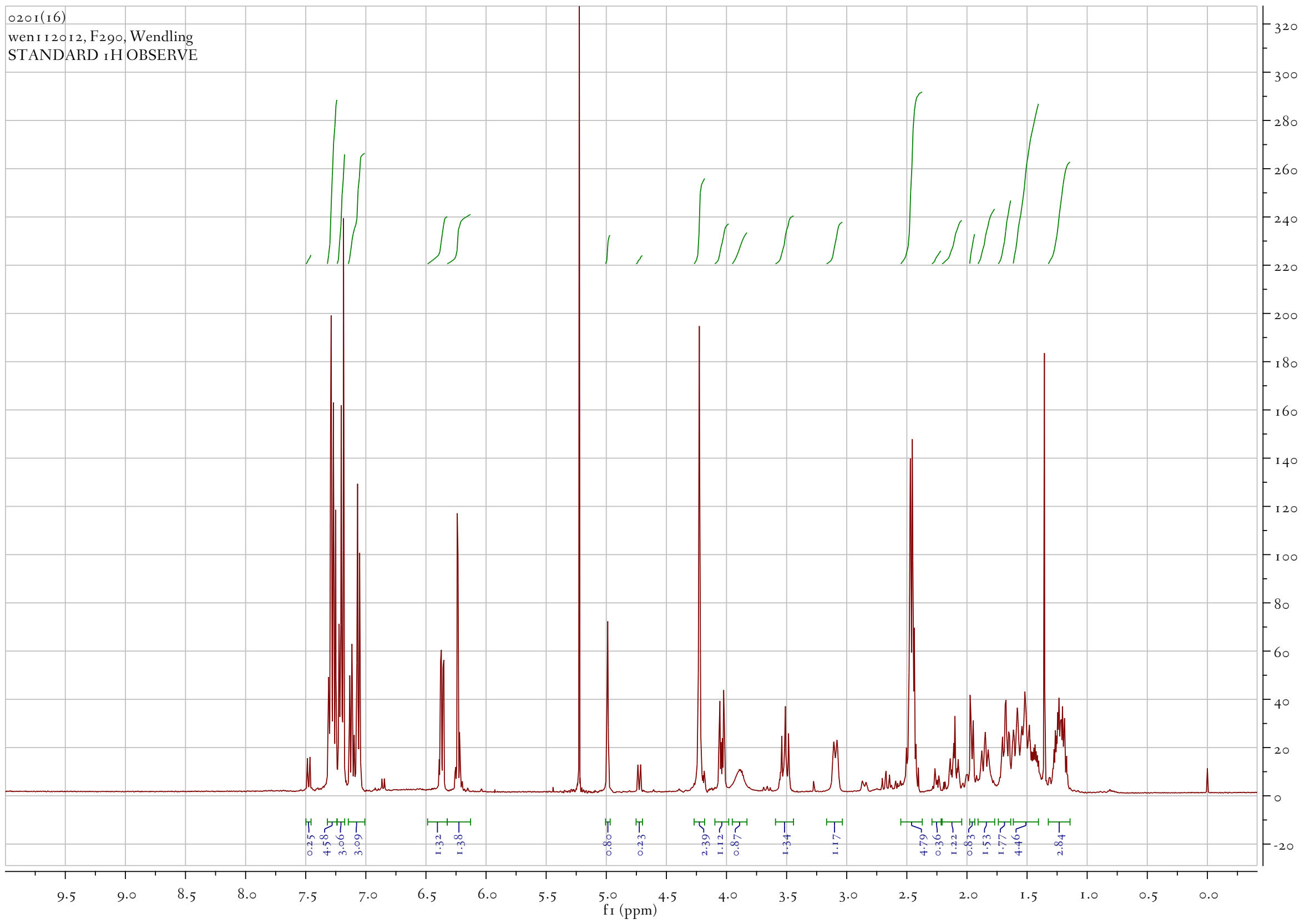

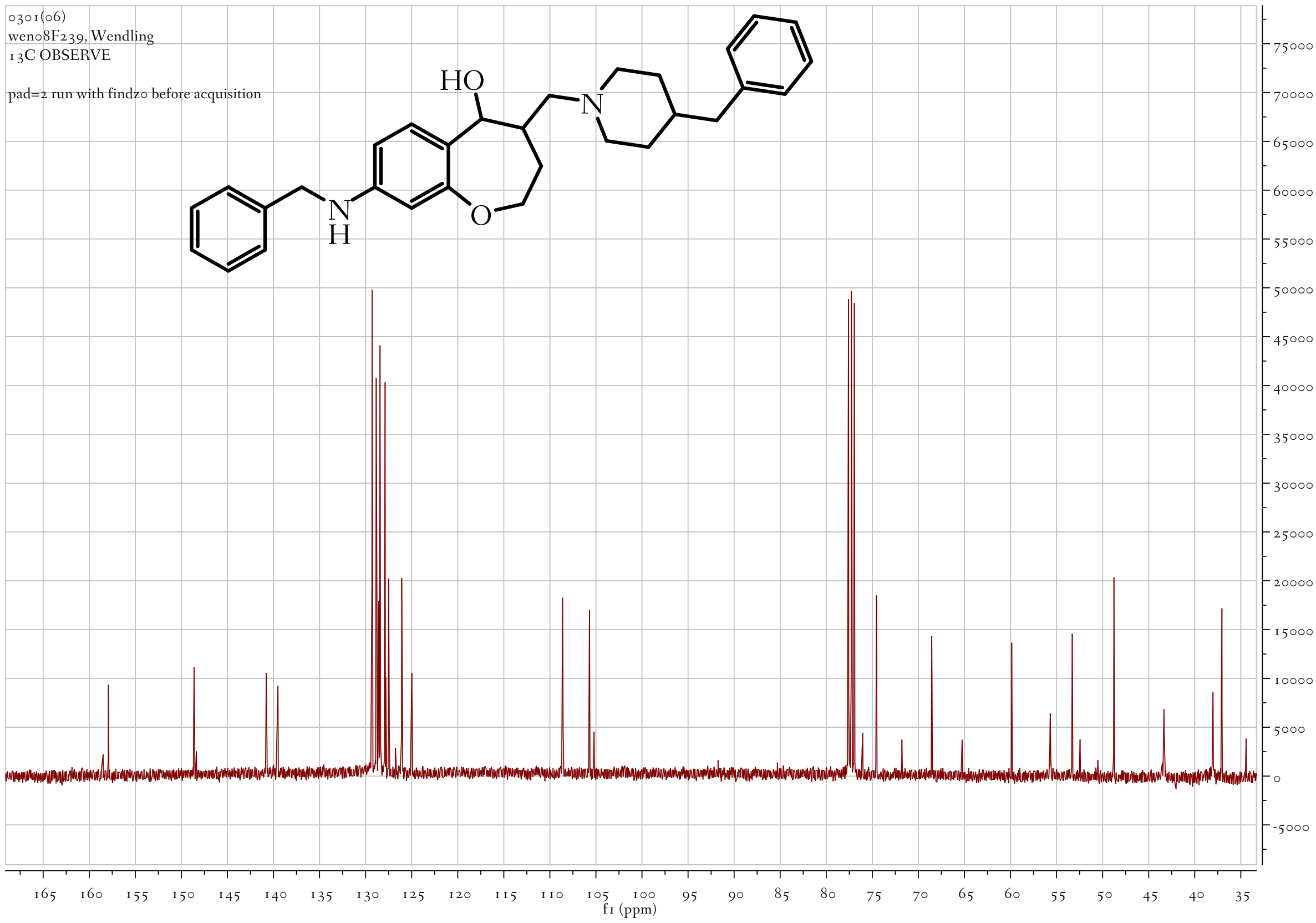

^{1}H and ^{13}C NMR spectrum ($CDCl_3$) of primary amine **12a**

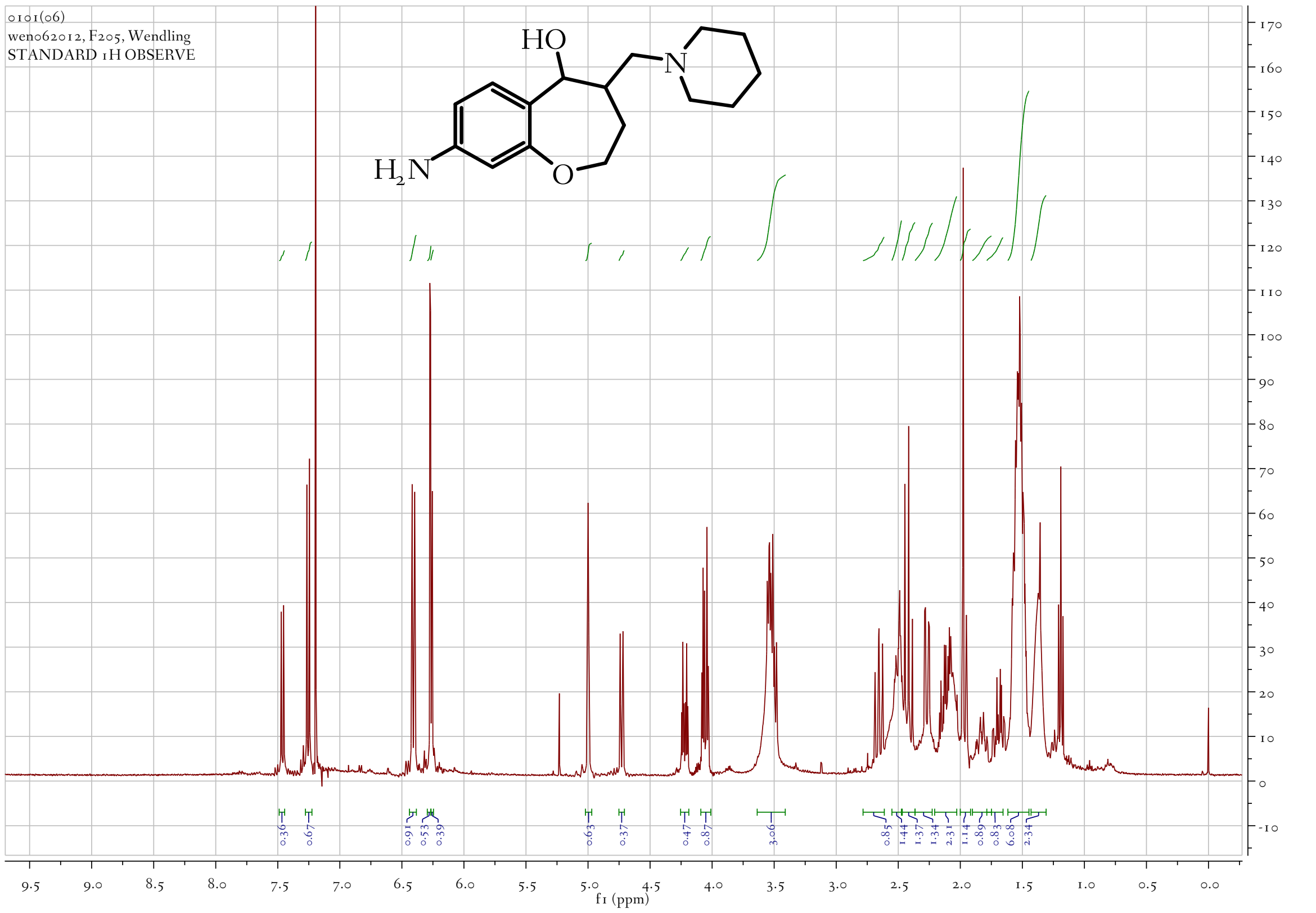

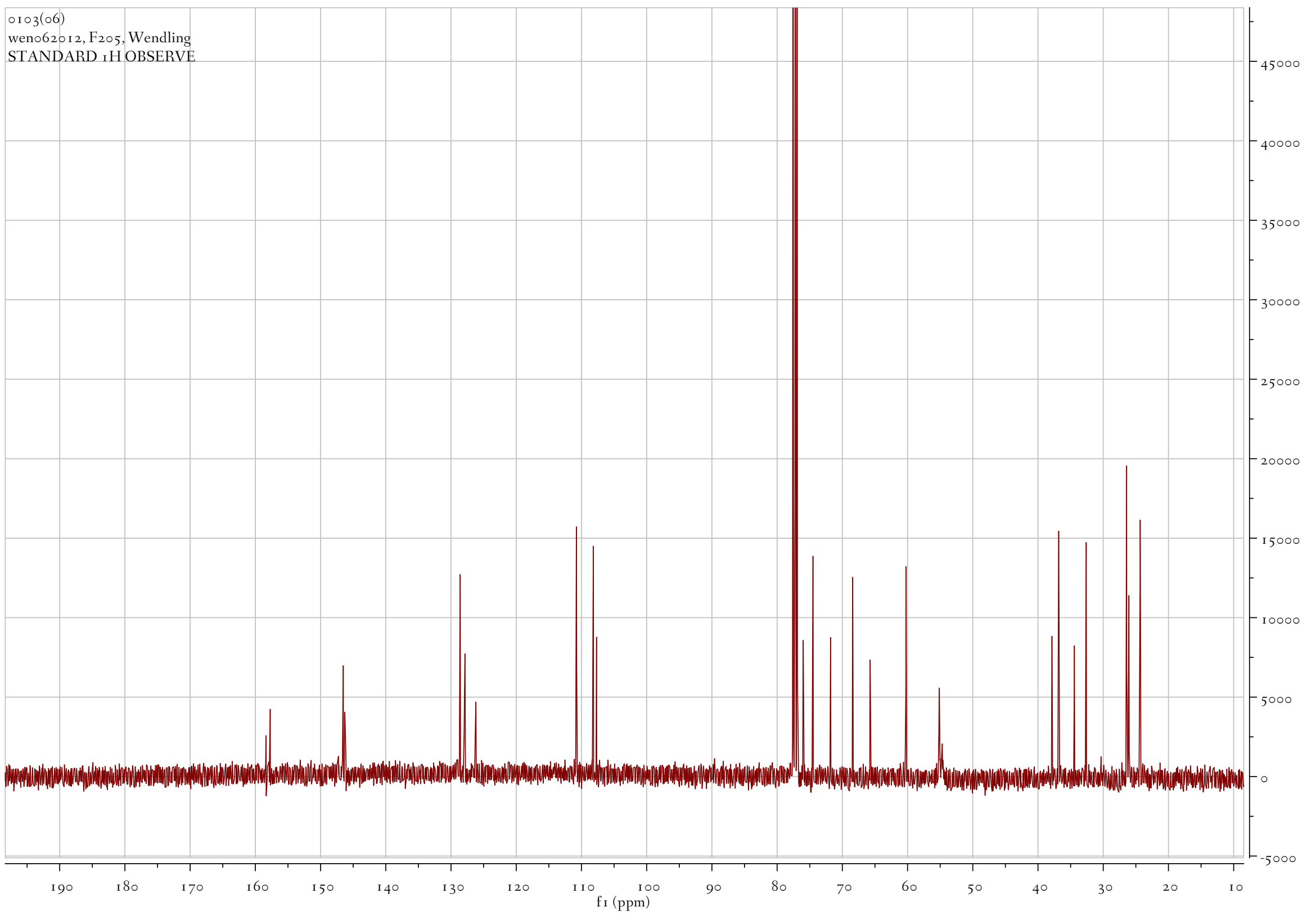

^{1}H and ^{13}C NMR spectrum ($CDCl_3$) of primary amine **12b**

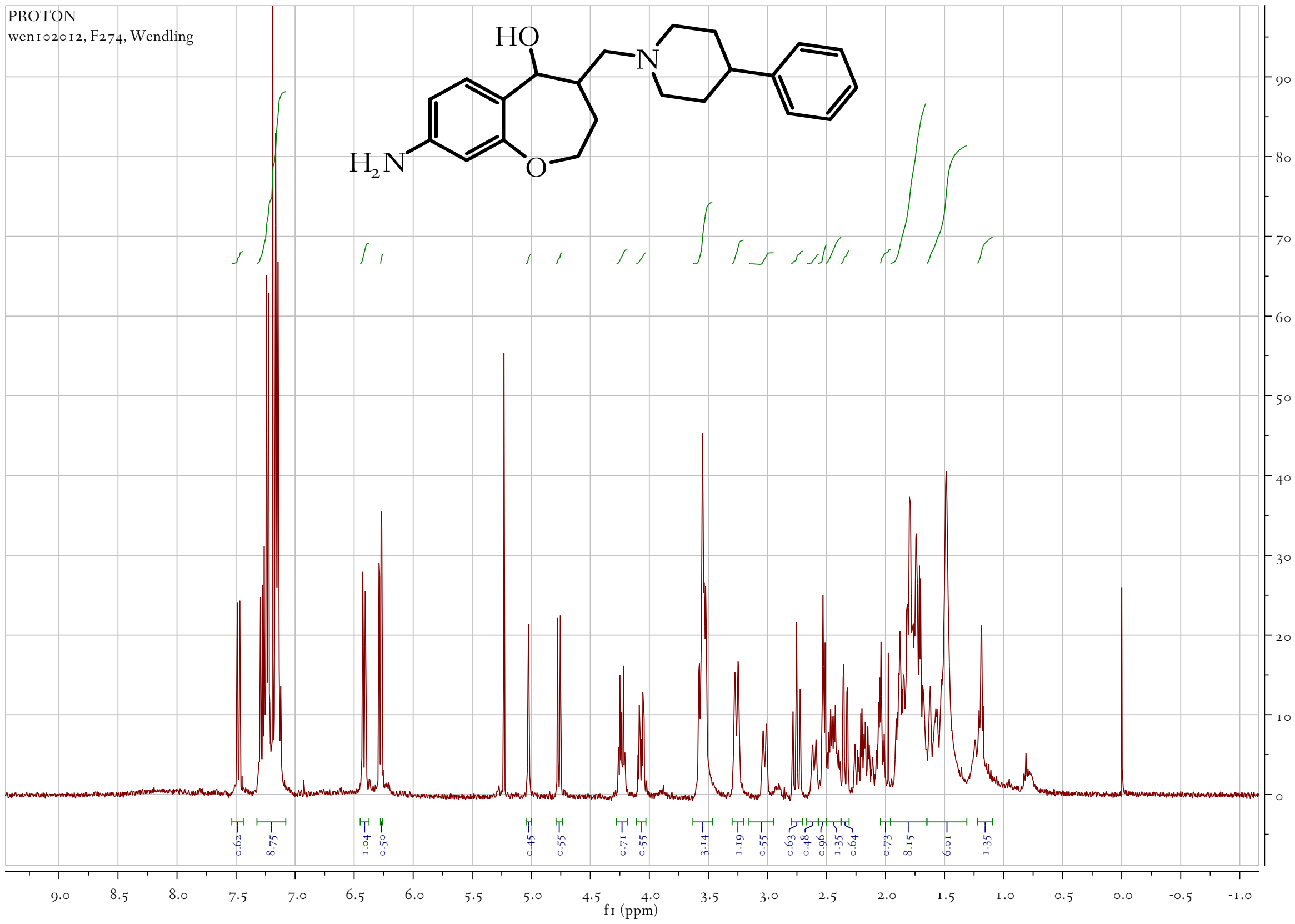

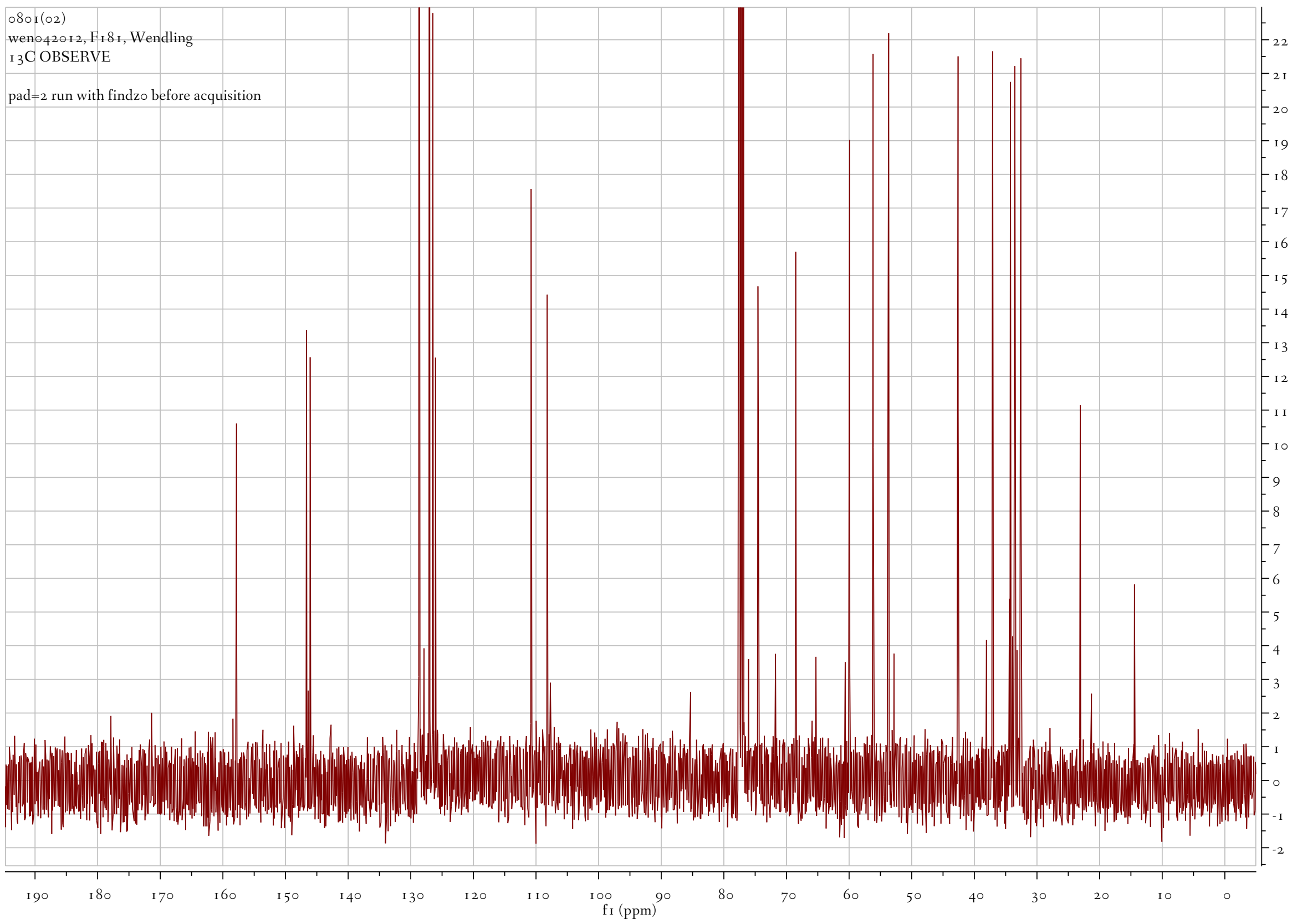

^{1}H and ^{13}C NMR spectrum ($CDCl_3$) of primary amine **12c**

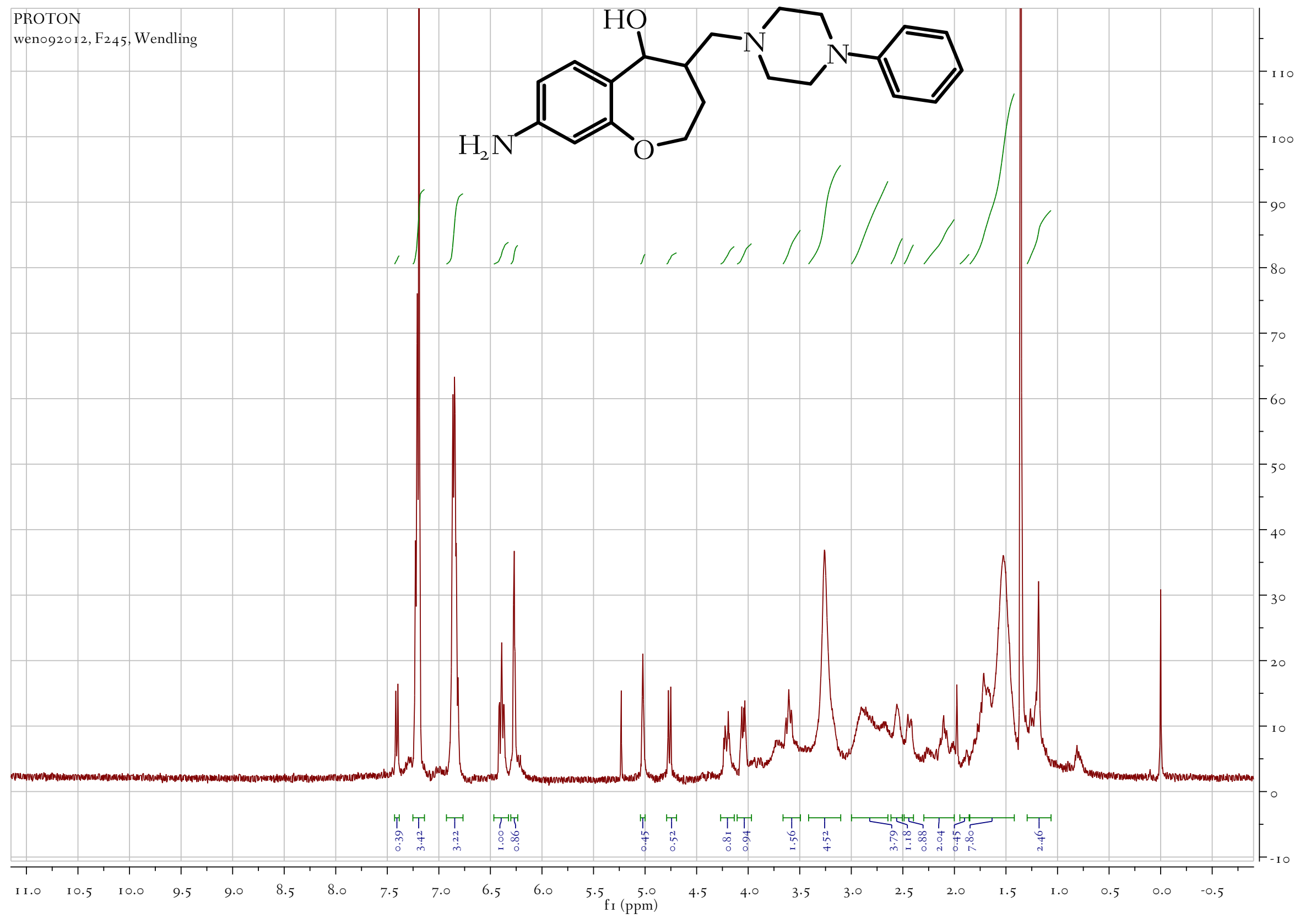

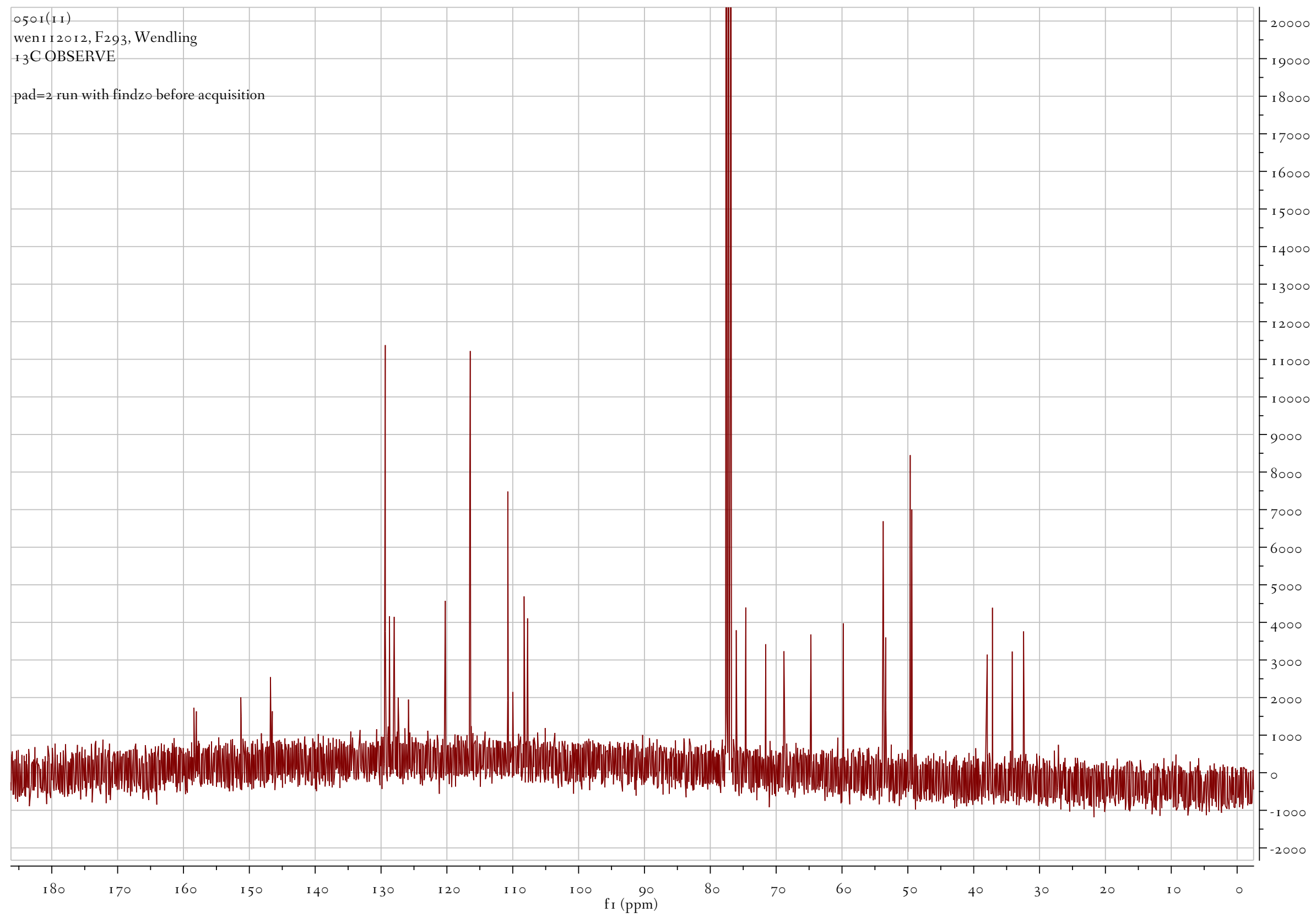

^{1}H NMR spectrum ($CDCl_3$) of primary amine **12d**

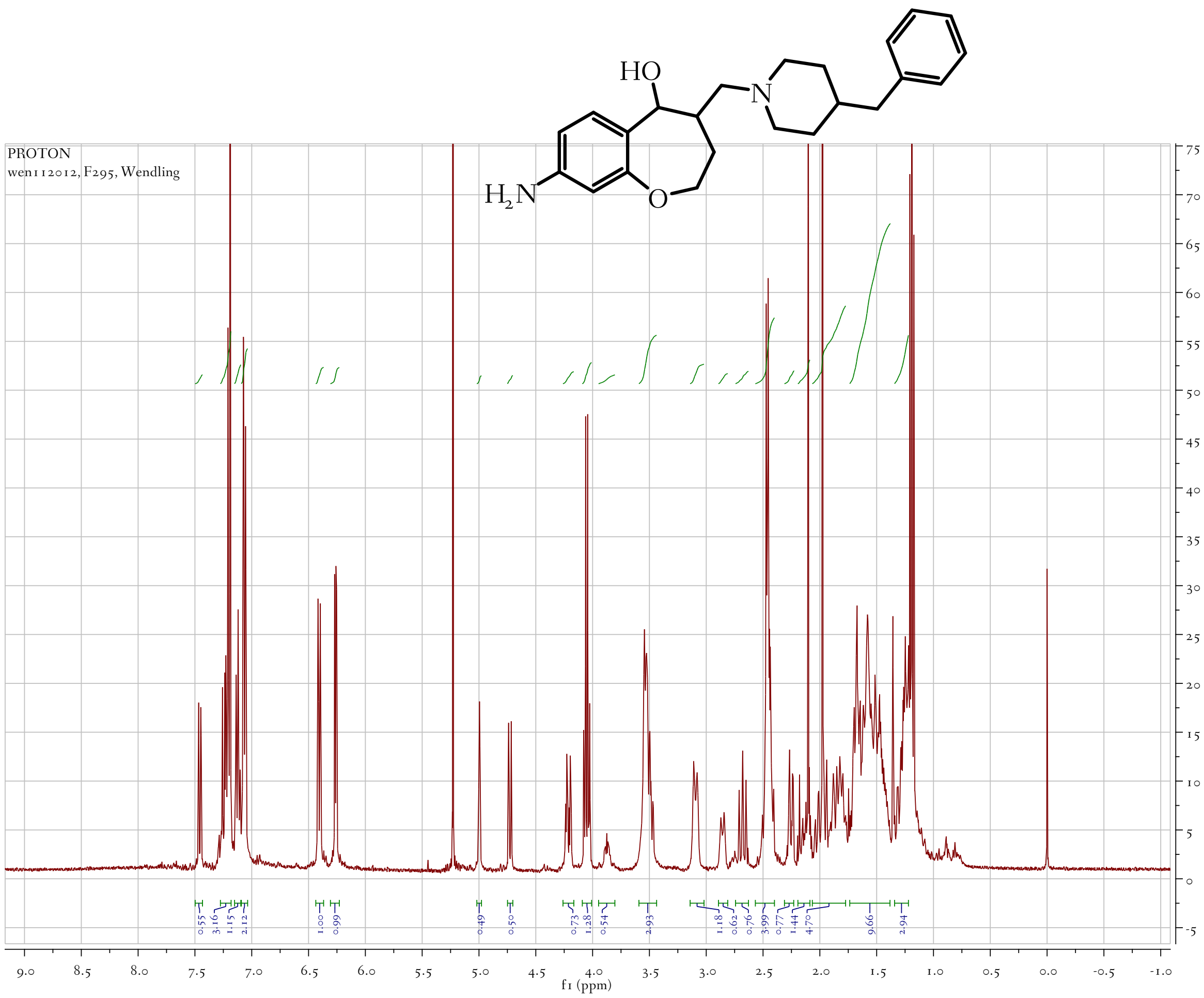

^{1}H and ^{13}C NMR spectrum ($CDCl_3$) of primary amine *cis*-**12d**

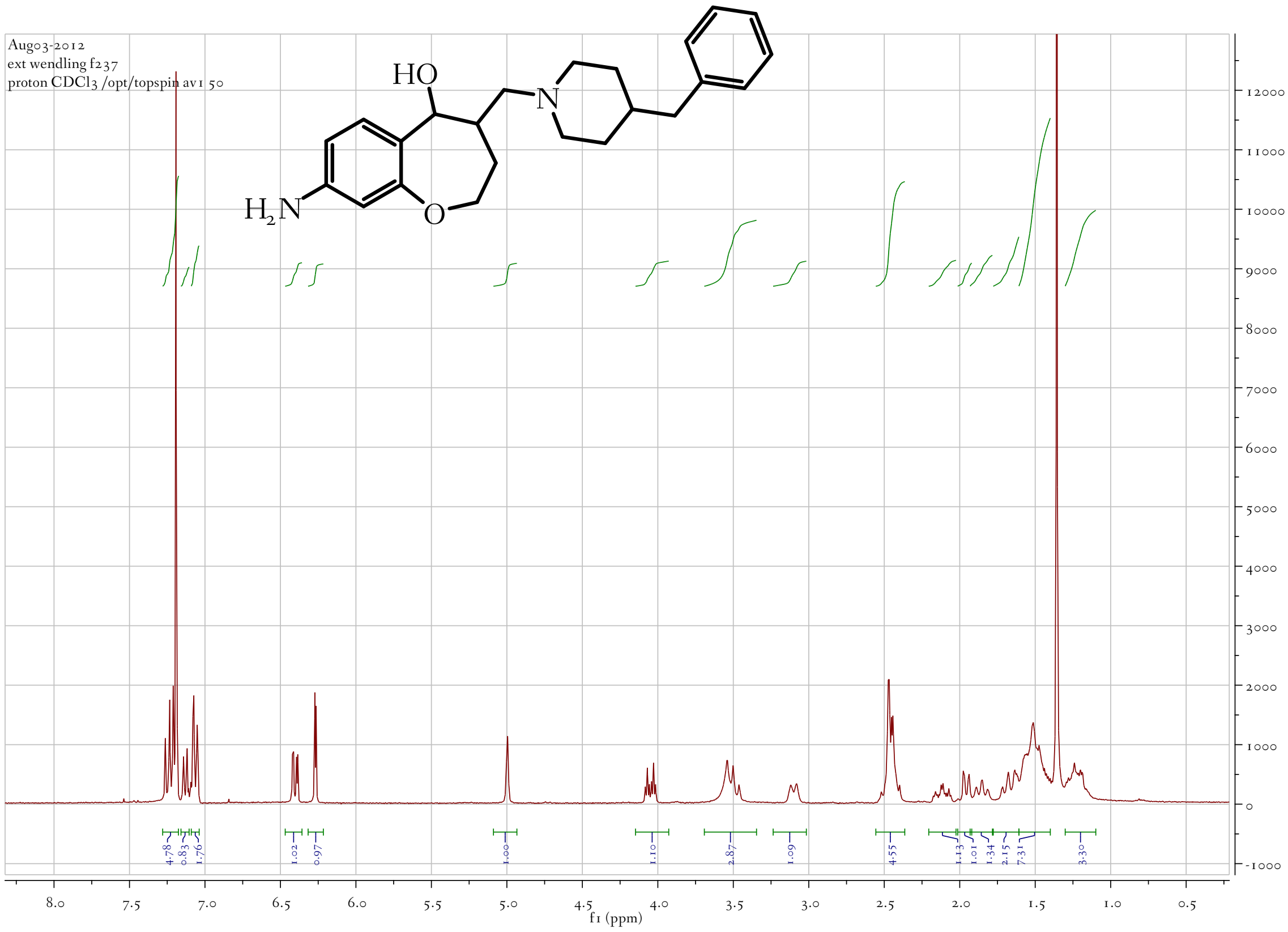

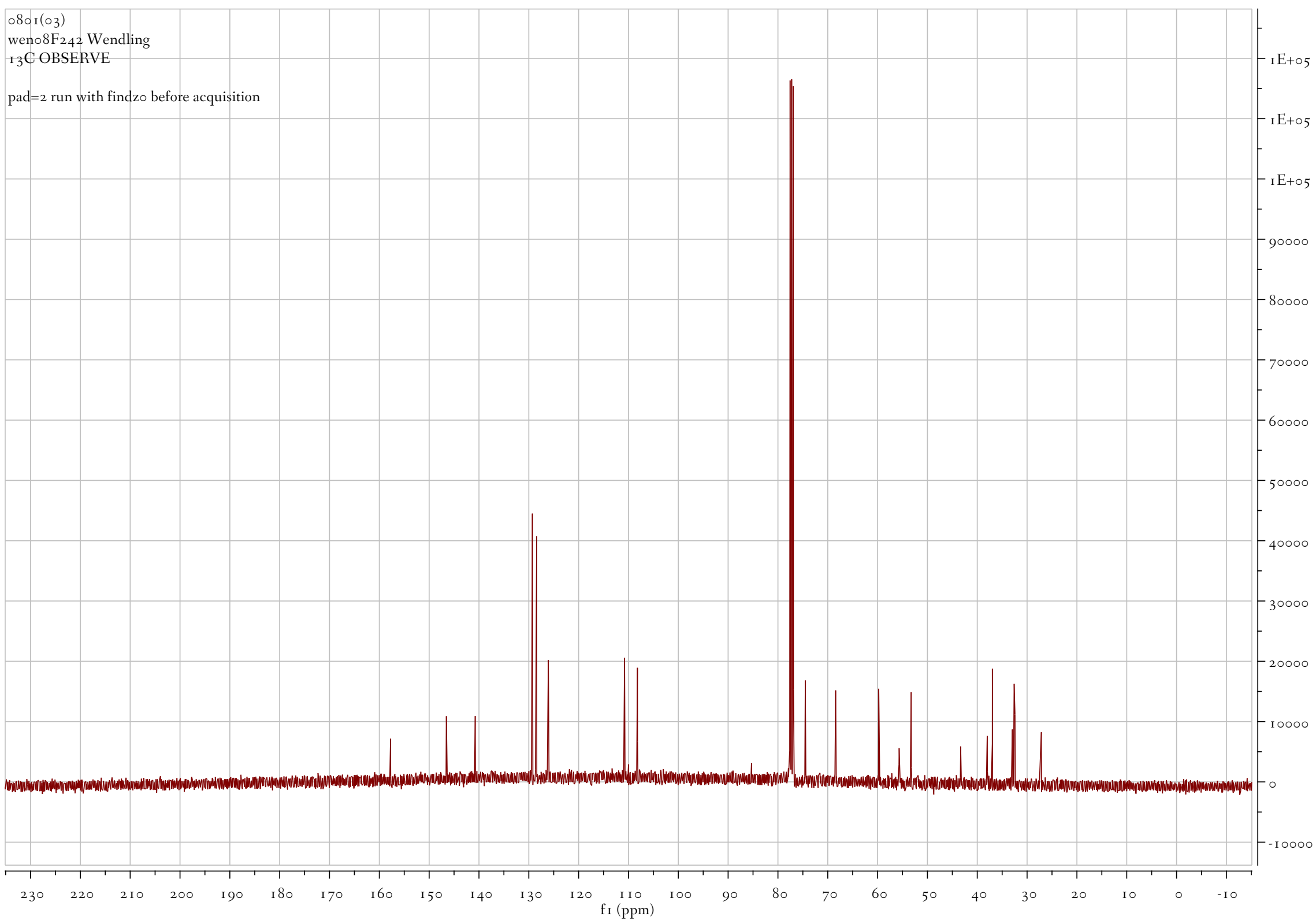

^{1}H and ^{13}C NMR spectrum ($CDCl_3$) of formamide **13b**

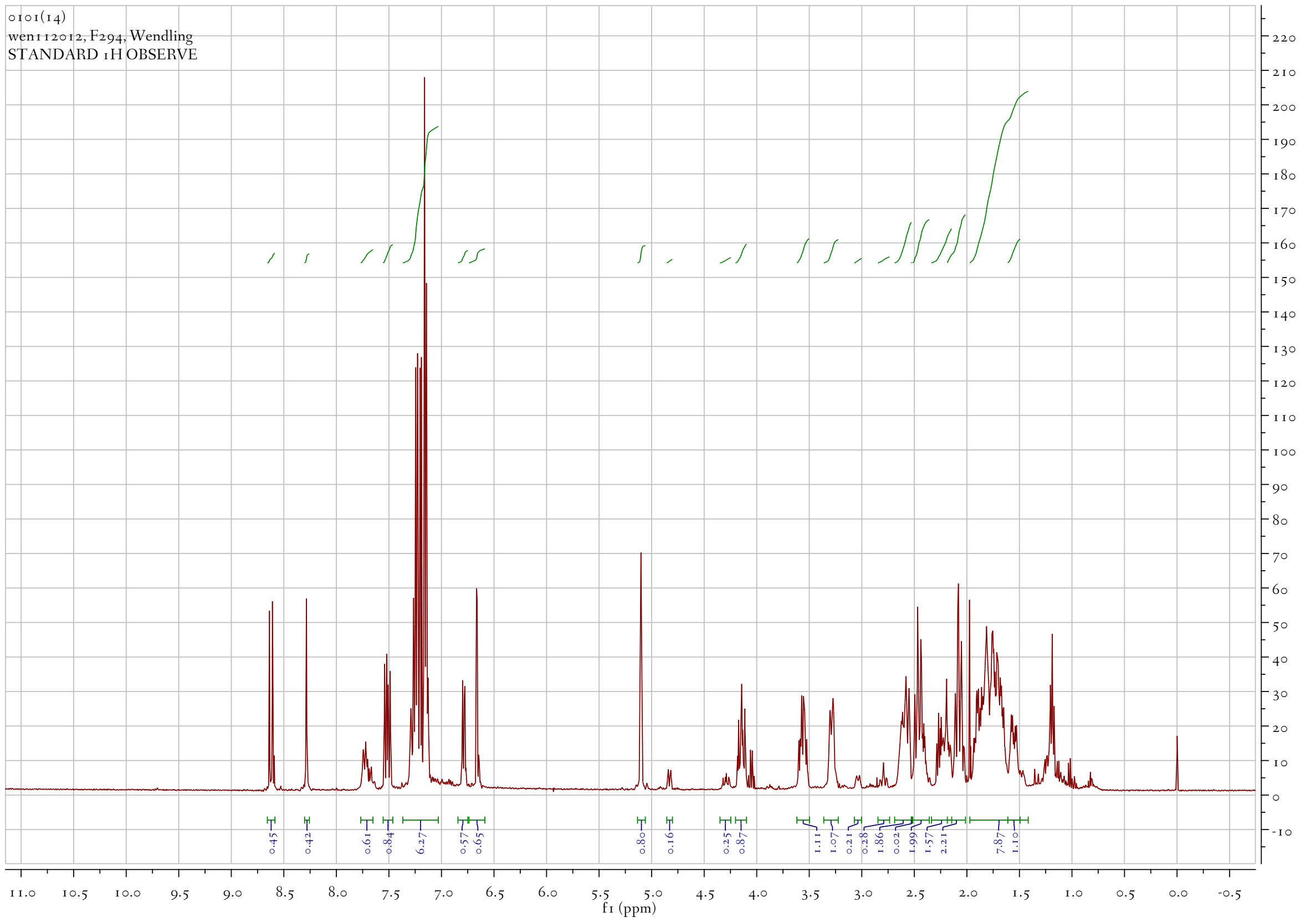

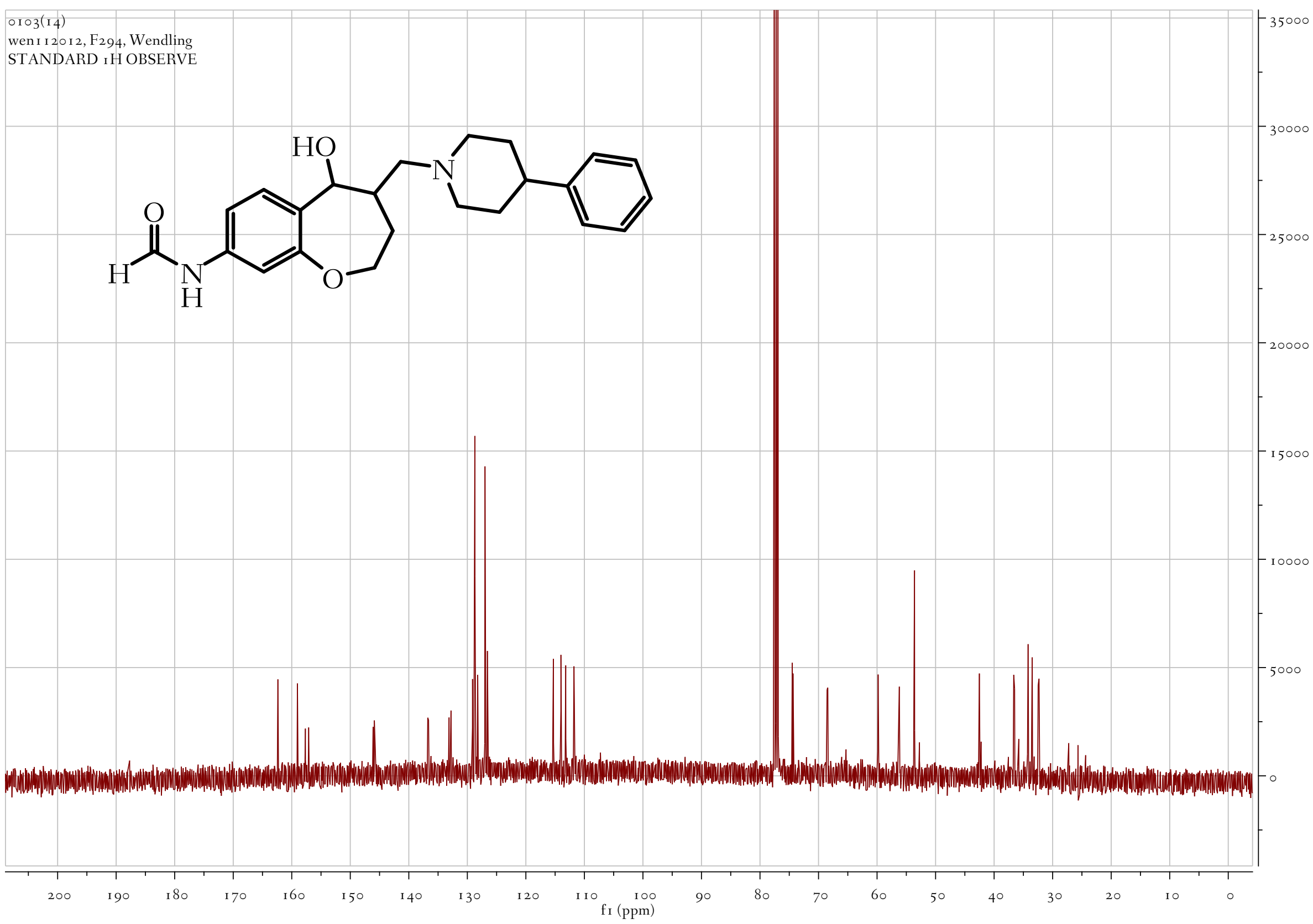

^{1}H NMR spectrum ($CDCl_3$) of formamide **13d**

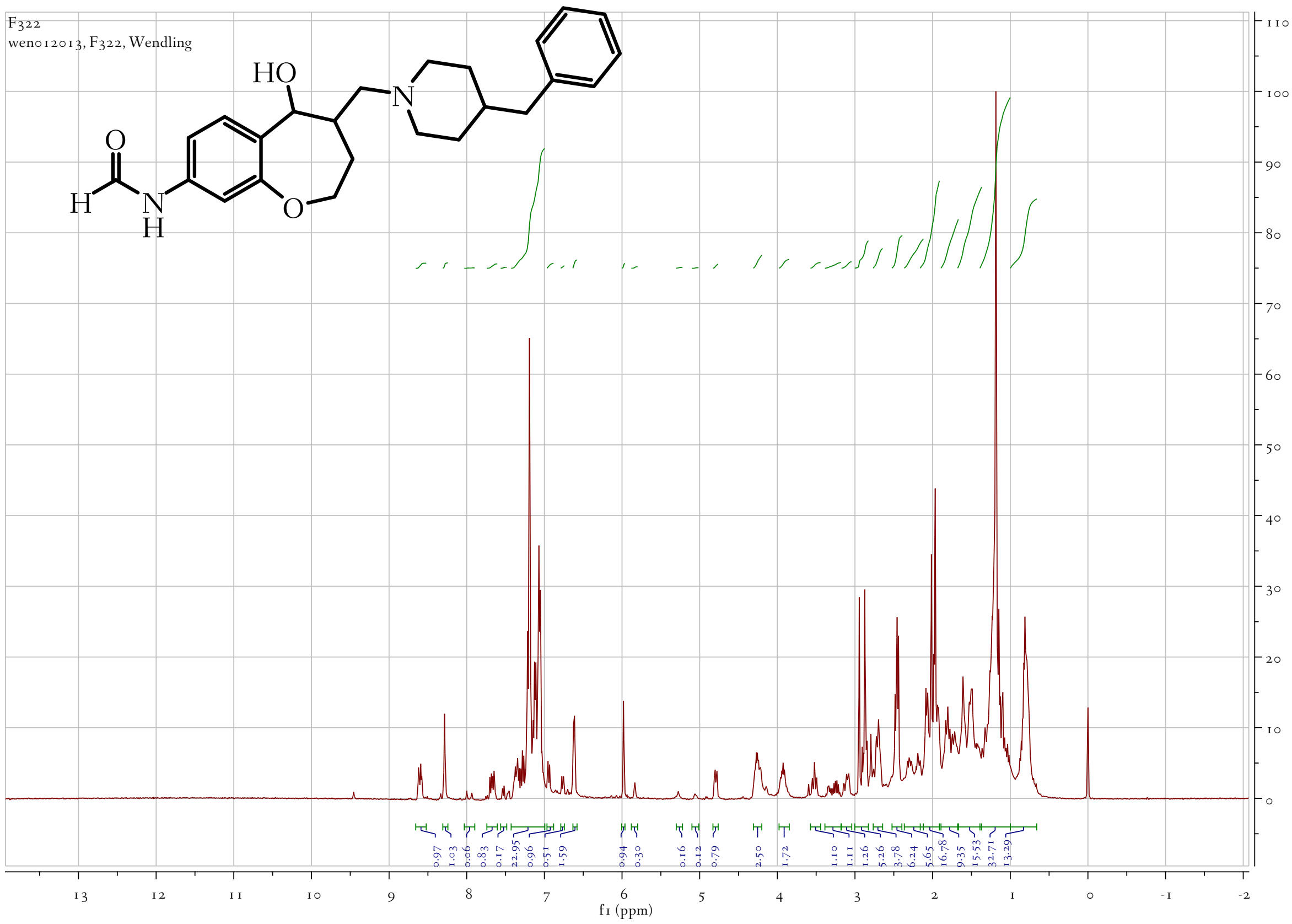

^{1}H NMR spectrum ($CDCl_3$) of acetamide **14a**

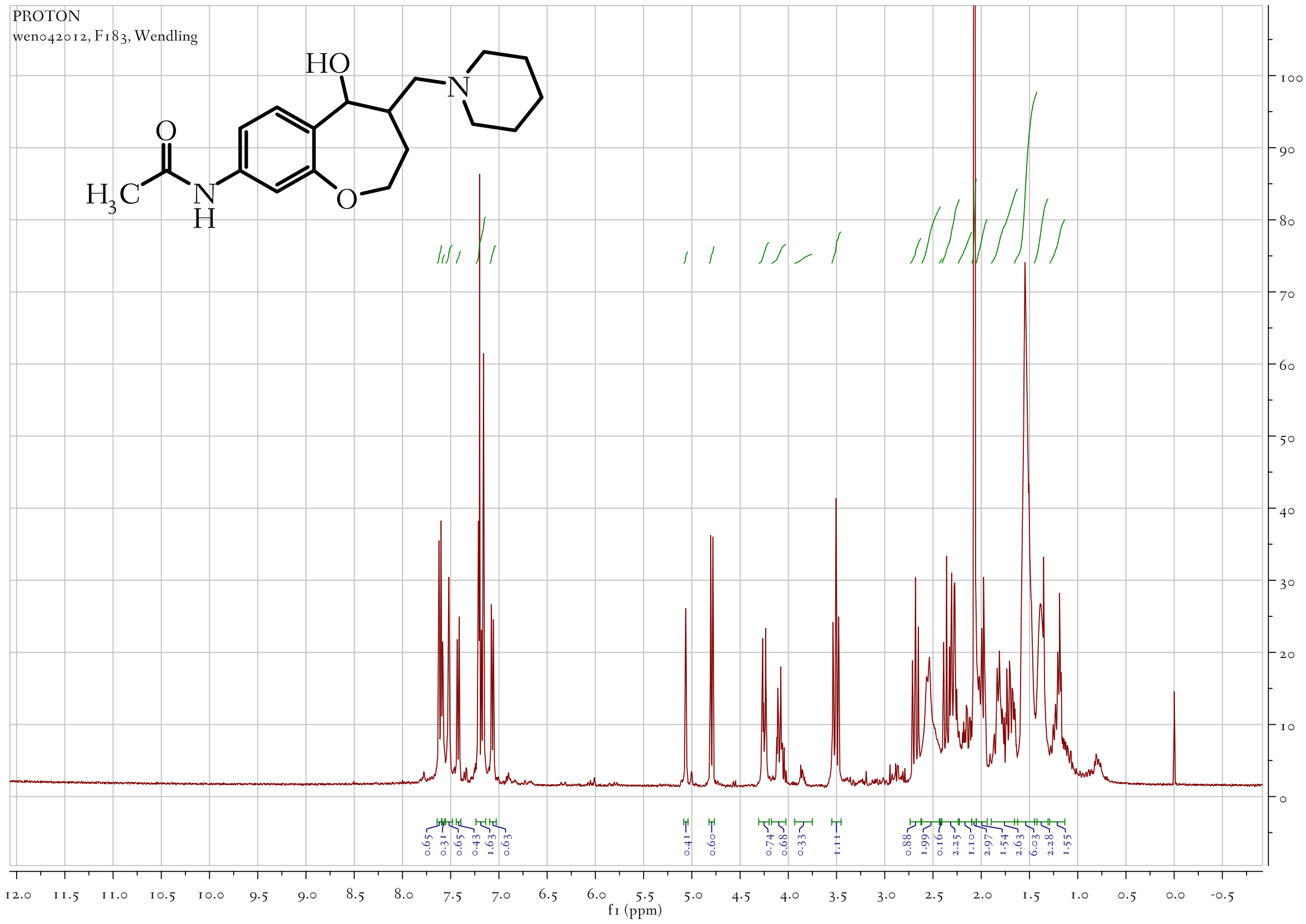

^{1}H and ^{13}C NMR spectrum ($CDCl_3$) of acetamide **14b**

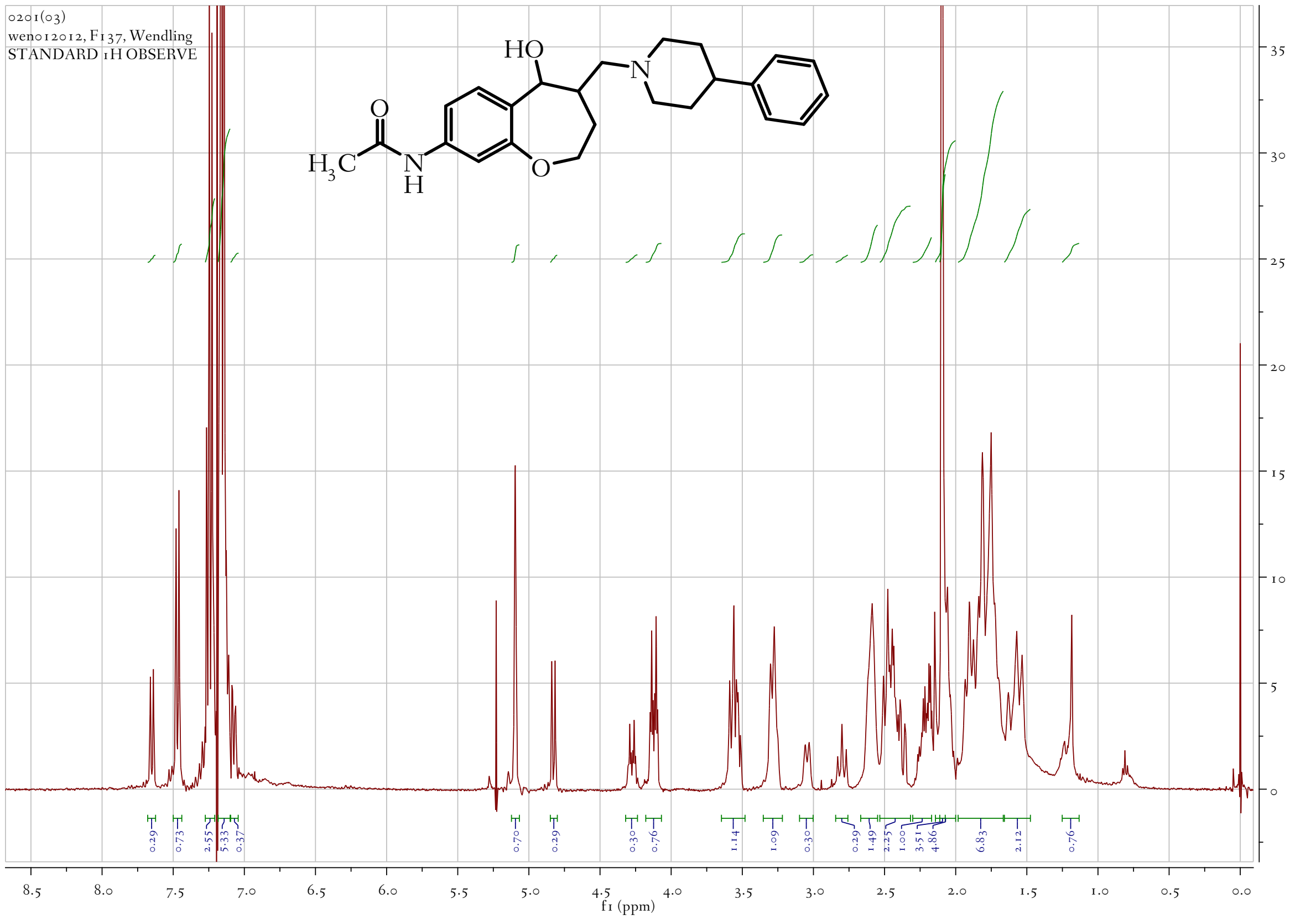

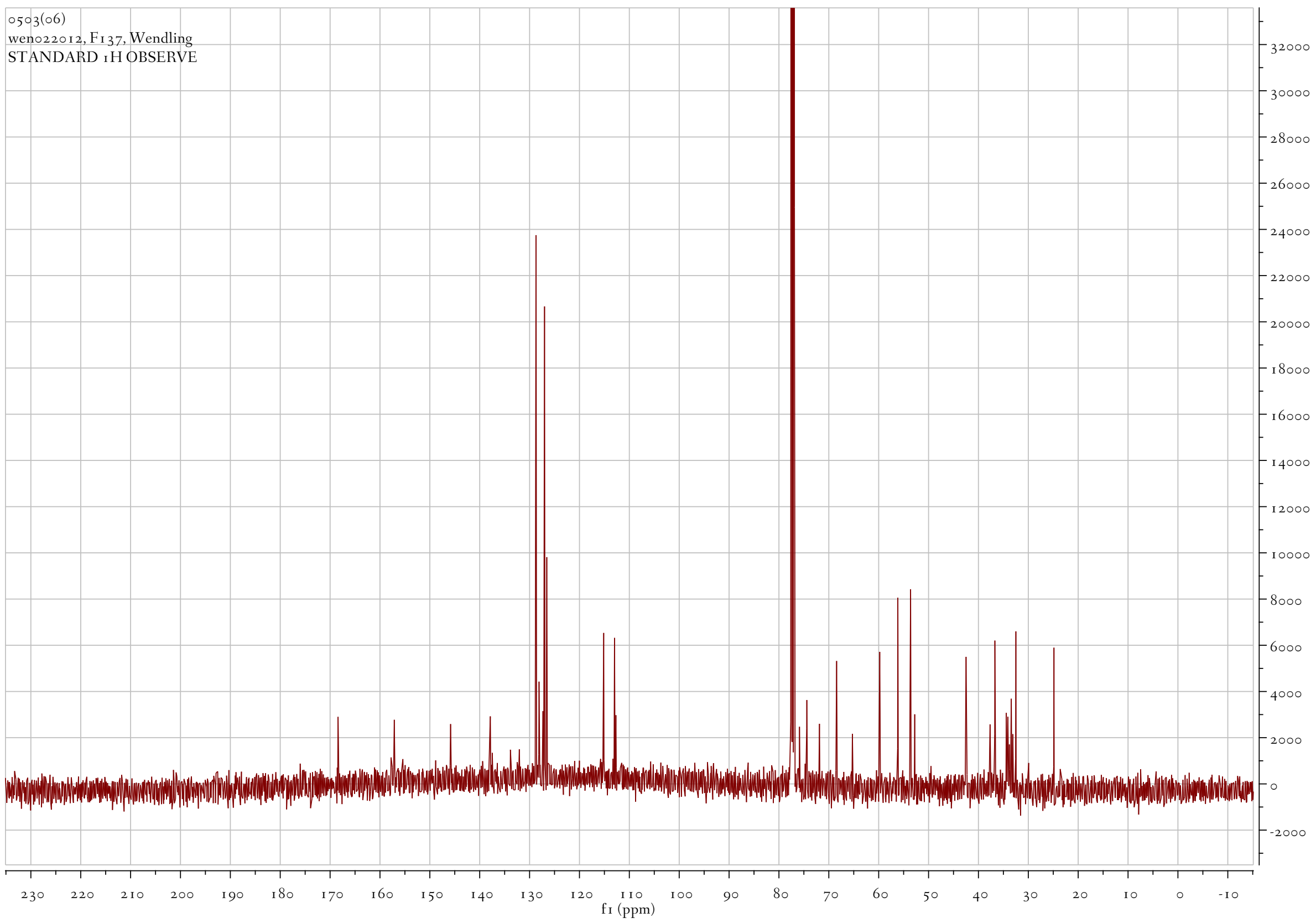

^{1}H and ^{13}C NMR spectrum ($CDCl_3$) of acetamide **14c**

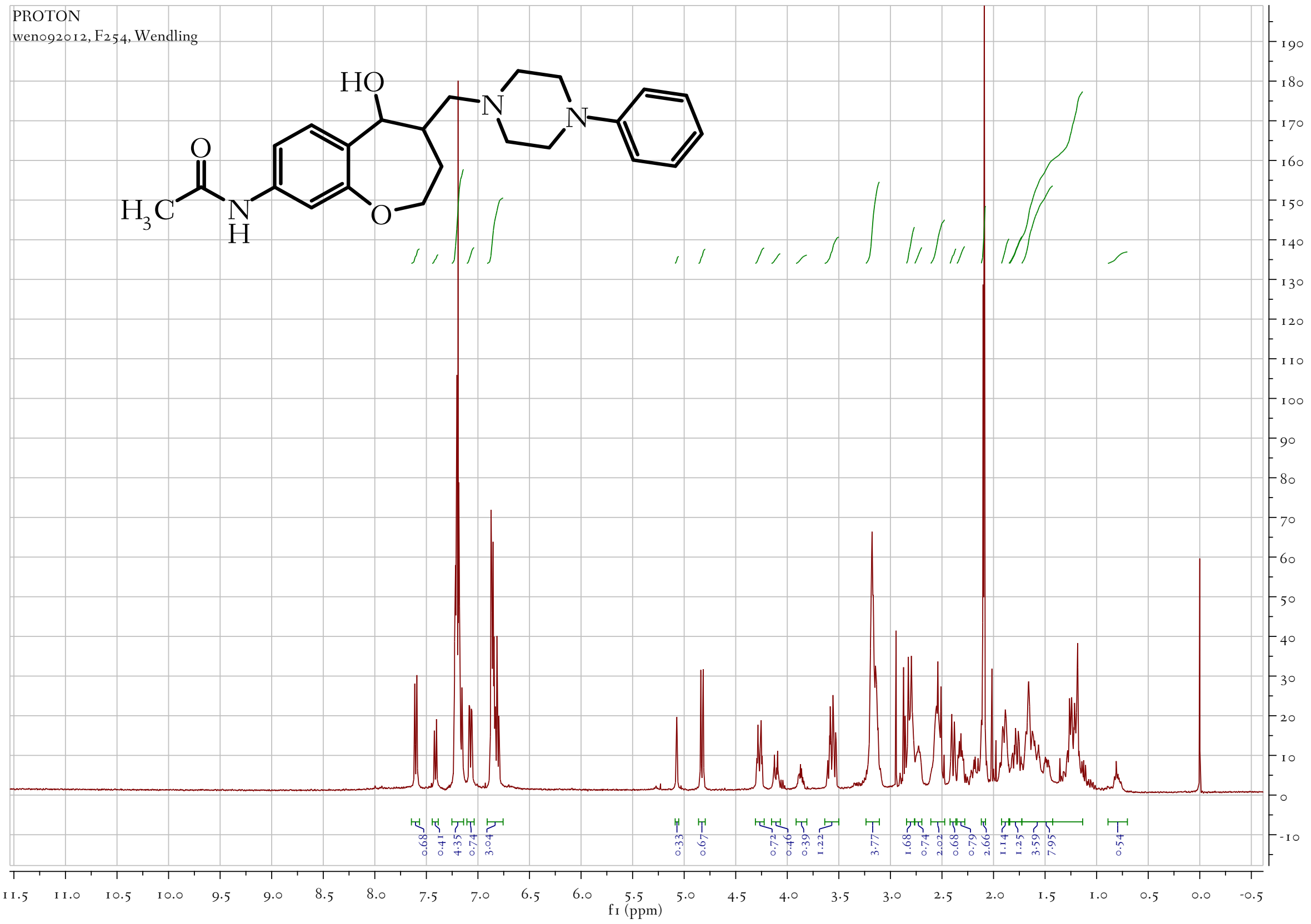

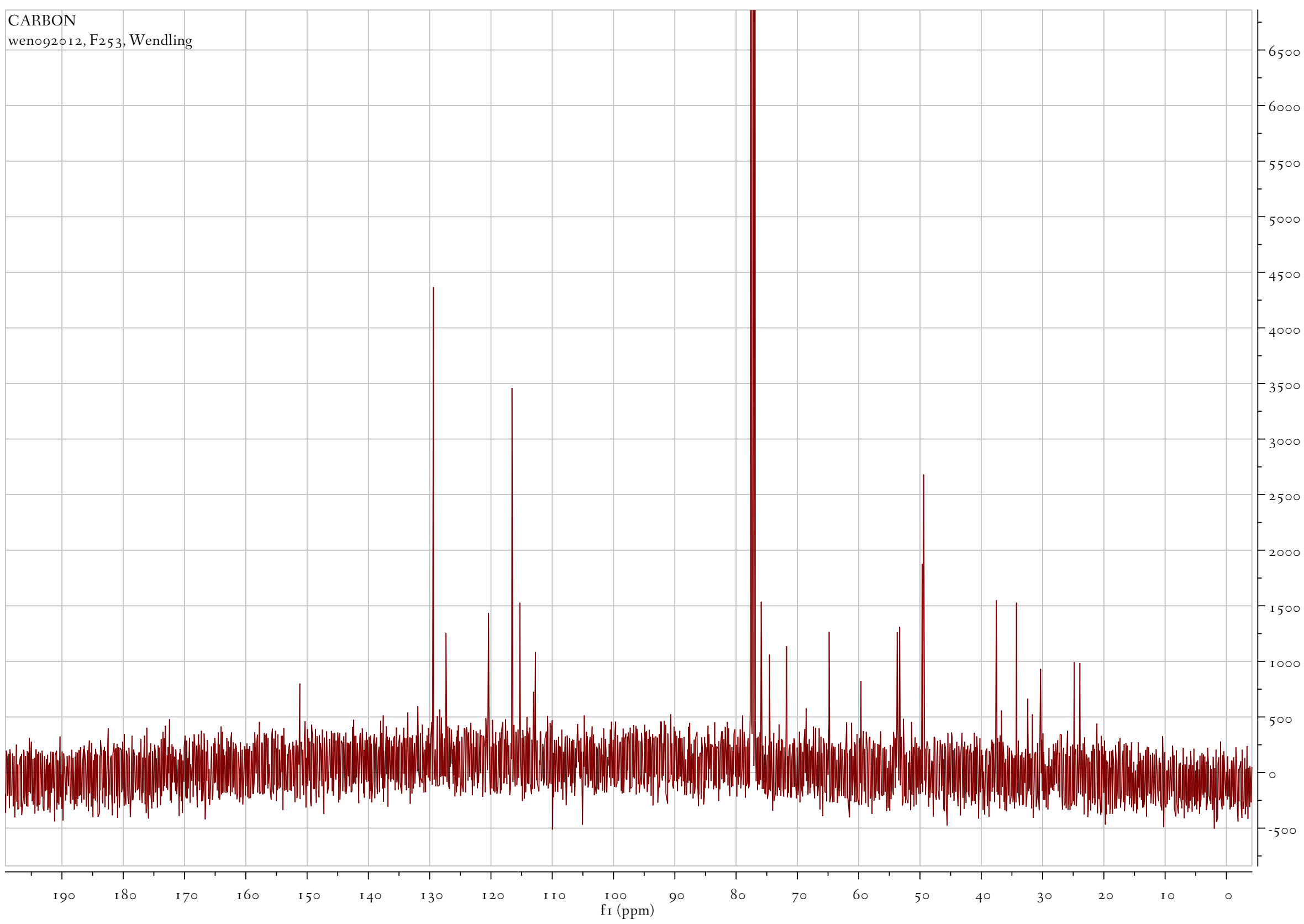

^{1}H NMR spectrum ($CDCl_3$) of acetamide **14d**

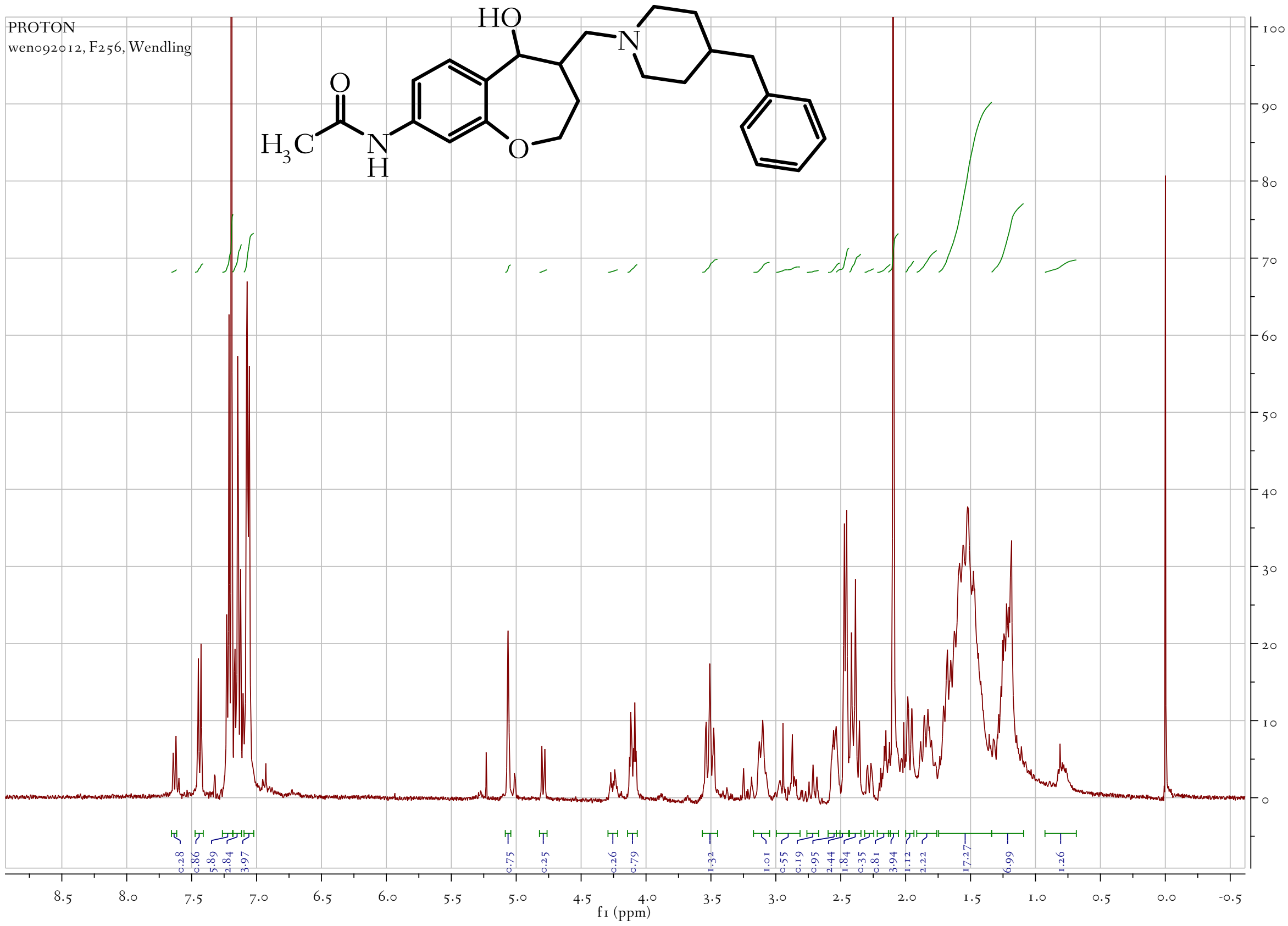

^{1}H and ^{13}C NMR spectrum ($CDCl_3$) of isobutyramide **15a**

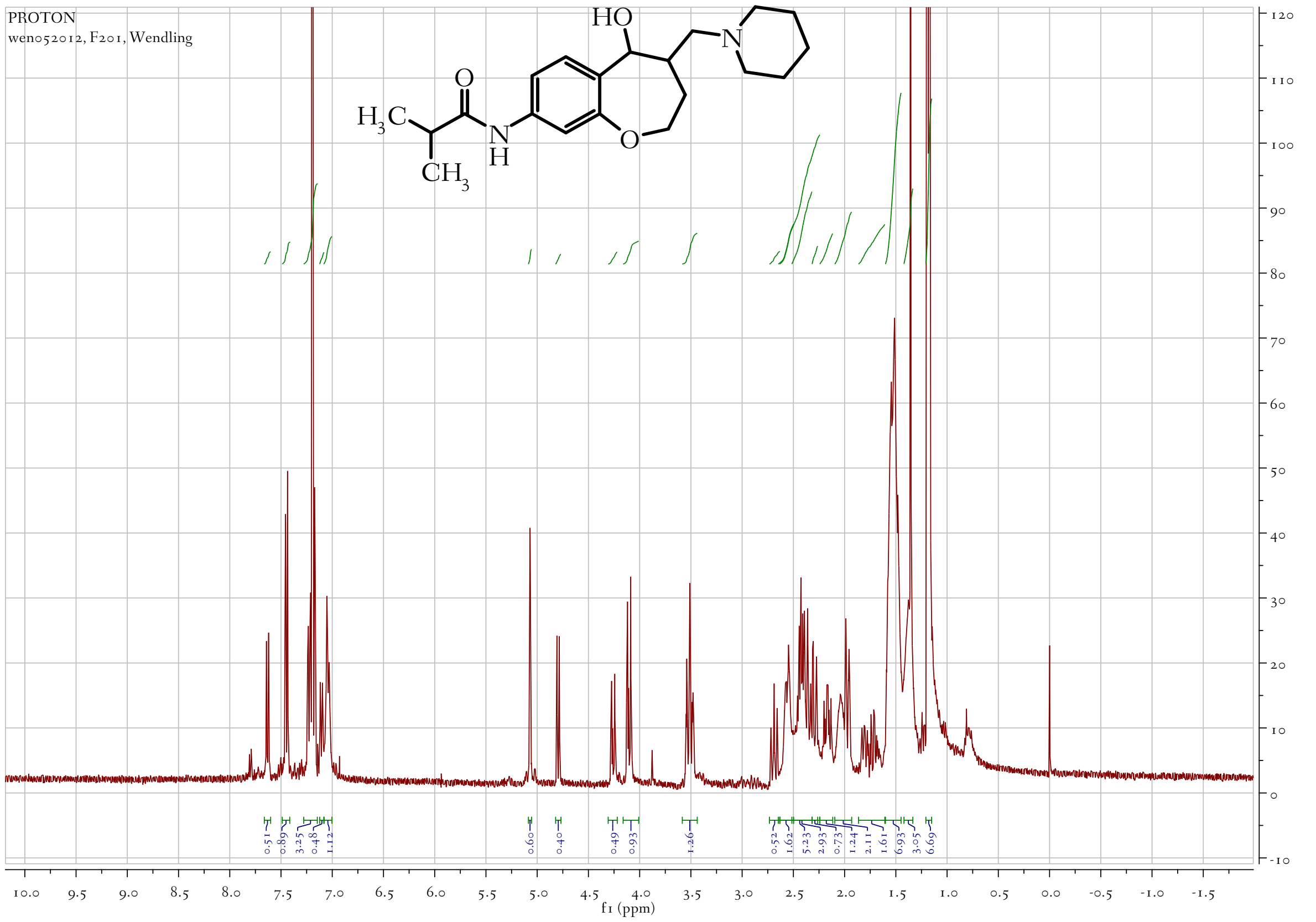

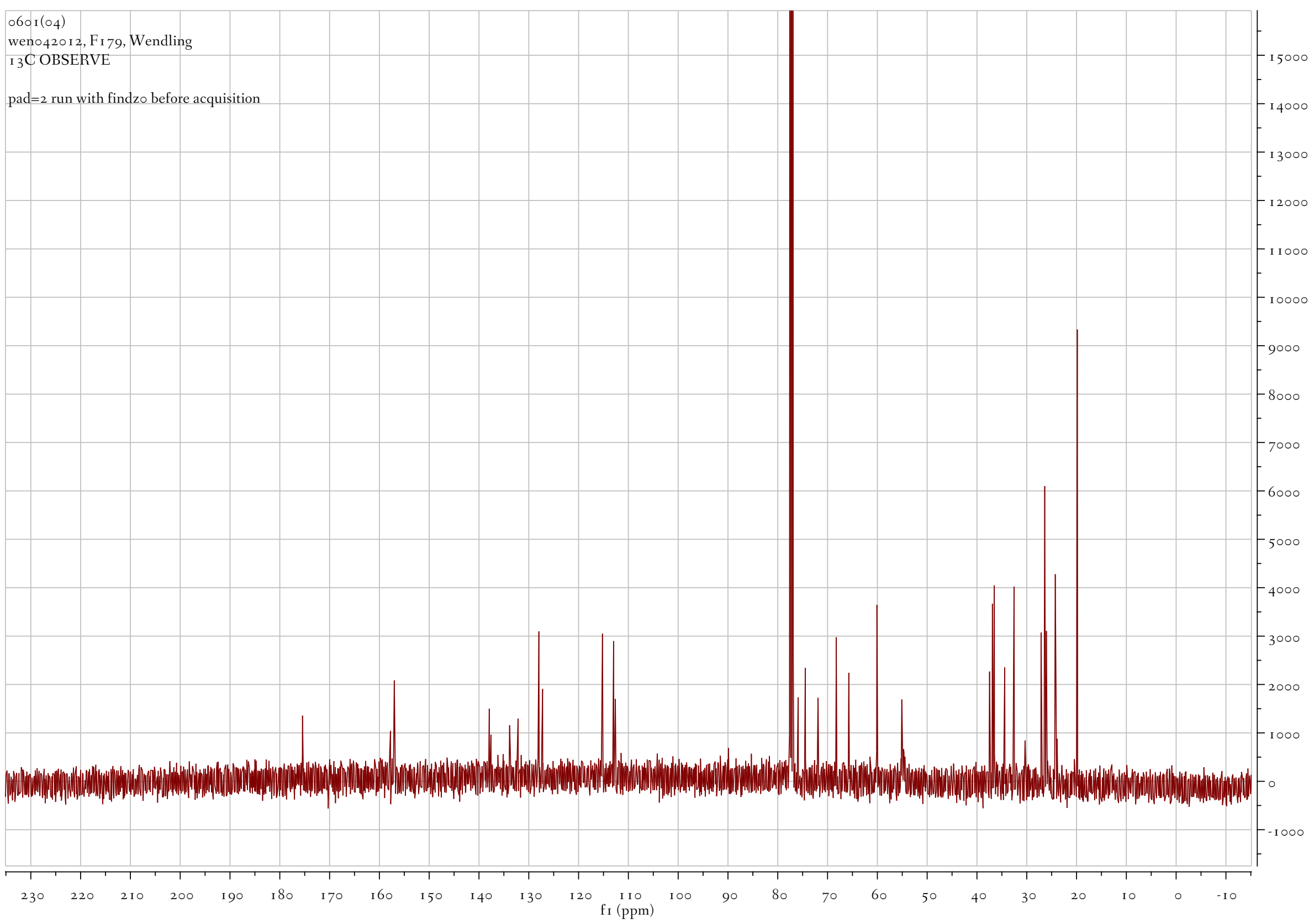

^{1}H and ^{13}C NMR spectrum ($CDCl_3$) of isobutyramide *trans*-**15a**

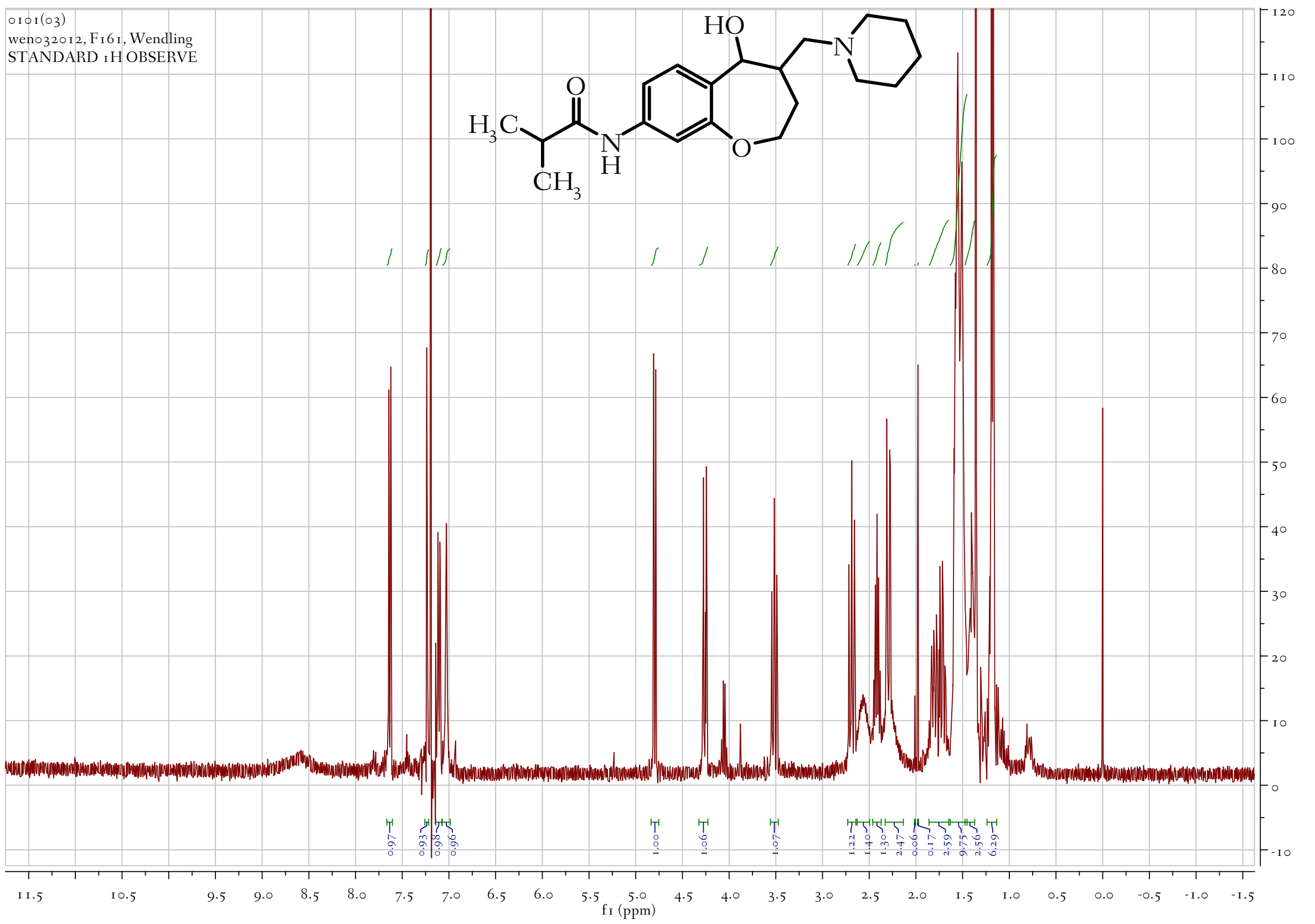

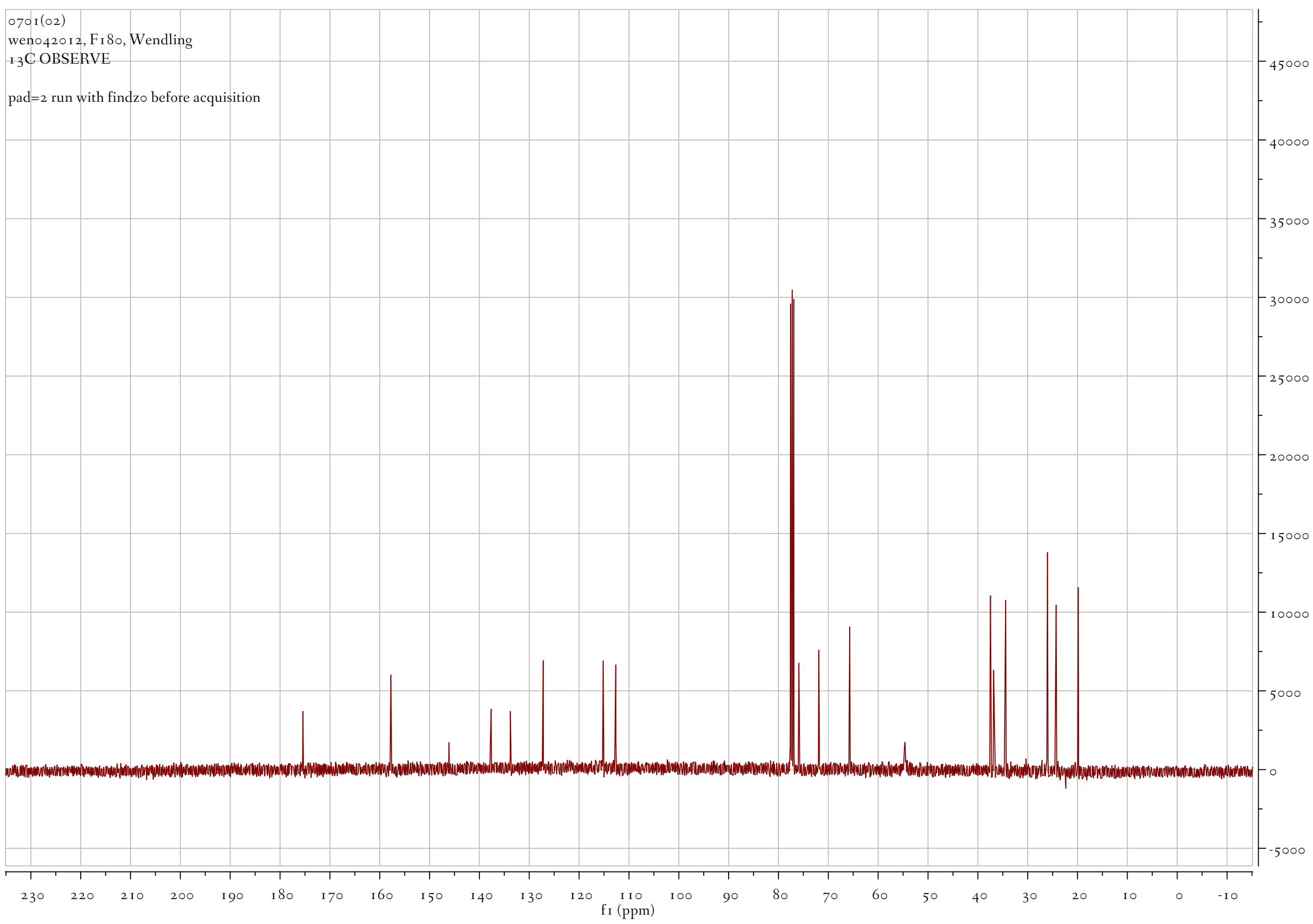

^{1}H and ^{13}C NMR spectrum ($CDCl_3$) of isobutyramide **15b**

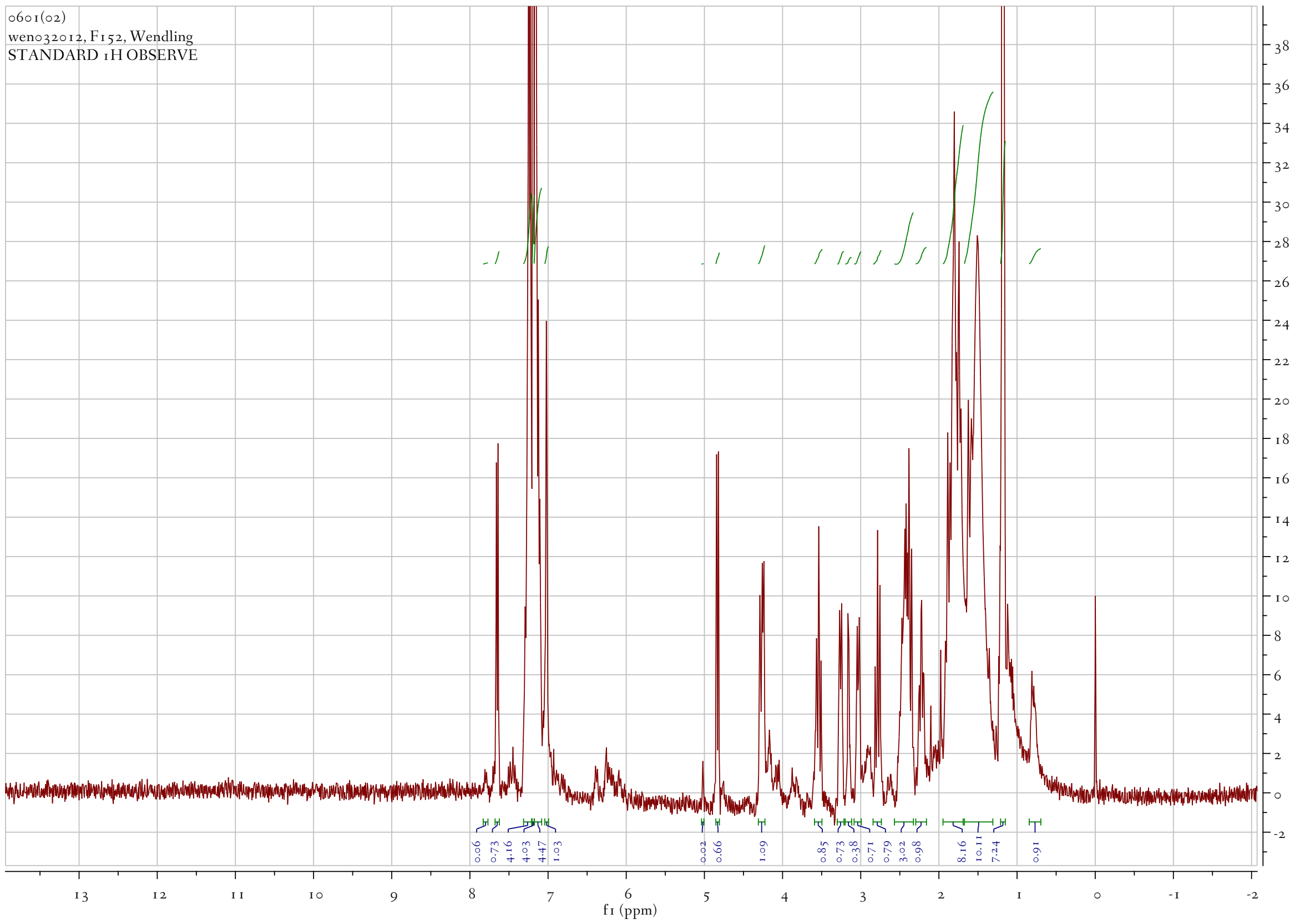

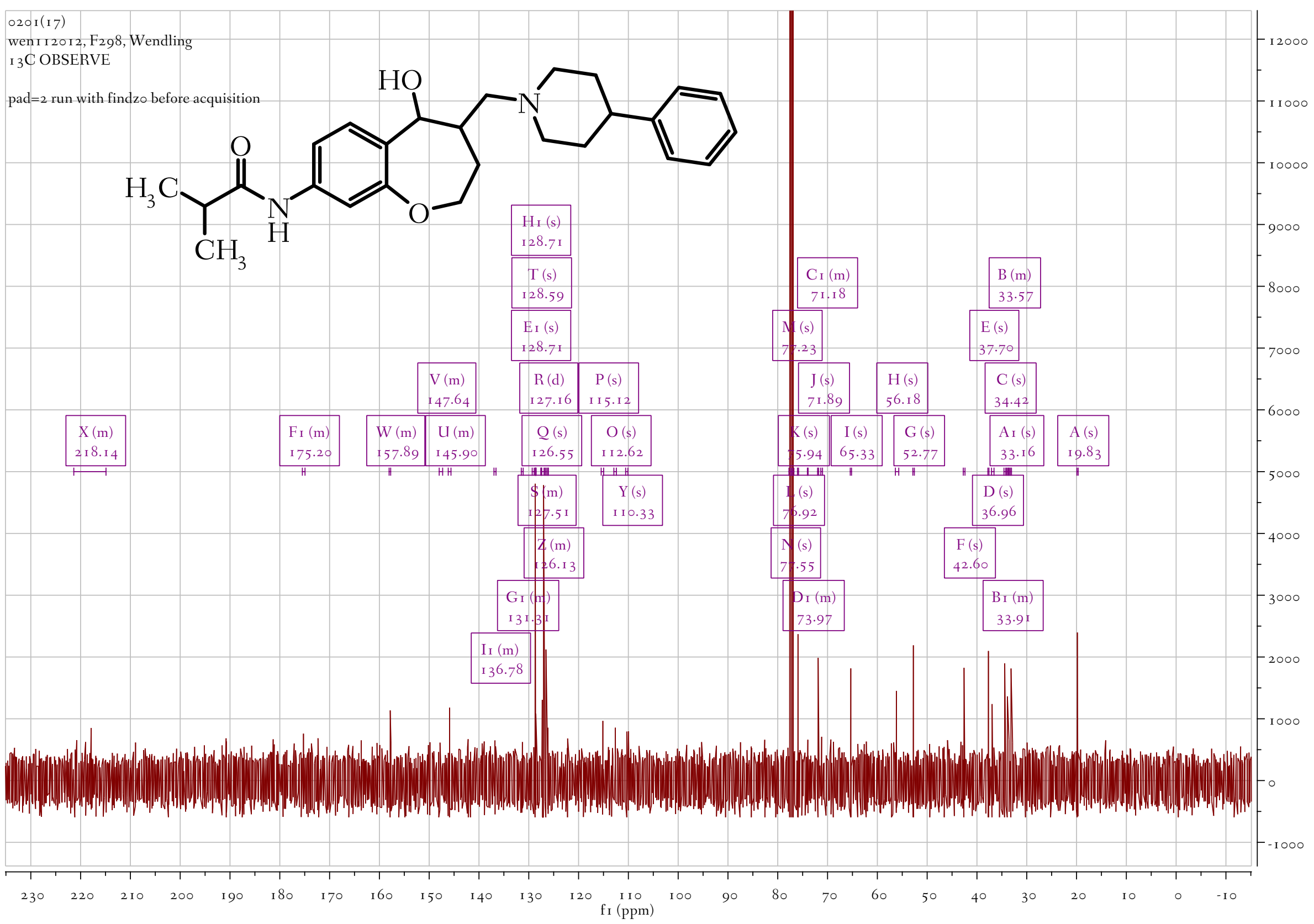

^{1}H and ^{13}C NMR spectrum ($CDCl_3$) of isobutyramide **15d**

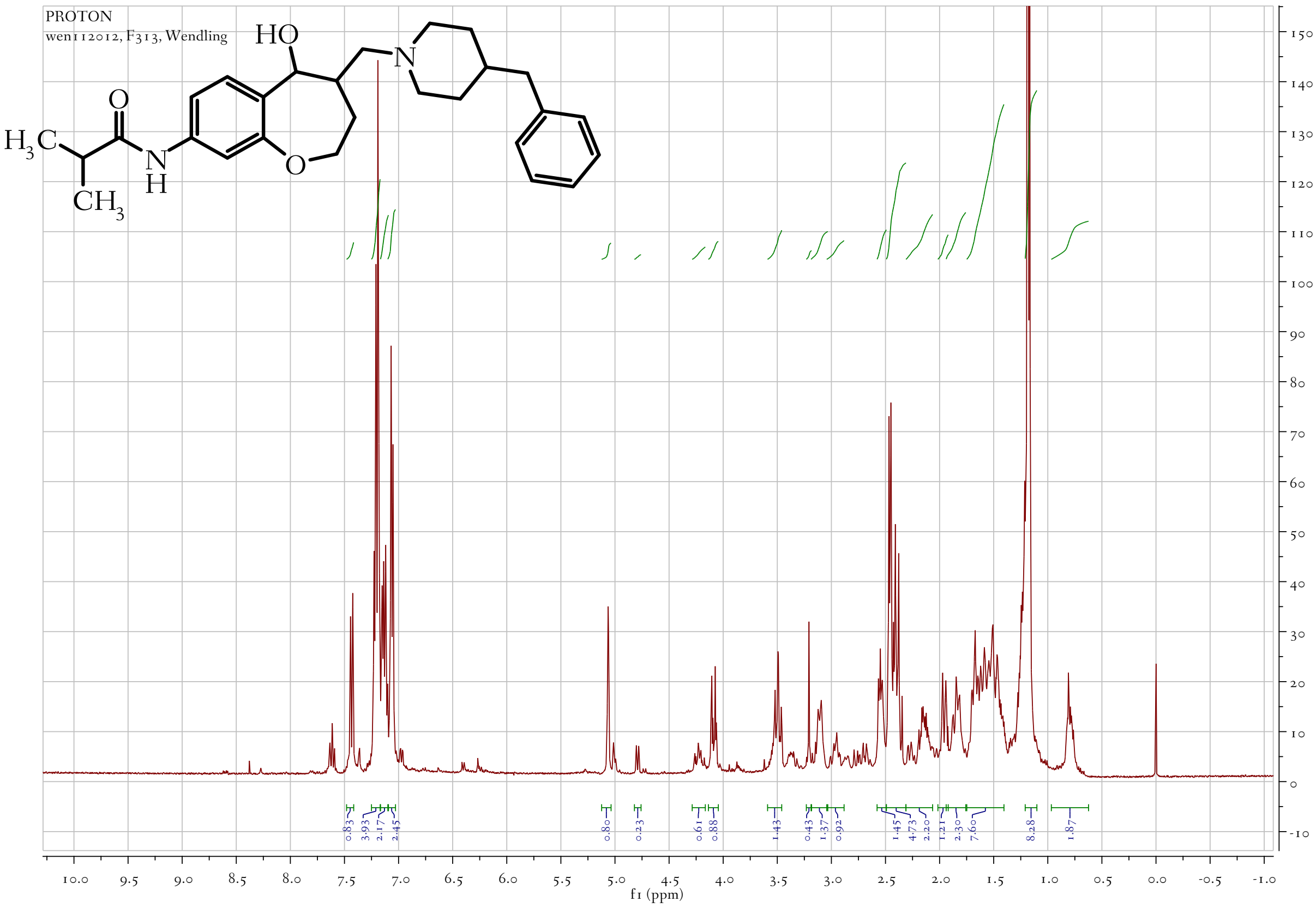

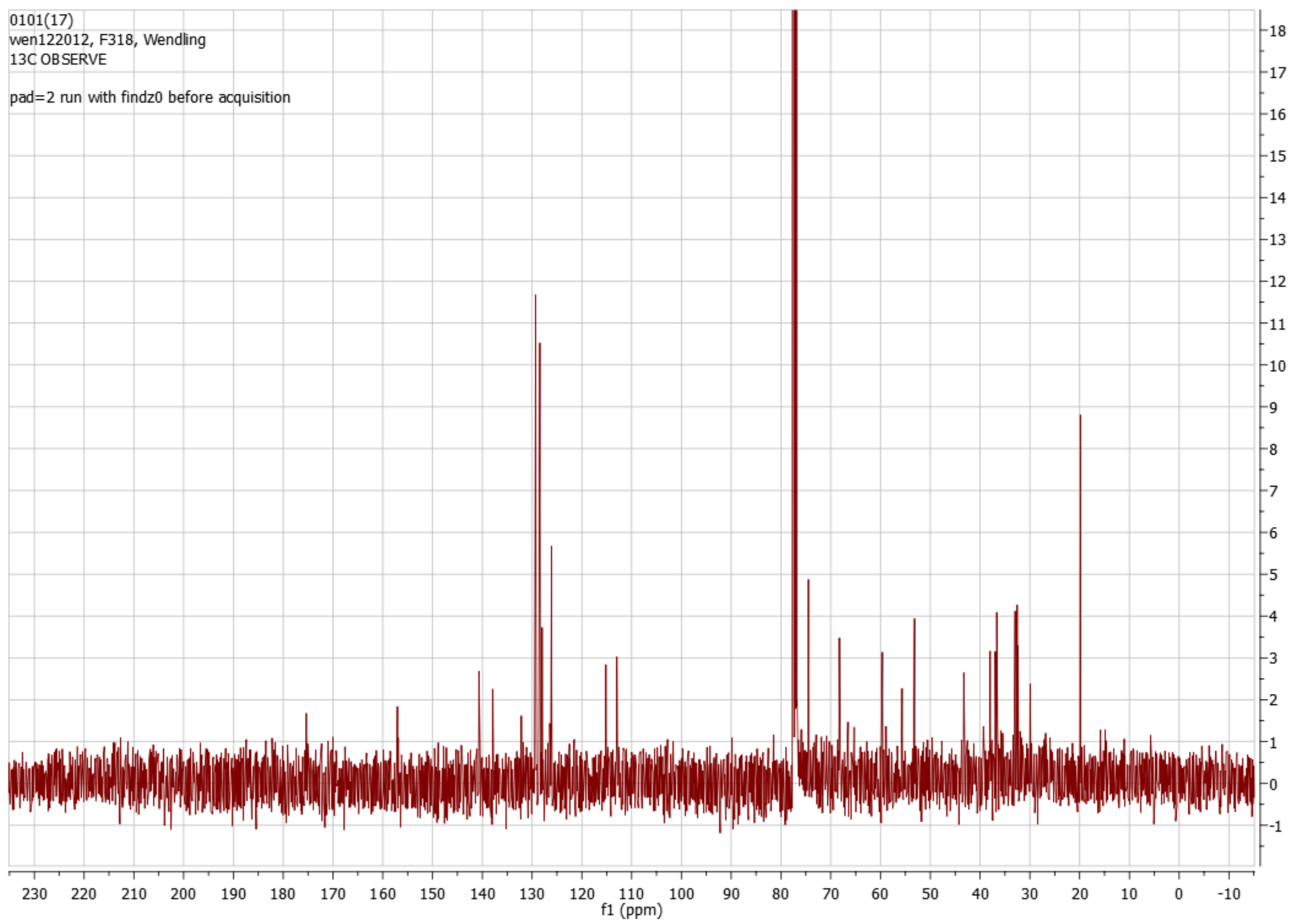

^{1}H and ^{13}C NMR spectrum ($CDCl_3$) of benzamide **16b**

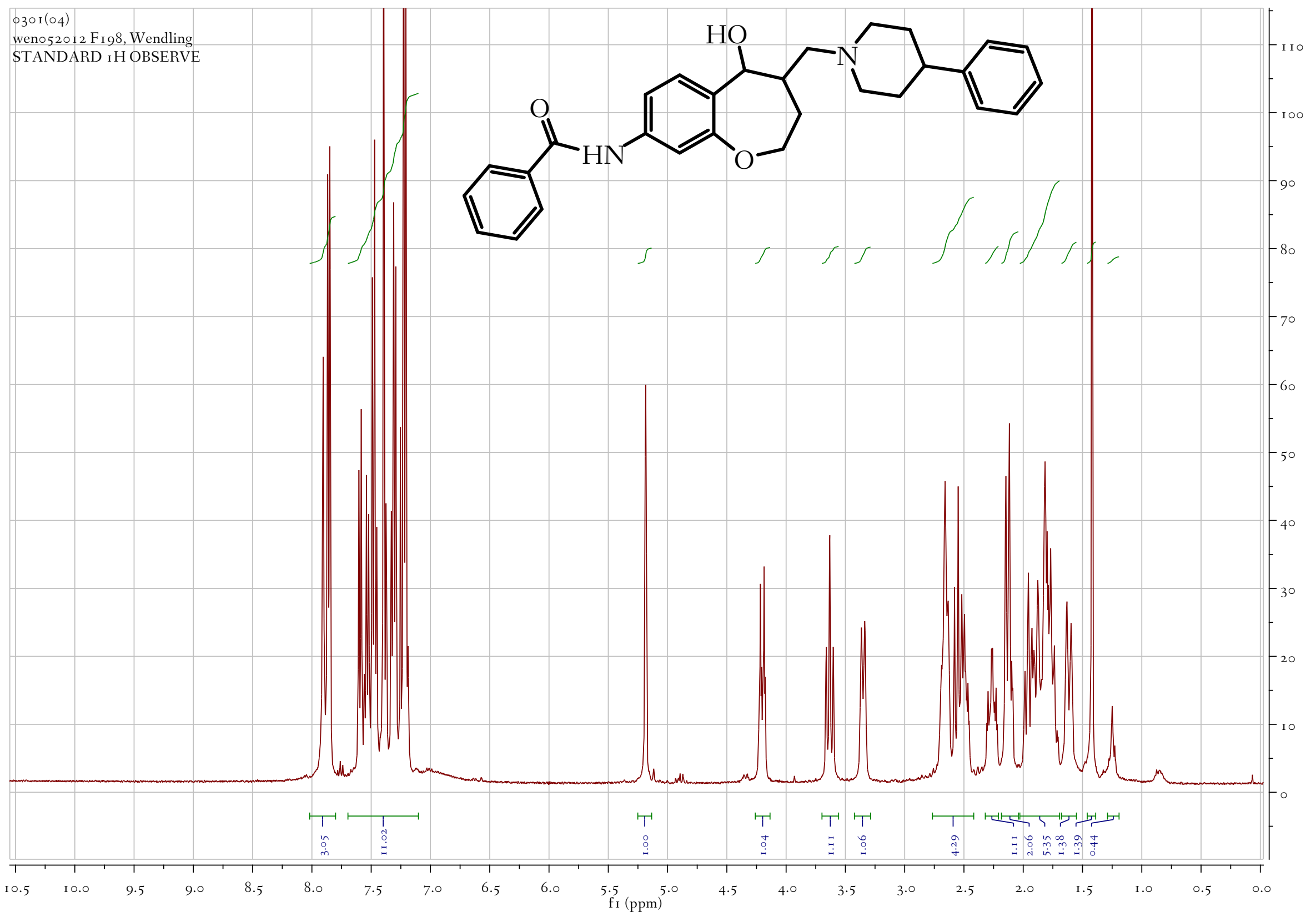

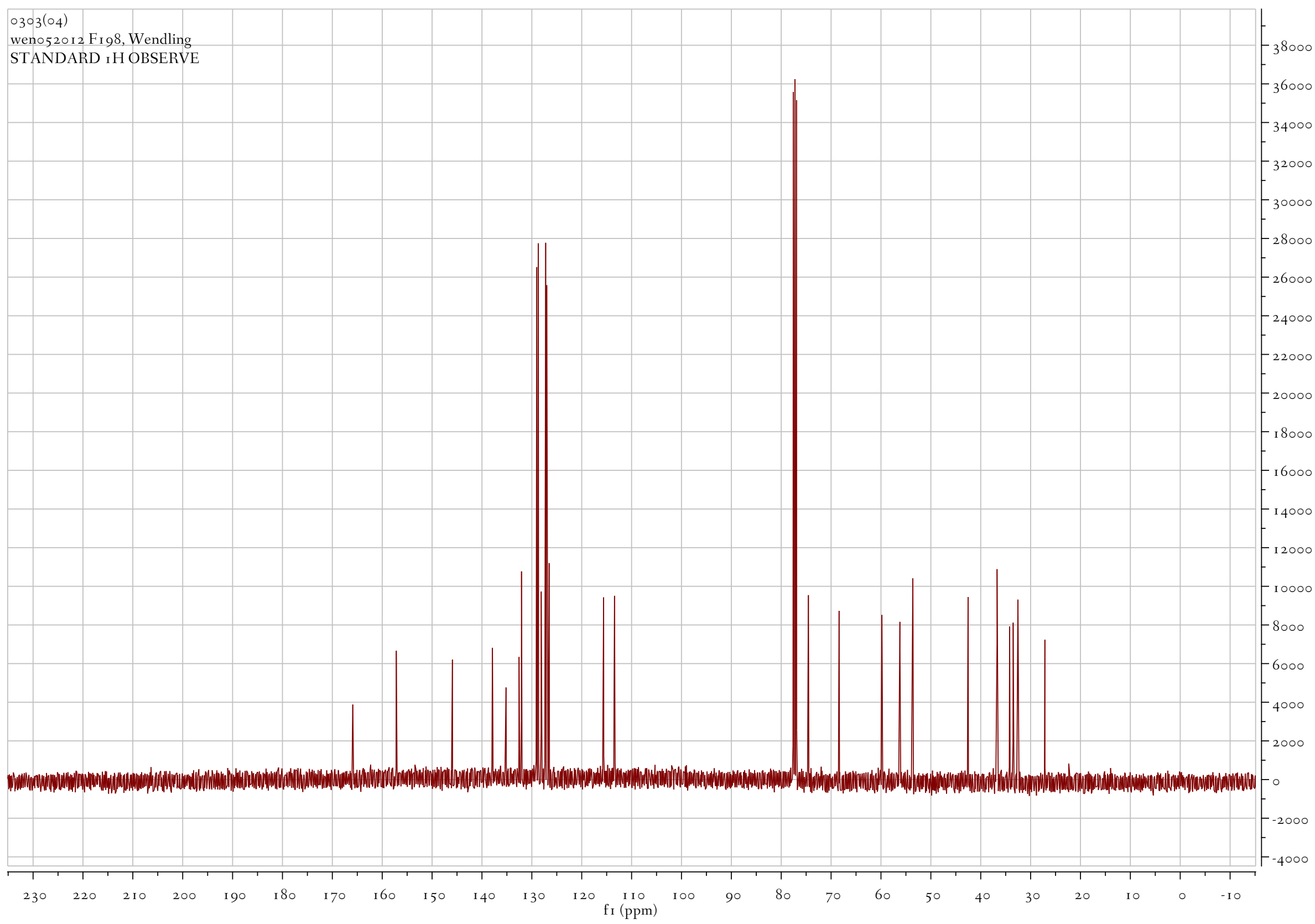

^{1}H and ^{13}C NMR spectrum ($CDCl_3$) of allylamine **17b**

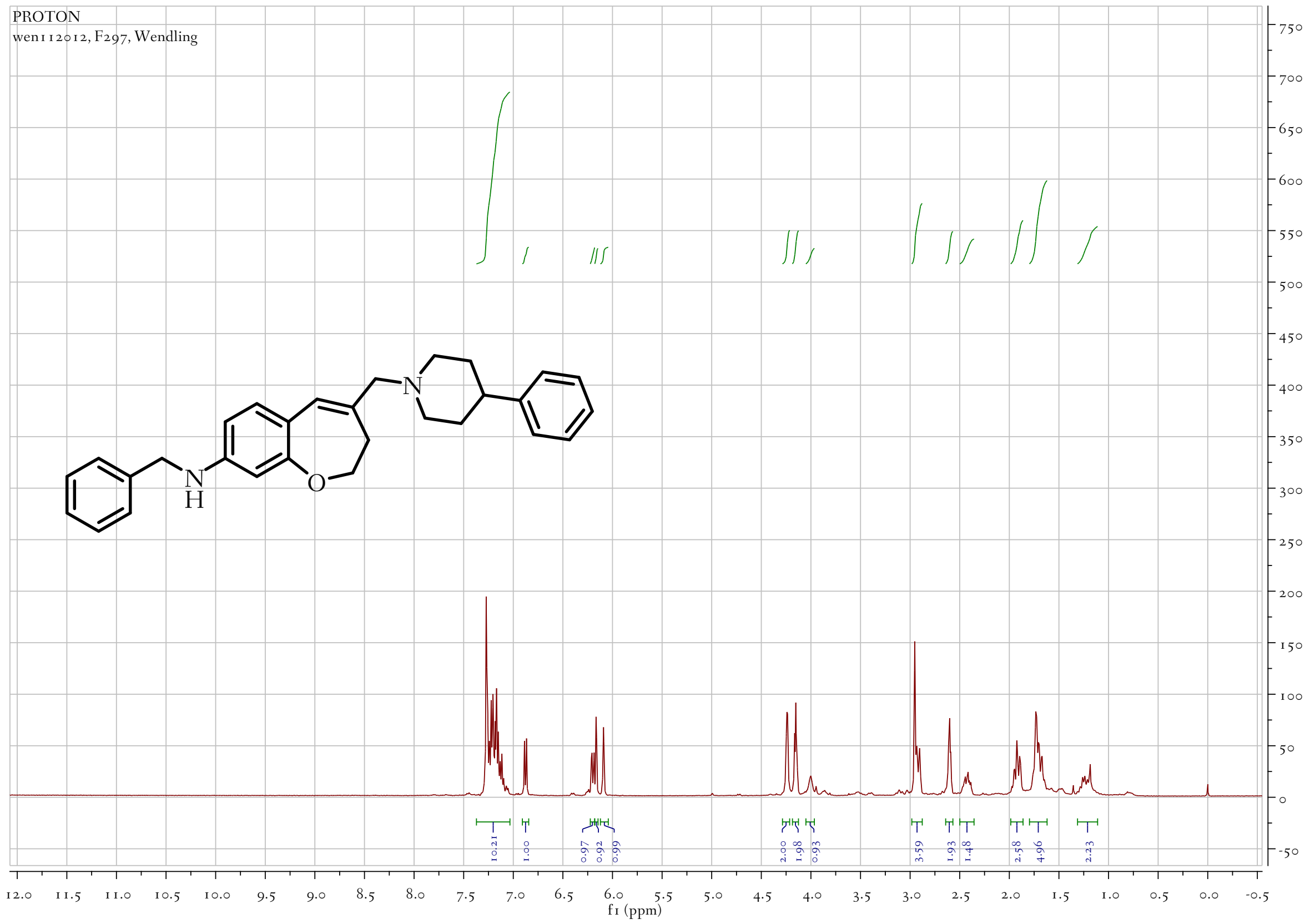

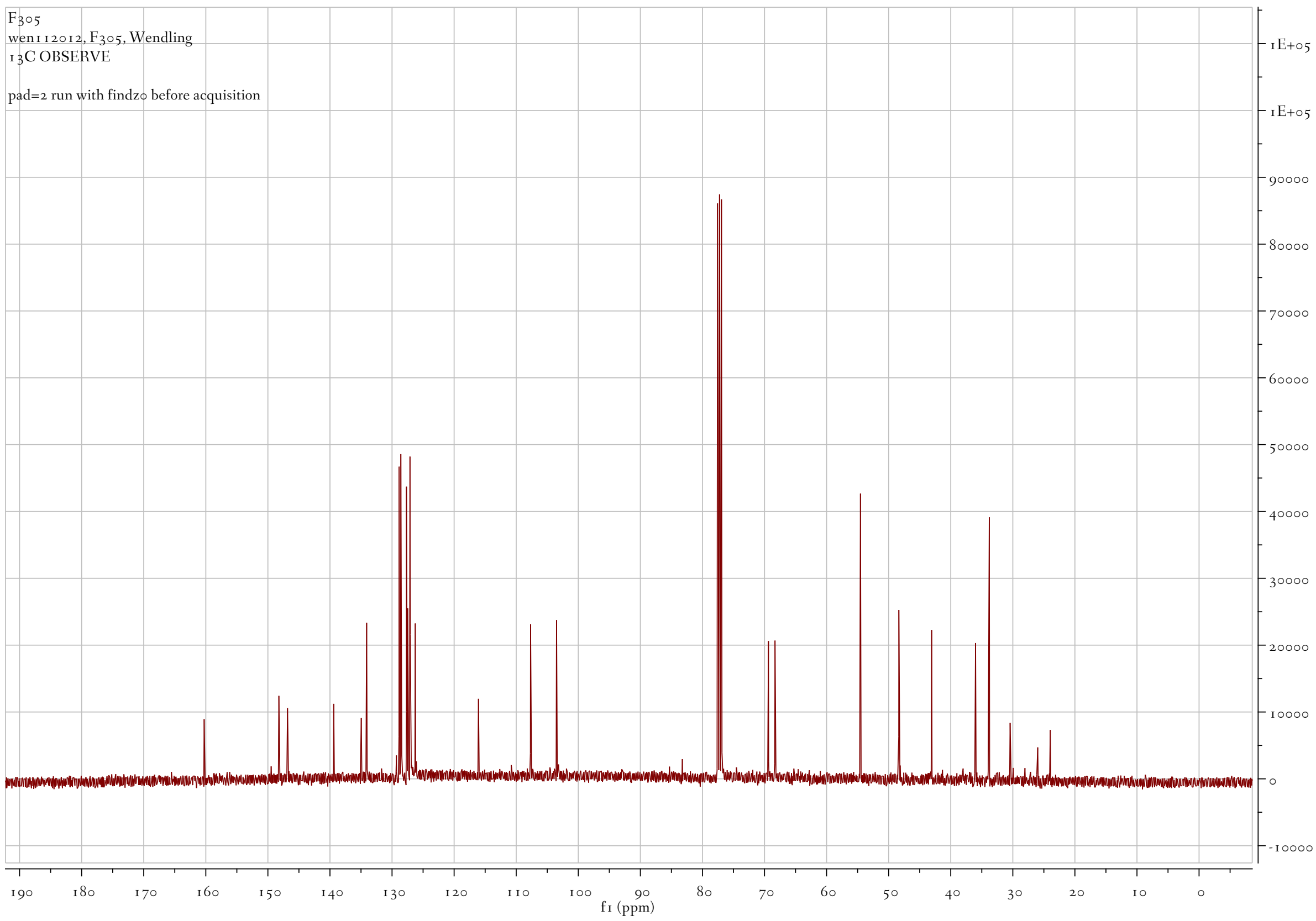

^{1}H and ^{13}C NMR spectrum ($CDCl_3$) of allylamine **17d**

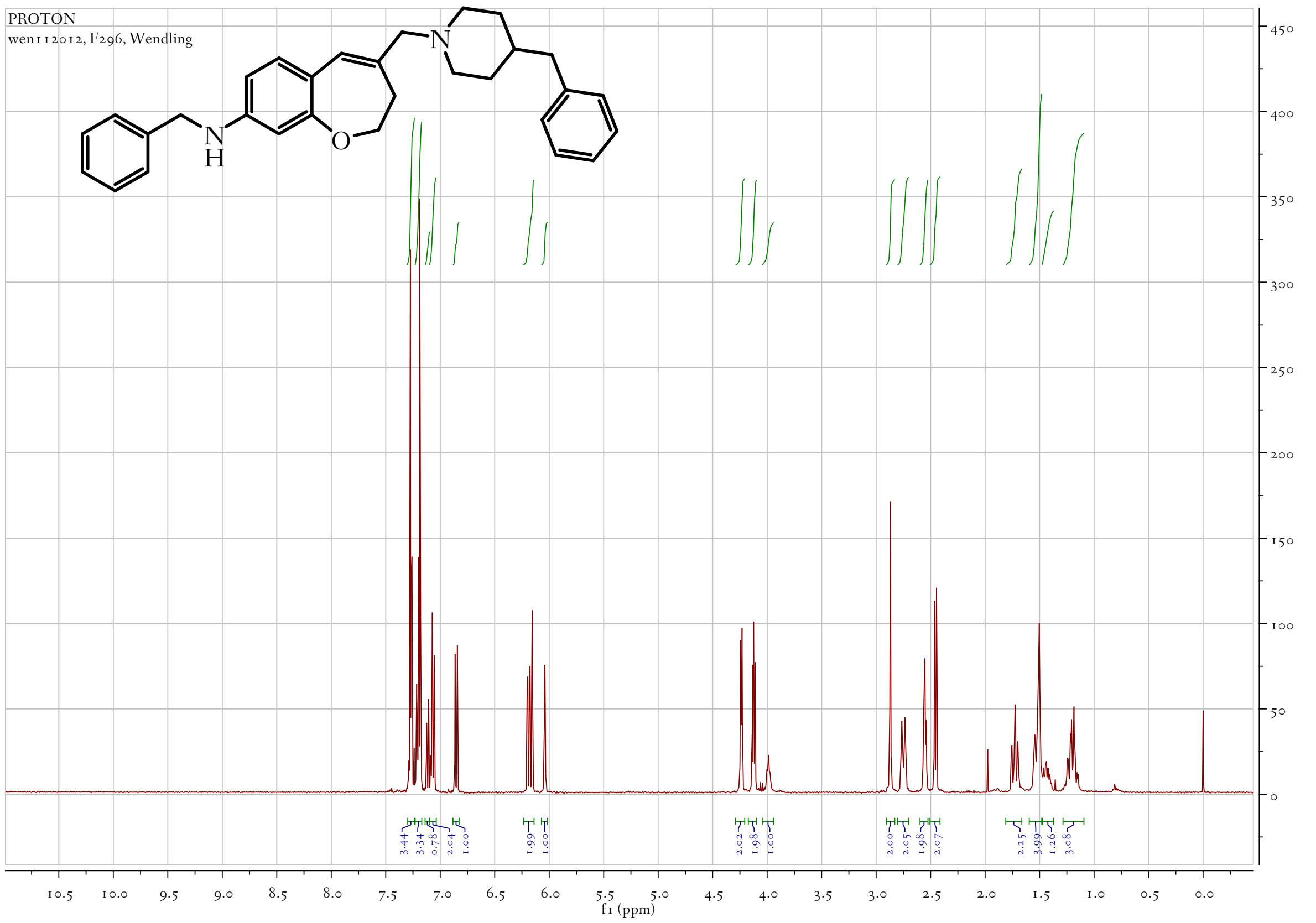

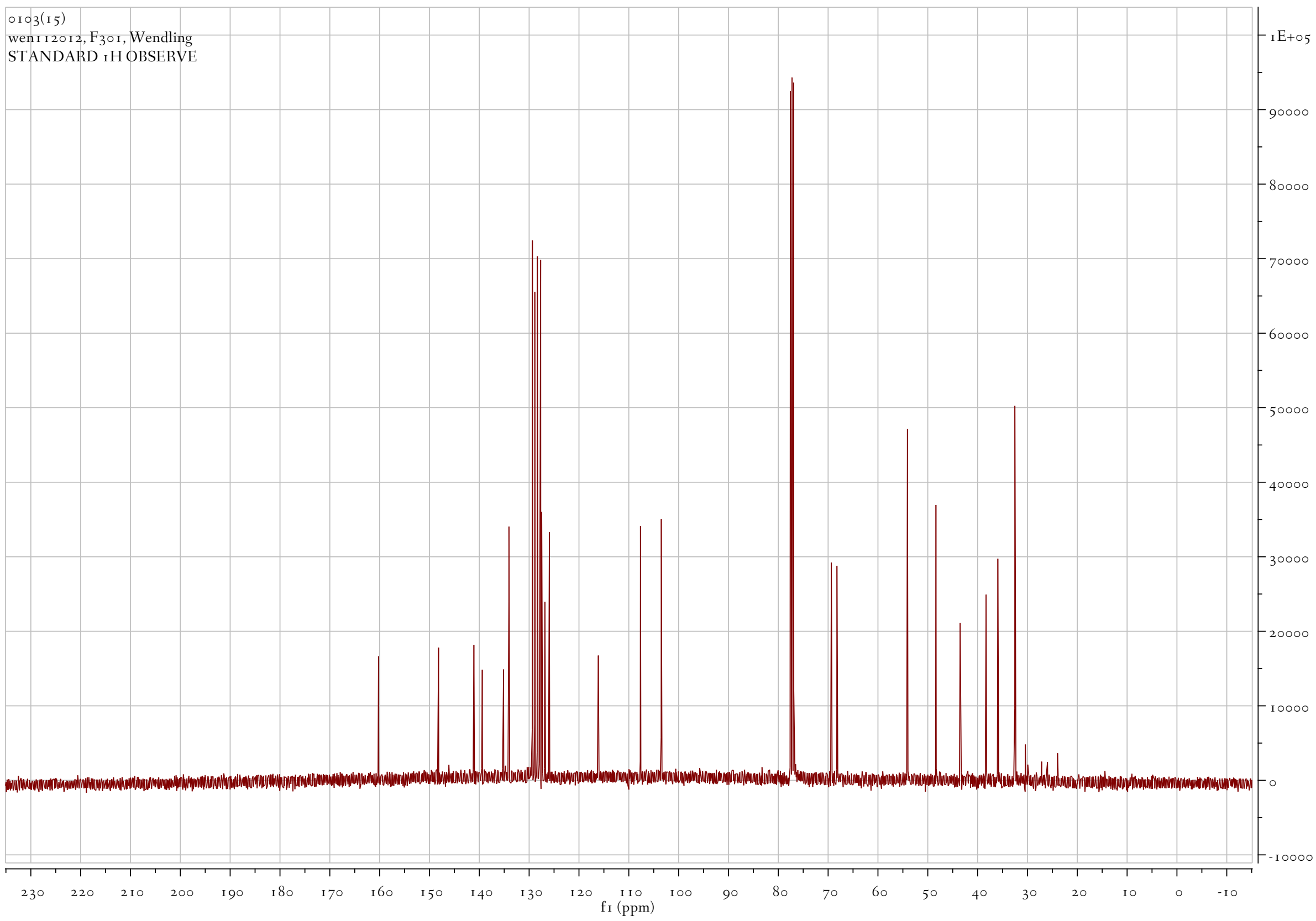

^{1}H and ^{13}C NMR spectrum ($CDCl_3$) of allylamine **18b**

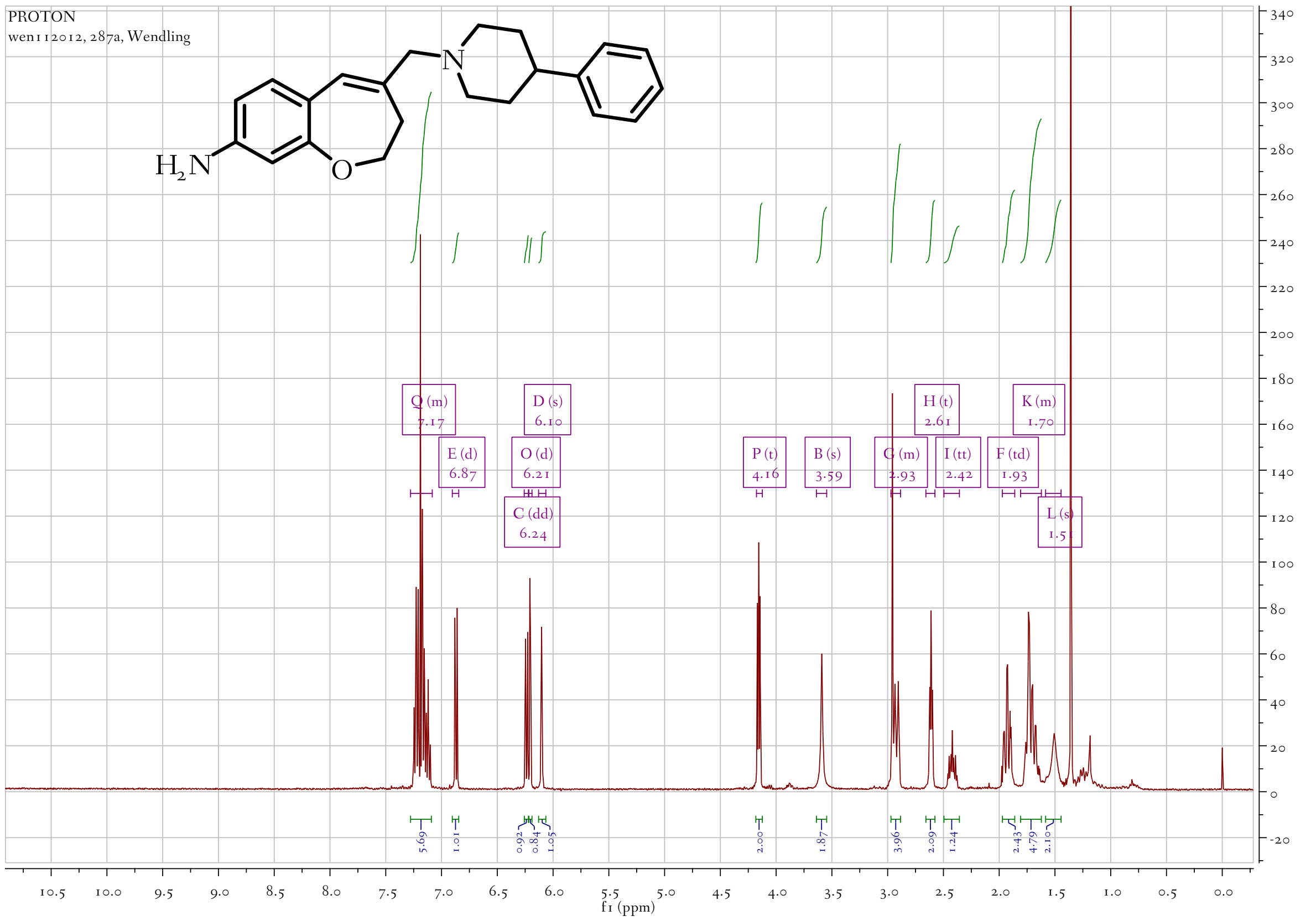

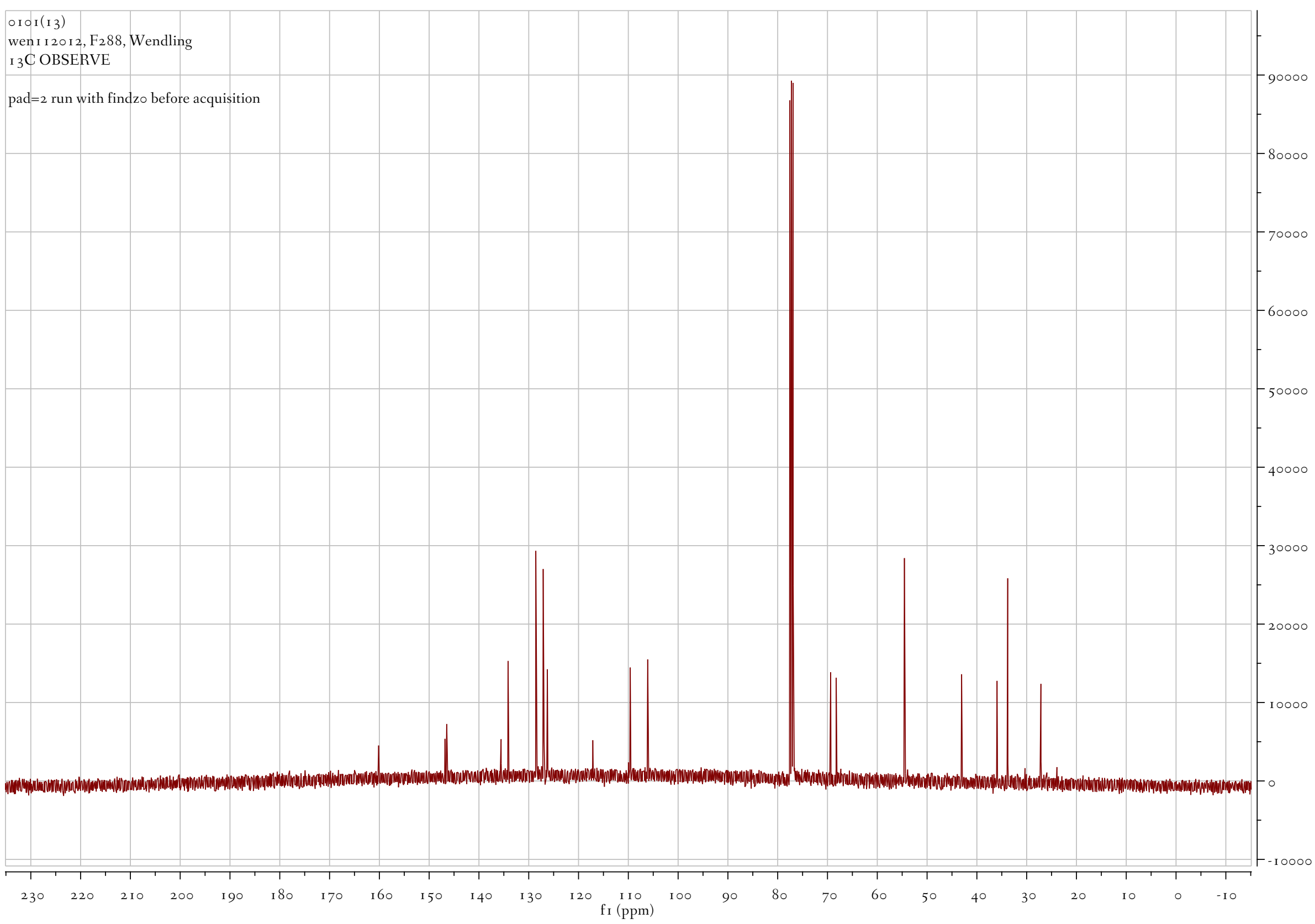

^{1}H NMR spectrum ($CDCl_3$) of allylamine **18d**

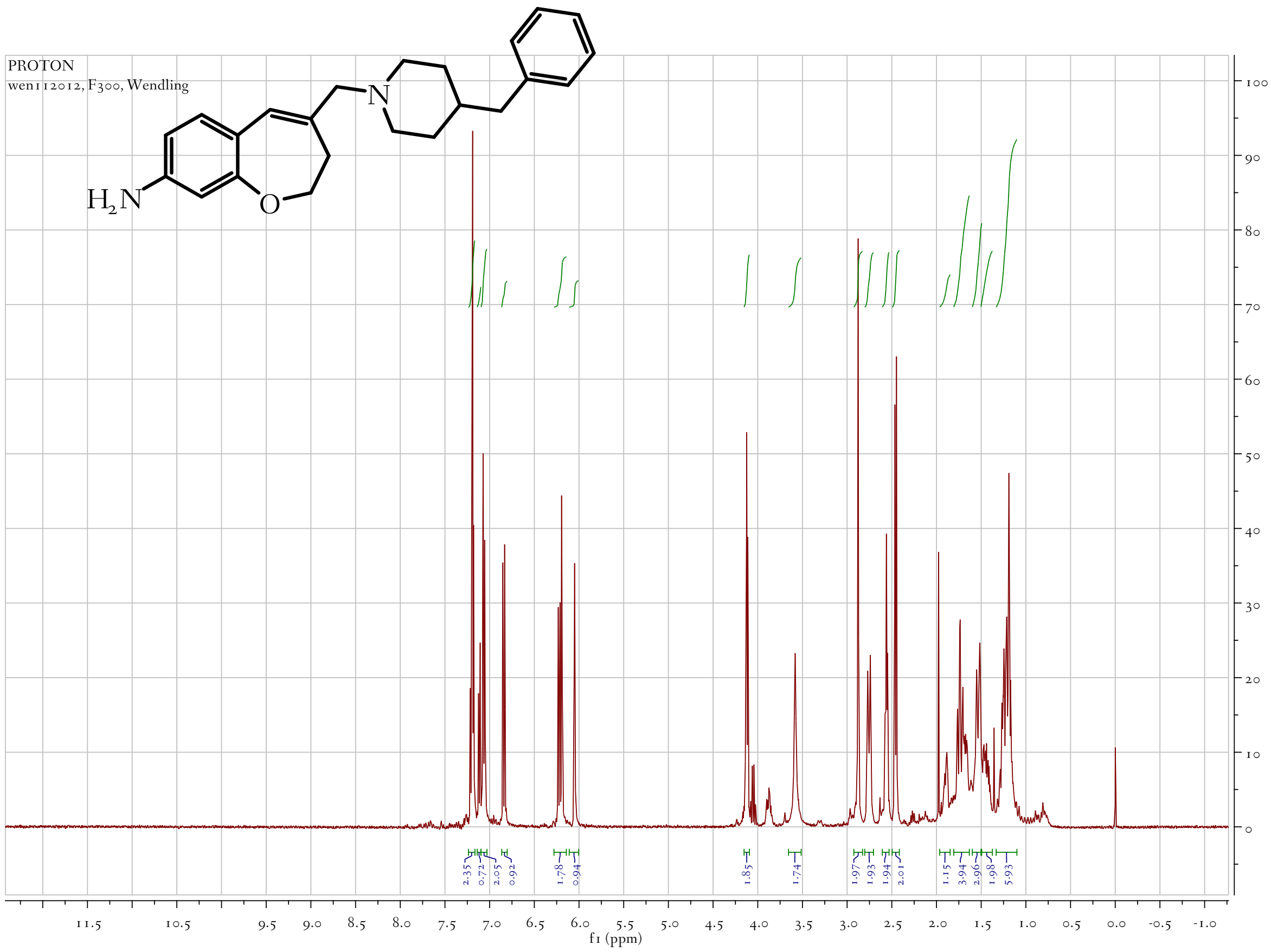

^{1}H and ^{13}C NMR spectrum ($CDCl_3$) of allylamine **19b**

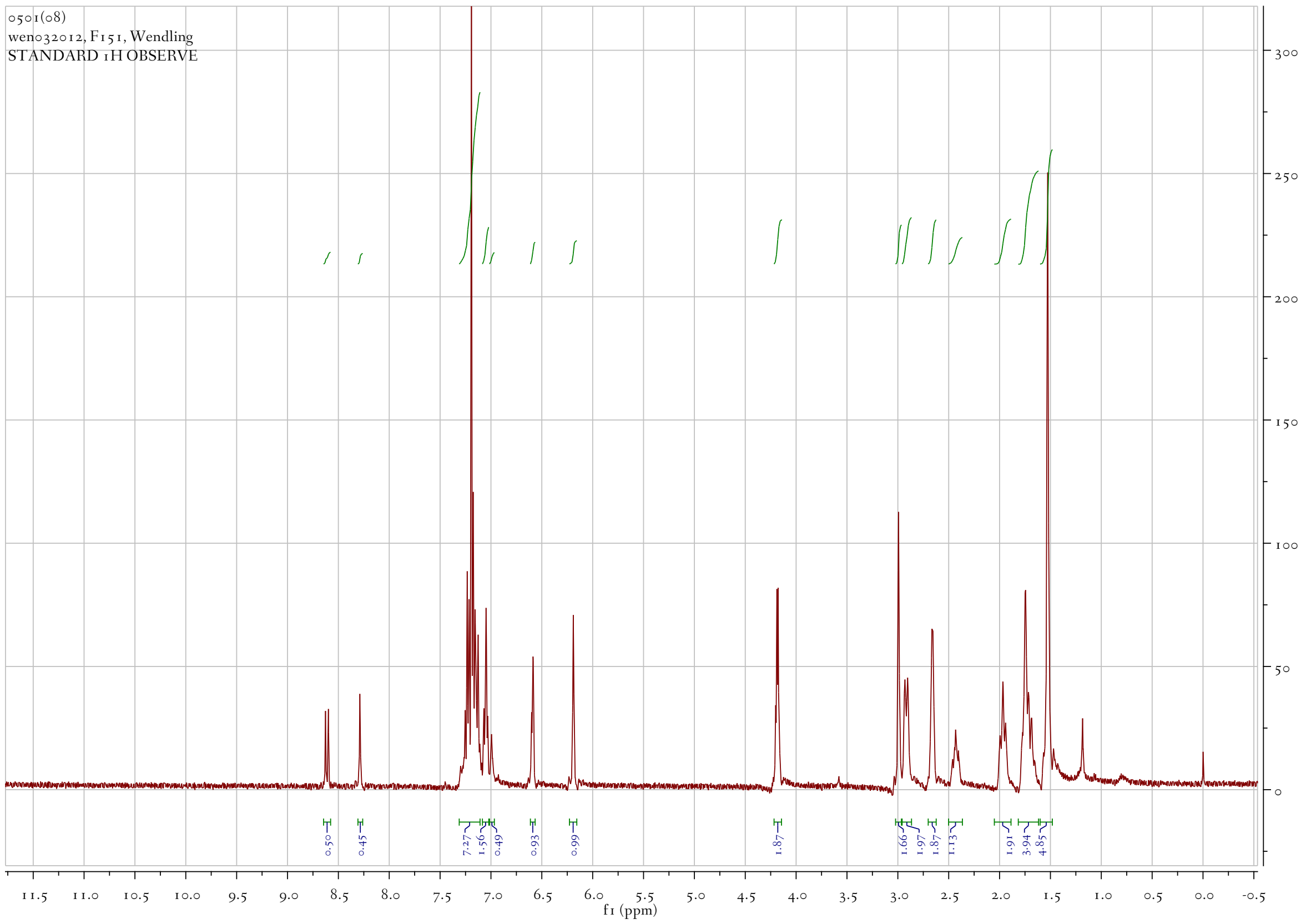

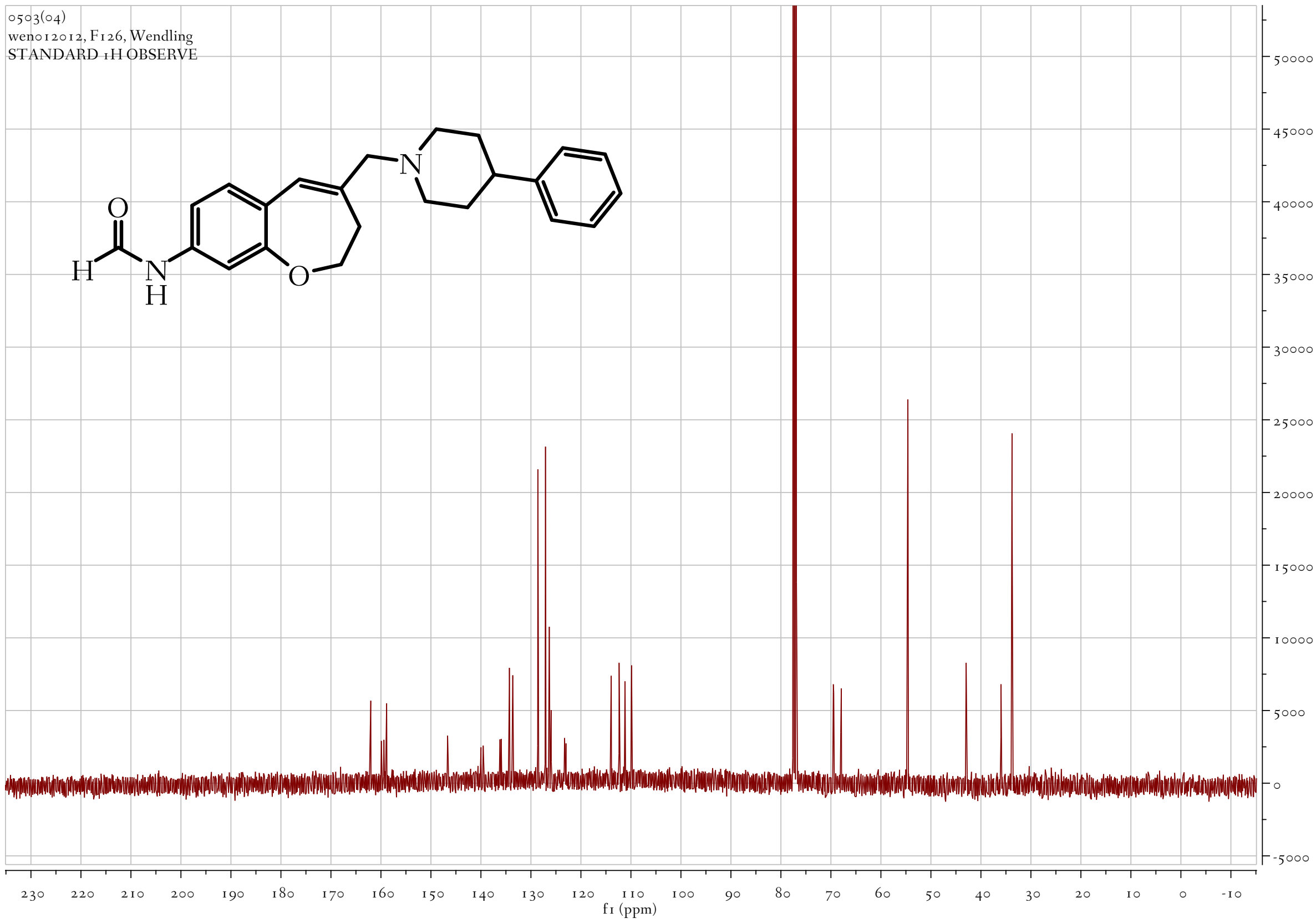
0503(04)
wen012012, F126, Wendling
STANDARD 1H OBSERVE
f1 (ppm)
